# Excessive mechanical strain accelerates intervertebral disc degeneration by disrupting intrinsic circadian rhythm

**DOI:** 10.1038/s12276-021-00716-6

**Published:** 2021-12-21

**Authors:** Sheng-Long Ding, Tai-Wei Zhang, Qi-Chen Zhang, Wang Ding, Ze-Fang Li, Guan-Jie Han, Jin-Song Bai, Xi-Lei Li, Jian Dong, Hui-Ren Wang, Li-Bo Jiang

**Affiliations:** 1 Department of Orthopedic Surgery, Zhongshan Hospital, Fudan University, 200032 Shanghai, China; 2 Department of Orthopedic Surgery, Minhang Hospital, Fudan University, 201100 Shanghai, China; 3 Department of Orthopedic Surgery, Qianjiang Central Hospital of Chongqing, 409000 Chongqing, China

**Keywords:** Osteoarthritis, Cytoskeleton, Ageing

## Abstract

Night shift workers with disordered rhythmic mechanical loading are more prone to intervertebral disc degeneration (IDD). Our results showed that circadian rhythm (CR) was dampened in degenerated and aged NP cells. Long-term environmental CR disruption promoted IDD in rats. Excessive mechanical strain disrupted the CR and inhibited the expression of core clock proteins. The inhibitory effect of mechanical loading on the expression of extracellular matrix genes could be reversed by BMAL1 overexpression in NP cells. The Rho/ROCK pathway was demonstrated to mediate the effect of mechanical stimulation on CR. Prolonged mechanical loading for 12 months affected intrinsic CR genes and induced IDD in a model of upright posture in a normal environment. Unexpectedly, mechanical loading further accelerated the IDD in an Light-Dark (LD) cycle-disrupted environment. These results indicated that intrinsic CR disruption might be a mechanism involved in overloading-induced IDD and a potential drug target for night shift workers.

## Lower back pain: Mechanical strain exacerbated by night shift work

Working long shifts at times when the body should be at rest can have lasting effects on the intervertebral discs in the back, leading to chronic pain. Night shift workers are susceptible to developing certain health conditions because of chronic disruption to their circadian rhythms. Now, Li-Bo Jiang at Zhongshan Hospitial, Fudan University in Shanghai and co-workers across China have uncovered a link between circadian rhythm disruption and intervertebral disc degeneration. In experiments on human tissue samples and rat models, the team found that oscillation of the expression of clock-related genes and proteins was reduced in severely degenerated disc cells. Cellular clock mechanisms were disrupted in disc cells that had been repeatedly placed under mechanical strain at night. This disruption appears to influence degradation of the extracellular matrix, which the team believe may in turn accelerate disc degeneration.

## Introduction

Treatment of intervertebral disc degeneration (IDD), which is the main cause of low back pain (LBP), remains a challenge for doctors^1,2^. LBP causes a heavy medical and economic burden because nearly 80% of the global population has experienced LBP in their lifetime^3–5^. It has been reported that genetic and environmental factors affect the IDD process^3^. Mechanical loading applied to an intervertebral disc (IVD) due to prolonged standing and obesity has been widely regarded as an important cause of both biological and structural changes^6^.

An IVD is a flexible joint between vertebral bodies that absorbs mechanical loading^6^. Excessive loading disrupts cellular and metabolic homeostasis, triggering a cascade of cell-mediated responses^7^. IVDs consist of residing nucleus pulposus (NP) cells, annulus fibrosus (AF) cells and end plate chondrocytes. In healthy IVDs, the height of NP tissue decreases when fluid flows to regions of low pressure due to compressive stimulation, leading to an increase in the osmolarity of the NP tissue^8,9^. In long-term irregular and/or excessive loading, notochordal cells change to chondrocyte-like cells in the NP tissue, transitioning from translucent fluidic tissue to soft amorphous tissue with accompanying IDD.

Emerging evidence indicates that mechanical loading has its own circadian rhythm (CR) in IVDs. During the daytime, IVDs undergo high-level loading during both standing and activity and undergo low-level pressure loading when lying down for rest^9^. The CR system includes the central and peripheral circadian clocks and acts as a time-measuring device organizing physiological activity in 24-h cycles. The central oscillator, in the suprachiasmatic nucleus (SCN), coordinates the peripheral oscillator, which adapts to external rhythmic changes, including light-dark (LD) changes. The clock system, with an autonomous molecular cycle, has two interlocking transcription-translation feedback loops (TTFLs), which are mainly driven by the circadian locomotor output cycles kaput (CLOCK) and brain and muscle ARNT-like protein-1 (BMAL1) complex and are repressed by the period (PER) and cryptochrome (CRY) complex. Disruption of the circadian clock has been found in musculoskeletal diseases. Osteoarthritis is induced by the disruption of environmental CR^10^, and the intrinsic circadian clock has been shown to be a factor in IVDs^11^. However, our previous review showed that the evidence supporting the effect of CR in IDD remains deficient^12^, and direct evidence linking mechanical stimulation to CR in IVDs has never been reported.

In summary, this is the first study to demonstrate a new clue for mechanics-chemistry interactions. Second, the present study shows that excessive mechanical loading affects CR genes, inhibits extracellular matrix (ECM) production and reduces cellular viability, leading to an increased risk of IDD.

## Materials and methods

### Reagents

The reagents are listed in Supplementary Data 1.

### Patient specimen collection

Human research ethics approval was obtained from Zhongshan Hospital Fudan University with informed consent forms signed by the patients. The work described in the paper involving humans was carried out in accordance with The Code of Ethics of the World Medical Association (Declaration of Helsinki)^13^. For histological analysis, NP specimens were collected from 21 patients with IDD or normal IVDs, as determined by magnetic resonance imaging (MRI) and according to the Pfirrmann classification (Supplementary Fig. 1)^14^. For mRNA sequencing, NP specimens were collected from 9 patients. Detailed patient information used for the histological assay is provided in Supplementary Table 1, and the mRNA sequences are listed in Supplementary Table 2.

### Establishment of a punctured IDD rat model

Animal experiments were performed according to a protocol approved by the Animal Experimentation Committee of Zhongshan Hospital, Fudan University. According to our previous study^15^, briefly, a portable X-ray machine was used to check the IVD level between the 6th and 7th caudal vertebrae (Co_6–7_ level). A 21-gauge needle was inserted into the center of the NP tissue through the AF tissue at the Co_6–7_ level, rotated 180° and maintained for 5 s. The Co_7–8_ level was used as the control. Six 3-month-old male SD rats weighing approximately 150‒200 g were randomized into control and 8-week groups with three rats in each group. Eight weeks after the operation, the punctured Co_6–7_ discs were resected and analyzed for histological analysis.

### Aged IVD rat model and tissue collection

One-, twelve-, and twenty-four-month-old male SD rats with 24 rats in each group were housed in groups at 20 ± 5 °C (55 ± 5% humidity) with a normal 12:12 LD cycle and free access to standard chow and water before the 4-week experimental intervention. Then, they were sacrificed in complete darkness using an infrared viewer. Twelve discs with four discs from each rat (L_1-5_) were harvested at 4-h intervals for use in real-time PCR, and three discs (L_5-6_) were harvested for histological staining. All tissues were either snap frozen in liquid nitrogen or freshly used. The circadian time (CT) corresponds to the administration of light in the animal room. CT0 indicates the lights being turned on, whereas CT12 indicates the light being turned off.

### Histological evaluation and immunohistochemistry

Rat and human tissues were stained with hematoxylin and eosin (HE), Alcian blue, or safranin-O and used for immunochemistry. The primary antibodies are shown in Supplementary Table 3. The extent of human IDD was determined according to previously published criteria^16,17^ (Supplementary Table 4), and the detailed classification information is shown in Supplementary Table 1. The evaluator was blinded to the group of samples. In each group, we randomly selected 6 regions of interest and then counted the percentage of positive cells in each area.

### NP cell culture and identification

Primary NP cells were collected from the IVDs (L_1-5_) of 4-week-old SD rats and cultured as we described previously^18^. In brief, NP tissue was isolated from each lumbar disc under a dissecting microscope (Supplementary Fig. 2a). Next, the tissues were digested with 0.25 mg/ml type II collagenase (Invitrogen, Carlsbad, CA, USA) at 37 °C for 4 h. The cells in the second passage were used for subsequent experiments. The NP cell phenotype was confirmed by using collagen II immunohistology, Alcian blue staining and toluidine blue staining (Supplementary Fig. 2b).

### RNA sequencing (RNA-seq) analysis

Total RNA was isolated from tissue samples or NP cells using TRIzol reagent (Takara Biochemicals, Tokyo, Japan). The rRNA was removed using a Ribo-Zero rRNA removal kit (Illumina, USA), and then, the RNA libraries were constructed with a TruSeq Stranded Total RNA Library Prep kit (Illumina, USA). Ten pM libraries were denatured into single-stranded DNA, captured on Illumina flow cells, amplified in situ as clusters and finally sequenced for 150 cycles on an Illumina HiSeq sequencer. High-quality reads were aligned to the reference genome (UCSC hg19) based on an Ensembl GTF gene annotation file with HISAT2 software (v2.04). Then, Cuffdiff software (part of Cufflinks v2.2.1) was used to obtain the fragments per kilobase of exon per million fragments mapped (FPKM) as the expression profile of the mRNA, and fold changes and *P*-values were calculated based on FPKM. The gene expression files were analyzed with the DESeq package. Differentially expressed genes (DEGs) were identified as those with a false-discovery rate (FDR) < 0.05, a fold-change over 2.0 and FPKM ≥ 0.1 in at least one group. ClusterProfiler was used for Gene Ontology (GO) term enrichment analysis, and FDR was used as a multiple comparison method. Heat maps and bubble plots were generated with the pheatmap.

### Cell senescence assay

NPCs were cultured for a second passage, and then Dulbecco’s modified Eagle medium (DMEM) was replaced with medium containing 6, 12, or 18 µg/µl D-gal to induce senescence. The negative control group was grown in normal DMEM, and the positive control group was cultured in normal medium containing an equal volume of PBS. Cells were placed on BIOFlex plates at 30 × 10^4^ cells/well, and the NPCs were cultured to P4. SA-β-gal staining was performed according to the protocol provided by the manufacturer of an SA-β-gal staining kit (#9860; Cell Signaling Technology, MA, USA). The number of SA-β-gal-positive cells in nine random fields per well was counted using an inverted microscope (Olympus Corporation, Japan).

### Cell viability assay

After the NP cells in each Flexcell well were subjected to 18% CTS at 0.2 Hz, CCK-8 solution diluted with DMEM to 1:10 was added. After a 2-h incubation at 37 °C, 100 μl of the DMEM mixture was collected and added to a 96-well plate. Six wells in parallel were used for each test group, as was a blank group (treated with an equal volume of culture medium containing no cells or sodium metasilicate). Then, the OD value was measured at 450 nm using a microplate reader, and the viability rate was calculated with the following formula: viability rate (%) = (OD treatment group − OD blank group) = (OD control group − OD blank group) × 100%.

### Quantitative reverse transcription PCR (qRT–PCR)

Total RNA was isolated from NP samples or cultured NP cells using TRIzol reagent (Takara Biochemicals, Tokyo, Japan). cDNA was synthesized from 1 µg of total RNA through reverse transcription using PrimeScript RT reagent kits (Takara Biochemicals, Tokyo, Japan), and real-time PCR was performed using a SYBR Green qRT–PCR kit (Takara Biochemicals, Tokyo, Japan) according to the manufacturer’s instructions. All the primers used are listed in Supplementary Table 5.

### Western blot analysis

Total protein was measured with radioimmunoprecipitation assay buffer (RIPA) and a BCA protein assay kit (Beyotime, China). The lysates were centrifuged, subjected to sodium dodecyl sulfate-polyacrylamide gels and transferred to polyvinylidene fluoride membranes (Bio–Rad, CA, USA) using a wet-spinning method. The membranes were blocked and incubated overnight with primary antibodies (details are provided in Supplementary Table 3) at 4 °C, followed by incubation with secondary antibodies for 1 h at room temperature. The blots were developed on an imaging system (PerkinElmer, Inc., Waltham, MA, USA).

### Application of cyclic tensile strain (CTS) to cultured NP cells

NP cells were seeded on 6-well flexible silicone membrane BIOFlex™ plates coated with type I collagen (Flexcell International Corp., McKeesport, PA, USA) at a density of 1 × 10^5^ cells/well. After reaching 70–80% confluence, the cells were stretched using an FX-5000T Flexcell tension plus system (Flexcell International Corp., USA) with 10% elongation (0.5 Hz sinusoidal waveforms) for 24 h and with 18% elongation (0.2 Hz) for 24 h, respectively. Then, the cells were cultured for another 24 h and collected at the same intervals based on the time points shown in Supplementary Fig. 3. The cells that remained static served a control. The morphology of the cells was observed and imaged using a phase contrast microscope (Olympus, Tokyo, Japan).

### Immunofluorescence microscopy

After treatment, 1 × 10^5^ NP cells in a 6-well plate were fixed, permeabilized and blocked with 2% BSA for 30 min at room temperature and incubated overnight with primary antibodies (Supplementary Table 3) at 4 °C. Then, the cells were incubated in goat anti-rabbit Alexa Fluor Plus 488, Alexa Fluor Plus 594 (Thermo Fisher Scientific) and Cy3-labeled antibodies (Beyotime, China) for 1 h at room temperature and stained with DAPI for 5 min or Alexa Fluor 488 phalloidin (1:100, Thermo Fisher Scientific, #A12379) for 30 min. Finally, the cells were visualized under a fluorescence microscope (Nikon, Tokyo, Japan).

### Transfection

NP cells (2 × 10^5^ per well) were transfected with 50 nM *RhoA* siRNA (Supplementary Table 6, Ribo Life Science, China) or a 2.5 μg/ml pCMV-BMAL1-3×Flag-copGFP plasmid (Ribo Life Science, China) using Lipofectamine 3000 (Invitrogen, USA) for 48 h according to the manufacturer’s protocol. The effect of siRNA was confirmed by Western blotting and qRT–PCR (Supplementary Fig. 4).

### Circadian rhythm disorder and upright models

Forty-eight 3-week-old male SD rats were randomly divided into four groups: a nonshifted crawl group, nonshifted upright group, shifted crawl group and shifted upright group.

To generate the upright model, the operational procedures described in previous studies were used^19,20^. Briefly, a 2-cm longitudinal incision was made laterally at the proximal third of the forelimbs to expose the deltoid vascular nerve bundles, which were ligated with silk thread. Then, the humerus, muscles, blood vessels and nerves were severed, and the incision was sutured. All animals were initially placed in ordinary cages under 12:12 LD cycles for 1 week and then moved into custom-made cages (65 cm long, 40 cm wide and 35 cm high) with an adjustable food bucket and water bottle, forcing the lumber discs to support the rhythmic loading caused by the rats standing to take in food and water (Supplementary Fig. 5). We recorded the behavior of the rats for 15 min with a camera to record the time spent in the upright position (with their forelegs completely suspended while standing on their hind limbs).

For the CR disorder model (shifted groups), the rats were placed under 24:24 LD cycles for 3 weeks; then, the light time was transferred into a conventional 12:12 LD for 1 week to prevent the animals from adapting the rhythm. The nonshifted group was placed under constant 12:12 LD. The rats were sacrificed 9 and 12 months after the operation, and lumbar IVDs were collected for Western blotting, qRT–PCR and immunochemistry assays.

### Radiography and MRI examination

The rats were placed laterally on a radiographic imaging unit (GE Mammography DMR 18 × 24 Bucky). A disc height index (DHI) was measured as described previously^21^. Briefly, we calculated the DHI by taking the width of one-quarter, central, and three-quarter portions of the end plate on both sides and dividing these values by the mean of the adjacent vertebral body heights obtained at the same locations in the sham rats^18^: DHI% = treated group DHI/sham group DHI × 100% (Supplementary Fig. 6). ImageJ 1.43 software (National Institutes of Health, Bethesda, MD, USA) was used to analyze the DHI.

A 7.0-T magnetic resonance (MR) system (Philips Intera Achieva 7.0 MR) was used to obtain sagittal T2-weighted images at 3, 6, 9, and 12 months after surgery. The MRI scans were evaluated for consensus by two radiologists according to Pfirrmann grades^14^. The quantitative T2 values were calculated as described previously^22^. The regions of interest of the IVDs included the NP, the AF set in a standardized manner, and the relative T2 value, which was equal to the T2 value for the ROI area/T2 value of the vertebral muscle. A color map was generated from the resulting T2 mapping scans.

### Statistical analyses

All data analyses were performed using GraphPad Prism software (version 8.0.2, San Diego, CA). The data are presented as the means ± standard deviations. The Shapiro–Wilk (W) test was used to analyze normality. Student’s t tests (two-tailed) were used for two group comparisons, and one-way ANOVA with Tukey’s multiple comparison test was used to analyze comparisons among multiple groups. *P* < 0.05 was considered to be significant. CircWave v1.4 software (Roelof Hut, Groningen, www.enclock.org) was used to analyze the oscillations of Bmal1, Clock, and Per1 by applying Fourier curve fit analysis.

## Results

### Downregulated expression of clock-related genes and proteins in degenerated NP tissues

**Fig. 1:**
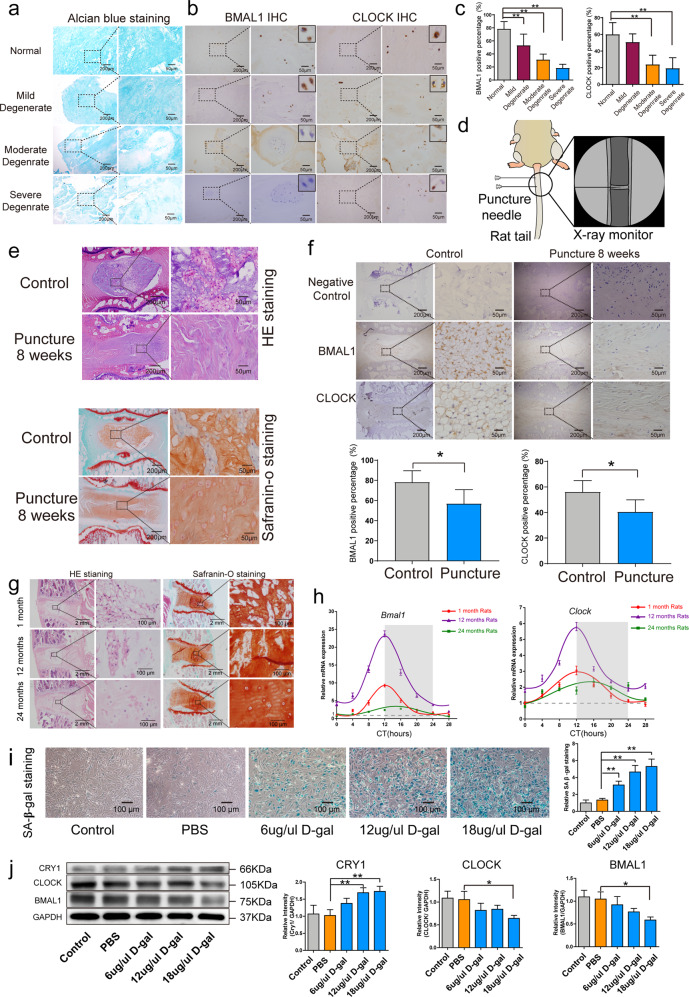
Downregulation of circadian-clock genes in NP tissue. **a** Typical images of Alcian blue-stained sections showing degenerated human NP tissue. **b** Immunohistochemistry assay of BMAL1 and CLOCK proteins in human NP tissue. **c** The percentage of cells with positive BMAL1 and CLOCK expression. Data are shown as the means ± SD, **P < 0.01. normal = 4, mild degeneration = 7, moderate degeneration = 6, severe degeneration = 4. Mann–Whitney *U*-test with Dunn’s multiple comparison test. **d** Illustration of the puncture model in an IDD in an SD rat. **e** IDD was shown by HE and safranin-O staining. **f** Immunohistochemistry assay showing BMAL1 and CLOCK in the NP tissue of IDD rats. The percentage of cells with positive BMAL1 and CLOCK expression. Data are shown as the means ± SD, **P < 0.01. *n* = 3. Unpaired Student’s *t*-test. **g** HE and safranin-O staining of NP tissue from 1-month-, 1-year-, and 2-year-old rats; *n* = 3 rats; *n* = 3 rats. **h** Rhythmic expression of *Bmal1* and *Clock* mRNA in 1-month-, 1-year-, and 2-year-old rat NP cells. Transcripts were detected by real-time qPCR over a period of 28 h; *n* = 3 rats. The gray panels indicate the nighttime (dark) periods. Data are superimposed with corresponding sine waves fitted through CircWave analyses. **i** NP cells were treated with D-gal to induce senescence. The percentage of SA-β-gal-positive cells is shown as the mean ± SD, **P < 0.01. *n* = 3 test. One-tailed ANOVA with Tukey’s multiple comparison test. **j** Western blot analyses of BMAL1, CLOCK and CRY1 protein expression in D-gal-treated NP cells. The relative intensity is presented as the mean ± SD, *P < 0.05, **P < 0.01. *n* = 3. One-tailed ANOVA with Tukey’s multiple comparison test.

The human discs were identified by MRI examination (Supplementary Fig. 1), and the degeneration degree was determined by Alcian blue (Fig. 1a) and HE staining (data not shown). The immunohistochemistry assay results showed an IDD degree-dependent decrease in BMAL1 and CLOCK expression (Fig. 1b, c and Supplementary Fig. 7). Compared with the percentages in normal NP cells, the percentage of BMAL1- and CLOCK-positive cells was significantly decreased in severely degenerated NP tissues. Furthermore, a punctured IDD model was also used to observe BMAL1 and CLOCK expression (Fig. 1d). HE and safranin-O staining demonstrated degeneration 8 weeks after the operation (Fig. 1e). Consistent with the results of the human NP tissues, the degenerated rat NP tissues showed a significantly lower percentage of BMAL1- and CLOCK-positive NP cells (Fig. 1f).

### CR disruption in the aged rat NP cells

HE and safranin-O staining revealed structural changes and proteoglycan decreases in the NP tissues of 24-month-old rats (Fig. 1g). In 1-month-old NP cells and tissues, PER2, CRY1, CLOCK and BMAL1 protein expression was detected (Supplementary Fig. 8a, b). A time-series transcript qPCR study showed a slight rhythmic fluctuation in *Bmal1* expression (as measures of circadian-clock rhythmicity) in NP cells; however, spontaneous robust circadian rhythmicity was observed after dexamethasone synchronization, with peak ZT12 expression that was approximately 2.85-fold greater than the oscillation amplitude of the control (Supplementary Fig. 8c).

Compared with that of the NP tissues in the 1-month-old rats, the amplitude of the circadian oscillation was higher in 12-month-old rats and lower in 24-month-old rats (Fig. 1h). In addition, the expression of *Bmal1* and *Clock* mRNA was higher in the 12-month-old rats than in the 1-month-old or 24-month-old rats at each time point (Fig. 1h). A senescent NP cell model was generated with D-gal treatment (Fig. 1i). After D-gal treatment, expression of the BMAL1 and CLOCK proteins was inhibited (Fig. 1j), suggesting that the autonomous circadian clock in NP tissue was dysregulated and suppressed with aging.

### Excessive mechanical strain disrupts the CR of NP cells

**Fig. 2:**
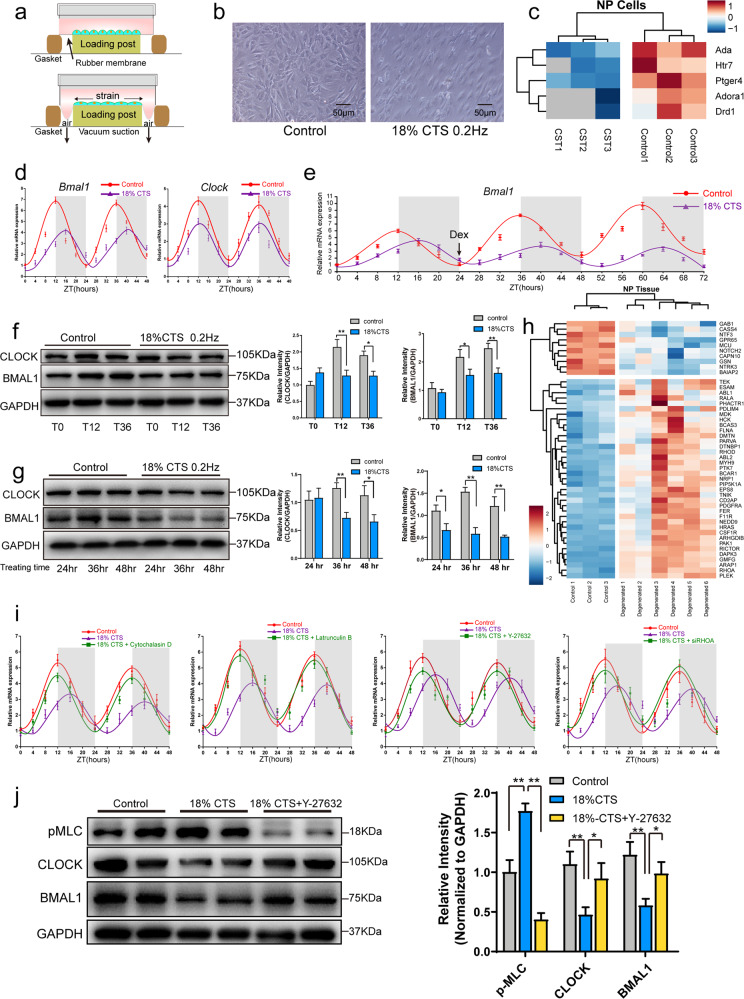
Excessive mechanical loading results in disruption of the circadian clock by the Rho/ROCK pathway. **a** Schematic diagram of the FX-5000 Flexcell system. **b** Morphology of the NP cells under cyclic tensile strain (CTS). **c** Heat map of the Gene Ontology (GO) analysis indicating the involvement of negative regulation of circadian rhythm under CTS. *n* = 3. **d** Rhythmic expression of *Bmal1* and *Clock* mRNA in 18% CTS-treated synchronized NP cells. Transcripts were detected by real-time qPCR over a period of 48 h; *n* = 3. Data were superimposed with corresponding sine waves fitted through CircWave analyses. **e** Rhythmic expression of *Bmal1* mRNA in 18% CTS-treated NP cells. Transcripts were detected by real-time qPCR over a period of 72 h; *n* = 3 Data were superimposed with corresponding sine waves fitted through CircWave analyses. **f** Western blot analyses for BMAL1 and CLOCK protein in 18% CTS-treated synchronized NP cells. The relative intensity is shown as the mean ± SD, *P < 0.05, **P < 0.01. *n* = 3. Unpaired Student’s *t*-test. **g** Western blot analyses for BMAL1 and CLOCK protein in 18% CTS-treated NP cells at different times. The relative intensity is shown as the mean ± SD, *P < 0.05, **P < 0.01. *n* = 3. Unpaired Student’s *t*-test. **h** RNA-sequence analysis shows the changed expression of actin cytoskeleton reorganization BP-related genes in human degenerated NP tissues. **i** Rhythmic expression of *Bmal1* mRNA in synchronized NP cells under 18% CTS with or without cytochalasin D, latrunculin B, Y-27632 treatment or *RhoA* siRNA transfection. Transcript levels were measured by real-time qPCR over a period of 48 h; *n* = 3. Data were superimposed with corresponding sine waves fitted through CircWave analyses. **j** Western blot analyses of pMLC, CLOCK and BMAL1 protein expression in NP cells under 18% CTS with or without Y-27632 treatment. The relative intensity is shown as the mean ± SD, *P < 0.05, **P < 0.01. *n* = 3. One-tailed ANOVA with Tukey’s multiple comparison test.

The cultured NP cells were treated with 10% CTS at 0.5 Hz or 18% CTS at 0.2 Hz for 24 h (Fig. 2a, b and Supplementary Fig. 9). Under 10% CTS treatment, time-series qPCR analysis confirmed that the amplitude of *Bmal1* and *Per2* expression after synchronization was not changed (Supplementary Fig. 10a). CTS at 10% increased *Col2a1* expression in NP cells but did not affect *Aggrecan*, *Mmp1* or *Adamts4* expression (Supplementary Fig. 10b). In addition, the rhythmicity of *Col2a1*, *Aggrecan* and *Mmp1* mRNA expression was observed (Supplementary Fig. 10b).

In contrast, RNA sequencing analysis showed that, among all 1419 DEGs, 5 genes were of negative regulation of circadian rhythm Biological Process (BP) of GO (Fig. 2c) and 28 genes were involved in the circadian rhythm BP in the GO analysis (Supplementary Fig. 11 and Supplementary Table 7). In addition, 18% CTS significantly inhibited cell proliferation and reduced the oscillation amplitude of *Bmal1* and *Clock* mRNA expression after synchronization (Fig. 2d), indicating the presence of CR disruption in the excessive strain-treated cells. The NP cells were then incubated with dexamethasone at 24 h after stopping CTS to detect whether the CTS-disrupted circadian clock could be rescued. Time-series qPCR analysis of *Bmal1* mRNA revealed that the amplitude of the circadian oscillation was not changed by dexamethasone treatment (Fig. 2e). We also used Western blotting to detect the expression of the BMAL1 and CLOCK proteins in NP cells treated with CTS. BMAL1 and CLOCK protein expression was significantly reduced by 18% CTS at T12 and T36, and after treatment with 18% CTS for 36 and 48 h, BMAL1 and CLOCK expression was also significantly inhibited (Fig. 2f, g).

### The Rho/ROCK pathway is involved in the effects of excessive mechanical strain on CR

Human NP tissues were analyzed by RNA sequencing (Supplementary Fig. 12). A total of 6172 genes were differentially expressed in the degenerated NP tissues. The GO analysis highlighted the actin cytoskeleton reorganization BP with the mRNA profiles from human degenerated NP, and the expression of 47 genes involved in this BP was significantly changed in the degenerated NP tissues (Fig. 2h and Supplementary Table 8). The RNA sequencing results suggested that the cytoskeleton might convert mechanical strain into cell signaling. Under tension, NP cells showed a spindle morphology compared with the polygonal morphology of the control cells (Fig. 2b). The transducing role of the cytoskeleton was investigated by treating the cells with cytochalasin D and latrunculin B to inhibit actin polymerization. After this disruption to the cytoskeleton, the oscillation amplitude of Bmal1 expression was significantly reversed in the CTS-treated cells after synchronization (Fig. 2i), especially in the latrunculin B-treated cells, indicating the involvement of the cytoskeleton.

The Rho/ROCK pathway, which accounts for actomyosin contractility, was analyzed by treatment with the ROCK1 inhibitor Y-27632 and RhoA siRNA. Y-27632 and Rhoa silencing partly reversed the inhibitory effect of 18% CTS on the oscillation amplitude of Bmal1 expression after synchronization (Fig. 2i). In addition, 18% CTS treatment significantly enhanced pMLC expression, but Y-27632 treatment inhibited pMLC activation and reversed the downregulated expression of the CLOCK and BMAL1 proteins that had been induced by the 18% CTS treatment (Fig. 2j), suggesting the mediation of the Rho/ROCK pathway upon CR disruption.

### CR mediates the effect of excessive mechanical strain on the expression of ECM-related genes and proteins

**Fig. 3:**
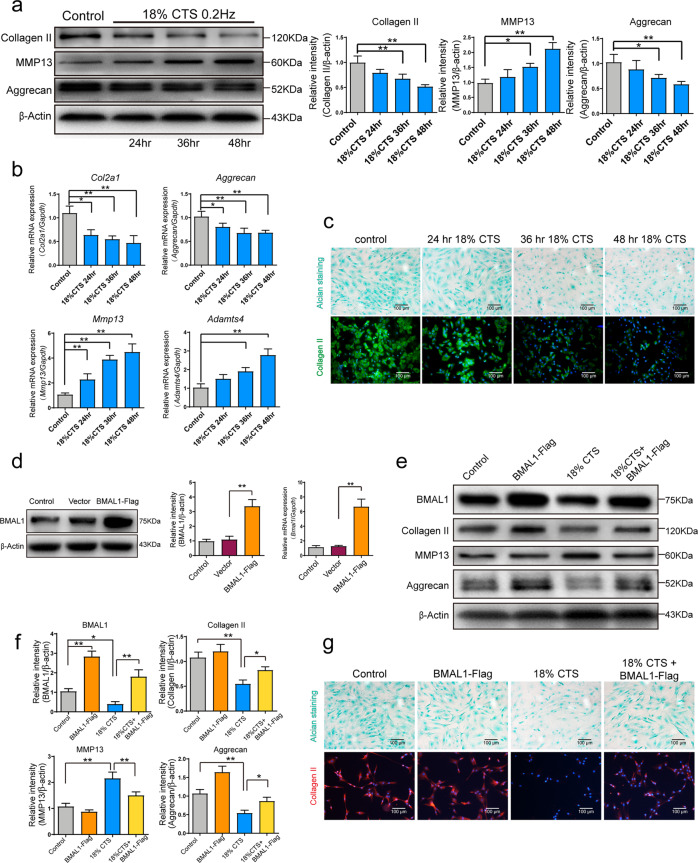
CR mediates the effect of excessive strain loading on the expression of extracellular matrix-related genes through the Rho/ROCK pathway. **a** Western blot analyses of Collagen II, aggrecan and MMP13 protein in 18% cyclic tensile strain (CTS)-treated NP cells. The relative intensity is shown as the mean ± SD, *P < 0.05, **P < 0.01. *n* = 3. One-tailed ANOVA with Tukey’s multiple comparison test. **b** Expression of *Col2Α1*, *Aggrecan*, *Mmp13* and *Adamst4* mRNA in 18% CTS-treated NP cells. Transcripts were detected by real-time qPCR. Data are shown as the mean ± SD, *P < 0.05, **P < 0.01. *n* = 3. One-tailed ANOVA with Tukey’s multiple comparison test. **c** Alcian blue staining and collagen II immunofluorescence study of NP cells under 18% CTS. **d** Western blot analyses of BMAL1 levels in NP cells transfected with an HA-BMAL1-overexpressing vector. The relative intensity is shown as the mean ± SD, *P < 0.05, **P < 0.01. *n* = 3. Unpaired Student’s *t*-test. **e** Western blot analyses of collagen II, aggrecan, MMP13 and BMAL1 protein levels in HA-BMAL1-overexpressing NP cells with or without 18% CTS treatment. The relative intensity is shown in **f** as the mean ± SD, *P < 0.05, **P < 0.01. *n* = 3. One-tailed ANOVA with Tukey’s multiple comparison test. **g** Alcian blue staining and Collagen II immunofluorescence study of NP cells under 18% CTS.

The expression of collagen II and aggrecan was significantly reduced and MMP13 expression increased in response to 18% CTS treatment for 36 and 48 h (Fig. 3a). A time-dependent change in the expression of *Col2a1, Aggrecan, Mmp13 and Adamts4* mRNA was observed in 18% CTS-treated cells (Fig. 3b). Alcian blue staining and collagen II immunofluorescence analysis also revealed that the levels of proteoglycan and collagen II were decreased upon CTS treatment (Fig. 3c), suggesting that CTS inhibited ECM expression.

It has been reported that BMAL1 might be an important link between CR and IDD because loss of BMAL1 in conditional knockout mice led to progressive disc degeneration^11^. However, the exact role of BMAL1 in IDD development requires further investigation. BMAL1 was overexpressed to investigate the involvement of the circadian clock in the effect of CTS on ECM expression. BMAL1 overexpression was confirmed (Fig. 3d), and it reversed the downregulation of collagen II and Aggrecan and the upregulation of MMP13 in the 18% CTS-treated NP cells (Fig. 3e, f). Alcian blue staining and collagen II immunofluorescence analysis also revealed the reversion induced by BMAL1 overexpression (Fig. 3g).

### Prolonged mechanical loading accelerates the effect of environmental CR disruption on IDD in a model of prolonged upright posture

**Fig. 4:**
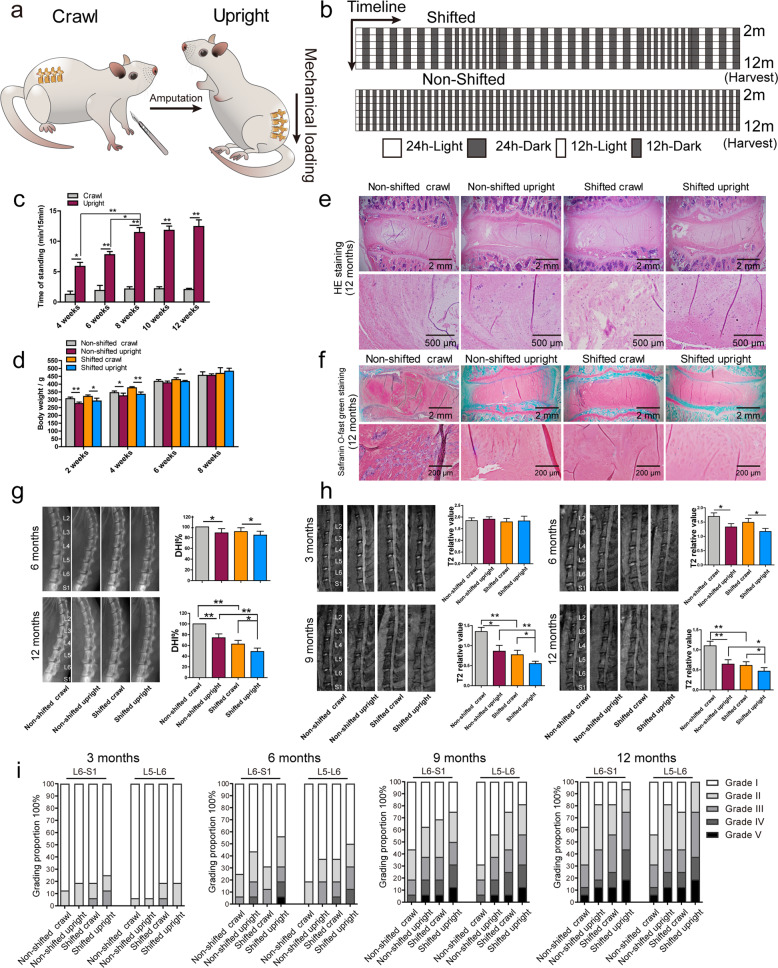
Establishing an IDD model with prolonged upright posture and environmental CR disruption. **a** Illustration showing the animal model of upright posture imitating the standing posture of human beings. **b** Experimental protocol for the environmental disruption of CRs for 12 months. **c** Calculation of the standing time of crawling and upright rats for 15 min. Data are shown as the means ± SD, *P < 0.05, **P < 0.01. *n* = 3 rats. Unpaired Student’s *t*-test. **d** Calculation of the weight of the rats in different groups. Data are shown as the means ± SD, *P < 0.05, **P < 0.01. *n* = 3 rats. One-tailed ANOVA with Tukey’s test. **e** Representative IVD image of HE-stained samples from the nonshifted crawling, nonshifted upright, shifted crawling and shifted upright groups. **f** Representative IVD image of safranin-O-stained tissues. **g** Representative images and DHI scores of the intervertebral space in rat lumbar vertebrae showing the decreased height in the shifted and upright rats at 6 and 12 months after the operation. Data are shown as the mean ± SD, *P < 0.05, **P < 0.01. *n* = 3 rats. One-tailed ANOVA with Tukey’s test. **h** Representative images and relative T2 signals of the lumbar IVDs in rats at 3, 6, 9, and 12 months after the operation. Data are shown as the means ± SD, *P < 0.05, **P < 0.01. *n* = 3 rats. One-tailed ANOVA with Tukey’s multiple comparison test. **i** Grade proportion in different groups according to the Pfirrmann classification. *n* = 3 rats.

To imitate rhythmic loading on human IVDs, a model of prolonged upright posture was established (Fig. 4a). Four weeks later, these rats were divided into an LD cycle shift group (Fig. 4b), mimicking the pathological condition of night shift workers with a high percentage of IDD. Four weeks after amputation, the rats had adapted to the standing posture with a significantly longer standing time (Fig. 4c and Supplementary Fig. 5), becoming stable by Week 8. In addition, the standing rats became thinner in both the nonshifted and shifted LD groups by Weeks 2 and 4 after the operation (Fig. 4d).

Twelve months after LD cycle disruption, the loss of demarcation and NP proteoglycan, fissures in the NP and AF tissues, and cell cluster formation could be observed in the discs of the nonshifted upright and shifted crawling rats (Fig. 4e, f). Importantly, compared with the nonshifted crawling rats, the shifted crawling rats showed increased signs of degeneration, indicating that long-term disruption of the CR induced IDD. The comparison became more obvious in the shifted standing rat discs with decreased proteoglycan content, suggesting that mechanical stress might accelerate this degeneration.

X-ray imaging and DHI calculation demonstrated significantly decreased disc heights in the nonshifted and shifted upright rats at 6 months after the shift compared with the matched crawling rats (Fig. 4g). However, the shifted rat discs showed no obvious decrease in DHI, indicating that CR might require a longer time to affect IDD than mechanical factors. By Month 12, the DHI of the shifted crawling and upright rats was significantly less than that of the matched nonshifted rats, with a particularly obvious difference in the shifted upright group, suggesting that the synergistic effect of CR and mechanical loading accelerated IDD. However, no clear calcification or osteophytes was observed, implying that the stimulation in this model was relatively mild and thus similar to clinicopathological change. By Month 3 after the shift, the differences in the T2 signals were not obvious in the MRI images of the lumbar discs, and the MRI evidence was consistent with that of the X-ray images obtained at Month 6 (Fig. 4h and Supplementary Fig. 13). Nine and 12 months after the shift, we observed a decrease in the T2 signals of the nonshifted upright, shifted crawling, and shifted upright rats. Surprisingly, in the shifted upright group, almost every disc showed a low T2 signal. Statistically, the T2 signal in the shifted upright groups was significantly less than that in the nonshifted upright and shifted crawling groups. In addition, a higher percentage of grade V IVDs and a lower percentage of grade I discs were observed in the shifted upright groups (Fig. 4i).

**Fig. 5:**
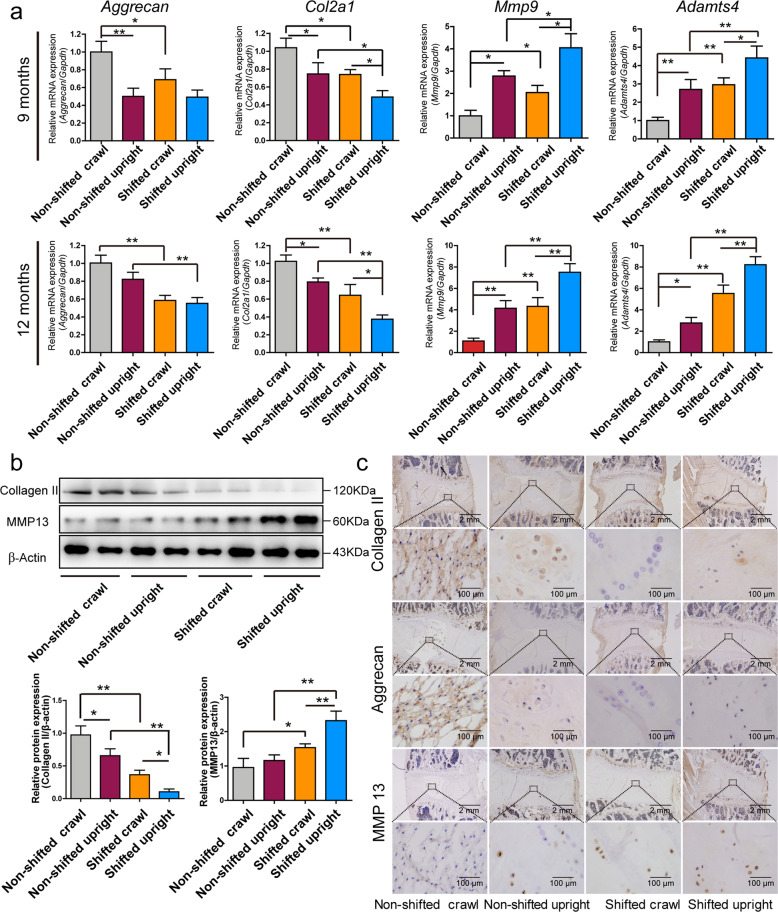
Expression of extracellular matrix-related genes and proteins in the NP tissues of different rat groups. **a** Expression of extracellular matrix-related genes in NP tissue from rat lumbar discs at 9 and 12 months after the operation. Data are shown as the means ± SD, *P < 0.05, **P < 0.01. *n* = 3 rats. One-tailed ANOVA with Tukey’s test. **b** Expression of collagen II and MMP12 proteins in the NP tissue of rat lumbar discs at 12 months after the operation. Data are shown as the mean ± SD, *P < 0.05, **P < 0.01. *n* = 3 rats. One-tailed ANOVA with Tukey’s test. **c** Immunohistochemistry for collagen II, aggrecan and MMP13 in the NP tissue of rat discs at 12 months after the operation.

The expression of ECM-related genes and proteins was analyzed. Nine and 12 months after the shift, the mRNA expression of *Aggrecan* and *Col2a1* was significantly reduced in the shifted crawling and shifted upright rats, and the standing posture also inhibited the expression of these mRNAs (Fig. 5a). In contrast, mRNA expression of *Mmp9* and *Adamst4* was significantly enhanced by CR disruption and mechanical loading. Compared with the shifted crawling group, Western blotting and immunohistochemistry results demonstrated a decrease in collagen II and an increase in MMP13 protein expression that was induced in the shifted upright group at 12 months after the shift (Fig. 5b, c). These results demonstrated that mechanical loading accelerated degradation of the ECM induced by circadian-clock disruption.

### Prolonged mechanical loading affects CR genes

**Fig. 6:**
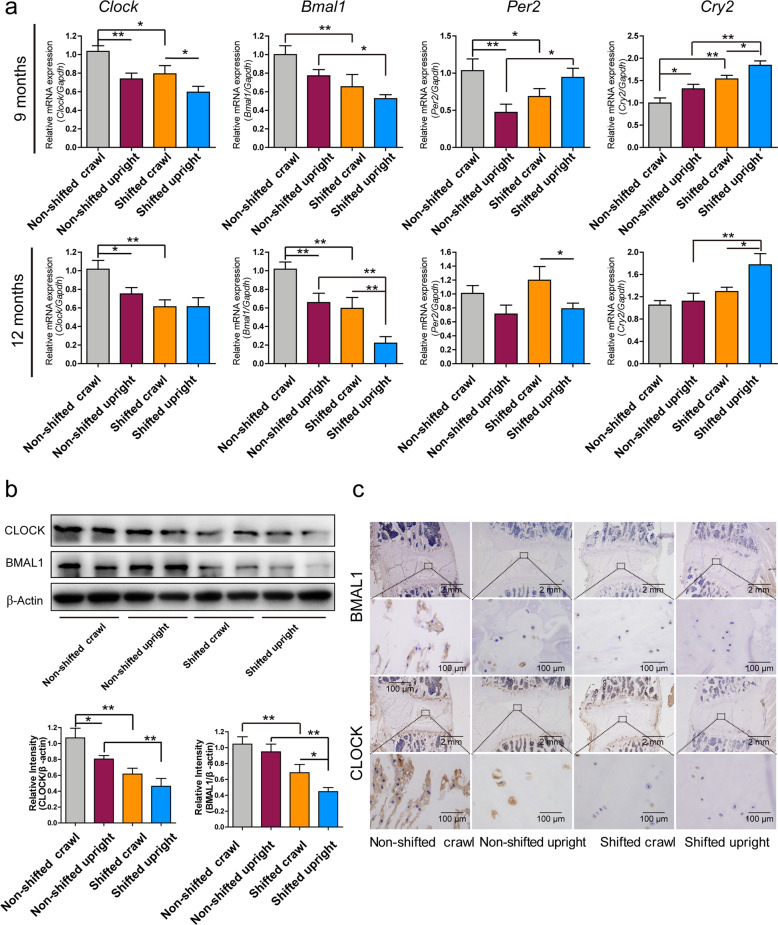
Expression of CR-related genes and proteins in NP tissues of the different rat groups. **a** Expression of CR-related genes in the NP tissue of rat lumbar discs at 9 and 12 months after the operation. Data are shown as the mean ± SD, *P < 0.05, **P < 0.01. *n* = 3 rats. One-tailed ANOVA with Tukey’s test. **b** BMAL1 and CLOCK protein expression in the NP tissues of rats in the nonshifted crawl, nonshifted upright, shifted crawl and shifted upright groups. Data are shown as the mean ± SD, *P < 0.05, **P < 0.01. *n* = 3 rats. One-tailed ANOVA with Tukey’s test. **c** Representative images of immunohistochemistry for BMAL1 in NP tissues.

Unsurprisingly, environmental disruption of CR inhibited the mRNA and protein expression of CLOCK and BMAL1 and increased *Cry2* mRNA expression in the NP tissue of the IVDS (Fig. 6a, b) at 9 and 12 months after the shift, indicating that the disc was sensitive to the LD change. Standing posture alone, another stimulating factor, also inhibited clock and *Bmal1* mRNA and protein expression and increased *Cry2* mRNA expression. Interestingly, the change in circadian-related genes was significant in the shifted upright group with prolonged standing posture due to the 24:24 LD cycle, suggesting that mechanical loading further affected the CR genes. The immunohistochemical assays of BMAL1 and CLOCK corroborated this finding (Fig. 6c).

## Discussion

**Fig. 7:**
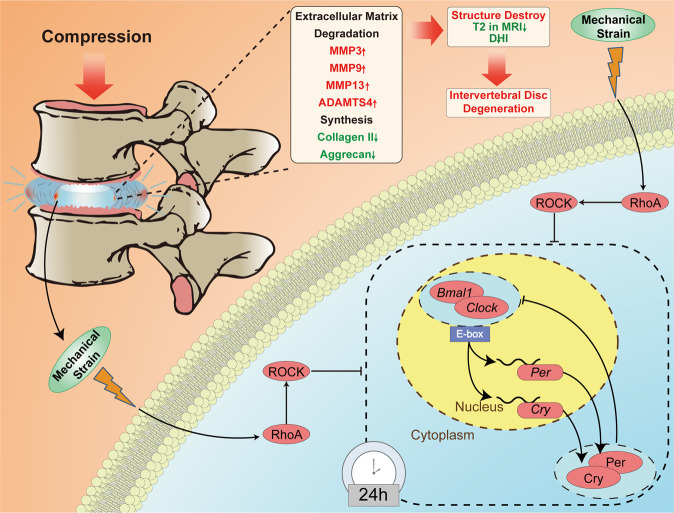
Mechanical stress dampens the CR, resulting in the degeneration of IVDs through the Rho/ROCK pathway. Mechanical stress dampens the CR, resulting in IDD through the Rho/ROCK pathway. Excessive mechanical loading on IVDs influenced the cytoskeleton of the NP cells and activated the Rho/Rock pathway, by which the CR of the NP cells was disrupted. This disruption affected the balanced synthesis and degradation of the ECM and resulted in the destruction of the NP cell structures, a decrease in the T2-weighted signal and a decline in DHI, leading to IDD.

Night shift work was thought to be an independent risk factor for IDD progression in some studies^23^. In this study, we revealed the association between intrinsic CR disruption and IDD in human tissues and a puncture-induced rat model, as well as a circadian oscillation decline in aged IVDs. The intrinsic CR is synchronized with daily environmental changes and the microenvironment^24^. The present research demonstrated that a 12-month LD shift could disturb intrinsic CR and induce IDD in rats, partly explaining the contribution of night shift work to IDD. More importantly, excessive CTS disrupted the CR in NP cells through the Rho/ROCK pathway, and CR disruption might mediate mechanical strain-induced ECM degradation (Fig. 7). Actin cytoskeleton reorganization BP was shown to transduce the mechanical stimulus to chemical signaling. Finally, the upright posture model demonstrated that mechanical loading accelerated IDD, promoted ECM degradation and induced CR disruption in NP tissue.

Physiological mechanical stress can induce anabolism, whereas the pathological magnitude of mechanical stress may cause catabolic events in NP cells^25^. The area change induced by physiological mechanical stress in IVDs is 15%^26,27^. In addition to the intensity of mechanical stress, the frequency of the stress applied to cells should be considered. In this study, the CTS with 10% elongation at 0.5 Hz and 18% CTS at 0.2 Hz were chosen to mimic physical and nonphysical stretching caused by compression, respectively. Cells are intricately connected to the external environment through their cytoskeleton^28^, and mRNA sequencing indicated the potential involvement of the cytoskeleton in this research. The tension generated by the contracting cytoskeleton can be used to sense the mechanical properties of the ECM, which in turn have been shown to affect cytoskeletal organization and cell behavior^28^.

Although mechanically induced IDD animal models have been established, the mechanism is still not clear^29^. Mechanical stress promotes ECM degradation by stimulating the expression of proinflammatory cytokines and catabolic enzymes^29–32^. It is necessary to exclude the influence of proinflammatory cytokines when determining the special role of other pathways in circadian-clock regulation, such as the Rho/ROCK pathway^11^. In this study, we excluded proinflammatory cytokines by directly inhibiting the Rho/Rock pathway with inhibitors of the Rho/ROCK pathway. These inhibitors directly reversed the damping of the circadian clock, suggesting that the Rho/ROCK pathway was directly related to the CR pathway.

The amputated rat model mimicking chronic mechanical loading led to the degradation of ECM and resulted in the degeneration of both lumbar and cervical IVDs^20,33^. An amputated rat model with overloading of IVD under an environment with light/dark disruption was used to simulate night shift workers, whose endogenous rhythm was disturbed. It has been demonstrated that night shift work is a significant risk factor for the development of IDD and its progression^23^. Diurnal shifts in mechanical loading lead to osmotic pressure changes, which is a limitation that we did not research^34,35^. The magnitude and frequency of the stress applied to the IVD of the modeled rats were not investigated either in the past or in this study because the activity of rats was not specifically controlled^19,20^. Previous studies have reported that the metabolism of the disc is not altered at a threshold magnitude of 0.2 MPa and frequency of 0.2 Hz when the disc is treated for only 1 h per day^36^. However, how the magnitude, frequency and duration of loading control anabolism and catabolism is complicated^36^. Our amputated rat model mimicked the cyclical loading process, and the observed progress of degeneration supported the supposition that circadian-clock disruption might participate in the IDD process induced by overloading. Adjusting the magnitude, frequency and duration of loading to change the circadian rhythm of the nucleus pulposus would be a new aspect for further research.

In this study, light shifting was successfully also used to develop an IDD model. Although the IVD is the largest avascular tissue in the human body, regulators from the central circadian clock may permeate the NP tissue through the end plate^37,38^. However, the evidence distinguishing the role of central and peripheral clocks remains deficient, and the exact role of the central clock in IDD progression is still unclear.

In tendon and wound closure, the synthesis of ECM also exhibits a clear CR mediated by BMAL1^39,40^. *Col1* mRNA expression showed no rhythmicity in tendons, but protein synthesis showed CR^39^. Our data revealed the oscillation of the mRNA expression of *Col2α1* and *Aggrecan* in NP cells in 24-h ZT; however, the CR of ECM synthesis in NP tissue remains uninvestigated. The significance of the oscillation of *Col2α1* and *Aggrecan* mRNA expression requires further investigation.

In conclusion, we established an association between IDD and CR disruption. The intrinsic CR might be regulated by environmental and local mechanical stimuli. Excessive mechanical strain dampens the circadian clock in NP cells, and the Rho/ROCK pathway might mediate this effect. The in vivo experiments support the in vitro finding. Thus, our results suggest that mechanical rhythmicity disruption might be the reason for the high prevalence of LBP in night shift workers.

## Supplementary information

Revised supplementary information

## Data Availability

Sequencing data are available in the GEO Database and National Genomics Data Center. Other data that support the findings of this study are available within this article and its Supplementary Information files or from the corresponding author upon reasonable request.

## References

[1] Khan AN (2017). Inflammatory biomarkers of low back pain and disc degeneration: a review. Ann. N. Y. Acad. Sci..

[2] Binch ALA, Fitzgerald JC, Growney EA, Barry F (2021). Cell-based strategies for IVD repair: clinical progress and translational obstacles. Nat. Rev. Rheumatol..

[3] Deyo RA, Mirza SK (2016). CLINICAL PRACTICE. Herniated Lumbar Intervertebral Disk. N. Engl. J. Med..

[4] GBD 2016 Disease and Injury Incidence and Prevalence Collaborators. (2017). Global, regional, and national incidence, prevalence, and years lived with disability for 328 diseases and injuries for 195 countries, 1990-2016: a systematic analysis for the Global Burden of Disease Study 2016. Lancet.

[5] Katz JN (2006). Lumbar disc disorders and low-back pain: socioeconomic factors and consequences. J. Bone Jt. Surg. Am..

[6] Molladavoodi S, McMorran J, Gregory D (2020). Mechanobiology of annulus fibrosus and nucleus pulposus cells in intervertebral discs. Cell Tissue Res..

[7] Adams MA, Roughley PJ (2006). What is intervertebral disc degeneration, and what causes it?. Spine (Philos. Pa 1976).

[8] Yang N (2017). Cellular mechano-environment regulates the mammary circadian clock. Nat. Commun..

[9] Haschtmann D, Stoyanov JV, Ferguson SJ (2006). Influence of diurnal hyperosmotic loading on the metabolism and matrix gene expression of a whole-organ intervertebral disc model. J. Orthop. Res..

[10] Ranjan KC (2015). Environmental disruption of circadian rhythm predisposes mice to osteoarthritis-like changes in knee joint. J. Cell. Physiol..

[11] Dudek M (2017). The intervertebral disc contains intrinsic circadian clocks that are regulated by age and cytokines and linked to degeneration. Ann. Rheum. Dis..

[12] Zhang TW, Li ZF, Dong J, Jiang LB (2020). The circadian rhythm in intervertebral disc degeneration: an autophagy connection. Exp. Mol. Med..

[13] Rickham PP (1964). Human experimentation. code of ethics of the world medical association. Declaration of Helsinki. Br. Med. J..

[14] Pfirrmann CW, Metzdorf A, Zanetti M, Hodler J, Boos N (2001). Magnetic resonance classification of lumbar intervertebral disc degeneration. Spine (Philos. Pa 1976).

[15] Jiang LB (2018). TIGAR mediates the inhibitory role of hypoxia on ROS production and apoptosis in rat nucleus pulposus cells. Osteoarthr. Cartil..

[16] Sive JI (2002). Expression of chondrocyte markers by cells of normal and degenerate intervertebral discs. Mol. Pathol..

[17] Le Maitre CL, Freemont AJ, Hoyland JA (2005). The role of interleukin-1 in the pathogenesis of human intervertebral disc degeneration. Arthritis Res. Ther..

[18] Jiang L, Jin Y, Wang H, Jiang Y, Dong J (2014). Glucosamine protects nucleus pulposus cells and induces autophagy via the mTOR-dependent pathway. J. Orthop. Res..

[19] Goff CW, Landmesser W (1957). Bipedal rats and mice; laboratory animals for orthopaedic research. J. Bone Jt. Surg. Am..

[20] Liang QQ (2008). Prolonged upright posture induces degenerative changes in intervertebral discs in rat lumbar spine. Spine (Philos. Pa 1976).

[21] Yuan FL (2014). Molecular actions of ovarian cancer G protein-coupled receptor 1 caused by extracellular acidification in bone. Int. J. Mol. Sci..

[22] Chen C (2014). Quantitative T2 magnetic resonance imaging compared to morphological grading of the early cervical intervertebral disc degeneration: an evaluation approach in asymptomatic young adults. PLoS ONE.

[23] Elfering A (2002). Risk factors for lumbar disc degeneration: a 5-year prospective MRI study in asymptomatic individuals. Spine (Philos. Pa 1976).

[24] Adamovich Y, Ladeuix B, Golik M, Koeners MP, Asher G (2017). Rhythmic oxygen levels reset circadian clocks through HIF1alpha. Cell Metab..

[25] Bleuel J, Zaucke F, Bruggemann GP, Niehoff A (2015). Effects of cyclic tensile strain on chondrocyte metabolism: a systematic review. PLoS ONE.

[26] Feng C (2018). Cyclic mechanical tension reinforces DNA damage and activates the p53-p21-Rb pathway to induce premature senescence of nucleus pulposus cells. Int. J. Mol. Med..

[27] Zhang YH, Zhao CQ, Jiang LS, Dai LY (2011). Cyclic stretch-induced apoptosis in rat annulus fibrosus cells is mediated in part by endoplasmic reticulum stress through nitric oxide production. Eur. Spine J..

[28] Fletcher DA, Mullins RD (2010). Cell mechanics and the cytoskeleton. Nature.

[29] Wang F, Cai F, Shi R, Wang XH, Wu XT (2016). Aging and age related stresses: a senescence mechanism of intervertebral disc degeneration. Osteoarthr. Cartil..

[30] Cho H, Seth A, Warmbold J, Robertson JT, Hasty KA (2011). Aging affects response to cyclic tensile stretch: paradigm for intervertebral disc degeneration. Eur. Cell Mater..

[31] Gilbert HT, Hoyland JA, Millward-Sadler SJ (2010). The response of human anulus fibrosus cells to cyclic tensile strain is frequency-dependent and altered with disc degeneration. Arthritis Rheum..

[32] Pratsinis H (2016). Cyclic tensile stress of human annulus fibrosus cells induces MAPK activation: involvement in proinflammatory gene expression. Osteoarthr. Cartil..

[33] Liang QQ (2011). Prolonged upright posture induces degenerative changes in intervertebral discs of rat cervical spine. Spine (Philos. Pa 1976).

[34] Massey CJ, van Donkelaar CC, Vresilovic E, Zavaliangos A, Marcolongo M (2012). Effects of aging and degeneration on the human intervertebral disc during the diurnal cycle: a finite element study. J. Orthop. Res..

[35] Sivan S, Neidlinger-Wilke C, Wurtz K, Maroudas A, Urban JP (2006). Diurnal fluid expression and activity of intervertebral disc cells. Biorheology.

[36] Maclean JJ, Lee CR, Alini M, Iatridis JC (2004). Anabolic and catabolic mRNA levels of the intervertebral disc vary with the magnitude and frequency of in vivo dynamic compression. J. Orthop. Res..

[37] Horner HA, Urban JP (2001). 2001 Volvo Award Winner in Basic Science Studies: effect of nutrient supply on the viability of cells from the nucleus pulposus of the intervertebral disc. Spine (Philos. Pa 1976).

[38] Raj PP (2008). Intervertebral disc: anatomy-physiology-pathophysiology-treatment. Pain. Pr..

[39] Chang J (2020). Circadian control of the secretory pathway maintains collagen homeostasis. Nat. Cell Biol..

[40] Kowalska E (2013). NONO couples the circadian clock to the cell cycle. Proc. Natl Acad. Sci. USA.

