# Author Correction: Excessive mechanical strain accelerates intervertebral disc degeneration by disrupting intrinsic circadian rhythm

**DOI:** 10.1038/s12276-022-00726-y

**Published:** 2022-08-15

**Authors:** Sheng-Long Ding, Tai-Wei Zhang, Qi-Chen Zhang, Wang Ding, Ze-Fang Li, Guan-Jie Han, Jin-Song Bai, Xi-Lei Li, Jian Dong, Hui-Ren Wang, Li-Bo Jiang

**Affiliations:** 1 Department of Orthopedic Surgery, Zhongshan Hospital, Fudan University, 200032 Shanghai, China; 2 Department of Orthopedic Surgery, Minhang Hospital, Fudan University, 201100 Shanghai, China; 3 Department of Orthopedic Surgery, Qianjiang Central Hospital of Chongqing, 409000 Chongqing, China

**Keywords:** Osteoarthritis, Cytoskeleton, Ageing, Osteoarthritis, Cytoskeleton, Ageing

Correction to: *Experimental & Molecular Medicine* 10.1038/s12276-021-00716-6, published online 21 December 2021

After online publication of this article, the authors noticed an error in Figure 1i.

The correct image of this plot should have shown as below.
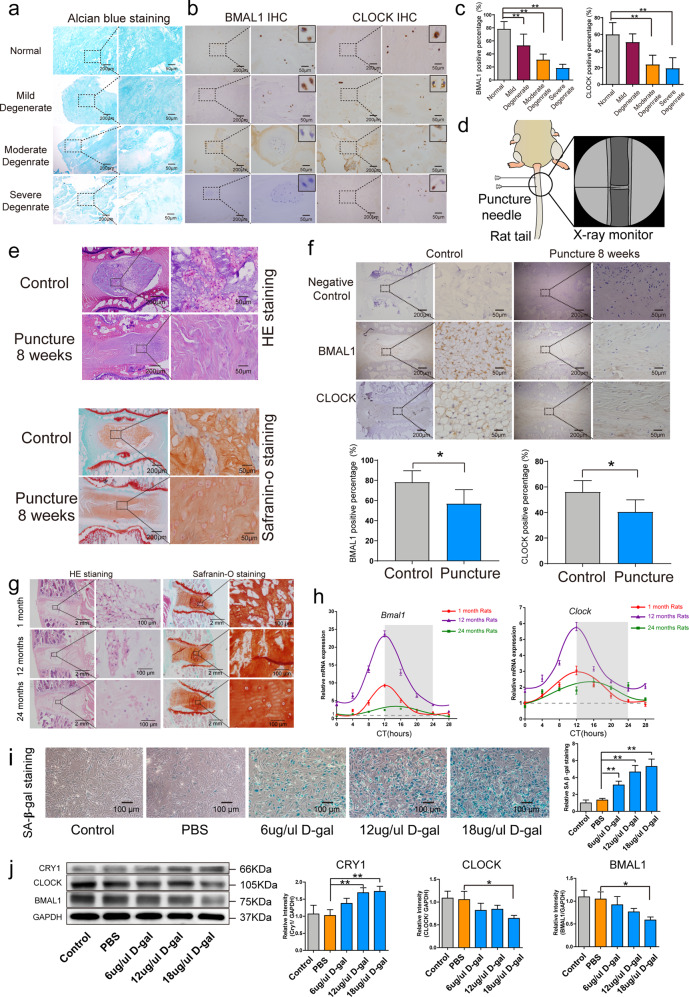


The corrected data were printed below. These corrected results do not alter the conclusions of this article. The authors apologize for any inconvenience caused.

The original article has been corrected.

