# Supplementary material for: Excessive mechanical strain accelerates intervertebral disc degeneration by disrupting intrinsic circadian rhythm
Source: Exp Mol Med. 2021 Dec 21;53(12):1911–23. doi: 10.1038/s12276-021-00716-6 (PMC8741925; doi:10.1038/s12276-021-00716-6)
Supplement: Supplementary file 1 — Revised supplementary information [file 12276_2021_716_MOESM1_ESM.pdf]

SupplementaryFiles

Excessive mechanical strain accelerates intervertebral disc degeneration by disrupting intrinsic circadian rhythm

Sheng-Long Ding[1*], Tai-Wei Zhang[1*], Qi-Chen Zhang[1*], Wang Ding[2], Ze-Fang Li[1,3], Guan-Jie Han[1], Jin-Song Bai[1], Xi-Lei Li[1#], Jian Dong[1#], Hui-Ren Wang[1#], Li-Bo Jiang[1#]

Conflict of Interests statement

The authors have declared that no conflict of interest exists.

Sheng-Long Ding, Tai-Wei Zhang and Qi-Chen Zhang contributed equally to the work.

*Correspondence should be addressed to Li-Bo Jiang, Hui-Ren Wang, Jian Dong and Xi-Lei Li. Department of Orthopedic Surgery, Zhongshan Hospital, Fudan University, Shanghai 200032, China, E-mail: jiang.libo@zs-hospital.sh.cn; wang.huiren@zs-hospital.sh.cn; dong.jian@zs-hospital.sh.cn; li.xilei@zs-hospital.sh.cn

Supplementary Data 1

Reagents

Latrunculin B, cytochalasin D, dexamethasone (Dex) and Y-27632 were purchased from Sigma. The following antibodies were used in this study: anti-BMAL1 (from Novus), anti-CLOCK and anti-CRY1 which were from Abcam; anti-Collagen II (from Bioss), anti-MMP13, anti-Aggrecan (from Novus), anti-phospho-myosin light chain 2 (Ser19) (Cell Signaling), anti-β-actin and anti-GAPDH purchased from Beyotime. The secondary antibodies were obtained from Jackson ImmunoResearch. The antibodies used are listed in online Supplementary Table 3. Among the inhibitors, Y-27632 (Sigma, SCM075) was included.

Supplementary Table 1. Patients' personal information and Pfirrmann classification for histological assessment.

No.	Source	Gender	Age	Diagnosis	Level of the IVD	Degeneration degree
1	Surgery	Male	21	Spine fracture	T12/L1	Normal disc
2	Surgery	Female	33	Spine fracture	L1/L2	Normal disc
3	Surgery	Female	25	Spine fracture	L2/L3	Normal disc
4	Surgery	Male	19	Spine fracture	L1/L2	Normal disc
5	Surgery	Female	68	Spinal canal stenosis	L5/S1	Mild degeneration

6	Surgery	Male	71	IVD herniation	L4/L5	Mild degeneration
7	Surgery	Male	66	Spinal canal stenosis	L5/S1	Mild degeneration
8	Surgery	Male	54	IVD herniation	L4/L5	Mild degeneration
9	Surgery	Male	62	IVD herniation	L5/S1	Mild degeneration
10	Surgery	Male	70	Spinal canal stenosis	L5/S1	Mild degeneration
11	Surgery	Female	43	IVD herniation	L4/L5	Mild degeneration
12	Surgery	Female	45	Lumbar spondylolisthesis	L5/S1	Moderate degeneration
13	Surgery	Male	44	Spinal canal stenosis	L4/L5	Moderate degeneration
14	Surgery	Male	69	IVD herniation	L4/L5	Moderate degeneration
15	Surgery	Male	61	IVD herniation	L5/S1	Moderate degeneration
16	Surgery	Male	67	Spinal canal stenosis	L5/S1	Moderate degeneration
17	Surgery	Female	72	IVD herniation	L3/L4	Moderate degeneration
18	Surgery	Female	55	IVD herniation	L4/L5	Severe degeneration

19	Surgery	Female	75	Spinal canal stenosis	L4/L5	Severe degeneration
20	Surgery	Female	61	Lumbar spondylolisthesis	L4-S1	Severe degeneration
21	Surgery	Male	56	IVD herniation	L5/S1	Severe degeneration

Supplementary Table 2. Patients' personal information and Pfirrmann classification for mRNA sequencing.

No.	Source	Gender	Age	Diagnosis	Level of the IVD	Pfirrmann classification
1	Surgery	Male	30	Lumbar spondylolisthesis	L5/S1	I
2	Surgery	Female	34	Spine fracture	L1/L2	I
3	Surgery	Female	25	Spine fracture	L2/L3	I
4	Surgery	Female	43	Spinal canal stenosis	L5/S1	V
5	Surgery	Female	69	Spinal canal stenosis	L5/S1	IV
6	Surgery	Male	72	Spinal canal stenosis	L4/L5	IV
7	Surgery	Male	69	Spinal canal stenosis	L4/L5	IV
8	Surgery	Male	38	IVD herniation	L5/S1	III
9	Surgery	Female	68	Spinal canal stenosis	L4/L5	IV

Supplementary Table 3. Antibodies and dilution levels for each study

**Antibody**	**Source**	**IF/IHC**	**WB**

**Primary antibody**			
Bmal1 (rabbit monoclonal)	Novus Biologicals #NB100-2288	1: 200	1: 1000
CRY1 (rabbit monoclonal)	Abcam #ab3518		1: 2000
PER2 (rabbit monoclonal)	Novus Biologicals #NB100-125		1: 1000
CLOCK (rabbit monoclonal)	Abcam #ab3517	1: 200	1: 2000
Collagen II (rabbit monoclonal)	Biossusa #bs-0709R		1: 1000
MMP13 (rabbit monoclonal)	Novus Biologicals #NBP2-45887		1: 1000
β-actin (mouse monoclonal)	Abcam #ab8226		1: 1000
GAPDH (rabbit monoclonal)	Abcam #ab181602		1: 2000
pMLC (rabbit monoclonal)	Cell Signaling Technology #3671		1: 1000
Aggrecan (rabbit monoclonal)	Novus Biologicals #NB100-74350		1: 1000
**Secondary antibody**			
Goat anti-Rabbit IgG	Thermo Fisher Scientific	1: 200	

Secondary Antibody, Alexa Fluor Plus 488	#A32731		
**Cy3-labeled Goat Anti-Rabbit IgG(H+L)**	BeyotimeA0516	1: 200	
Goat anti-Rabbit IgG Secondary Antibody, Alexa Fluor Plus 594	Thermo Fisher Scientific # A32740	1: 200	
horseradish peroxidase - conjugated Peroxidase AffiniPure Goat Anti-Mouse IgG (H+L)	Jackson ImmunoResearch #115-035-003		1:5000
horseradish peroxidase - conjugated Peroxidase AffiniPure Goat Anti-Rabbit IgG (H+L)	Jackson ImmunoResearch #111-035-003	1:5000	1:5000

Supplementary Table 4. Histological scoring of the degenerated human intervertebral discs

Grades	Description
	Loss of demarcation between NP and AF
0	Clear demarcation
1	Limited loss of demarcation

2	Substantial loss of demarcation
3	Complete loss of demarcation
	Loss of proteoglycan from NP
0	No loss of haematoxophilia
1	Limited loss of haematoxophilia
2	Substantial loss of haematoxophilia
3	Complete loss of haematoxophilia
	Presence and extent of fissures
0	No fissures
1	Fissures present within NP
2	Fissures extending to junction of NP and AF
3	Fissures extending to within AF
	Cell cluster formation
0	No cell clusters
1	Less than 25% of cells formed into clusters
2	25–75% of cells formed into clusters
3	Over 75% of cells formed into clusters

1) grades 0 to 3 represent a histologically normal disc; 2) grades 4 to 6 indicate mild degeneration; 3) grades 7 to 9 indicate moderate degeneration; and 4) grades 10 to 12 indicate severe degeneration.

Supplementary Table 5. Primer sequences for *BMAL1*, *CLOCK*, *PER2*, *CRY1*,

Col2a1, *Aggrecan, MMP1*, *MMP9*, *MMP13*, *RhoA*, *ADAMTS4*, and *GAPDH*.

Gene	Sequence (5′–3′)	Product size (bp)	Accession no.
Bmal1	CCGTGGACCAAGGAAGTAGA<br>CTGTGAGCTGTGGGAAGGTT	20<br>20	NM_024362.2
Clock	ACAGCGCACACACAGGCCTTC<br>TGGCGGCGCCCTGTGATCTA	21<br>20	NM_021856.2
Per2	GACGGGTCGAGCAAAGGA<br>GGGAAAAGTCCACATATCCATT<br>CA	18<br>24	NM_031678.1
Cry1	AGCTCAAGTTGGTGGGAGAGT<br>CAG<br>TGATTCCACGGAGCTTCTAAAG<br>C	24<br>23	NM_198750.2
Col2a1	ACGCTCAAGTCGCTGAACAAC<br>C<br>ATCCAGTAGTCTCCGCTCTTCC<br>AC	22<br>24	NM_012929.1
Aggrecan	CTGATCCACTGTCCAAGCACCA<br>TG<br>ATCCACGCCAGGCTCCACTC	24<br>20	J03485.1
Mmp1	TGTTCGCCTTCTACAGAGGAGA	24	NM_001134530.1

	CC		
	TGTCGGTCCACGTCTCATCCAG	22	
	CTACACGGAGCATGGCAACGG	21	NM_031055.1
Mmp9	TGGTGCAGGCAGAGTAGGAGT	22	
	G		
	AACCAAGATGTGGAGTGCCTG	24	NM_133530.1
	ATG	24	
Mmp13	CACATCAGACCAGACCTTGAA		
	GGC		
	GCTTGTGGTAAGACATGCTTGC	24	NM_057132.3
	TC	24	
Rhoa	GGCCTCAGACGGTCATAATCTT		
	CC		
	CCGTTCCGCTCCTGTAACACTA	24	NM_023959.1
Adamts4	AG	22	
	AGGTCGGTTCGGTGGTTGTAGG		
	AACCTGCCAAGTATGATGACAT	24	NM_017008.4
	CA	24	
Gapdh	AACCTGCCAAGTATGATGACAT		
	CA		

Supplementary Table 6. Sequence of the *RhoA* siRNA.

Item	Sequence	Product size (bp)
siRhoa1	5’-TGGACGGGAAGCAGGTAGA-3’	19
siRhoa2	5’- GGTGATTGTTGGTGATGGA-3’	19
siRhoa3	5’- GAAGTCAAGCATTTCTGTC-3’	19

Supplementary Figures

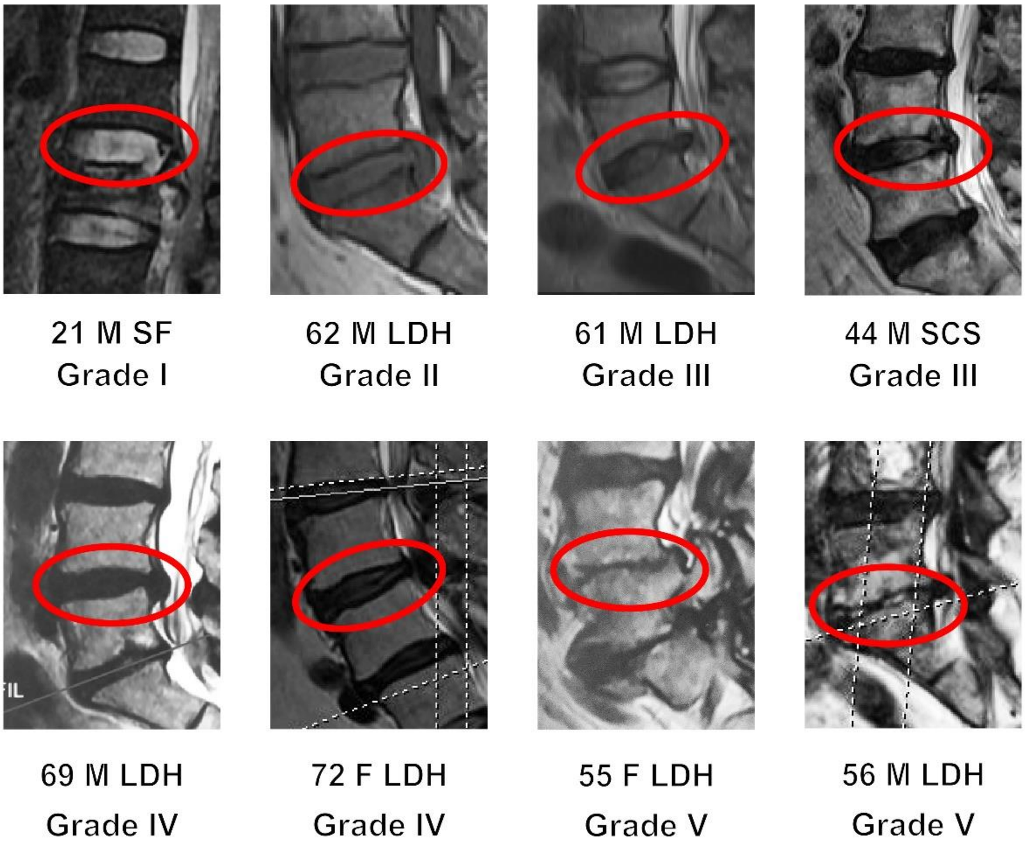

Supplementary Fig. 1. Example of a degenerated IVD grade based on the Pfirrmann guide. The number below each image represents the age of the patient. M, male; F, female; SF, spine fracture; LDH, lumbar disc hernia; and SCS, spinal canal stenosis.

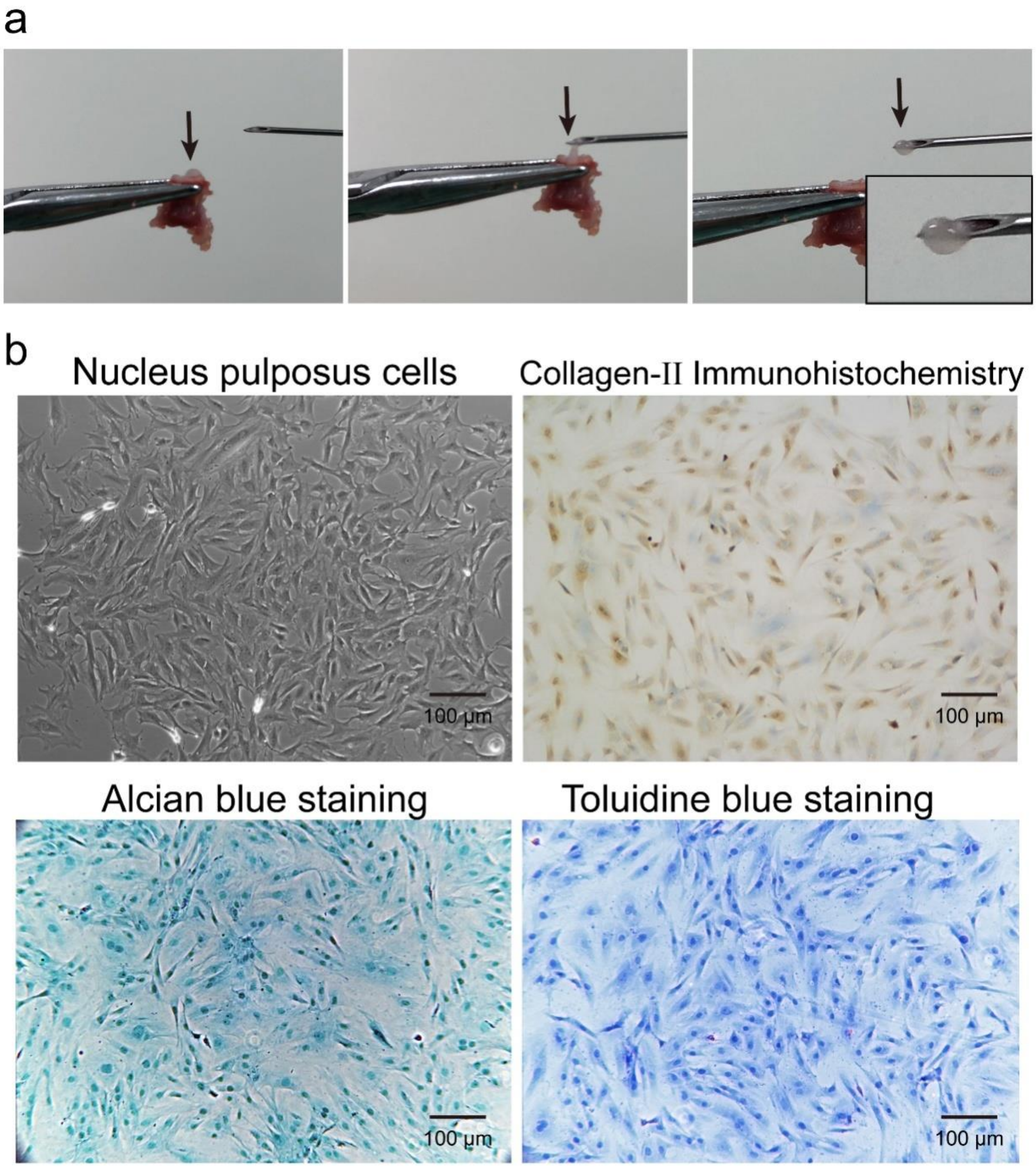

Supplementary Fig. 2. **Isolation and identification of the NP cells. a** Spinal columns from L1 to L5 were separated under aseptic conditions. The annulus fibrosus tissues was removed, and the gelatinous NP tissue was collected. **b** NP cells were visualized under an optical microscope with or without collagen-II immunohistological, Alcian blue or toluidine blue staining. n=3.

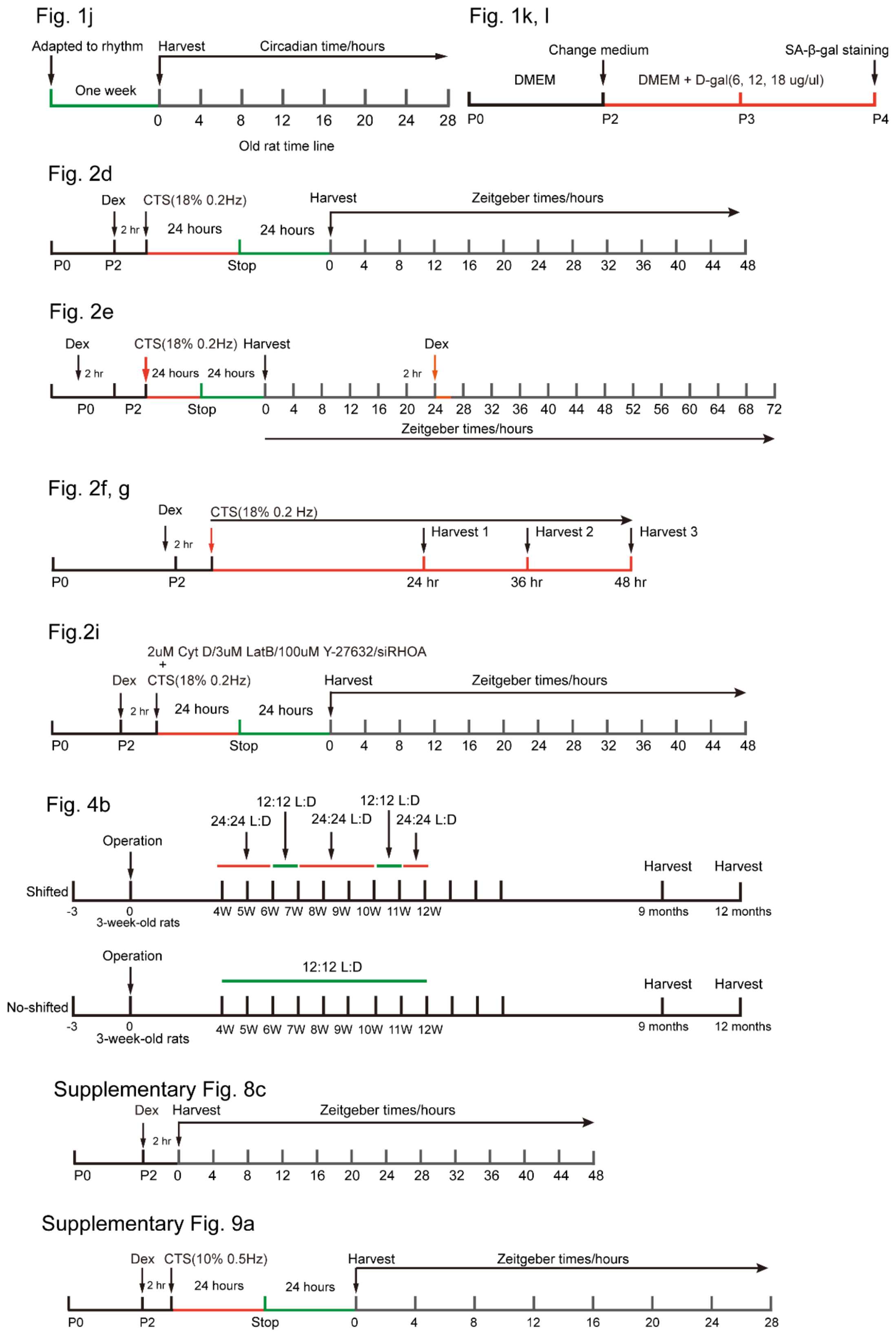

Supplementary Fig. 3. Timeline of the treatments applied in this study.

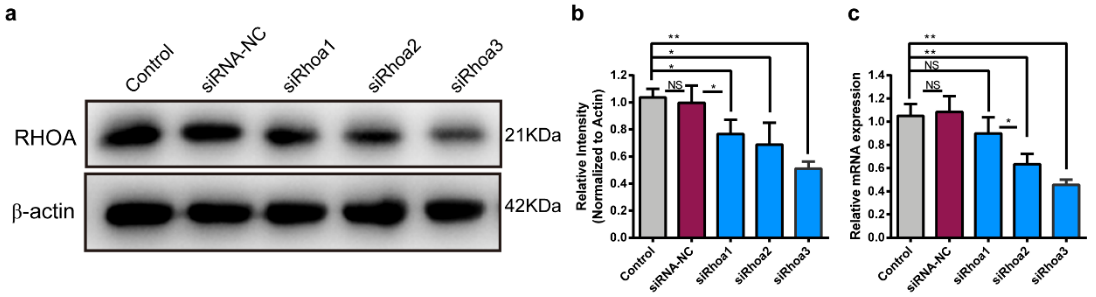

Supplementary Fig. 4. Effect of *RhoA* siRNA in the NP cells. **a, b** Protein expression of RhoA in the NP cells. n=3. **c** mRNA expression of *RhoA* in the NP cells. Transcripts were detected by real-time qPCR over a period of 48 h; n = 3.

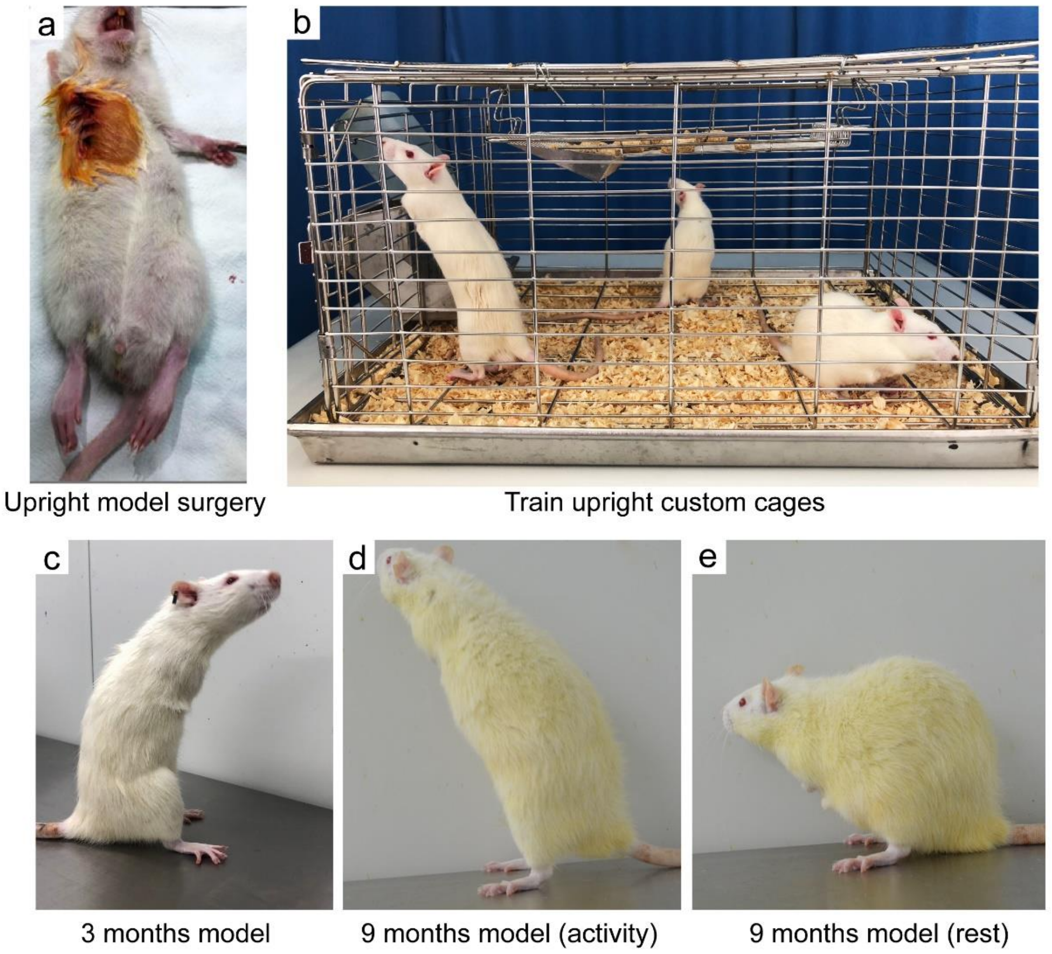

Supplementary Fig. 5. Rat model with forelimb amputation. a Forelimbs of the rats showing the amputation at the middle part of the humerus. **b** The cage is set at the highest height. The rats can stretch their bodies and stand upright. Food and water were

hung from the top of the cage. Rats were allowed to stand upright to eat and drink. **c** Rats after 3 months of modeling had become accustomed to standing upright. **d** Rats 9 months after the model was established showing activity. **e** Rats at rest 9 months after the model was established.

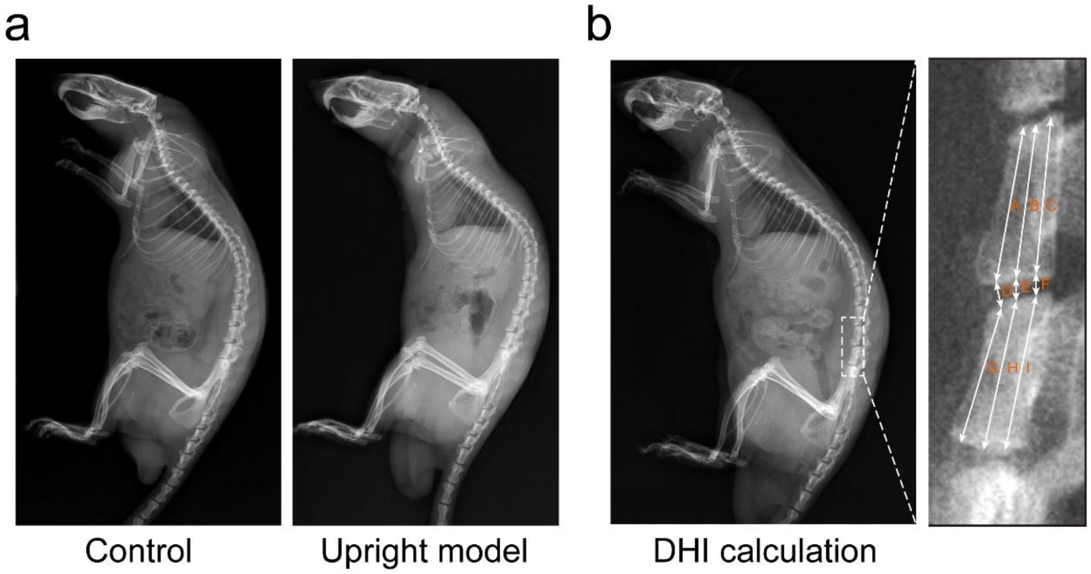

Supplementary Fig. 6. X-ray image of the lumbar spine of the rats. a Rats in the control group and rats in the upright model group. **b** DHI measurement, DHI = 2 (D + E + F)/(A + B + C + G + H + I).

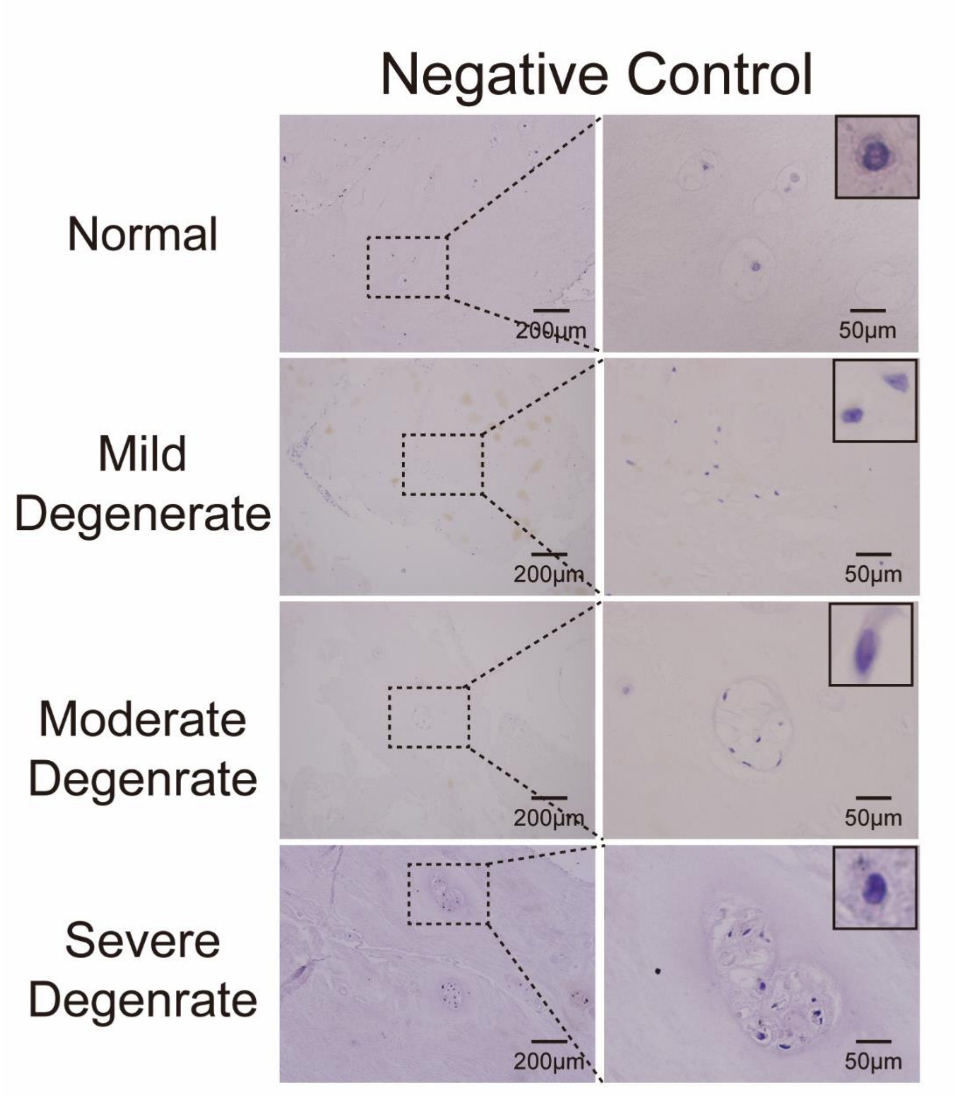

Supplementary Fig. 7. Negative control for the IHC assays of the normal and degenerated IVDs. Negative control for the IHC assays of BMAL1 and CLOCK proteins for sections of the normal and degenerated IVDs; n=3. Magnification of 5× for the left panels and 10× for the right panels.

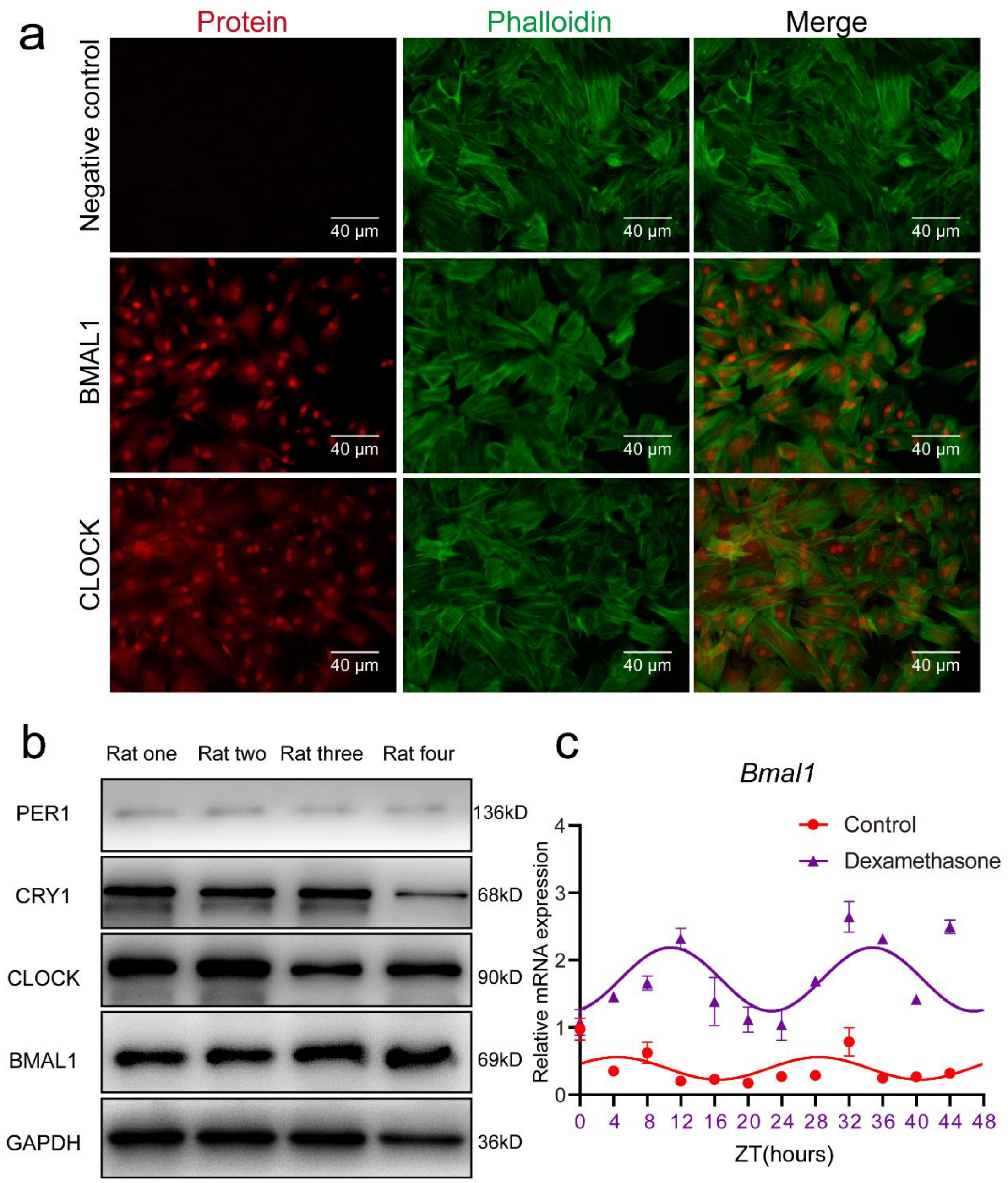

Supplementary Fig. 8. Expression of the circadian clock-related genes in the NP cells. a The immunofluorescent BMAL1 and CLOCK proteins were counterstained with phalloidin. **b** Protein expression of PER1, CRY1, CLOCK and BMAL1 in the rat NP tissues. n=3. **c** Rhythmic expression of *BMAL1* mRNA in the NP cells treated for 1 h with DEX. Transcripts were detected by real-time qPCR over a period of 48 h; n = 3 Data were superimposed with corresponding sine waves fitted through CircWave

analyses.

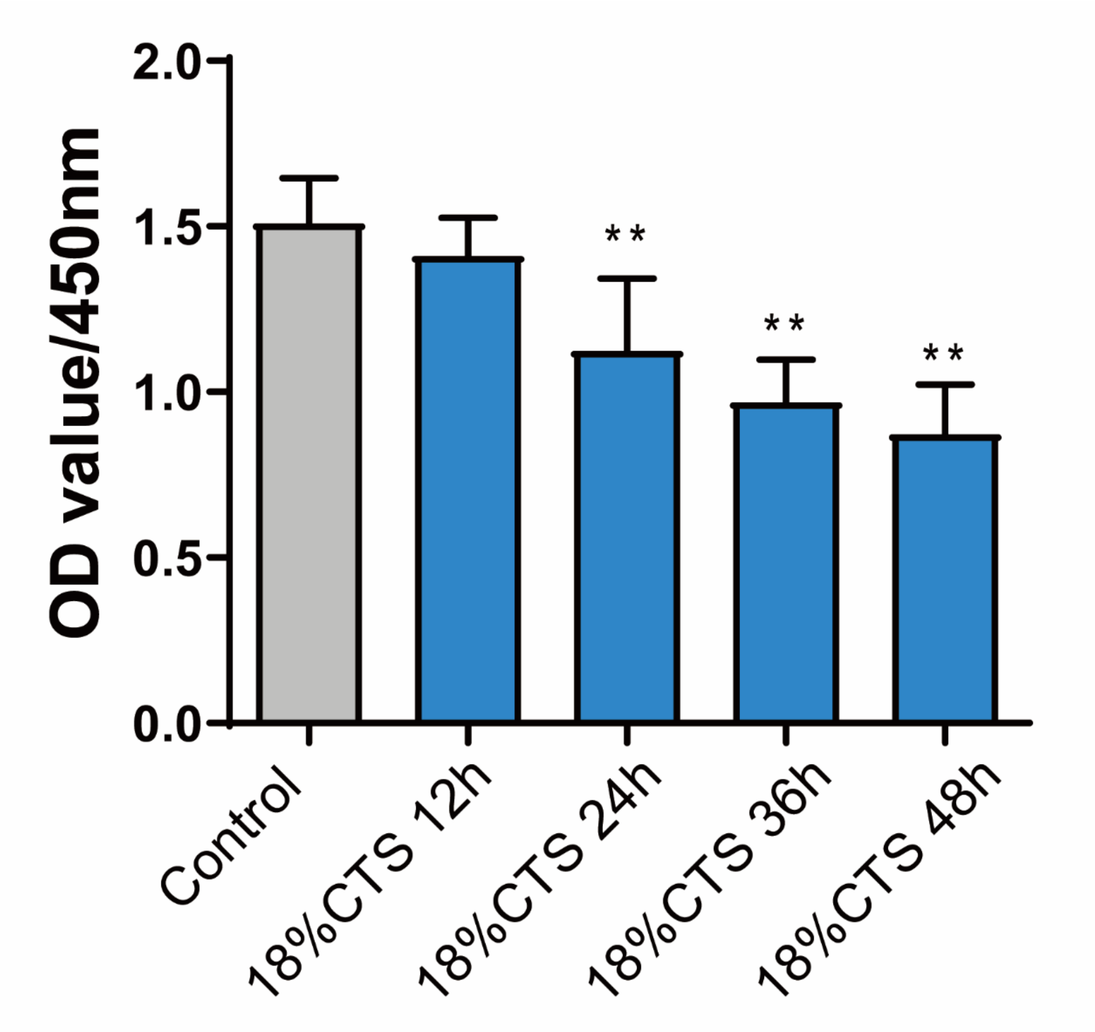

Supplementary Fig. 9. Cell viability under 18% CTS at 0.2 Hz.

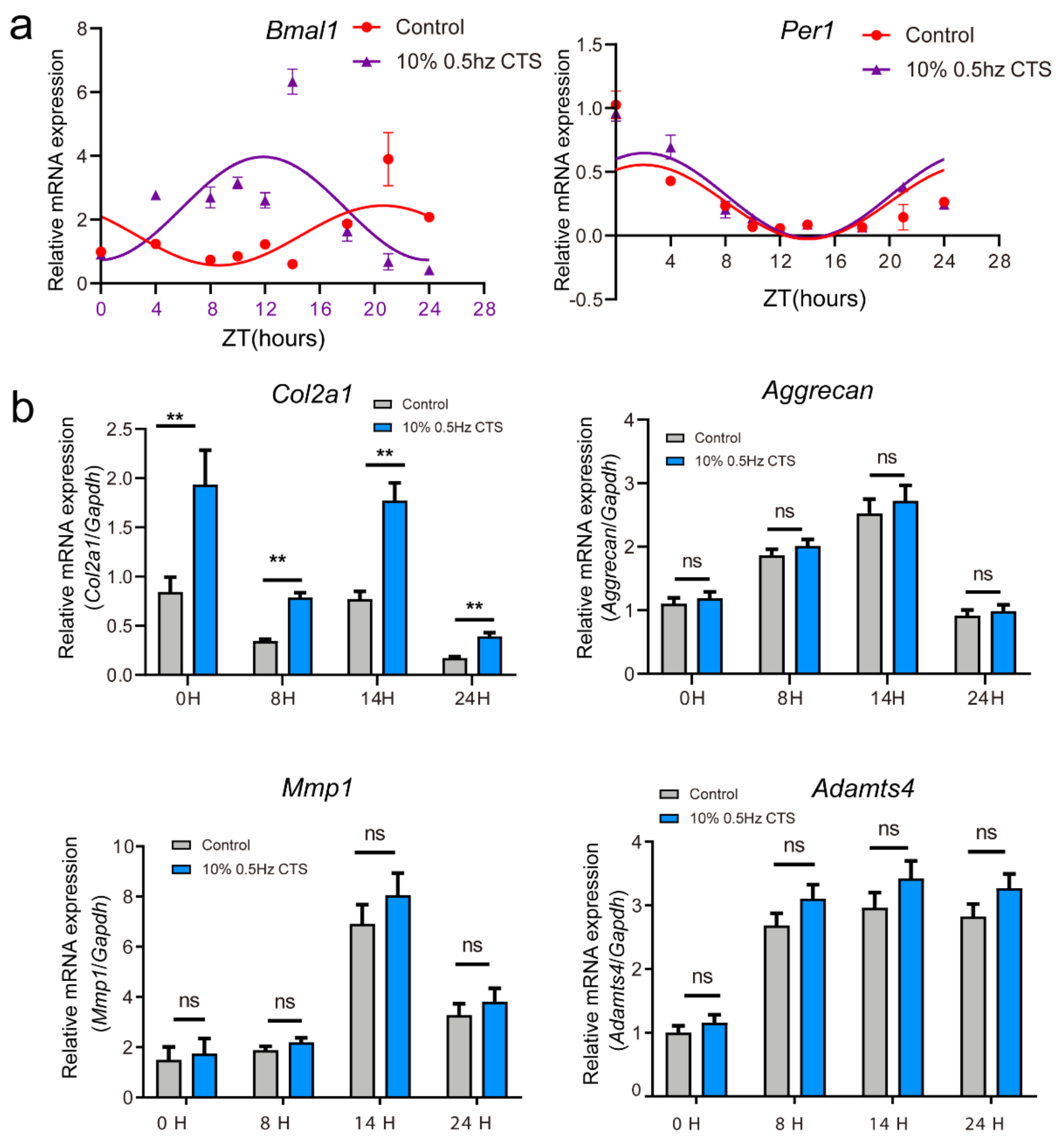

Supplementary Fig. 10. Rhythmic expression of genes in the NP cells treated for 24 h with 10% CTS at 0.5 Hz and with a nonspecific treatment. a Rhythmic expression of *BMAL1* and *PER2* mRNA in the NP cells. Transcripts were detected ed by real-time qPCR over a period of 48 h; n = 3. **b** Expression of *Col2a1*, *Aggrecan*, *MMP1* and *ADAMTS4* was determined 0, 8, 14, and 24 h after CTS treatment. The increase in the expression of *Col2a1* was notable after the treatment.

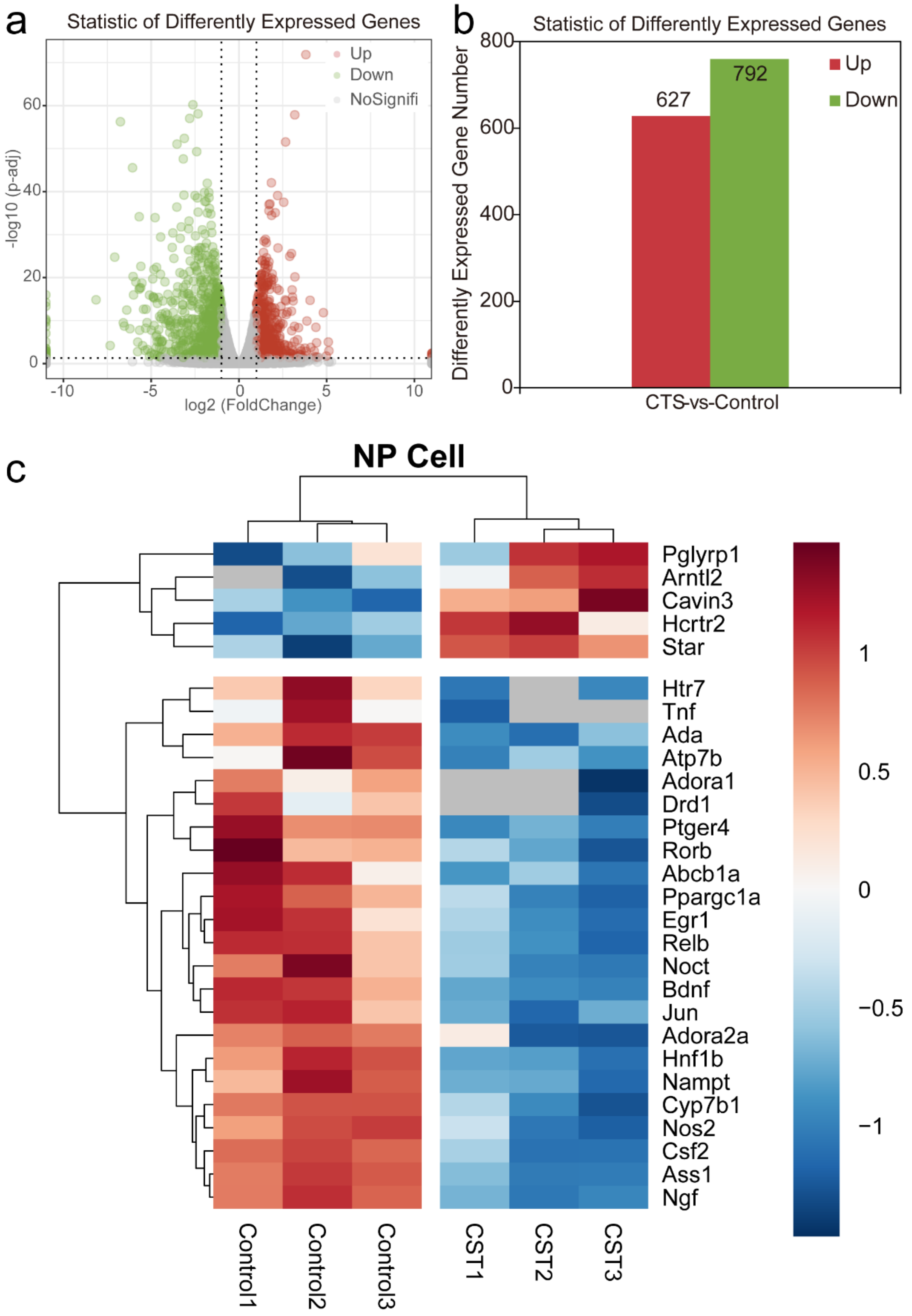

Supplementary Fig. 11. Variation of genes in the CTS-treated and control NP cells shown in a scatter diagram based on the mean FPKM. a Red points indicate upregulated mRNA; green points indicate downregulated mRNA; gray points indicate

mRNA without significant variation. The default fold change threshold is 2.0. **b** Statistics of the differentially expressed genes. **c** Heat map of the genes of Gene Ontology (GO) analysis indicating the involvement of CR under CTS. n=3

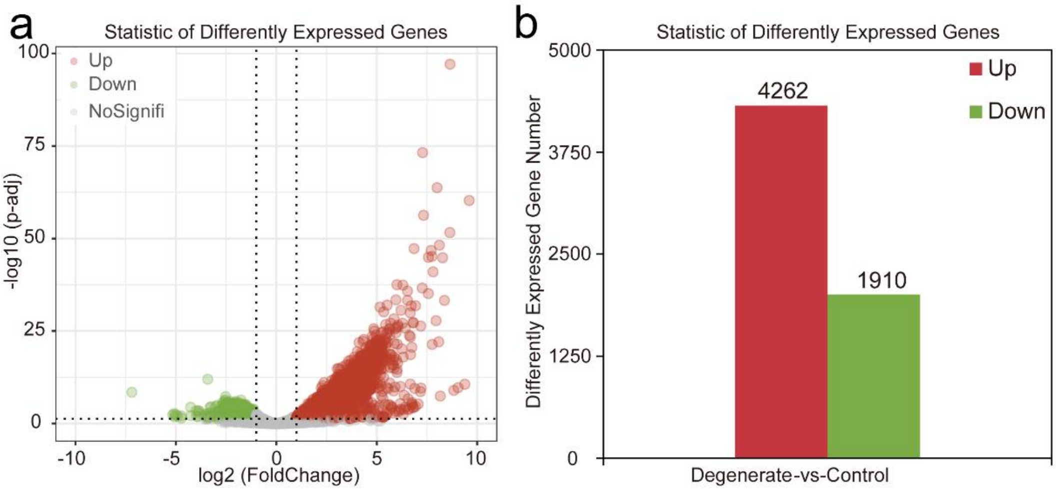

Supplementary Fig. 12. Variation of the genes between the degenerated and control IVD groups in a scatter diagram according to the mean FPKM. a Red points indicate upregulated mRNA; green points indicate downregulated mRNA; gray points indicate mRNA without significant variation. The default fold change threshold was 2.0. **b** Statistics of the differentially expressed genes.

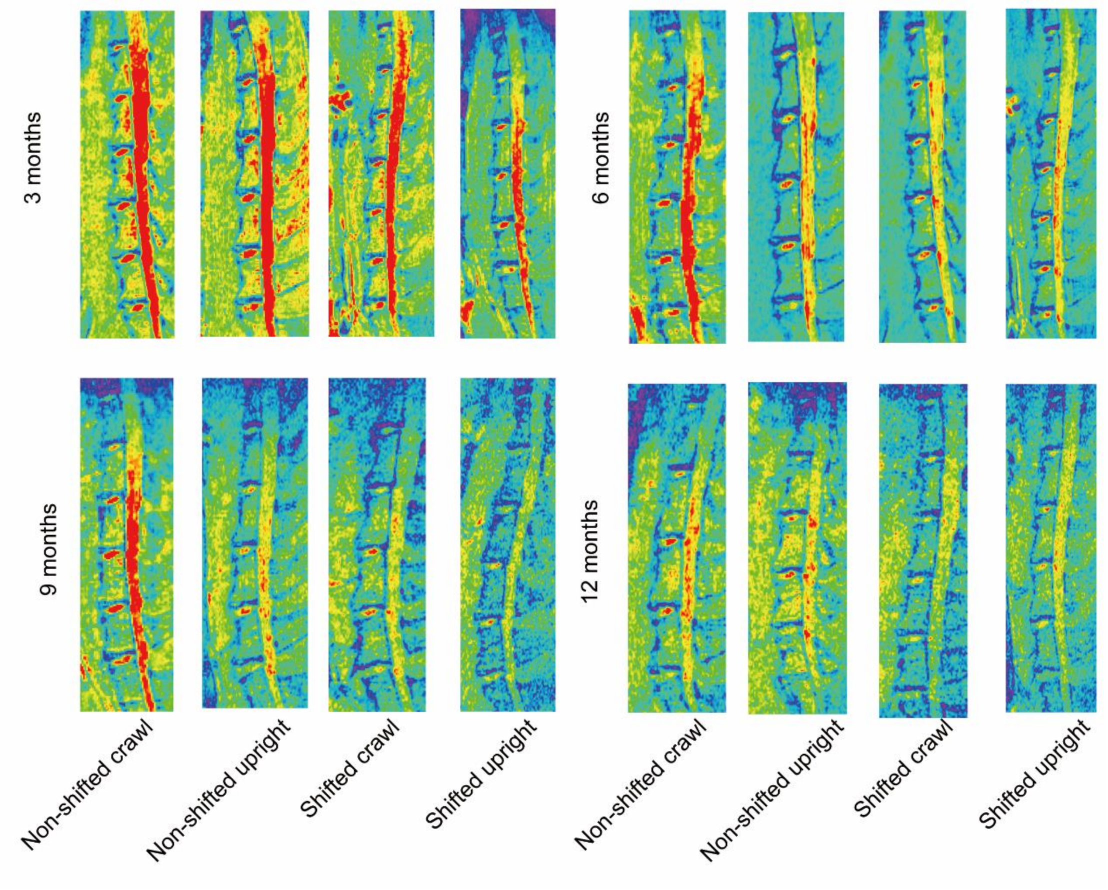

Supplementary Fig. 13. Pseudo-colored spine MRI scans in rats 3, 6, 9 and 12 months after the operation, n=3.

Supplementary Table 7 Clusterprofiler enriched GO terms in compare between CST vs Con

ID	Description	GeneRatio	BgRatio	pvalue	p.adjust	qvalue	geneID	Count
GO:0050900	leukocyte migration	78/1242	370/18303	1.04E-19	3.41E-16	2.33E-16	Ada/Adora1/Bdkrb1/Cadm1/Ccl2/Ccl20/Ccl22/Ccl3/Ccl5/Ccl9/Ccr1/Cd200r1/Cd99l2/Ch25h/Cmklr1/Coro1a/Csf1/Cx3cl1/Cxcl1/Cxcl10/Cxcl11/Cxcl12/Cxcl16/Cxcl2/Cxcl3/Cyp7b1/Dapk2/Dock8/Ednrb/Emilin1/F11r/Ripor2/Vegfd/Flt1/Gcnt1/Il1a/Il1b/Il1r1/Il23a/Il33/Itga2/Itgal/Itgb3/Jaml/Kitlg/LOC290595/Lbp/Lgals3/Lgals9/Mcoln2/Mmp9/Nfkb1/Nfkbia/Nlrp3/Nod2/Pde4b/Pdgfb/Pgf/Pla2g7/Plcb1/Ptger4/Ptpro/Pycard/Rarres2/Selp/Sirpa/Slit2/Syk/Thbs1/Thbs4/Tlr2/Tnf/Tnfsf18/Trem3/Trpv4/Vc	78
GO:0030198	extracellular matrix organization	69/1242	302/18303	1.47E-19	3.41E-16	2.33E-16	Adamts15/Adamts4/Adamts6/Adamts8/Adamtsl3/Bcl3/Ccdc80/Cflar/Col11a1/Col13a1/Col14a1/Col17a1/Col1a1/Col25a1/Col2a1/Col3a1/Col4a1/Col5a1/Col5a3/Col8a2/Col9a3/Creb3l1/Crispld2/Cyp1b1/Elf3/Emilin1/Ero1b/Ets1/Fbln1/Fbln5/Flot1/Foxc1/Fscn1/Has2/Hpse2/Ibsp/Itgb3/Kazald1/Klk4/Angptl7/Lamb3/Lgals3/Meltf/Mkx/Mmp10/Mmp12/Mmp13/Mmp16/Mmp1b/Mmp3/Mmp9/Ndnf/Nfkb2/Nox1/Npnt/Ntn4/Olfml2b/Pmp22/Ramp2/Rgcc/Scara3/Scx/Serpinf2/Slc2a10/Smoc1/Tnf/Tnfrsf	69
GO:0043062	extracellular structure organization	69/1242	303/18303	1.78E-19	3.41E-16	2.33E-16	Adamts15/Adamts4/Adamts6/Adamts8/Adamtsl3/Bcl3/Ccdc80/Cflar/Col11a1/Col13a1/Col14a1/Col17a1/Col1a1/Col25a1/Col2a1/Col3a1/Col4a1/Col5a1/Col5a3/Col8a2/Col9a3/Creb3l1/Crispld2/Cyp1b1/Elf3/Emilin1/Ero1b/Ets1/Fbln1/Fbln5/Flot1/Foxc1/Fscn1/Has2/Hpse2/Ibsp/Itgb3/Kazald1/Klk4/Angptl7/Lamb3/Lgals3/Meltf/Mkx/Mmp10/Mmp12/Mmp13/Mmp16/Mmp1b/Mmp3/Mmp9/Ndnf/Nfkb2/Nox1/Npnt/Ntn4/Olfml2b/Pmp22/Ramp2/Rgcc/Scara3/Scx/Serpinf2/Slc2a10/Smoc1/Tnf/Tnfrsf	69
GO:1901342	regulation of vasculature development	73/1242	356/18303	8.14E-18	1.17E-14	8.03E-15	Agtr1a/Angpt4/Anxa3/Aqp1/Bmp4/Bmp7/Bmper/Camp/Ccl2/Ccl5/Cd40/Cflar/Creb3l1/Cth/Cx3cl1/Cxcl10/Cxcr4/Cyp1b1/Dcn/Dll1/Efna1/Egr1/Emilin1/Enpp2/Ereg/Ets1/F3/Fbln5/Fgf9/Vegfd/Flt1/Foxc1/Gata2/Gata6/Gpr4/Hdac9/Hey2/Hipk2/Hk2/Hmga2/Hyal1/Il1a/Il1b/Itga5/Itgax/Itgb3/Klf2/Angptl7/Lgals3/Lif/Meox2/Mmp9/Pdgfb/Pgf/Plk2/Plxnd1/Ppp1r15a/Ptger4/Ptgs2/Ramp2/Rgcc/Rhoj/Sema4a/Sema5a/Sema6a/Stat1/Thbs1/Thbs2/Thbs4/Tlr3/Tmem100/Tnf/Wnt5a	73
GO:0042060	wound healing	76/1242	420/18303	2.45E-15	2.82E-12	1.93E-12	Adra2a/Alox15/Anxa8/Aqp1/Arhgef19/Bmp4/C1qtnf1/Cadm4/Cask/Ccl2/Ccl20/Cd44/Cdh3/Cfh/Cflar/Cldn3/Col1a1/Col3a1/Col5a1/Cx3cl1/Cxcl2/Cxcr4/Dcn/Dusp10/Egr1/Ephb2/Eppk1/Ets1/F11r/F13a1/F3/Fbln1/Fgfr2/Gata2/Gna13/Gpr4/Hmgcr/Hpse/Il1a/Il1b/Itga2/Itga5/Itgb3/Jaml/Klf6/Mmp12/Mmp3/Mmrn1/Mylk/Ndnf/Nos2/Nrg1/P2ry2/Pdgfb/Pik3cb/Plat/Plau/Plek/Plet1/Plpp3/Ppard/Ppl/Procr/Ptger4/Selp/Serpinb2/Serpinf2/Serping1/Slc7a11/Syk/Tfpi2/Thbd/Thbs1/Tnf/Wfdc1/Wnt5a	76
GO:0097529	myeloid leukocyte migration	50/1242	218/18303	1.34E-14	1.29E-11	8.83E-12	Ccl2/Ccl20/Ccl22/Ccl3/Ccl5/Ccl9/Ccr1/Cd200r1/Cd99l2/Cmklr1/Csf1/Cx3cl1/Cxcl1/Cxcl10/Cxcl11/Cxcl12/Cxcl2/Cxcl3/Dapk2/Ednrb/Emilin1/Ripor2/Vegfd/Flt1/Il1a/Il1b/Il1r1/Il23a/Jaml/Lbp/Lgals3/Mcoln2/Nod2/Pde4b/Pdgfb/Pgf/Pla2g7/Plcb1/Ptger4/Ptpro/Rarres2/Sirpa/Slit2/Syk/Thbs1/Thbs4/Tnfsf18/Trem3/Trpv4/Xcl1	50
GO:0070555	response to interleukin-1	43/1242	170/18303	2.51E-14	2.07E-11	1.42E-11	Akap12/Ankrd1/Camp/Ccl2/Ccl20/Ccl22/Ccl3/Ccl5/Ccl9/Cd40/Cx3cl1/Cxcl2/Egr1/Ets1/Vegfd/Gclc/Has2/Hyal1/Il1a/Il1b/Il1r1/Il1rn/Irak2/Irf1/Acod1/Klf2/Lgals9/Mmp1/Mmp3/Mmp9/Nfkb1/Nos2/P2ry2/Pcsk1/Plcb1/Ptger4/Pycard/Ripk2/Serpina3n/Sfrp1/Sirpa/Xcl1/Xylt1	43
GO:0045765	regulation of angiogenesis	62/1242	320/18303	4.00E-14	2.88E-11	1.97E-11	Agtr1a/Angpt4/Anxa3/Aqp1/Bmper/Camp/Ccl2/Ccl5/Cd40/Creb3l1/Cx3cl1/Cxcl10/Cxcr4/Cyp1b1/Dcn/Dll1/Efna1/Emilin1/Enpp2/Ereg/Ets1/F3/Fbln5/Vegfd/Flt1/Foxc1/Gata2/Gata6/Gpr4/Hdac9/Hipk2/Hk2/Hmga2/Hyal1/Il1a/Il1b/Itga5/Itgax/Itgb3/Klf2/Lgals3/Lif/Meox2/Mmp9/Pgf/Plk2/Plxnd1/Ppp1r15a/Ptgs2/Ramp2/Rgcc/Rhoj/Sema4a/Sema5a/Sema6a/Stat1/Thbs1/Thbs2/Thbs4/Tlr3/	62
GO:0009612	response to mechanical stimulus	63/1242	331/18303	5.84E-14	3.74E-11	2.56E-11	Adgrv1/Adora1/Ankrd1/Aqp1/Bdkrb1/Bdnf/Bmp2/Bmp4/Btg2/Ccl2/Cd40/Col11a1/Col1a1/Col2a1/Col3a1/Cxcl10/Cxcl12/Cxcr4/Cyp11b2/Dcn/Egr1/Ets1/Etv1/F11r/F3/Ripor2/Fosl1/Gclc/Htr2a/Htr7/Il13ra2/Il1b/Irf1/Itga2/Itgb3/Jun/Junb/Kcnk2/Map3k14/Mgp/Mmp1/Mmp13/Mmp3/Mmp9/Nfkb1/Nfkbia/Ngf/Nos2/Pde2a/Pdgfb/Piezo2/Ppl/Ptger4/Ptgs2/Ryr2/Scx/Stat1/Thbs1/Timp3/Tlr3/T	63
GO:0070372	regulation of ERK1 and ERK2 cascade	61/1242	319/18303	1.19E-13	6.84E-11	4.68E-11	Akap12/Alox15/Atf3/Bmp2/Bmp4/Bmper/C1qtnf3/Ccl2/Ccl20/Ccl22/Ccl3/Ccl5/Ccl9/Ccr1/Cd44/Cflar/Cx3cl1/Dusp10/Dusp6/Emilin1/Ephb2/Errfi1/Fbln1/Fgfr2/Flcn/Flt4/Gstp1/Hand2/Hmgcr/Htr2a/Il1a/Il1b/Itgb3/Jun/Lgals9/Lif/Ndrg2/Nek10/Ngf/Nod2/Npnt/P2ry6/Pdgfb/Pla2g2a/Pla2g5/Cavin3/Pycard/Rap1b/Rasgrp1/Rgs14/Ripk2/Sema6a/Serpinf2/Sirpa/Spry4/Syk/Timp3/Tlr2/Tnip1/Tr	61

ID	Description	GeneRatio	BgRatio	pvalue	p.adjust	qvalue	geneID	Count
GO:0070371	ERK1 and ERK2 cascade	63/1242	340/18303	2.08E-13	1.00E-10	6.87E-11	Akap12/Alox15/Atf3/Bmp2/Bmp4/Bmper/C1qtnf3/Ccl2/Ccl20/Ccl22/Ccl3/Ccl5/Ccl9/Ccr1/Cd44/Cflar/Csf2/Cx3cl1/Dusp10/Dusp6/Emilin1/Ephb2/Errfi1/Fbln1/Fgfr2/Flcn/Flt4/Gstp1/Hand2/Hmgcr/Htr2a/Il1a/Il1b/Itgb3/Jun/Lgals9/Lif/Ndrg2/Nek10/Ngf/Nod2/Npnt/P2ry6/Pdgfb/Pla2g2a/Pla2g5/Cavin3/Ptger4/Pycard/Rap1b/Rasgrp1/Rgs14/Ripk2/Sema6a/Serpinf2/Sirpa/Spry4/Syk/Timp3/Tlr2/Tnip1/Trpv4/Xcl1	63
GO:0071219	cellular response to molecule of bacterial origin	60/1242	315/18303	2.26E-13	1.00E-10	6.87E-11	Ankrd1/Ass1/Camp/Ccl2/Ccl20/Ccl3/Ccl5/Cd180/Cd274/Cd40/Cd68/Cfh/Csf2/Csf3/Cx3cl1/Cxcl1/Cxcl10/Cxcl11/Cxcl16/Cxcl2/Cxcl3/Ednrb/Fzd5/Gstp1/Il1a/Il1b/Irak2/Acod1/Jun/Lbp/Mef2c/Mmp1/Mmp9/Ncam1/Nfkb1/Nfkbia/Nfkbib/Nlrp3/Nod2/Nos2/P2ry2/Pde2a/Pde4b/Plau/Ppard/Ppargc1a/Prdm1/Pycard/Ripk2/Sirpa/Star/Stat1/Tlr2/Tlr5/Tlr6/Tnf/Tnfaip3/Tnfrsf1b/Vim/Wnt5a	60
GO:0001819	positive regulation of cytokine production	76/1242	457/18303	2.41E-13	1.00E-10	6.87E-11	Adra2a/Agtr1a/Bcl3/C1qtnf3/Cadm1/Camp/Card11/Ccl2/Ccl20/Ccl3/Cd274/Cd40/Cd83/Clec4e/Csf2/Cx3cl1/Cyp1b1/Egr1/Ereg/F3/Fgr/Vegfd/Flot1/Flt4/Furin/Fzd5/Gbp5/Hilpda/Hpse/Hspa1b/Icoslg/Il15/Il1a/Il1b/Il1r1/Il23a/Il33/Irf1/Lbp/Lgals9/Mcoln2/Mmp12/Nlrc4/Nlrp3/Nod2/Nox1/P2ry2/Pde4b/Peli1/Plcb1/Ptger4/Ptgs2/Pycard/Inava/Myb/Rasgrp1/Rel/Rgcc/Riok3/Ripk2/Rsad2/Serpinf2/Stat1/Syk/Thbs1/Tlr2/Tlr3/Tlr5/Tlr6/Tnf/Tnfsf15/Trim6/Trpv4/Tslp/Wnt5a/Xcl1	76
GO:0002687	positive regulation of leukocyte migration	40/1242	159/18303	2.44E-13	1.00E-10	6.87E-11	Bdkrb1/Ccl2/Ccl20/Ccl3/Ccl5/Ccr1/Cd99l2/Cmklr1/Csf1/Cx3cl1/Cxcl10/Cxcl12/Dapk2/Dock8/Ripor2/Vegfd/Il1a/Il1b/Il1r1/Il23a/Itga2/Itgb3/Kitlg/Lbp/Lgals3/Lgals9/Mmp9/Pgf/Pla2g7/Ptger4/Pycard/Rarres2/Selp/Thbs1/Thbs4/Tlr2/Tnfsf18/Trpv4/Wnt5a/Xcl1	40
GO:0071216	cellular response to biotic stimulus	63/1242	342/18303	2.74E-13	1.05E-10	7.18E-11	Abcb1a/Abcb1b/Ankrd1/Ass1/Camp/Ccl2/Ccl20/Ccl3/Ccl5/Cd180/Cd274/Cd40/Cd68/Cfh/Csf2/Csf3/Cx3cl1/Cxcl1/Cxcl10/Cxcl11/Cxcl16/Cxcl2/Cxcl3/Ednrb/Fzd5/Gstp1/Il1a/Il1b/Irak2/Acod1/Jun/Lbp/Mef2c/Mmp1/Mmp9/Ncam1/Nfkb1/Nfkbia/Nfkbib/Nlrp3/Nod2/Nos2/P2ry2/Pde2a/Pde4b/Plau/Ppard/Ppargc1a/Prdm1/Pycard/Ripk2/Sirpa/Star/Stat1/Syk/Tlr2/Tlr5/Tlr6/Tnf/Tnfaip3/	63
GO:0002685	regulation of leukocyte migration	49/1242	227/18303	2.91E-13	1.05E-10	7.18E-11	Ada/Adora1/Bdkrb1/Ccl2/Ccl20/Ccl3/Ccl5/Ccr1/Cd200r1/Cd99l2/Cmklr1/Csf1/Cx3cl1/Cxcl10/Cxcl12/Dapk2/Dock8/Emilin1/Ripor2/Vegfd/Il1a/Il1b/Il1r1/Il23a/Il33/Itga2/Itgb3/Kitlg/LOC290595/Lbp/Lgals3/Lgals9/Mmp9/Nod2/Pgf/Pla2g7/Plcb1/Ptger4/Pycard/Rarres2/Selp/Slit2/Thbs1/Thbs4/Tlr2/Tnfsf18/Trpv4/Wnt5a/Xcl1	49
GO:0050727	regulation of inflammatory response	66/1242	372/18303	4.54E-13	1.53E-10	1.05E-10	Abcd1/Ada/Adora1/Adora2a/Akna/Alox15/C1qtnf3/Calcrl/Casp12/Ccl3/Ccl5/Cd200r1/Cd44/Chid1/Cx3cl1/Dusp10/Ednrb/Ets1/Fabp4/Gbp5/Ggt1/Gpr4/Ier3/Il1b/Il1r1/Il33/Acod1/Itga2/Lbp/Ldlr/Lgals9/Mgll/Nfkb1/Nfkbia/Nlrp3/Nod2/Nos2/Pde2a/Pglyrp1/Pla2g5/Pmp22/Ppard/Ptger4/Ptgs2/Pycard/Siglec10/Sirpa/Slc7a2/Slit2/Socs3/Tgm2/Tlr2/Tlr3/Tlr6/Tnf/Tnfaip3/Tnfaip6/Tnfrsf1b/Tnfsf18/Tnip1/Trpv4/Tslp/Usp18/Wfdc1/Wnt5a/Xcl1	66
GO:0030595	leukocyte chemotaxis	48/1242	222/18303	4.78E-13	1.53E-10	1.05E-10	Ccl2/Ccl20/Ccl22/Ccl3/Ccl5/Ccl9/Ccr1/Ch25h/Cmklr1/Coro1a/Csf1/Cx3cl1/Cxcl1/Cxcl10/Cxcl11/Cxcl12/Cxcl16/Cxcl2/Cxcl3/Cyp7b1/Dapk2/Ednrb/Ripor2/Vegfd/Flt1/Il1b/Il23a/Jaml/LOC290595/Lbp/Lgals3/Lgals9/Nod2/Pde4b/Pdgfb/Pgf/Pla2g7/Ptpro/Rarres2/Slit2/Syk/Thbs1/Thbs4/Tnfsf18/Trem3/Trpv4/Wnt5a/Xcl1	48
GO:0071347	cellular response to interleukin-1	36/1242	137/18303	9.41E-13	2.86E-10	1.95E-10	Akap12/Ankrd1/Camp/Ccl2/Ccl20/Ccl22/Ccl3/Ccl5/Ccl9/Cd40/Cx3cl1/Cxcl2/Egr1/Has2/Hyal1/Il1a/Il1b/Il1r1/Il1rn/Irak2/Irf1/Acod1/Klf2/Mmp1/Mmp3/Mmp9/Nfkb1/Nos2/P2ry2/Plcb1/Ptger4/Pycard/Serpina3n/Sfrp1/Sirp	36
GO:0097530	granulocyte migration	38/1242	154/18303	1.84E-12	5.31E-10	3.63E-10	Ccl2/Ccl20/Ccl22/Ccl3/Ccl5/Ccl9/Cd99l2/Cmklr1/Csf1/Cx3cl1/Cxcl1/Cxcl10/Cxcl11/Cxcl2/Cxcl3/Dapk2/Ripor2/Il1a/Il1b/Il1r1/Il23a/Jaml/Lbp/Lgals3/Mcoln2/Nod2/Pde4b/Ptger4/Rarres2/Sirpa/Slit2/Syk/Thbs1/Thbs4/Tnfsf18/Trem3/Trpv4/Xcl1	38
GO:1904018	positive regulation of vasculature development	45/1242	208/18303	2.51E-12	6.88E-10	4.71E-10	Agtr1a/Angpt4/Anxa3/Aqp1/Bmper/Camp/Ccl5/Cd40/Cflar/Cth/Cx3cl1/Cxcr4/Cyp1b1/Dll1/Egr1/Ereg/Ets1/F3/Vegfd/Flt1/Gata2/Gata6/Hdac9/Hipk2/Hk2/Hmga2/Hyal1/Il1a/Il1b/Itga5/Itgax/Itgb3/Lgals3/Mmp9/Pdgfb/Pgf/Plk2/Ptgs2/Ramp2/Rhoj/Sema5a/Thbs1/Tlr3/Tmem100/	45
GO:0033002	muscle cell proliferation	50/1242	249/18303	3.00E-12	7.85E-10	5.37E-10	Akr1b1/Bmp4/Calcrl/Ccl5/Cflar/Cx3cl1/Egr1/Ereg/Ern1/Fgf9/Fgfr2/Flt1/Foxc1/Gata6/Gli1/Gstp1/Hey2/Hmgcr/Hpgd/Igfbp5/Il15/Itga2/Itgb3/Jun/Kcnk2/Mef2c/Mmp9/Nampt/Ncam1/Ndrg2/Nox1/Npr3/Nrg1/Ogn/P2ry6/Pcsk5/Pdgfb/Ppard/Ppargc1a/Ptgir/Ptgs2/Myb/Rbpj/Serpinf2/Stat1/Tenm4/Tgm2/Thbs1/Tnf/Tnfaip3	50

ID	Description	GeneRatio	BgRatio	pvalue	p.adjust	qvalue	geneID	Count
GO:0032102	negative regulation of response to external stimulus	66/1242	388/18303	3.32E-12	8.31E-10	5.68E-10	Abcd1/Ada/Adora1/Adora2a/C1qtnf1/C1qtnf3/Calcrl/Cask/Ccl2/Ccr1/Cd200r1/Cd44/Chid1/Cldn3/Cx3cl1/Dusp10/Epha4/Eppk1/Ets1/Gstp1/Hmgcr/Ier3/Acod1/Ldlr/Lgals9/Micb/Mmp12/Nfkb1/Nlrc5/Nlrp3/Nod2/Pdgfb/Pglyrp1/Pla2g5/Plau/Plxna3/Ppard/Prdm1/Ptger4/Pycard/Rgma/Riok3/Sema3f/Sema4a/Sema4b/Sema5a/Sema6a/Sema6d/Serpinb3a/Serpinb9/Serpinf2/Serping1/Siglec10/Sirpa/Slit2/Snai2/Socs3/Thbd/Thbs1/Tnf/Tnfaip3/Tnfaip6/Tnfrsf1b/Wfdc1/Wnt5a/Xylt1	66
GO:0007584	response to nutrient	57/1242	309/18303	3.58E-12	8.59E-10	5.87E-10	Aacs/Abcb1a/Abcb1b/Acat2/Acsl1/Acsl3/Acsl4/Ada/Ass1/Bdnf/Bmp7/Ccl2/Ccl5/Ccnd1/Cd40/Cd44/Col1a1/Col2a1/Ctrb1/Cxcl10/Cyp11b2/Cyp1b1/Cyp26b1/Cyp27b1/Ercc1/Fzd2/Gclc/Gstp1/Hmgcr/Hmgcs1/Il15/Il1a/Il1b/Itga2/Lipg/Mef2c/Mgp/Mmp1/Mmp9/Nod2/Nos2/Nqo1/Penk/Ppard/Ptgs2/Rbp1/Ryr2/Serpina3n/Sfrp1/Snai2/Star/Stat1/Suox/Timp3/Tnfrsf11b/Vcam1/Vim	57
GO:0071222	cellular response to lipopolysaccharide	56/1242	304/18303	5.87E-12	1.35E-09	9.26E-10	Ankrd1/Ass1/Camp/Ccl2/Ccl20/Ccl3/Ccl5/Cd180/Cd274/Cd40/Cd68/Cfh/Csf2/Csf3/Cx3cl1/Cxcl1/Cxcl10/Cxcl11/Cxcl16/Cxcl2/Cxcl3/Ednrb/Gstp1/Il1a/Il1b/Irak2/Acod1/Jun/Lbp/Mef2c/Mmp1/Mmp9/Nfkb1/Nfkbia/Nfkbib/Nlrp3/Nod2/Nos2/P2ry2/Pde2a/Pde4b/Plau/Ppard/Ppargc1a/Prdm1/Pycard/Ripk2/Sirpa/Star/Stat1/Tlr5/Tnf/Tnfaip3/Tnfrsf1b/Vim/Wnt5a	56
GO:0048659	smooth muscle cell proliferation	41/1242	183/18303	7.23E-12	1.60E-09	1.10E-09	Akr1b1/Bmp4/Calcrl/Ccl5/Cx3cl1/Egr1/Ereg/Ern1/Fgf9/Fgfr2/Flt1/Gstp1/Hmgcr/Hpgd/Igfbp5/Il15/Itga2/Itgb3/Jun/Mef2c/Mmp9/Nampt/Ndrg2/Nox1/Npr3/Ogn/P2ry6/Pcsk5/Pdgfb/Ppard/Ppargc1a/Ptgir/Ptgs2/Myb/Rbpj/Serpinf2/Stat1/Tgm2/Thbs1/Tnf/Tnfaip3	41
GO:0048660	regulation of smooth muscle cell proliferation	40/1242	177/18303	9.76E-12	2.08E-09	1.42E-09	Akr1b1/Bmp4/Calcrl/Ccl5/Cx3cl1/Egr1/Ereg/Ern1/Fgf9/Fgfr2/Flt1/Gstp1/Hmgcr/Hpgd/Igfbp5/Il15/Itga2/Itgb3/Jun/Mef2c/Mmp9/Nampt/Ndrg2/Nox1/Npr3/Ogn/P2ry6/Pdgfb/Ppard/Ppargc1a/Ptgir/Ptgs2/Myb/Rbpj/Serpinf2/Stat1/Tgm2/Thbs1/Tnf/Tnfaip3	40
GO:0032103	positive regulation of response to external stimulus	70/1242	435/18303	1.06E-11	2.18E-09	1.49E-09	Cadm1/Ccl2/Ccl3/Ccl5/Ccr1/Cd180/Cmklr1/Csf1/Cx3cl1/Cxcl1/Cxcl10/Cxcl12/Cxcr4/Cyp27b1/Dapk2/Ereg/Ets1/Fabp4/Ripor2/Vegfd/Gbp5/Gpr4/Il18rap/Il1b/Il23a/Il33/Acod1/Itga2/Lbp/Ldlr/Lgals9/Mmp12/Nfkbia/Nlrc4/Nlrc5/Nod2/Nrg1/Pvr/Pde2a/Pdgfb/Pgf/Pla2g5/Pla2g7/Ptger4/Ptgs2/Pycard/Rarres2/Rasgrp1/Riok3/Ripk2/Scarf1/Sema5a/Serpinf2/Slit2/Tgm2/Thbs1/Thbs4/Tlr2/Tlr3/Tlr6/Tnf/Tnfsf18/Tnip1/Trem3/Trim6/Trpv4/Tslp/Tubb	70
GO:0045785	positive regulation of cell adhesion	71/1242	449/18303	1.73E-11	3.33E-09	2.28E-09	Ada/Alox15/Ank3/Blm/Bmp7/Card11/Ccdc80/Ccl2/Ccl5/Cd274/Cd44/Cd83/Chrd/Coro1a/Csf1/Cspg5/Cx3cl1/Cxcl12/Dock8/Dusp10/Edil3/Emilin1/Enpp2/Ets1/F11r/Fbln1/Flcn/Flot1/Has2/Hyal1/Ibsp/Ikzf1/Il15/Il1a/Il1b/Il23a/Itga2/Itga5/Itgal/Itgb3/Lgals9/Lif/Lilrb4/Ndnf/Net1/Nlrp3/Npnt/Nrg1/Pcsk5/Plaur/Plpp3/Ptger4/Pycard/Myb/Rap1gap/Rasal3/Rasgrp1/Ripk2/Selp/Sirpa/Smoc1/Sox12/Syk/Tgm2/Thbs1/Tnf/Tnfsf18/Vcam1/Vsir/Wnt5a/X	71
GO:0048661	positive regulation of smooth muscle cell proliferation	31/1242	115/18303	1.73E-11	3.33E-09	2.28E-09	Akr1b1/Bmp4/Calcrl/Ccl5/Cx3cl1/Egr1/Ereg/Ern1/Fgf9/Fgfr2/Flt1/Hmgcr/Hpgd/Igfbp5/Itga2/Itgb3/Jun/Mmp9/Nampt/Nox1/P2ry6/Pdgfb/Ppargc1a/Ptgs2/Myb/Rbpj/Serpinf2/Stat1/Tgm2/Thbs1/Tnf	31
GO:0060326	cell chemotaxis	55/1242	305/18303	2.16E-11	4.01E-09	2.74E-09	Agtr1a/Ccl2/Ccl20/Ccl22/Ccl3/Ccl5/Ccl9/Ccr1/Ch25h/Cmklr1/Coro1a/Csf1/Cx3cl1/Cxcl1/Cxcl10/Cxcl11/Cxcl12/Cxcl16/Cxcl2/Cxcl3/Cxcr4/Cyp7b1/Dapk2/Dock4/Ednrb/Enpp2/Ripor2/Vegfd/Flt1/Gstp1/Il1b/Il23a/Jaml/LOC290595/Lbp/Lgals3/Lgals9/Nod2/Pde4b/Pdgfb/Pgf/Pla2g7/Ptpro/Rarres2/Sema5a/Slit2/Syk/Thbs1/Thbs4/Tnfsf18/Trem3/Trpv4/Vcam1/Wnt5a/Xcl1	55
GO:0071621	granulocyte chemotaxis	32/1242	125/18303	3.64E-11	6.56E-09	4.48E-09	Ccl2/Ccl20/Ccl22/Ccl3/Ccl5/Ccl9/Cmklr1/Csf1/Cx3cl1/Cxcl1/Cxcl10/Cxcl11/Cxcl2/Cxcl3/Dapk2/Ripor2/Il1b/Il23a/Jaml/Lbp/Lgals3/Nod2/Pde4b/Rarres2/Slit2/Syk/Thbs1/Thbs4/Tnfsf18/Trem3/Trpv4/Xcl1	32
GO:0071674	mononuclear cell migration	26/1242	87/18303	5.97E-11	1.04E-08	7.14E-09	Ccl2/Ccl20/Ccl22/Ccl3/Ccl5/Ccl9/Ccr1/Cmklr1/Csf1/Cx3cl1/Cxcl10/Cxcl12/Flt1/Jaml/Lgals3/Pdgfb/Pla2g7/Plcb1/Ptpro/Rarres2/Sirpa/Slit2/Thbs1/Tnfsf18/Trpv4/Xcl	26
GO:0045766	positive regulation of angiogenesis	40/1242	188/18303	7.10E-11	1.20E-08	8.23E-09	Agtr1a/Angpt4/Anxa3/Aqp1/Bmper/Camp/Ccl5/Cd40/Cx3cl1/Cxcr4/Cyp1b1/Dll1/Ereg/Ets1/F3/Vegfd/Flt1/Gata2/Gata6/Hdac9/Hipk2/Hk2/Hmga2/Hyal1/Il1a/Il1b/Itga5/Itgax/Itgb3/Lgals3/Mmp9/Pgf/Plk2/Ptgs2/Ramp2/Rhoj/Sema5a/Thbs1/Tlr3/Wnt5a	40
GO:0034612	response to tumor necrosis factor	50/1242	271/18303	7.58E-11	1.25E-08	8.54E-09	Abcb1b/Akap12/Ankrd1/Ass1/Bdnf/Birc3/Camp/Card14/Ccl2/Ccl20/Ccl22/Ccl3/Ccl5/Ccl9/Cd40/Col1a1/Col2a1/Cx3cl1/Cxcl16/Cyp1b1/Fabp4/Gch1/Ggt1/Has2/Hspa1b/Hyal1/Irf1/Acod1/Klf2/Mmp1/Mmp3/Mmp9/Nfkb1/Nfkbia/Nos2/Npnt/Ppargc1a/Ptgs2/Pycard/Sfrp1/Stat1/Syk/Thbs1/Tnf/Tnfrsf1b/Tnfsf18/Traf1/Vcam1/Wfdc21/	50

ID	Description	GeneRatio	BgRatio	pvalue	p.adjust	qvalue	geneID	Count
GO:0071496	cellular response to external stimulus	65/1242	409/18303	1.01E-10	1.62E-08	1.11E-08	Abcb1a/Abcb1b/Acat2/Ankrd1/Aqp1/Asgr1/Atf3/Bmf/Bmp2/Bmp4/Ccl5/Cd40/Cd68/Col1a1/Col2a1/Cpeb4/Cyp11b2/Cyp27b1/Egr1/F11r/Ripor2/Flcn/Fnip1/Fosl1/Fscn1/Fzd2/Gclc/Gstp1/Hfe/Il13ra2/Il15/Il1b/Irf1/Itga2/Itgb3/Jun/Map3k14/Mmp1/Mmp3/Mmp9/Nampt/Nfkb1/Nos2/Pcsk9/Pde2a/Pdgfb/Penk/Piezo2/Pik3c2b/Ppp1r15a/Ptger4/Ptgs2/Rragd/Scx/Sesn3/Sfrp1/Slc1a2/Snai2/Tfeb/Tlr3/Tlr5/Ucn2/Upp1/Vcam1/Wnt2b	65
GO:1903706	regulation of hemopoiesis	66/1242	419/18303	1.07E-10	1.67E-08	1.14E-08	Ada/Bmp4/Card11/Ccl3/Ccl5/Ccr1/Cd44/Cd83/Csf1/Csf2/Csf3/Cxcl1/Cyp26b1/Dll1/Dusp10/Ets1/Faxdc2/Flcn/Flt1/Flt3lg/Fnip1/Foxc1/Foxn1/Gata2/Gpr68/Hoxb8/Hspa1b/Ikzf1/Il15/Il15ra/Il1a/Il1b/Il23a/Inhba/Irf1/Isg15/Jun/Kitlg/LOC102551184/Lgals9/Lif/Lilrb4/Lmo2/Mafb/Mef2c/Nfkbia/Nlrp3/Pglyrp1/Pglyrp4/Prdm1/Myb/RGD1562378/Rasgrp1/Rbm15/Rbp1/Ripk2/Scin/Sfrp1/Sox12/Stat1/Syk/Tnf/Tnfsf18/Tox/Twist2/Vsir	66
GO:1990266	neutrophil migration	31/1242	123/18303	1.13E-10	1.71E-08	1.17E-08	Ccl2/Ccl20/Ccl22/Ccl3/Ccl5/Ccl9/Cd99l2/Cx3cl1/Cxcl1/Cxcl10/Cxcl11/Cxcl2/Cxcl3/Dapk2/Ripor2/Il1a/Il1b/Il1r1/Il23a/Jaml/Lbp/Lgals3/Mcoln2/Nod2/Pde4b/Ptger4/Slit2/Syk/Thbs4/Trem3/Xcl1	31
GO:1901617	organic hydroxy compound biosynthetic process	44/1242	226/18303	1.74E-10	2.57E-08	1.76E-08	Acat2/Adcyap1r1/Agtr1a/Akr1b1/Alox15/Bmp2/Bmp5/Cdh3/Ch25h/Cyp11b2/Cyp27a1/Cyp27b1/Cyp51/Cyp7b1/Dhcr24/Fdft1/Fdps/Gch1/Gpr37/Hand2/Hmgcr/Hmgcs1/Hsd17b7/Idi1/Il1b/Insig1/Msmo1/Mvd/Nfkb1/Nsdhl/P2ry6/Plek/Pltp/Pth1r/Sc5d/Slc7a11/Snai1/Snai2/Sqle/Star/Stard4/Tm7sf2/Tnf/Wnt5a	44
GO:0019221	cytokine-mediated signaling pathway	62/1242	387/18303	1.97E-10	2.84E-08	1.94E-08	Acsl1/Card14/Ccl2/Ccl20/Ccl22/Ccl3/Ccl5/Ccl9/Ccr1/Cd44/Crebrf/Csf1/Cx3cl1/Cxcl1/Cxcl10/Cxcl11/Cxcl12/Cxcl2/Cxcl3/Cxcr4/Egr1/Ereg/F3/Foxc1/Fzd4/Gpr75/Grem2/Hspa1b/Il13ra2/Il15/Il15ra/Il18rap/Il1a/Il1b/Il1r1/Il1rn/Il31ra/Il33/Irak2/Irf1/Klf6/Mmp12/Nfkbia/Nlrc5/Pdgfb/Plcb1/Pycard/Rbm15/Ripk2/Sh2b2/Slit2/Slit3/Stat1/Syk/Tnf/Tnfrsf1b/Tnfsf18/Traf1/Trim6/Tslp/Wnt5a/X	62
GO:0032874	positive regulation of stress-activated MAPK cascade	37/1242	172/18303	2.62E-10	3.69E-08	2.52E-08	Ankrd6/Bmp2/Dusp19/Edar/Epha4/Ern1/Flt4/Fzd4/Fzd5/Fzd8/Gadd45g/Gdf6/Hand2/Hipk2/Hmgcr/Il1a/Il1b/Il1rn/Map3k9/Mdfi/Nod2/Nox1/Plcb1/Pycard/Inava/Rasgrp1/Ripk2/Serpinf2/Syk/Tlr3/Tlr6/Tnf/Tnik/Traf1/Trpv4	37
GO:0001503	ossification	65/1242	420/18303	3.18E-10	4.36E-08	2.98E-08	Adgrv1/Alox15/Areg/Bmp2/Bmp3/Bmp4/Bmp7/Ccl3/Ccr1/Cebpd/Chrd/Clec11a/Col11a1/Col13a1/Col1a1/Col2a1/Creb3l1/Csf1/Cthrc1/Cyp27b1/Dhrs3/Fam20c/Fat4/Fgf9/Fgfr2/Fgr/Foxc1/Gdpd2/Gfra4/Gli1/Hand2/Ibsp/Igfbp5/Isg15/Junb/Mef2c/Mgp/Mmp13/Mmp16/Mmp9/Noct/Npnt/P2ry2/Pcp4/Penk/Ptger4/Ptgs2/Pth1r/RGD1562378/Rbpj/Rorb/Rsad2/Scx/Sfrp1/Slc24a3/Smoc1/Snai1/Snai2/Thra/Thrb/Tnf/Tnn/Twist2/Wnt5a/Xylt1	65
GO:0070304	positive regulation of stress-activated protein kinase signaling cascade	37/1242	174/18303	3.71E-10	4.98E-08	3.40E-08	Ankrd6/Bmp2/Dusp19/Edar/Epha4/Ern1/Flt4/Fzd4/Fzd5/Fzd8/Gadd45g/Gdf6/Hand2/Hipk2/Hmgcr/Il1a/Il1b/Il1rn/Map3k9/Mdfi/Nod2/Nox1/Plcb1/Pycard/Inava/Rasgrp1/Ripk2/Serpinf2/Syk/Tlr3/Tlr6/Tnf/Tnik/Traf1/Trpv4	37
GO:1903708	positive regulation of hemopoiesis	43/1242	225/18303	5.15E-10	6.75E-08	4.62E-08	Ada/Ccl3/Ccl5/Ccr1/Cd83/Csf1/Csf2/Csf3/Cxcl1/Dusp10/Ets1/Faxdc2/Flt1/Flt3lg/Foxc1/Gata2/Gpr68/Hspa1b/Ikzf1/Il15/Il15ra/Il1a/Il1b/Il23a/Inhba/Isg15/Jun/Kitlg/Lgals9/Lif/Lilrb4/Nlrp3/Prdm1/Myb/Rasgrp1/Ripk2/Scin/Sox12/Stat1/Syk/Tnf/Tox/Vsir	43
GO:0032963	collagen metabolic process	28/1242	110/18303	6.98E-10	8.94E-08	6.11E-08	Bmp4/Ccl2/Col1a1/Col5a1/Creb3l1/Cyp2j4/Emilin1/Errfi1/Itga2/Larp6/Mkx/Mmp1/Mmp10/Mmp12/Mmp13/Mmp16/Mmp1b/Mmp3/Mmp9/Pdgfb/Ppard/Myb/Rgcc/Scx/Serpinf2/Tram2/Vim/Vsir	28
GO:0046330	positive regulation of JNK cascade	32/1242	141/18303	1.02E-09	1.28E-07	8.74E-08	Ankrd6/Dusp19/Edar/Epha4/Ern1/Flt4/Fzd4/Fzd5/Fzd8/Gadd45g/Hipk2/Il1a/Il1b/Il1rn/Map3k9/Mdfi/Nod2/Nox1/Plcb1/Pycard/Rasgrp1/Ripk2/Serpinf2/Syk/Tlr3/Tlr6/Tnf/Tnik/Traf1/Trpv4/Wnt5a/Zeb2	32
GO:0030593	neutrophil chemotaxis	26/1242	98/18303	1.07E-09	1.31E-07	8.99E-08	Ccl2/Ccl20/Ccl22/Ccl3/Ccl5/Ccl9/Cx3cl1/Cxcl1/Cxcl10/Cxcl11/Cxcl2/Cxcl3/Dapk2/Ripor2/Il1b/Il23a/Jaml/Lbp/Lgals3/Nod2/Pde4b/Slit2/Syk/Thbs4/Trem3/Xcl1	26
GO:0001818	negative regulation of cytokine production	48/1242	274/18303	1.12E-09	1.34E-07	9.18E-08	Abcd1/Bcl3/C1qtnf3/Cd200r1/Cd274/Cd83/Cdh3/Chid1/Cmklr1/Cx3cl1/Dll1/Errfi1/Flt1/Furin/Gata6/Gstp1/Hdac9/Hfe/Il23a/Il33/Acod1/Klf2/Lbp/Lgals9/Ndrg2/Nfkb1/Nlrp3/Nod2/Pglyrp1/Pglyrp4/Ptger4/Pycard/Rel/Relb/Rgcc/Rnf128/Sirpa/Slc2a10/Ssc5d/Thbs1/Tlr2/Tlr6/Tnf/Tnfaip3/Trib2/Twist2/Vsir/Xcl1	48
GO:0032355	response to estradiol	46/1242	257/18303	1.21E-09	1.43E-07	9.76E-08	Abcb1a/Adcyap1r1/Areg/Ass1/Bmp4/Bmp7/Ccl2/Ccnd1/Cflar/Col1a1/Cxcl1/Cxcl2/Cyp1b1/Drd1/Enpp2/Ets1/F3/Flt1/Ggt1/Gjb2/Gstp1/Hpgd/Hsd17b7/Il1b/Insig1/Itga2/Kif18a/Ldlr/Mmp3/Mmp9/Nefh/Nos2/Npy1r/Nqo1/Pdgfb/Penk/Ppargc1a/Ptgs2/Ramp2/Rgs9/Sfrp1/Socs3/Sprr2d/Vim/Wfdc1/Wnt5a	46

ID	Description	GeneRatio	BgRatio	pvalue	p.adjust	qvalue	geneID	Count
GO:0071356	cellular response to tumor necrosis factor	44/1242	244/18303	2.18E-09	2.52E-07	1.72E-07	Abcb1b/Akap12/Ankrd1/Ass1/Bdnf/Birc3/Camp/Card14/Ccl2/Ccl20/Ccl22/Ccl3/Ccl5/Ccl9/Cd40/Col1a1/Col2a1/Cx3cl1/Cyp1b1/Fabp4/Has2/Hspa1b/Hyal1/Irf1/Acod1/Klf2/Mmp1/Mmp9/Nfkb1/Nfkbia/Nos2/Npnt/Ppargc1a/Pycard/Sfrp1/Stat1/Syk/Thbs1/Tnf/Tnfrsf1b/Tnfsf18/Traf1/Vcam1/Xcl1	44
GO:0070374	positive regulation of ERK1 and ERK2 cascade	42/1242	227/18303	2.27E-09	2.57E-07	1.76E-07	Akap12/Alox15/Bmp2/Bmp4/Bmper/C1qtnf3/Ccl2/Ccl20/Ccl22/Ccl3/Ccl5/Ccl9/Ccr1/Cd44/Cflar/Cx3cl1/Fgfr2/Flt4/Hand2/Hmgcr/Htr2a/Il1a/Il1b/Itgb3/Jun/Lgals9/Ngf/Nod2/Npnt/P2ry6/Pdgfb/Pla2g2a/Pla2g5/Cavin3/Pycard/Rap1b/Rasgrp1/Ripk2/Serpinf2/Tlr2/Trpv4/Xcl1	42
GO:0043405	regulation of MAP kinase activity	52/1242	317/18303	2.50E-09	2.77E-07	1.89E-07	Adora1/Adra2a/Bmp2/Bmp4/Bmp7/Cd40/Cspg4/Dusp10/Dusp16/Dusp19/Dusp5/Dusp6/Epha4/Ephb2/Ern1/Flt1/Fzd4/Fzd5/Fzd8/Gadd45g/Gdf15/Gstp1/Hmgcr/Htr2a/Igfbp6/Il1b/Il1rn/Kitlg/Map3k9/Mdfi/Mst1r/Nek10/Nod2/Nrg1/Pdgfb/Pik3r5/Ptpn5/Inava/Rasgrp1/Rgs14/Ripk2/Serpinb3/Sfrp1/Sprv4/Syk/Thbs1/Tlr6/Tnf/Tnik/Trib2	52
GO:0007254	JNK cascade	39/1242	203/18303	2.80E-09	3.04E-07	2.08E-07	Ankrd6/Cdc42ep5/Dusp10/Dusp19/Edar/Epha4/Ern1/Flt4/Fzd4/Fzd5/Fzd8/Gadd45g/Gstp1/Hipk2/Il1a/Il1b/Il1rn/Map3k9/Mdfi/Nod2/Nox1/Plcb1/Ptger4/Pycard/Rasgrp1/Ripk2/Serpinb3/Serpinf2/Sfrp1/Sirpa/Syk/Tlr3/Tlr6/Tnf/Tnik/Traf1/Trpv4/Wnt5a/Zeb2	39
GO:0046328	regulation of JNK cascade	37/1242	188/18303	3.58E-09	3.82E-07	2.61E-07	Ankrd6/Dusp10/Dusp19/Edar/Epha4/Ern1/Flt4/Fzd4/Fzd5/Fzd8/Gadd45g/Gstp1/Hipk2/Il1a/Il1b/Il1rn/Map3k9/Mdfi/Nod2/Nox1/Plcb1/Pycard/Rasgrp1/Ripk2/Serpinb3/Serpinf2/Sfrp1/Sirpa/Syk/Tlr3/Tlr6/Tnf/Tnik/Traf1/Trpv4/Wnt5a/Zeb2	37
GO:0071900	regulation of protein serine/threonine kinase activity	69/1242	486/18303	3.88E-09	4.00E-07	2.73E-07	Acsl1/Adora1/Adra2a/Als2/Blm/Bmp2/Bmp4/Bmp7/Ccna1/Ccnd1/Ccno/Cd40/Cemip/Cks2/Cspg4/Dusp10/Dusp16/Dusp19/Dusp5/Dusp6/Epha4/Ephb2/Ern1/Flt1/Fzd4/Fzd5/Fzd8/Gadd45g/Gdf15/Gstp1/Hmga2/Hmgcr/Htr2a/Igfbp6/Il1b/Il1rn/Kitlg/Lgals9/Map3k9/Mdfi/Mst1r/Nek10/Nod2/Nos2/Nrg1/Pdgfb/Pik3r5/Pmp22/Prkar2a/Prkar2b/Ptpn5/Pycard/Inava/Rasgrp1/Rgcc/Rgs14/Ripk2/Serpinb3/Sfrp1/Sprv4/Syk/Thbs1/Tlr6/Tnf/Tnfaip3/Tn	69
GO:0014812	muscle cell migration	28/1242	118/18303	3.88E-09	4.00E-07	2.73E-07	Ccl5/Cyp1b1/Dock4/Egr1/F3/Fgf9/Gstp1/Has2/Igfbp5/Itga2/Itgb3/LOC100910418/Mef2c/Mmp1/Net1/P2ry2/P2ry6/Pcsk5/Pdgfb/Plat/Plau/Ppard/Ppargc1a/Ptger4/Rbpj/Sema6d/Slit2/Thbs4	28
GO:0032964	collagen biosynthetic process	19/1242	58/18303	4.09E-09	4.14E-07	2.83E-07	Bmp4/Ccl2/Col1a1/Col5a1/Creb3l1/Cyp2j4/Emilin1/Errfi1/Itga2/Larp6/Mkx/Pdgfb/Ppard/Myb/Rgcc/Scx/Serpinf2/Tram2/Vim	19
GO:0014909	smooth muscle cell migration	26/1242	104/18303	4.27E-09	4.24E-07	2.90E-07	Ccl5/Cyp1b1/Dock4/Egr1/F3/Fgf9/Gstp1/Has2/Igfbp5/Itga2/Itgb3/LOC100910418/Mef2c/Mmp1/P2ry2/P2ry6/Pcsk5/Pdgfb/Plat/Plau/Ppard/Ppargc1a/Ptger4/Rbpj/Sem	26
GO:0001763	morphogenesis of a branching structure	43/1242	241/18303	4.63E-09	4.53E-07	3.10E-07	Agtr1a/Areg/Bmp2/Bmp4/Bmp7/Cd44/Col13a1/Col4a1/Csf1/Csmd1/Ctnnd2/Cxcl12/Cxcr4/Esrp2/Fat4/Fgfr2/Flt1/Fzd5/Gbx2/Gna13/Hmga2/Hnf1b/Il1b/Mgp/Npnt/Ntn4/Pax8/Pdgfb/Pgf/Plxnd1/Prdm1/Rbm15/Sema5a/Sfrp1/Slit2/Snai2/Socs3/St14/Tbx3/Tgm2/Tnf/Wnt2b/Wnt5a	43
GO:1990868	response to chemokine	23/1242	84/18303	5.02E-09	4.75E-07	3.25E-07	Ccl2/Ccl20/Ccl22/Ccl3/Ccl5/Ccl9/Ccr1/Cx3cl1/Cxcl1/Cxcl10/Cxcl11/Cxcl12/Cxcl2/Cxcl3/Cxcr4/Dock8/Ripor2/Foxc1/Gpr75/Rbm15/Slit2/Slit3/Xcl1	23
GO:1990869	cellular response to chemokine	23/1242	84/18303	5.02E-09	4.75E-07	3.25E-07	Ccl2/Ccl20/Ccl22/Ccl3/Ccl5/Ccl9/Ccr1/Cx3cl1/Cxcl1/Cxcl10/Cxcl11/Cxcl12/Cxcl2/Cxcl3/Cxcr4/Dock8/Ripor2/Foxc1/Gpr75/Rbm15/Slit2/Slit3/Xcl1	23
GO:0006694	steroid biosynthetic process	33/1242	158/18303	5.30E-09	4.92E-07	3.37E-07	Acat2/Bmp2/Bmp5/Ch25h/Cyp11b2/Cyp27a1/Cyp27b1/Cyp51/Cyp7b1/Dhcr24/Egr1/Fdft1/Fdps/Hmgcr/Hmgcs1/Hsd17b7/Idi1/Il1a/Il1b/Insig1/Msmo1/Mvd/Nfkb1/Nsdhl/Ppargc1a/Sc5d/Snai1/Snai2/Sqle/Star/Stard4/Tm7sf	33
GO:0072676	lymphocyte migration	26/1242	106/18303	6.58E-09	5.96E-07	4.08E-07	Cadm1/Ccl2/Ccl20/Ccl22/Ccl3/Ccl5/Ccl9/Cd200r1/Cd99l2/Ch25h/Cx3cl1/Cxcl10/Cxcl11/Cxcl12/Cxcl16/Cyp7b1/Dock8/F11r/Ripor2/Itgal/Itgb3/LOC290595/Lgals9/Pycard/Wnt5a/Xcl1	26
GO:0036293	response to decreased oxygen levels	66/1242	462/18303	6.62E-09	5.96E-07	4.08E-07	Abcb1a/Abcb1b/Ada/Adora1/Angpt4/Angptl4/Ankrd1/Aqp1/Bach1/Bdkrb2/Bdnf/Bmp2/Casp12/Ccl2/Cflar/Cldn3/Cox4i2/Cpeb2/Cpeb4/Cx3cl1/Cxcl12/Cxcr4/Drd1/Egln3/Egr1/Ets1/Vegfd/Flt1/Fndc1/Gata6/Hilpda/Hk2/Il1a/Il1b/Itga2/Kcnk2/Kcnk3/Ldlr/Mmp12/Mmp13/Mmp3/Mmp9/Nampt/Ndnf/Nos2/Pdgfb/Penk/Pgf/Plat/Plau/Plod2/Ppard/Ppargc1a/Ppp1r15a/Ptgs2/Myb/Ramp2/Rbpj/Rgcc/Ryr2/Sfrp1/Socs3/Tlr2/Tnf/Ucn2/Vcam1	66
GO:0072678	T cell migration	20/1242	66/18303	7.39E-09	6.55E-07	4.48E-07	Ccl2/Ccl20/Ccl3/Ccl5/Cd200r1/Cd99l2/Cxcl10/Cxcl11/Cxcl12/Cxcl16/Dock8/F11r/Ripor2/Itgal/Itgb3/LOC290595/Lgals9/Pycard/Wnt5a/Xcl1	20

ID	Description	GeneRatio	BgRatio	pvalue	p.adjust	qvalue	geneID	Count
GO:0040013	negative regulation of locomotion	54/1242	346/18303	7.53E-09	6.57E-07	4.50E-07	Ada/Adora1/Adora2a/Angpt4/Arap3/Arhgdib/Arrdc3/Ccl2/Cd200r1/Chrd/Cldn3/Col3a1/Cx3cl1/Cxcl12/Cyp1b1/Dcn/Drd1/Dusp10/Emilin1/Eppk1/Ripor2/Fbln1/Flcn/Fuz/Gstp1/Igfbp5/Il1rn/Il33/Limch1/Mef2c/Meox2/Nrg1/Pdgfb/Plcb1/Plxna3/Ppard/Ppargc1a/Ptger4/Ptprt/Rbpj/Rgcc/Sema3f/Sema4a/Sema4b/Sema5a/Sema6a/Sema6d/Sfrp1/Slit2/Srgap1/Thbs1/Tnf/Tnn/Wnt5a	54
GO:0033273	response to vitamin	35/1242	177/18303	8.29E-09	7.10E-07	4.86E-07	Abcb1a/Abcb1b/Ada/Bdnf/Bmp7/Ccl2/Ccl5/Ccnd1/Cd40/Cd44/Col1a1/Col2a1/Cxcl10/Cyp26b1/Cyp27b1/Fzd2/Gstp1/Hmgcs1/Il15/Il1a/Il1b/Itga2/Mef2c/Mmp1/Mmp9/Nos2/Penk/Ppard/Ptgs2/Rbp1/Serpina3n/Sfrp1/Snai2/	35
GO:0015908	fatty acid transport	25/1242	100/18303	8.44E-09	7.10E-07	4.86E-07	Abcd1/Acsl1/Acsl3/Acsl4/Bdkrb2/Cpt1b/Erfe/Fabp3/Fabp4/Fabp5/Il1a/Il1b/Nos2/P2ry2/Pla2g2a/Pla2g5/Pla2r1/Plin2/Ppard/Ptges/Slc27a3/Slco3a1/Syk/Thbs1/Trpv4	25
GO:0032872	regulation of stress-activated MAPK cascade	42/1242	237/18303	8.60E-09	7.10E-07	4.86E-07	Ankrd6/Bmp2/Dusp10/Dusp19/Edar/Epha4/Ern1/Flt4/Fzd4/Fzd5/Fzd8/Gadd45g/Gdf6/Gstp1/Hand2/Hipk2/Hmgcr/Il1a/Il1b/Il1rn/Map3k9/Mdfi/Nod2/Nox1/Plcb1/Pycard/Inava/Rasgrp1/Ripk2/Serpinb3/Serpinf2/Sfrp1/Sirpa/Syk/Tlr3/Tlr6/Tnf/Tnik/Traf1/Trpv4/Wnt5a/Zeb2	42
GO:0050728	negative regulation of inflammatory response	32/1242	153/18303	8.69E-09	7.10E-07	4.86E-07	Abcd1/Ada/Adora1/Adora2a/C1qtnf3/Calcrl/Cd200r1/Cd44/Chid1/Cx3cl1/Dusp10/Ets1/Ier3/Acod1/Ldlr/Lgals9/Nfkb1/Nlrp3/Nod2/Pglyrp1/Pla2g5/Ppard/Ptger4/Pycard/Siglec10/Sirpa/Slit2/Socs3/Tnfaip3/Tnfaip6/Tnfrsf1b/	32
GO:0070098	chemokine-mediated signaling pathway	21/1242	73/18303	8.84E-09	7.10E-07	4.86E-07	Ccl2/Ccl20/Ccl22/Ccl3/Ccl5/Ccl9/Ccr1/Cx3cl1/Cxcl1/Cxcl10/Cxcl11/Cxcl12/Cxcl2/Cxcl3/Cxcr4/Foxc1/Gpr75/Rbm15/Slit2/Slit3/Xcl1	21
GO:0051403	stress-activated MAPK cascade	45/1242	264/18303	8.87E-09	7.10E-07	4.86E-07	Ankrd6/Bmp2/Cdc42ep5/Dusp10/Dusp19/Edar/Epha4/Ern1/Flt4/Fzd4/Fzd5/Fzd8/Gadd45g/Gdf6/Gstp1/Hand2/Hipk2/Hmgcr/Il1a/Il1b/Il1rn/Lgals9/Map3k9/Mdfi/Nod2/Nox1/Plcb1/Ptger4/Pycard/Inava/Rasgrp1/Ripk2/Serpinb3/Serpinf2/Sfrp1/Sirpa/Syk/Tlr3/Tlr6/Tnf/Tnik/Traf1/Trpv4/Wnt5a/Zeb2	45
GO:0051480	regulation of cytosolic calcium ion concentration	60/1242	406/18303	9.13E-09	7.21E-07	4.93E-07	Adcy8/Adcyap1r1/Adora1/Agtr1a/Ank2/Bdkrb1/Bdkrb2/Bmp4/C1qtnf1/Cacna1g/Cacnb2/Casq1/Ccl3/Ccr1/Cemip/Cmklr1/Cngb1/Coro1a/Cx3cl1/Cxcl1/Cxcl10/Cxcl11/Cxcl2/Cxcl3/Cxcr4/Drd1/Ednrb/Fzd2/Gata2/Gna13/Gng3/Gpr4/Grin2a/Hcrtr2/Htr2a/Il1b/Itgb3/Kcnh1/Kcnk3/Mcoln2/Mcoln3/Ngf/Npy1r/P2ry2/P2ry6/Ptger1/Ptger4/Ptgir/Pth1r/RGD1560455/Rxfp3/Ryr2/Ryr3/Slc35g1/Tgm2/Trpc4/Trpv3/Trpv4/Wnt5a/Xcl1	60
GO:0034341	response to interferon-gamma	32/1242	155/18303	1.21E-08	9.43E-07	6.45E-07	Ass1/Ccl2/Ccl20/Ccl22/Ccl3/Ccl5/Ccl9/Cd40/Cfh/Ciita/Cx3cl1/Cxcl16/Cyp27b1/Gbp4/Gbp5/Gch1/Irf1/Acod1/Lgals9/Nlrc5/Nos2/Rab20/Serpina3n/Sirpa/Star/Stat1/Tlr2/Tlr3/Tnf/Vim/Wnt5a/Xcl1	32
GO:0070302	regulation of stress-activated protein kinase signaling cascade	42/1242	240/18303	1.26E-08	9.67E-07	6.61E-07	Ankrd6/Bmp2/Dusp10/Dusp19/Edar/Epha4/Ern1/Flt4/Fzd4/Fzd5/Fzd8/Gadd45g/Gdf6/Gstp1/Hand2/Hipk2/Hmgcr/Il1a/Il1b/Il1rn/Map3k9/Mdfi/Nod2/Nox1/Plcb1/Pycard/Inava/Rasgrp1/Ripk2/Serpinb3/Serpinf2/Sfrp1/Sirpa/Syk/Tlr3/Tlr6/Tnf/Tnik/Traf1/Trpv4/Wnt5a/Zeb2	42
GO:0002690	positive regulation of leukocyte chemotaxis	25/1242	102/18303	1.30E-08	9.88E-07	6.76E-07	Ccl2/Ccl3/Ccl5/Ccr1/Cmklr1/Csf1/Cx3cl1/Cxcl10/Cxcl12/Dapk2/Ripor2/Vegfd/Il1b/Il23a/Lbp/Lgals9/Pgf/Pla2g7/Rarres2/Thbs1/Thbs4/Tnfsf18/Trpv4/Wnt5a/Xcl1	25
GO:0014910	regulation of smooth muscle cell migration	24/1242	95/18303	1.34E-08	9.90E-07	6.77E-07	Ccl5/Cyp1b1/Dock4/Egr1/F3/Fgf9/Gstp1/Has2/Igfbp5/Itga2/Itgb3/Mef2c/Mmp1/P2ry2/P2ry6/Pcsk5/Pdgfb/Plau/Ppard/Ppargc1a/Ptger4/Rbpj/Sema6d/Slit2	24
GO:0001666	response to hypoxia	62/1242	430/18303	1.34E-08	9.90E-07	6.77E-07	Abcb1a/Abcb1b/Ada/Adora1/Angpt4/Angptl4/Ankrd1/Aqp1/Bach1/Bdkrb2/Bdnf/Bmp2/Ccl2/Cflar/Cldn3/Cox4i2/Cpeb2/Cx3cl1/Cxcl12/Cxcr4/Drd1/Egln3/Egr1/Ets1/Vegfd/Flt1/Fndc1/Gata6/Hilpda/Hk2/Il1a/Il1b/Itga2/Kcnk2/Kcnk3/Ldlr/Mmp12/Mmp13/Mmp3/Mmp9/Ndnf/Nos2/Pdgfb/Penk/Pgf/Plat/Plau/Plod2/Ppard/Ppargc1a/Ptgs2/Myb/Ramp2/Rbpj/Rgcc/Ryr2/Sfrp1/Socs3/Tlr2/Tnf/	62
GO:0002548	monocyte chemotaxis	18/1242	56/18303	1.47E-08	1.07E-06	7.34E-07	Ccl2/Ccl20/Ccl22/Ccl3/Ccl5/Ccl9/Ccr1/Cx3cl1/Cxcl10/Cxcl12/Flt1/Lgals3/Pdgfb/Pla2g7/Ptpro/Slit2/Tnfsf18/X	18
GO:0031098	stress-activated protein kinase signaling cascade	46/1242	278/18303	1.57E-08	1.13E-06	7.74E-07	Akr1b1/Ankrd6/Bmp2/Cdc42ep5/Dusp10/Dusp19/Edar/Epha4/Ern1/Flt4/Fzd4/Fzd5/Fzd8/Gadd45g/Gdf6/Gstp1/Hand2/Hipk2/Hmgcr/Il1a/Il1b/Il1rn/Lgals9/Map3k9/Mdfi/Nod2/Nox1/Plcb1/Ptger4/Pycard/Inava/Rasgrp1/Ripk2/Serpinb3/Serpinf2/Sfrp1/Sirpa/Syk/Tlr3/Tlr6/Tnf/Tnik/Traf1/Trpv4/Wnt5a/Zeb2	46
GO:0022409	positive regulation of cell-cell adhesion	45/1242	269/18303	1.60E-08	1.13E-06	7.74E-07	Ada/Alox15/Ank3/Blm/Bmp7/Card11/Ccl2/Ccl5/Cd274/Cd44/Cd83/Coro1a/Cx3cl1/Dock8/Dusp10/Ets1/F11r/Flot1/Has2/Ikzf1/Il15/Il1a/Il1b/Il23a/Itgal/Lgals9/Lilrb4/Nlrp3/Plaur/Plpp3/Pycard/Myb/Rap1gap/Rasal3/Rasgrp1/Ripk2/Selp/Sirpa/Sox12/Syk/Tnf/Vcam1/Vsir/Wnt5a/	45
GO:0016125	sterol metabolic process	29/1242	133/18303	1.61E-08	1.13E-06	7.74E-07	Acat2/Ch25h/Cyp11b2/Cyp26b1/Cyp27a1/Cyp51/Cyp7b1/Dhcr24/Fdft1/Fdps/Fmo5/Hmgcr/Hmgcs1/Hsd17b7/Idi1/Insig1/Ldlr/Msmo1/Mvd/Nsdhl/Pcsk9/Pmp22/Sc5d/Sqle/Star/Thrb/Tm7sf2/Pip4p1/Ttc39b	29

ID	Description	GeneRatio	BgRatio	pvalue	p.adjust	qvalue	geneID	Count
GO:0051271	negative regulation of cellular component movement	53/1242	345/18303	1.79E-08	1.25E-06	8.52E-07	Ada/Adora1/Angpt4/Arap3/Arhgdib/Ccl2/Cd200r1/Chrd/Cldn3/Cnih2/Col3a1/Cx3cl1/Cxcl12/Cyp1b1/Dcn/Drd1/Dusp10/Emilin1/Eppk1/Ripor2/Fbln1/Flcn/Fuz/Gstp1/Igfbp5/Il1rn/Il33/Limch1/Mef2c/Meox2/Nrg1/Pdgfb/Plcb1/Plxna3/Ppard/Ppargc1a/Ptger4/Ptprt/Rbpj/Rgcc/Sema3f/Sema4a/Sema4b/Sema5a/Sema6a/Sema6d/Sfrp1/Slit2/Srgap1/Thbs1/Tnf/Tnn/Wnt5a	53
GO:0008203	cholesterol metabolic process	28/1242	126/18303	1.83E-08	1.25E-06	8.58E-07	Acat2/Ch25h/Cyp11b2/Cyp27a1/Cyp51/Cyp7b1/Dhcr24/Fdft1/Fdps/Fmo5/Hmgcr/Hmgcs1/Hsd17b7/Idi1/Insig1/Ldlr/Msmo1/Mvd/Nsdhl/Pcsk9/Pmp22/Sc5d/Sqle/Star/Thrb/Tm7sf2/Pip4p1/Ttc39b	28
GO:0022407	regulation of cell-cell adhesion	62/1242	434/18303	1.91E-08	1.29E-06	8.85E-07	Ada/Adora2a/Akna/Alox15/Ank3/Ass1/Blm/Bmp2/Bmp4/Bmp7/C1qtnf1/Card11/Ccl2/Ccl5/Cd274/Cd44/Cd83/Coro1a/Cx3cl1/Cxcl12/Dock8/Dusp10/Ephb3/Ets1/F11r/Ripor2/Flot1/Has2/Hfe/Ikzf1/Il15/Il1a/Il1b/Il1rn/Il23a/Irf1/Itgal/Lgals3/Lgals9/Lilrb4/Nlrp3/Peli1/Plaur/Plpp3/Pycard/Myb/Rap1gap/Rasal3/Rasgrp1/Rgcc/Ripk2/Selp/Sirpa/Sox12/Syk/Tnf/Tnfsf18/Trpv4/Vcam1/Vsir/Wnt5	62
GO:0051091	positive regulation of DNA-binding transcription factor activity	44/1242	263/18303	2.30E-08	1.54E-06	1.06E-06	Adcy8/Anxa3/Bdnf/Card11/Card14/Cd40/Cflar/Csf3/Cth/Cx3cl1/Flot1/Fosl1/Fzd2/Fzd4/Hipk2/Hmga2/Hspa1b/Il18rap/Il1b/Irak2/Jun/Lgals9/Lrp8/Ngf/Nlrc4/Nlrp3/Nod2/Plpp3/Ppargc1a/Pycard/Rgcc/Ripk2/Ror1/Slco3a1/Tlr2/Tlr3/Tlr6/Tnf/Tnfsf18/Traf1/Traf5/Trim6/Trim68/	44
GO:0031348	negative regulation of defense response	40/1242	228/18303	2.59E-08	1.72E-06	1.17E-06	Abcd1/Ada/Adora1/Adora2a/C1qtnf3/Calcrl/Ccr1/Cd200r1/Cd44/Chid1/Cx3cl1/Dusp10/Ets1/Ier3/Acod1/Ldlr/Lgals9/Micb/Mmp12/Nfkb1/Nlrc5/Nlrp3/Nod2/Pglyrp1/Pla2g5/Ppard/Ptger4/Pycard/Riok3/Serpinb3a/Serpinb9/Serping1/Siglec10/Sirpa/Slit2/Socs3/Tnfaip3/Tnfaip6/Tnfrsf1b/Wfdc1	40
GO:0071346	cellular response to interferon-gamma	28/1242	128/18303	2.63E-08	1.72E-06	1.18E-06	Ass1/Ccl2/Ccl20/Ccl22/Ccl3/Ccl5/Ccl9/Cfh/Ciita/Cx3cl1/Gbp4/Gbp5/Irf1/Acod1/Lgals9/Nlrc5/Nos2/Rab20/Serpina3n/Sirpa/Star/Stat1/Tlr2/Tlr3/Tnf/Vim/Wnt5a/Xcl1	28
GO:0071622	regulation of granulocyte chemotaxis	18/1242	58/18303	2.74E-08	1.76E-06	1.20E-06	Ccl2/Ccl5/Cmklr1/Csf1/Cx3cl1/Dapk2/Ripor2/Il1b/Il23a/Lbp/Nod2/Rarres2/Slit2/Thbs1/Thbs4/Tnfsf18/Trpv4/Xcl1	18
GO:0046942	carboxylic acid transport	54/1242	359/18303	2.75E-08	1.76E-06	1.20E-06	Abcd1/Acsl1/Acsl3/Acsl4/Adora1/Adora2a/Bdkrb2/Bdnf/Cpt1b/Erfe/Fabp3/Fabp4/Fabp5/Il1a/Il1b/Il1rn/Lrrc8c/Lrrc8d/Nfkbie/Nos2/P2ry2/Pla2g2a/Pla2g5/Pla2r1/Plin2/Ppard/Ptges/Slc16a12/Slc16a14/Slc16a6/Slc17a1/Slc17a3/Slc17a6/Slc1a1/Slc1a2/Slc22a4/Slc25a48/Slc26a10/Slc26a4/Slc26a9/Slc27a3/Slc47a1/Slc6a12/Slc6a17/Slc7a11/Slc7a2/Slc7a3/Slc7a8/Slco3a1/Syk/Thbs1/Tnf/Trp	54
GO:1902652	secondary alcohol metabolic process	29/1242	137/18303	3.24E-08	2.05E-06	1.40E-06	Acat2/Ch25h/Cyp11b2/Cyp27a1/Cyp27b1/Cyp51/Cyp7b1/Dhcr24/Fdft1/Fdps/Fmo5/Hmgcr/Hmgcs1/Hsd17b7/Idi1/Insig1/Ldlr/Msmo1/Mvd/Nsdhl/Pcsk9/Pmp22/Sc5d/Sqle/Star/Thrb/Tm7sf2/Pip4p1/Ttc39b	29
GO:0050731	positive regulation of peptidyl-tyrosine phosphorylation	37/1242	204/18303	3.50E-08	2.19E-06	1.50E-06	Adora1/Adra2a/Angpt4/Areg/Ccl5/Cd40/Cd44/Clcf1/Csf2/Csf3/Cspg4/Efna1/Enpp2/Epha4/Ereg/Htr2a/Il11/Il15/Il23a/Il31ra/Itga5/Itgb3/Kitlg/Lif/Lrp8/Nod2/Nrg1/Pdgfb/Plpp3/Ptger4/Ripk2/Syk/Thbs4/Tnf/Tnfsf18/Trpv4/T	37
GO:0061138	morphogenesis of a branching epithelium	39/1242	222/18303	3.71E-08	2.30E-06	1.57E-06	Agtr1a/Areg/Bmp2/Bmp4/Bmp7/Cd44/Col4a1/Csf1/Csmd1/Cxcl12/Cxcr4/Esrp2/Fat4/Fgfr2/Flt1/Fzd5/Gbx2/Gna13/Hmga2/Hnf1b/Mgp/Npnt/Ntn4/Pax8/Pdgfb/Pgf/Plxnd1/Rbm15/Sema5a/Sfrp1/Slit2/Snai2/Socs3/St14/Tbx3/Tgm2/Tnf/Wnt2b/Wnt5a	39
GO:0015849	organic acid transport	54/1242	363/18303	4.03E-08	2.47E-06	1.69E-06	Abcd1/Acsl1/Acsl3/Acsl4/Adora1/Adora2a/Bdkrb2/Bdnf/Cpt1b/Erfe/Fabp3/Fabp4/Fabp5/Il1a/Il1b/Il1rn/Lrrc8c/Lrrc8d/Nfkbie/Nos2/P2ry2/Pla2g2a/Pla2g5/Pla2r1/Plin2/Ppard/Ptges/Slc16a12/Slc16a14/Slc16a6/Slc17a1/Slc17a3/Slc17a6/Slc1a1/Slc1a2/Slc22a4/Slc25a48/Slc26a10/Slc26a4/Slc26a9/Slc27a3/Slc47a1/Slc6a12/Slc6a17/Slc7a11/Slc7a2/Slc7a3/Slc7a8/Slco3a1/Syk/Thbs1/Tnf/Trp	54
GO:0016126	sterol biosynthetic process	17/1242	54/18303	5.24E-08	3.18E-06	2.17E-06	Acat2/Ch25h/Cyp51/Dhcr24/Fdft1/Fdps/Hmgcr/Hmgcs1/Hsd17b7/Idi1/Insig1/Msmo1/Mvd/Nsdhl/Sc5d/Sqle/T	17
GO:0001655	urogenital system development	60/1242	426/18303	5.43E-08	3.26E-06	2.23E-06	Adamts6/Agtr1a/Akr1b1/Aqp1/Ass1/Bdnf/Bmp2/Bmp4/Bmp7/Bmper/Cd44/Cfh/Cflar/Col4a1/Crip1/Cxcr4/Cyp26b1/Cyp7b1/Dact2/Dcn/Dll1/Egr1/Epha4/Ephb2/Ephb3/Fat4/Fgfr2/Flcn/Foxc1/Gata2/Gcnt1/Gli1/Gpr4/Has2/Hnf1b/Hpgd/Irx3/Itgb3/Lgr5/Lif/Mef2c/Mmp9/Npnt/Pax8/Pcsk5/Pcsk9/Pdgfb/Pgf/Plaur/Plxnd1/Prdm1/Ptpro/Sfrp1/Slit2/Stat1/Thra/Tiparp/Wnk4/Wnt2b/Wnt5a	60
GO:0050673	epithelial cell proliferation	63/1242	459/18303	6.67E-08	3.96E-06	2.71E-06	Agtr1a/Areg/Bmp2/Bmp4/Bmp5/Bmper/Cask/Ccl2/Ccl5/Ccnd1/Cdh3/Cflar/Col8a2/Csf2/Cxcl12/Cyp7b1/Dusp10/Ednrb/Eppk1/Ereg/Ern1/Errfi1/Esrp2/F3/Fgf9/Fgfr2/Flt1/Flt4/Gas1/Gata2/Gli1/Has2/Hmga2/Hnf1b/Hyal1/Igfbp4/Igfbp5/Itgb3/Jaml/Jun/Lgr5/Mef2c/Mmp12/Nod2/Nos2/Pdgfb/Pgf/Pik3cb/Plau/Ppard/Rap1gap/Rgcc/Sema5a/Sfrp1/Snai2/Stat1/Thbs1/Thbs4/Tnf/Tnfaip3/Twist2	63

ID	Description	GeneRatio	BgRatio	pvalue	p.adjust	qvalue	geneID	Count
GO:0050921	positive regulation of chemotaxis	31/1242	158/18303	7.04E-08	4.14E-06	2.83E-06	Ccl2/Ccl3/Ccl5/Ccr1/Cmklr1/Csf1/Cx3cl1/Cxcl10/Cxcl12/Cxcr4/Dapk2/Ripor2/Vegfd/Il1b/Il23a/Itga2/Lbp/Lgals9/Pdgfb/Pgf/Pla2g7/Rarres2/Sema5a/Slit2/Thbs1/Thbs4/Tnfsf18/Trpv4/Tubb2b/Wnt5a/Xcl1	31
GO:0032965	regulation of collagen biosynthetic process	16/1242	49/18303	7.25E-08	4.22E-06	2.89E-06	Bmp4/Ccl2/Creb3l1/Cyp2j4/Emilin1/Errfi1/Itga2/Larp6/Mkx/Pdgfb/Ppard/Myb/Rgcc/Scx/Serpinf2/Vim	16
GO:0007249	I-kappaB kinase/NF-kappaB signaling	43/1242	265/18303	8.19E-08	4.72E-06	3.23E-06	Bcl3/Card11/Cd40/Cflar/Cth/Cx3cl1/Edar/Gstp1/Il1a/Il1b/Irak2/Irf1/Lgals9/Lurap1l/Map3k14/Nfkbia/Nod2/Peli1/Plk2/Pycard/Inava/Rel/Relb/Riok3/Ripk2/Ror1/Sirpa/Slc20a1/Slc44a2/Stat1/Tgm2/Tifa/Tlr2/Tlr3/Tlr6/Tnf/Tnfaip3/Tnip1/Traf1/Traf5/Trim6/Trim68/Wnt5a	43
GO:0002683	negative regulation of immune system process	64/1242	472/18303	8.29E-08	4.73E-06	3.24E-06	Ada/Adora1/Adora2a/Alox15/Bmp4/Ccl2/Ccl3/Ccr1/Cd200r1/Cd274/Cd44/Cd68/Col3a1/Cx3cl1/Cxcl12/Dll1/Dusp10/Emilin1/Ripor2/Fgr/Gata2/Gpr68/Hfe/Hoxb8/Il13ra2/Il33/Irf1/Acod1/Kitlg/LOC102551184/Ldlr/Lgals3/Lgals9/Lilrb4/Lmo2/Lpxn/Mafb/Micb/Mmp12/Nfkbia/Nlrc5/Nod2/Peli1/Pglyrp1/Pglyrp4/Plcb1/Prdm1/Ptger4/RGD1562378/Rbm15/Riok3/Serpinb3a/Serpinb9/Serping1/Sfrp1/Siglec10/Slit2/Thbs1/Tnf/Tnfaip3/Tnfsf18/T	64
GO:0046165	alcohol biosynthetic process	29/1242	143/18303	8.73E-08	4.89E-06	3.35E-06	Acat2/Adcyap1r1/Akr1b1/Bmp2/Bmp5/Cyp11b2/Cyp27a1/Cyp27b1/Cyp51/Dhcr24/Fdft1/Fdps/Gch1/Hmgcr/Hmgcs1/Hsd17b7/Idi1/Il1b/Insig1/Msmo1/Mvd/Nfkb1/Nsdhl/P2ry6/Plek/Pth1r/Sc5d/Tm7sf2/Tnf	29
GO:0050920	regulation of chemotaxis	40/1242	238/18303	8.74E-08	4.89E-06	3.35E-06	Ccl2/Ccl3/Ccl5/Ccr1/Cmklr1/Csf1/Cx3cl1/Cxcl10/Cxcl12/Cxcr4/Dapk2/Ripor2/Vegfd/Gstp1/Il1b/Il23a/Itga2/Lbp/Lgals9/Nod2/Pdgfb/Pgf/Pla2g7/Plxna3/Rarres2/Sema3f/Sema4a/Sema4b/Sema5a/Sema6a/Sema6d/Slit2/Thbs1/Thbs4/Tnfsf18/Trpv4/Tubb2b/Wnt5a/Xcl1/Zswim5	40
GO:0043270	positive regulation of ion transport	51/1242	342/18303	8.84E-08	4.90E-06	3.35E-06	Abcb1a/Adcyap1r1/Adora1/Adora2a/Adra2a/Agtr1a/Ank2/Ank3/Bdkrb1/Bmp4/Cacnb2/Cask/Casq1/Ccl2/Ccl3/Ccl5/Ccr1/Cemip/Cx3cl1/Cxcl1/Cxcl10/Cxcl11/Cxcl12/Cxcr4/Drd1/Ephb2/Erfe/Fabp3/Fhl1/Hcn1/Il1a/Il1b/Kcnh2/Lgals3/Lrrc38/Mylk/Nat8l/P2ry2/P2ry6/Pcp4/Pdgfb/Pla2r1/Ptges/Rgs7/Rgs9/Ryr2/Sgk1/Trpv3/Trpv4/Wnk4	51
GO:0031349	positive regulation of defense response	45/1242	285/18303	9.40E-08	5.16E-06	3.53E-06	Cadm1/Ccl3/Ccl5/Cx3cl1/Cxcl1/Ereg/Ets1/Fabp4/Gbp5/Gpr4/Il18rap/Il1b/Il33/Itga2/Lbp/Ldlr/Lgals9/Mef2c/Mmp12/Nfkbia/Nlrc4/Nlrc5/Nod2/Pvr/Pde2a/Penk/Pla2g5/Ptger4/Ptgs2/Pycard/Rasgrp1/Riok3/Ripk2/Tgm2/Tlr2/Tlr3/Tlr6/Tnf/Tnfsf18/Tnip1/Trem3/Trim6/Trpv4/Tslp/	45
GO:0002688	regulation of leukocyte chemotaxis	27/1242	128/18303	1.03E-07	5.62E-06	3.84E-06	Ccl2/Ccl3/Ccl5/Ccr1/Cmklr1/Csf1/Cx3cl1/Cxcl10/Cxcl12/Dapk2/Ripor2/Vegfd/Il1b/Il23a/Lbp/Lgals9/Nod2/Pgf/Pla2g7/Rarres2/Slit2/Thbs1/Thbs4/Tnfsf18/Trpv4/W	27
GO:0050867	positive regulation of cell activation	53/1242	365/18303	1.20E-07	6.47E-06	4.43E-06	Ada/Blm/Card11/Ccl2/Ccl5/Cd274/Cd40/Cd83/Clcf1/Coro1a/Dock8/Dusp10/Exosc6/F11r/Fgr/Flt3lg/Gata2/Ikzf1/Il15/Il15ra/Il1a/Il1b/Il23a/Il33/Itgal/Lbp/Lgals9/Lilrb4/Mef2c/Nlrp3/Nod2/Pdgfb/Peli1/Plek/Prdm1/Pycard/Myb/Rasal3/Rasgrp1/Ripk2/Selp/Sirpa/Sox12/Syk/Thbs1/Tlr6/Tnf/Tox/Tslp/Vcam1/Vsir/Wnt5a/Xcl1	53
GO:0071260	cellular response to mechanical stimulus	27/1242	129/18303	1.22E-07	6.53E-06	4.47E-06	Ankrd1/Aqp1/Bmp2/Bmp4/Cd40/Col1a1/Col2a1/Cyp11b2/Egr1/F11r/Ripor2/Gclc/Il13ra2/Il1b/Irf1/Itga2/Itgb3/Map3k14/Nfkb1/Pde2a/Pdgfb/Piezo2/Ptger4/Ptgs2/Scx	27
GO:0046394	carboxylic acid biosynthetic process	47/1242	307/18303	1.27E-07	6.69E-06	4.58E-06	Abcd1/Acsl3/Acss1/Alox15/Ass1/Bdkrb1/Carns1/Chst14/Cth/Cyp27a1/Cyp7b1/Elovl4/Fa2h/Fabp5/Fasn/Gamt/Gch1/Ggt1/Gstp1/Hacd4/Has2/Hoga1/Hyal1/Il1b/Insig1/Lipg/Liph/Mgll/Mgst3/Mthfd2l/Nfkb1/Olah/Pdgfb/Pla2g5/Plod2/Prkab2/Ptger4/Ptges/Ptgs2/Pycr1/Rbp1/Sardh/Scd/Star/Stard4/Syk/Thnsl2	47
GO:0010712	regulation of collagen metabolic process	17/1242	57/18303	1.28E-07	6.69E-06	4.58E-06	Bmp4/Ccl2/Creb3l1/Cyp2j4/Emilin1/Errfi1/Itga2/Larp6/Mkx/Pdgfb/Ppard/Myb/Rgcc/Scx/Serpinf2/Vim/Vsir	17
GO:1901343	negative regulation of vasculature development	28/1242	138/18303	1.43E-07	7.45E-06	5.09E-06	Angpt4/Bmp4/Bmp7/Ccl2/Creb3l1/Cxcl10/Dcn/Emilin1/Fbln5/Fgf9/Flt1/Foxc1/Gpr4/Klf2/Angptl7/Lif/Meox2/Pdgfb/Plk2/Ptger4/Rgcc/Sema4a/Sema6a/Stat1/Thbs1/Thbs2/Thbs4/Tnf	28
GO:0016053	organic acid biosynthetic process	47/1242	309/18303	1.55E-07	7.98E-06	5.46E-06	Abcd1/Acsl3/Acss1/Alox15/Ass1/Bdkrb1/Carns1/Chst14/Cth/Cyp27a1/Cyp7b1/Elovl4/Fa2h/Fabp5/Fasn/Gamt/Gch1/Ggt1/Gstp1/Hacd4/Has2/Hoga1/Hyal1/Il1b/Insig1/Lipg/Liph/Mgll/Mgst3/Mthfd2l/Nfkb1/Olah/Pdgfb/Pla2g5/Plod2/Prkab2/Ptger4/Ptges/Ptgs2/Pycr1/Rbp1/Sardh/Scd/Star/Stard4/Syk/Thnsl2	47
GO:0000302	response to reactive oxygen species	46/1242	301/18303	1.82E-07	9.29E-06	6.35E-06	Ada/Akr1b1/Aqp1/Areg/Bmp4/Casp12/Cfh/Cflar/Col1a1/Cyp1b1/Ern1/Ets1/F3/Fbln5/Fosl1/Gch1/Gpr37/Gpr37l1/Gstp1/Hk3/Hyal1/Il18rap/Il1a/Il1b/Il1r1/Jun/Klf2/Klf6/Mmp9/Net1/Ngf/Nox1/Nptxr/Nqo1/Olr1/Ppargc1a/Ppif/Pycr1/Myb/Sesn3/Sirpa/Star/Stat1/Thbs1/Tnf/Tnfaip	46

ID	Description	GeneRatio	BgRatio	pvalue	p.adjust	qvalue	geneID	Count
GO:0045860	positive regulation of protein kinase activity	65/1242	493/18303	1.87E-07	9.47E-06	6.48E-06	Acsl1/Adcy8/Adora1/Adra2a/Agtr1a/Als2/Angpt4/Areg/Bmp2/Bmp4/Ccl5/Ccnd1/Cd40/Cemip/Cks2/Csf1/Cspg4/Dusp19/Efna1/Egr1/Epha4/Ereg/Ern1/Flt1/Fzd4/Fzd5/Fzd8/Gadd45g/Gdf15/Gprc5c/Gprc5d/Hmga2/Htr2a/Igfbp6/Il1b/Il1rn/Il23a/Itgb3/Kitlg/Lgals9/Lrp8/Map3k14/Map3k9/Mdfi/Mmd/Mst1r/Nek10/Ngf/Nod2/Nrg1/Pdgfb/Pik3r5/Inava/Rasgrp1/Rgcc/Ripk2/Syk/Thbs1/Tlr6/Tnf/Tnfsf15/Tnik/Trpv4/Wnt5a/Zeb2	65
GO:0010714	positive regulation of collagen metabolic process	13/1242	35/18303	2.19E-07	1.10E-05	7.50E-06	Bmp4/Ccl2/Creb3l1/Itga2/Larp6/Mkx/Pdgfb/Myb/Rgcc/Scx/Serpinf2/Vim/Vsir	13
GO:0051092	positive regulation of NF-kappaB transcription factor activity	29/1242	149/18303	2.21E-07	1.10E-05	7.50E-06	Card11/Card14/Cd40/Cflar/Cth/Cx3cl1/Flot1/Hspa1b/Il18rap/Il1b/Irak2/Lgals9/Nlrc4/Nlrp3/Nod2/Pycard/Ripk2/Ror1/Slco3a1/Tlr2/Tlr3/Tlr6/Tnf/Tnfsf18/Traf1/Traf5/Trim6/Trim68/Wnt5a	29
GO:0062197	cellular response to chemical stress	57/1242	413/18303	2.34E-07	1.15E-05	7.87E-06	Abcb1a/Abcd1/Akr1b1/Aldh3b1/Aqp1/Bdkrb2/Bmp4/Casp12/Ccl5/Cfh/Cflar/Cpeb2/Cyp1b1/Egr1/Ern1/Errfi1/Ets1/F3/Fbln5/Gch1/Ggt1/Gjb2/Gpr37/Gpr37l1/Hk3/Il18rap/Jun/Klf2/Klf6/Lrrc8c/Lrrc8d/Mgat3/Mmp3/Mmp9/Mylk/Nefh/Net1/Nox1/Nqo1/Penk/Pla2r1/Ppargc1a/Ppif/Ptgs2/Pycr1/Myb/Relb/Sirpa/Slc1a1/Slc6a12/Slc7a11/Thbs1/Tlr6/Tnf/Tnfaip3/Trpv4/Txnrd1	57
GO:0071214	cellular response to abiotic stimulus	55/1242	393/18303	2.40E-07	1.16E-05	7.96E-06	Abcb1a/Akr1b1/Ankrd1/Aqp1/Bdkrb2/Blm/Bmf/Bmp2/Bmp4/Cd40/Ciita/Col1a1/Col2a1/Crip1/Cyp11b2/Egr1/Ercc1/Errfi1/F11r/Ripor2/Gclc/Gpr68/Gpr88/Hmga2/Hvcn1/Hyal1/Il13ra2/Il1b/Irf1/Itga2/Itgb3/Lrrc8c/Lrrc8d/Map3k14/Mylk/Nampt/Net1/Nfkb1/Nox1/Opn3/Palm/Pde2a/Pdgfb/Piezo2/Ppp1r15a/Ptger4/Ptgs2/Relb/Scx/Sfrp1/Slc6a12/Snai2/Tlr3/Tlr5/Trpv4	55
GO:0104004	cellular response to environmental stimulus	55/1242	393/18303	2.40E-07	1.16E-05	7.96E-06	Abcb1a/Akr1b1/Ankrd1/Aqp1/Bdkrb2/Blm/Bmf/Bmp2/Bmp4/Cd40/Ciita/Col1a1/Col2a1/Crip1/Cyp11b2/Egr1/Ercc1/Errfi1/F11r/Ripor2/Gclc/Gpr68/Gpr88/Hmga2/Hvcn1/Hyal1/Il13ra2/Il1b/Irf1/Itga2/Itgb3/Lrrc8c/Lrrc8d/Map3k14/Mylk/Nampt/Net1/Nfkb1/Nox1/Opn3/Palm/Pde2a/Pdgfb/Piezo2/Ppp1r15a/Ptger4/Ptgs2/Relb/Scx/Sfrp1/Slc6a12/Snai2/Tlr3/Tlr5/Trpv4	55
GO:0007159	leukocyte cell-cell adhesion	51/1242	353/18303	2.43E-07	1.17E-05	7.99E-06	Ada/Adora2a/Ass1/Blm/Bmp4/Bmp7/Card11/Ccl2/Ccl5/Cd274/Cd44/Cd83/Coro1a/Cxcl12/Dock8/Dusp10/Ets1/F11r/Ripor2/Gcnt1/Has2/Hfe/Ikzf1/Il15/Il1a/Il1b/Il23a/Irf1/Itga5/Itgal/Lgals3/Lgals9/Lilrb4/Nlrp3/Olr1/Peli1/Pycard/Myb/Rasal3/Rasgrp1/Ripk2/Selp/Sirpa/Sox12/Syk/Tnf/Tnfsf18/Tnip1/Vcam1/Vsir/Xcl1	51
GO:0048247	lymphocyte chemotaxis	16/1242	53/18303	2.48E-07	1.18E-05	8.08E-06	Ccl2/Ccl20/Ccl22/Ccl3/Ccl5/Ccl9/Ch25h/Cx3cl1/Cxcl10/Cxcl11/Cxcl12/Cxcl16/Cyp7b1/LOC290595/Wnt5a/X	16
GO:0043583	ear development	42/1242	266/18303	2.50E-07	1.18E-05	8.08E-06	Adgrv1/Bdnf/Bmp2/Bmp4/Bmp5/Bmper/Cebpd/Clic5/Col11a1/Col2a1/Cthrc1/Dll1/Epha4/Ephb2/Ripor2/Fat4/Fgf9/Fgfr2/Frzb/Fzd2/Gas1/Gata2/Gbx2/Gjb2/Hey2/Insig1/Kcnk2/Kcnk3/Lgr5/Mafb/Mcoln3/Mycl/Myo15a/Myo7a/Nkx3-	42
GO:0045123	cellular extravasation	19/1242	73/18303	2.58E-07	1.21E-05	8.28E-06	Ccl2/Ccl5/Cd99l2/Cx3cl1/Cxcl12/F11r/Ripor2/Gcnt1/Il1a/Il1r1/Itgal/Jaml/Plcb1/Ptger4/Selp/Sirpa/Tnf/Trem3/	19
GO:0048839	inner ear development	39/1242	239/18303	2.80E-07	1.30E-05	8.89E-06	Adgrv1/Bdnf/Bmp2/Bmp4/Bmper/Cebpd/Clic5/Col11a1/Col2a1/Cthrc1/Dll1/Epha4/Ephb2/Ripor2/Fat4/Fgf9/Fgfr2/Frzb/Fzd2/Gata2/Gbx2/Gjb2/Hey2/Insig1/Kcnk2/Kcnk3/Lgr5/Mafb/Mcoln3/Mycl/Myo15a/Myo7a/Pax8/Rbpj/Ror1/Slc4a7/Tshr/Whrn/Wnt5a	39
GO:0007204	positive regulation of cytosolic calcium ion concentration	51/1242	356/18303	3.18E-07	1.46E-05	1.00E-05	Adcy8/Adcyap1r1/Agtr1a/Ank2/Bdkrb1/Bdkrb2/Bmp4/C1qtnf1/Cacna1g/Cacnb2/Casq1/Ccl3/Ccr1/Cemip/Cmklr1/Coro1a/Cx3cl1/Cxcl1/Cxcl10/Cxcl11/Cxcl2/Cxcl3/Cxcr4/Drd1/Ednrb/Fzd2/Gata2/Gna13/Gng3/Gpr4/Grin2a/Htr2a/Il1b/Itgb3/Mcoln2/Mcoln3/Ngf/P2ry2/P2ry6/Ptger1/Ptger4/Ptgir/Pth1r/RGD1560455/Rxfp3/Ryr2/Ryr3/Tgm2/Trpv3/Trpv4/Xcl1	51
GO:0032612	interleukin-1 production	24/1242	111/18303	3.22E-07	1.47E-05	1.01E-05	Ccl20/Ccl3/Cx3cl1/Egr1/Errfi1/Fzd5/Gbp5/Gstp1/Il1b/Lgals9/Mr1/Nlrc4/Nlrp3/Nod2/Ptger4/Pycard/Inava/Ripk2/Sirpa/Tlr2/Tlr6/Tnf/Tnfaip3/Wnt5a	24
GO:0008202	steroid metabolic process	46/1242	307/18303	3.27E-07	1.48E-05	1.01E-05	Acat2/Agtr1a/Bmp2/Bmp5/Ch25h/Cyp11b2/Cyp1b1/Cyp26b1/Cyp27a1/Cyp27b1/Cyp51/Cyp7b1/Dhcr24/Egr1/Fdft1/Fdps/Fmo5/Hmgcr/Hmgcs1/Hsd17b7/Idi1/Il1a/Il1b/Insig1/Ldlr/Msmo1/Mvd/Nfkb1/Nsdhl/Pcsk9/Pmp22/Ppargc1a/Rdh5/Sc5d/Scd/Snai1/Snai2/Sqle/Star/Stard4/Thrb/Tiparp/Tm7sf2/Pip4p1/Tnf/Ttc39b	46
GO:0072001	renal system development	53/1242	377/18303	3.44E-07	1.55E-05	1.06E-05	Adamts6/Agtr1a/Akr1b1/Aqp1/Ass1/Bdnf/Bmp2/Bmp4/Bmp7/Bmper/Cd44/Cfh/Cflar/Col4a1/Cxcr4/Cyp26b1/Dact2/Dcn/Dll1/Egr1/Epha4/Fat4/Fgfr2/Flcn/Foxc1/Gcnt1/Gpr4/Has2/Hnf1b/Hpgd/Irx3/Itgb3/Lgr5/Lif/Mef2c/Mmp9/Npnt/Pax8/Pcsk5/Pcsk9/Pdgfb/Pgf/Plxnd1/Prdm1/Ptpro/Sfrp1/Slit2/Stat1/Thra/Tiparp/Wnk4/Wnt2b/Wnt	53

ID	Description	GeneRatio	BgRatio	pvalue	p.adjust	qvalue	geneID	Count
GO:0014911	positive regulation of smooth muscle cell migration	18/1242	68/18303	4.04E-07	1.81E-05	1.23E-05	Ccl5/Cyp1b1/Dock4/Egr1/F3/Fgf9/Has2/Igfbp5/Itga2/Itgb3/Mmp1/P2ry2/P2ry6/Pcsk5/Pdgfb/Plau/Ptger4/Sema6d	18
GO:0051090	regulation of DNA-binding transcription factor activity	57/1242	420/18303	4.11E-07	1.81E-05	1.23E-05	Adcy8/Anxa3/Bdnf/Card11/Card14/Cd40/Cflar/Cmklr1/Csf3/Cth/Cx3cl1/Cyp1b1/Flot1/Fosl1/Fzd2/Fzd4/Hand2/Hipk2/Hmga2/Hr/Hspa1b/Il18rap/Il1b/Irak2/Acod1/Jun/Lgals9/Lrp8/Nfkbia/Nfkbib/Ngf/Nlrc4/Nlrc5/Nlrp3/Nod2/Peli1/Plpp3/Ppargc1a/Pycard/Rgcc/Ripk2/Ror1/Slco3a1/Syk/Thra/Tlr2/Tlr3/Tlr6/Tnf/Tnfaip3/Tnfsf18/Traf1/Traf5/Trim6/Trim68/Wnt5a/Xcl1	57
GO:0061448	connective tissue development	44/1242	290/18303	4.12E-07	1.81E-05	1.23E-05	Aacs/Arrdc3/Barx2/Bmp2/Bmp3/Bmp4/Bmp7/Cd44/Cflar/Col11a1/Col1a1/Col2a1/Col5a1/Crip1/Csf1/Egr1/Fgf9/Frzb/Gdf6/Gpr4/Hand2/Hmga2/Hyal1/Itgb3/Mef2c/Mgp/Mkx/Mmp13/Nkx3-2/Pdgfb/Ppard/Ppargc1a/Pth1r/Snorc/Scx/Slc25a25/Snai1/Snai2/Thbs1/Thra/Thrb/Trpv4/Wnt2b/Wnt5a	44
GO:0042362	fat-soluble vitamin biosynthetic process	8/1242	13/18303	4.16E-07	1.81E-05	1.23E-05	Cyp27a1/Cyp27b1/Il1b/Nfkb1/Pltp/Snai1/Snai2/Tnf	8
GO:1903034	regulation of response to wounding	33/1242	188/18303	4.17E-07	1.81E-05	1.23E-05	Adra2a/C1qtnf1/Cadm4/Cask/Ccl2/Cldn3/Cxcr4/Dusp10/Epha4/Ephb2/Eppk1/F11r/Hmgcr/Hpse/Mylk/Nrg1/Pdgfb/Plau/Plek/Plpp3/Ptger4/Rgma/Scarf1/Selp/Serpinf2/Serping1/Siglec10/Syk/Thbd/Thbs1/Tnf/Wfdc1/Xylt1	33
GO:0031589	cell-substrate adhesion	51/1242	360/18303	4.50E-07	1.93E-05	1.32E-05	Alox15/Bcam/Bves/Cask/Ccdc80/Col13a1/Col1a1/Col3a1/Col5a3/Coro1a/Coro2b/Csf1/Cspg5/Edil3/Efna1/Emilin1/Enpp2/Ephb3/Fbln1/Frem1/Fzd4/Has2/Hpse/Itga11/Itga2/Itga5/Itgal/Itgb3/Itgbl1/Lamb3/Limch1/Lpxn/Meltf/Mmp12/Msln/Ndnf/Net1/Nid2/Npnt/Ntn4/Pcsk5/Pik3cb/Plau/Plet1/Ppard/Sirpa/Smoc1/Sned1/Thbs1/Tnn/Vca	51
GO:0006066	alcohol metabolic process	49/1242	341/18303	4.92E-07	2.10E-05	1.44E-05	Acat2/Adcyap1r1/Akr1b1/Aldh3b1/Bmp2/Bmp5/Ch25h/Chka/Cyp11b2/Cyp1b1/Cyp27a1/Cyp27b1/Cyp51/Cyp7b1/Dhcr24/Dhrs3/Fdft1/Fdps/Fmo5/Gch1/Hmgcr/Hmgcs1/Hsd17b7/Idi1/Il1b/Insig1/Ldlr/Msmo1/Mtmr7/Mvd/Nfkb1/Nsdhl/P2ry6/Pcsk9/Plcb1/Plek/Plpp3/Pmp22/Pth1r/Rbp1/Sc5d/Slc5a3/Sqle/Star/Thrb/Tm7sf2/Pip4p1/Tn	49
GO:0043406	positive regulation of MAP kinase activity	38/1242	236/18303	5.59E-07	2.37E-05	1.62E-05	Adora1/Adra2a/Bmp2/Bmp4/Cd40/Cspg4/Dusp19/Epha4/Ern1/Flt1/Fzd4/Fzd5/Fzd8/Gadd45g/Gdf15/Htr2a/Igfbp6/Il1b/Il1rn/Kitlg/Map3k9/Mdfi/Mst1r/Nek10/Nod2/Nrg1/Pdgfb/Pik3r5/Inava/Rasgrp1/Ripk2/Syk/Thbs1/Tlr6/Tnf/Tnik/Wnt5a/Zeb2	38
GO:0050730	regulation of peptidyl-tyrosine phosphorylation	42/1242	274/18303	5.68E-07	2.39E-05	1.63E-05	Adora1/Adra2a/Angpt4/Areg/Cadm4/Ccl5/Cd40/Cd44/Clcf1/Csf2/Csf3/Cspg4/Efna1/Enpp2/Epha4/Ereg/Errfi1/Htr2a/Il11/Il15/Il23a/Il31ra/Irf1/Itga5/Itgb3/Kitlg/Lif/Lrp8/Nod2/Nrg1/Pdgfb/Plpp3/Ptger4/Ripk2/Sfrp1/Socs3/Syk/Thbs4/Tnf/Tnfsf18/Trpv4/Tslp	42
GO:0032967	positive regulation of collagen biosynthetic process	12/1242	32/18303	5.71E-07	2.39E-05	1.63E-05	Bmp4/Ccl2/Creb3l1/Itga2/Larp6/Mkx/Pdgfb/Myb/Rgcc/Scx/Serpinf2/Vim	12
GO:0050878	regulation of body fluid levels	55/1242	404/18303	5.92E-07	2.45E-05	1.68E-05	Abcb1a/Abcb1b/Ada/Adora1/Adora2a/Adra2a/Akr1b1/Anxa8/Aqp1/Atp7b/C1qtnf1/Ccnd1/Cfh/Coro2b/Cx3cl1/Cyp26b1/Cyp27b1/Ednrb/Ephb2/F11r/F13a1/F3/Fa2h/Fbln1/Gja5/Gna13/Has2/Hk2/Hpse/Itgb3/Kcnn4/Mmp13/Mmrn1/Npr3/P2ry2/Pdgfb/Pik3cb/Plau/Plek/Prl8a9/Procr/Ptger4/Ptpro/Jhy/Scd/Selp/Serpinf2/Serping1/Slc7a11/Syk/Tfpi2/Thbd/Thbs1/Trpv4/Wnk4	55
GO:0030336	negative regulation of cell migration	44/1242	294/18303	6.06E-07	2.50E-05	1.71E-05	Ada/Adora1/Angpt4/Arap3/Arhgdib/Ccl2/Cd200r1/Chrd/Cldn3/Col3a1/Cx3cl1/Cxcl12/Cyp1b1/Dcn/Drd1/Dusp10/Emilin1/Eppk1/Ripor2/Flcn/Fuz/Gstp1/Igfbp5/Il1rn/Il33/Limch1/Mef2c/Meox2/Nrg1/Pdgfb/Plcb1/Ppard/Ppargc1a/Ptger4/Ptprt/Rbpj/Rgcc/Sema6d/Sfrp1/Slit2/Srgap1/Thbs1/Tnf/Tnn	44
GO:0018108	peptidyl-tyrosine phosphorylation	53/1242	384/18303	6.16E-07	2.52E-05	1.72E-05	Adora1/Adra2a/Agtr1a/Angpt4/Areg/Cadm4/Ccl5/Cd40/Cd44/Clcf1/Csf2/Csf3/Cspg4/Efna1/Enpp2/Epha4/Ephb2/Ephb3/Ereg/Errfi1/Fgfr2/Fgr/Flt1/Flt4/Hipk2/Htr2a/Il11/Il15/Il23a/Il31ra/Irf1/Itga5/Itgb3/Kitlg/Lif/Lmtk2/Lrp8/Mst1r/Nod2/Nrg1/Pdgfb/Plpp3/Ptger4/Ripk2/Ror1/Sfrp1/Socs3/Syk/Thbs4/Tnf/Tnfsf18/Trpv4/Tslp	53
GO:0006695	cholesterol biosynthetic process	15/1242	50/18303	6.43E-07	2.59E-05	1.77E-05	Acat2/Cyp51/Dhcr24/Fdft1/Fdps/Hmgcr/Hmgcs1/Hsd17b7/Idi1/Insig1/Msmo1/Mvd/Nsdhl/Sc5d/Tm7sf2	15
GO:1902653	secondary alcohol biosynthetic process	15/1242	50/18303	6.43E-07	2.59E-05	1.77E-05	Acat2/Cyp51/Dhcr24/Fdft1/Fdps/Hmgcr/Hmgcs1/Hsd17b7/Idi1/Insig1/Msmo1/Mvd/Nsdhl/Sc5d/Tm7sf2	15
GO:0050679	positive regulation of epithelial cell proliferation	36/1242	219/18303	6.63E-07	2.65E-05	1.82E-05	Agtr1a/Bmp2/Bmp4/Bmp5/Ccl2/Ccl5/Ccnd1/Cdh3/Cflar/Cxcl12/Cyp7b1/Eppk1/Esrp2/F3/Fgf9/Fgfr2/Flt4/Gas1/Gata2/Has2/Hmga2/Hyal1/Itgb3/Jaml/Jun/Mmp12/Nod2/Pdgfb/Pgf/Sema5a/Sfrp1/Thbs4/Tnf/Tnfaip3/Twist2/	36
GO:0032611	interleukin-1 beta production	21/1242	92/18303	6.74E-07	2.68E-05	1.83E-05	Ccl3/Egr1/Errfi1/Fzd5/Gbp5/Gstp1/Il1b/Lgals9/Mr1/Nlrc4/Nlrp3/Nod2/Pycard/Inava/Ripk2/Sirpa/Tlr2/Tlr6/Tnf/Tnfaip3/Wnt5a	21

ID	Description	GeneRatio	BgRatio	pvalue	p.adjust	qvalue	geneID	Count
GO:0001890	placenta development	33/1242	192/18303	6.85E-07	2.70E-05	1.85E-05	Abcb1a/Abcb1b/Ada/Bmp5/Bmp7/Csf2/Cyp27b1/Dcn/Dlx3/Fgfr2/Fosl1/Fzd5/Gata2/Gjb2/Hey2/Junb/Lif/Mdfi/Nsdhl/Pdgfb/Peg10/Ppard/Prdm1/Ptgs2/Rbm15/Rbpj/Snai1/Socs3/St14/Tfeb/Txnrd1/Vcam1/Vim	33
GO:0030278	regulation of ossification	38/1242	238/18303	6.95E-07	2.72E-05	1.86E-05	Adgrv1/Areg/Bmp2/Bmp4/Bmp7/Ccl3/Ccr1/Cebpd/Chrd/Creb3l1/Csf1/Cthrc1/Cyp27b1/Dhrs3/Fam20c/Gdpd2/Gfra4/Gli1/Hand2/Igfbp5/Isg15/Mef2c/Mgp/Noct/Npnt/P2ry2/Ptger4/Rbpj/Rorb/Rsad2/Sfrp1/Smoc1/Snai2/Thrb/Tnf/Tnn/Twist2/Wnt5a	38
GO:0001667	ameboidal-type cell migration	58/1242	438/18303	7.40E-07	2.88E-05	1.97E-05	Abcc9/Akap12/Angpt4/Anxa3/Aqp1/Arhgdib/Bmp4/Bmp7/Bmper/Cd40/Cxcl12/Cxcr4/Cyp1b1/Dcn/Dusp10/Ednrb/Efna1/Enpp2/Eppk1/Ets1/Flt4/Gata2/Gbx2/Hand2/Has2/Hdac9/Hyal1/Itga2/Itgb3/Jun/Kitlg/Lpxn/Mef2c/Meox2/Mmp1/Mmp12/Mmp9/Pdgfb/Plk2/Plpp3/Plxnd1/Pmp22/Ppard/Ptgs2/Ptp4a3/Rgcc/Rhoj/Sema3f/Sema4a/Sema4b/Sema5a/Sema6a/Sema6d/Slit2/Thbs1/Tnf/Wnt	58
GO:0071675	regulation of mononuclear cell migration	16/1242	57/18303	7.47E-07	2.89E-05	1.98E-05	Ccl2/Ccl5/Ccr1/Cmklr1/Csf1/Cx3cl1/Cxcl10/Cxcl12/Lgals3/Pla2g7/Plcb1/Rarres2/Slit2/Thbs1/Tnfsf18/Trpv4	16
GO:0018212	peptidyl-tyrosine modification	53/1242	387/18303	7.86E-07	3.02E-05	2.07E-05	Adora1/Adra2a/Agtr1a/Angpt4/Areg/Cadm4/Ccl5/Cd40/Cd44/Clcf1/Csf2/Csf3/Cspg4/Efna1/Enpp2/Epha4/Ephb2/Ephb3/Ereg/Errfi1/Fgfr2/Fgr/Flt1/Flt4/Hipk2/Htr2a/Il11/Il15/Il23a/Il31ra/Irf1/Itga5/Itgb3/Kitlg/Lif/Lmtk2/Lrp8/Mst1r/Nod2/Nrg1/Pdgfb/Plpp3/Ptger4/Ripk2/Ror1/Sfrp1/Socs3/Syk/Thbs4/Tnf/Tnfsf18/Trpv4/Tslp	53
GO:0032613	interleukin-10 production	17/1242	64/18303	8.02E-07	3.06E-05	2.09E-05	Bcl3/Cd274/Cd83/Dll1/Il23a/Isg15/Lgals9/Nod2/Ptger4/Pycard/Inava/Syk/Tlr2/Trib2/Tslp/Vsir/Xcl1	17
GO:0032823	regulation of natural killer cell differentiation	9/1242	18/18303	8.23E-07	3.12E-05	2.13E-05	Flt3lg/Il15/Il15ra/Lgals9/Pglyrp1/Pglyrp4/Prdm1/Rasgrp1/Tox	9
GO:0060562	epithelial tube morphogenesis	53/1242	388/18303	8.52E-07	3.21E-05	2.19E-05	Agtr1a/Areg/Bmp2/Bmp4/Bmp5/Bmp7/Cd44/Cobl/Col4a1/Csf1/Csmd1/Cthrc1/Cxcl10/Cxcl12/Cxcr4/Dll1/Epha4/Esrp2/Fat4/Fgfr2/Flt1/Fuz/Fzd2/Gbx2/Gna13/Hand2/Hmga2/Hnf1b/Irx3/Itgax/Lgr5/Mef2c/Mgp/Npnt/Pax8/Pgf/Plxnd1/Prickle1/Rbm15/Rbpj/Rgma/Ryr2/Sema5a/Sfrp1/Slit2/St14/Tbx3/Tgif1/Tnf/Wnk4/Wnt2b/Wnt5a/Z	53
GO:0002696	positive regulation of leukocyte activation	49/1242	348/18303	9.03E-07	3.38E-05	2.31E-05	Ada/Blm/Card11/Ccl2/Ccl5/Cd274/Cd40/Cd83/Clcf1/Coro1a/Dock8/Dusp10/Exosc6/Fgr/Flt3lg/Gata2/Ikzf1/Il15/Il15ra/Il1a/Il1b/Il23a/Il33/Itgal/Lbp/Lgals9/Lilrb4/Mef2c/Nlrp3/Nod2/Peli1/Prdm1/Pycard/Myb/Rasal3/Rasgrp1/Ripk2/Sirpa/Sox12/Syk/Thbs1/Tlr6/Tnf/Tox/Tslp/Vcam1/Vsir/Wnt5a/Xcl1	49
GO:0030217	T cell differentiation	42/1242	279/18303	9.28E-07	3.45E-05	2.36E-05	Ada/Bcl3/Blm/Bmp4/Card11/Cd44/Cd83/Clec4e/Coro1a/Cyp26b1/Dusp10/Egr1/Foxn1/Fzd5/Fzd8/Ikzf1/Il15/Il1a/Il1b/Il23a/Irf1/Lgals9/Lilrb4/Mafb/Mr1/Nlrp3/Pax1/Prdm1/Ptger4/Myb/Rasgrp1/Relb/Ripk2/Rsad2/Satb1/Sema4a/Sox12/Syk/Tnfsf18/Tnfsf8/Tox/Vsir	42
GO:0046631	alpha-beta T cell activation	30/1242	168/18303	9.50E-07	3.50E-05	2.39E-05	Ada/Adora2a/Bcl3/Blm/Cd274/Cd44/Cd83/Hfe/Ikzf1/Il15/Il23a/Irf1/Lgals9/Nlrp3/Pax1/Prdm1/Ptger4/Myb/Rasal3/Relb/Ripk2/Rsad2/Satb1/Sema4a/Syk/Tnfsf18/Tnfsf8/Tox/Vsir/Xcl1	30
GO:0045444	fat cell differentiation	40/1242	260/18303	9.54E-07	3.50E-05	2.39E-05	Aacs/Arl4a/Bmp2/Bmp7/C1qtnf3/Ccdc85b/Ccnd1/Cebpd/Cmklr1/Dusp10/Fabp4/Flcn/Frzb/Gata2/Gdf6/Hmga2/Htr2a/Insig1/Lamb3/Lrrc8c/Mafb/Medag/Noct/Plcb1/Pnpla3/Ppard/Ppargc1a/Ptgs2/Rarres2/Scd/Sfrp1/Sh2b2/Snai2/Tnf/Trib2/Trpv4/Vim/Wfdc21/Wnt5a/Zbtb7c	40
GO:0043506	regulation of JUN kinase activity	21/1242	94/18303	9.83E-07	3.59E-05	2.45E-05	Dusp10/Dusp19/Epha4/Ern1/Fzd4/Fzd5/Fzd8/Gstp1/Il1b/Il1rn/Map3k9/Mdfi/Ripk2/Serpinb3/Sfrp1/Syk/Tlr6/Tnf/Tnik/Wnt5a/Zeb2	21
GO:0030098	lymphocyte differentiation	53/1242	390/18303	9.99E-07	3.62E-05	2.48E-05	Ada/Bcl3/Blm/Bmp4/Card11/Cd44/Cd83/Clcf1/Clec4e/Coro1a/Cyp26b1/Dll1/Dock10/Dusp10/Egr1/Flt3lg/Fnip1/Foxn1/Fzd5/Fzd8/Ikzf1/Il15/Il15ra/Il1a/Il1b/Il23a/Irf1/Lgals9/Lilrb4/Mafb/Mr1/Nlrp3/Pax1/Pglyrp1/Pglyrp4/Prdm1/Ptger4/Myb/Rasgrp1/Rbpj/Relb/Ripk2/Rsad2/Satb1/Sema4a/Sfrp1/Sox12/Syk/Tnfsf18/Tnfsf8/Tox/Tshr	53
GO:2000146	negative regulation of cell motility	45/1242	310/18303	1.08E-06	3.88E-05	2.65E-05	Ada/Adora1/Angpt4/Arap3/Arhgdib/Ccl2/Cd200r1/Chrd/Cldn3/Col3a1/Cx3cl1/Cxcl12/Cyp1b1/Dcn/Drd1/Dusp10/Emilin1/Eppk1/Ripor2/Fbln1/Flcn/Fuz/Gstp1/Igfbp5/Il1rn/Il33/Limch1/Mef2c/Meox2/Nrg1/Pdgfb/Plcb1/Ppard/Ppargc1a/Ptger4/Ptprt/Rbpj/Rgcc/Sema6d/Sfrp1/Slit2/Srgap1/Thbs1/Tnf/Tnn	45
GO:0003018	vascular process in circulatory system	37/1242	234/18303	1.23E-06	4.42E-05	3.02E-05	Abcb1a/Adora1/Adora2a/Adra2a/Agtr1a/Akap12/Bdkrb2/Cacna1g/Cx3cl1/Dock4/Drd1/Dusp5/Ednrb/Fabp5/Flt1/Foxc1/Gch1/Gclc/Gja5/Gpr4/Hmgcr/Hspa1b/Htr2a/Htr7/Mgll/Npy1r/Olr1/P2ry2/Pde2a/Ppard/Ptgs2/Ptp4a3/Ramp2/Serpinf2/Slit2/Tnf/Trpv4	37
GO:1902107	positive regulation of leukocyte differentiation	31/1242	179/18303	1.25E-06	4.44E-05	3.04E-05	Ada/Ccl3/Ccl5/Ccr1/Cd83/Csf1/Csf2/Dusp10/Flt3lg/Gpr68/Ikzf1/Il15/Il15ra/Il1a/Il1b/Il23a/Jun/Kitlg/Lgals9/Lif/Lilrb4/Nlrp3/Prdm1/Myb/Rasgrp1/Ripk2/Sox12/Syk/T	31

ID	Description	GeneRatio	BgRatio	pvalue	p.adjust	qvalue	geneID	Count
GO:1903037	regulation of leukocyte cell-cell adhesion	45/1242	312/18303	1.29E-06	4.54E-05	3.10E-05	Ada/Adora2a/Ass1/Blm/Bmp4/Card11/Ccl2/Ccl5/Cd274/Cd44/Cd83/Coro1a/Cxcl12/Dock8/Dusp10/Ets1/Ripor2/Has2/Hfe/Ikzf1/Il15/Il1a/Il1b/Il23a/Irf1/Itgal/Lgals3/Lgals9/Lilrb4/Nlrp3/Peli1/Pycard/Myb/Rasal3/Rasgrp1/Ripk2/Selp/Sirpa/Sox12/Syk/Tnf/Tnfsf18/Vcam1/Vsir/	45
GO:0071902	positive regulation of protein serine/threonine kinase activity	46/1242	322/18303	1.29E-06	4.54E-05	3.10E-05	Acsl1/Adora1/Adra2a/Als2/Bmp2/Bmp4/Ccnd1/Cd40/Cemip/Cks2/Cspg4/Dusp19/Epha4/Ern1/Flt1/Fzd4/Fzd5/Fzd8/Gadd45g/Gdf15/Hmga2/Htr2a/Igfbp6/Il1b/Il1rn/Kitlg/Lgals9/Map3k9/Mdfi/Mst1r/Nek10/Nod2/Nrg1/Pdgfb/Pik3r5/Inava/Rasgrp1/Rgcc/Ripk2/Syk/Thbs1/Tlr6/Tnf/Tnik/Wnt5a/Zeb2	46
GO:0032733	positive regulation of interleukin-10 production	13/1242	40/18303	1.30E-06	4.54E-05	3.10E-05	Bcl3/Cd274/Cd83/Il23a/Lgals9/Nod2/Ptger4/Pycard/Inava/Syk/Tlr2/Tslp/Xcl1	13
GO:0001822	kidney development	50/1242	363/18303	1.35E-06	4.70E-05	3.21E-05	Adamts6/Agtr1a/Akr1b1/Aqp1/Ass1/Bdnf/Bmp2/Bmp4/Bmp7/Bmper/Cd44/Cfh/Cflar/Cxcr4/Cyp26b1/Dact2/Dcn/Dll1/Egr1/Epha4/Fat4/Fgfr2/Flcn/Foxc1/Gcnt1/Gpr4/Has2/Hnf1b/Hpgd/Irx3/Itgb3/Lif/Mef2c/Mmp9/Npnt/Pax8/Pcsk5/Pcsk9/Pdgfb/Pgf/Plxnd1/Prdm1/Ptpro/Sfrp1/Slit2/Thra/Tiparp/Wnk4/Wnt2b/Wnt5a	50
GO:0019932	second-messenger-mediated signaling	60/1242	469/18303	1.52E-06	5.25E-05	3.59E-05	Ada/Adcy8/Adcyap1r1/Adgrd1/Adora2a/Adra2a/Agtr1a/Ank2/Aqp1/Arrdc3/Calcrl/Calml4/Casq1/Ccl20/Ccl3/Ccr1/Cmklr1/Cxcl10/Cxcl11/Cxcr4/Drd1/Ednrb/Gna13/Gpr4/Grin2a/Htr7/Itgal/Lgr5/Mafa/Ncam1/Ndnf/Nos2/Nrg1/P2ry6/Pclo/Pde2a/Pde4b/Pdgfb/Prkar2a/Prkar2b/Ptger1/Ptger4/Ptgir/Pth1r/RGD1564899/Ramp2/Rcan2/Ryr2/Selp/Sgcd/Siglec10/Syk/Tcp11/Thbs1/Tmem100/Tnf/Tshr/Ucn2/Vcam1/Vsnl1	60
GO:0050663	cytokine secretion	18/1242	74/18303	1.56E-06	5.36E-05	3.67E-05	Agtr1a/C1qtnf3/Cadm1/Clec4e/Cx3cl1/Fgr/Hdac9/Il1a/Nlrp3/Nod2/Ptger4/Rgcc/Syk/Tlr2/Tlr5/Tnf/Tnfsf15/Tre	18
GO:0032653	regulation of interleukin-10 production	16/1242	60/18303	1.59E-06	5.40E-05	3.69E-05	Bcl3/Cd274/Cd83/Dll1/Il23a/Lgals9/Nod2/Ptger4/Pycard/Inava/Syk/Tlr2/Trib2/Tslp/Vsir/Xcl1	16
GO:0014074	response to purine-containing compound	36/1242	227/18303	1.59E-06	5.40E-05	3.69E-05	Aacs/Abcc9/Adora2a/Aqp1/Areg/Ass1/Atp7b/Ccl2/Col1a1/Cyp1b1/Cyp27b1/Cyp7b1/Egr1/Fosl1/Gata6/Hcn1/Hmgcs1/Igfbp5/Il1b/Jun/Junb/P2ry2/P2ry6/Pde2a/Penk/Pla2g5/Plat/Ppargc1a/Ppp1r15a/Ptgs2/Rap1b/Ryr2/Serpina3n/Star/Stat1/Thbd	36
GO:0061041	regulation of wound healing	27/1242	146/18303	1.63E-06	5.49E-05	3.76E-05	Adra2a/C1qtnf1/Cadm4/Cask/Ccl2/Cldn3/Cxcr4/Dusp10/Ephb2/Eppk1/F11r/Hmgcr/Hpse/Mylk/Pdgfb/Plau/Plek/Plpp3/Ptger4/Selp/Serpinf2/Serping1/Syk/Thbd/Thbs1/Tnf/Wfdc1	27
GO:0042368	vitamin D biosynthetic process	7/1242	11/18303	1.69E-06	5.66E-05	3.87E-05	Cyp27a1/Cyp27b1/Il1b/Nfkb1/Snai1/Snai2/Tnf	7
GO:0090596	sensory organ morphogenesis	45/1242	316/18303	1.83E-06	6.10E-05	4.17E-05	Aqp1/Bdnf/Bmp4/Bmp7/Cfh/Clic5/Col11a1/Col2a1/Col5a1/Col8a2/Cthrc1/Cyp26b1/Dll1/Ephb2/Ripor2/Fat3/Fgf9/Fgfr2/Flt1/Frzb/Fuz/Fzd2/Fzd5/Gas1/Gata2/Gbx2/Hcn1/Hipk2/Ikzf1/Insig1/Mafb/Myo15a/Myo7a/Nectin1/Nkx3-2/Obsl1/Pax8/Pdgfb/Prdm1/Rorb/Slc4a7/Thrb/Tshr/Whr	45
GO:0043122	regulation of I-kappaB kinase/NF-kappaB signaling	37/1242	238/18303	1.87E-06	6.20E-05	4.24E-05	Card11/Cd40/Cflar/Cth/Cx3cl1/Edar/Gstp1/Il1a/Il1b/Lgals9/Lurap1l/Map3k14/Nod2/Peli1/Plk2/Pycard/Inava/Rel/Riok3/Ripk2/Ror1/Sirpa/Slc20a1/Slc44a2/Stat1/Tgm2/Tifa/Tlr3/Tlr6/Tnf/Tnfaip3/Tnip1/Traf1/Traf5/Trim6/Trim68/Wnt5a	37
GO:2000404	regulation of T cell migration	14/1242	48/18303	2.21E-06	7.29E-05	4.98E-05	Ccl20/Ccl5/Cd200r1/Cd99l2/Cxcl10/Cxcl12/Dock8/Ripor2/Itgb3/LOC290595/Lgals9/Pycard/Wnt5a/Xcl1	14
GO:1902105	regulation of leukocyte differentiation	44/1242	309/18303	2.38E-06	7.80E-05	5.33E-05	Ada/Bmp4/Card11/Ccl3/Ccl5/Ccr1/Cd44/Cd83/Csf1/Csf2/Cyp26b1/Dusp10/Flt3lg/Foxn1/Gata2/Gpr68/Ikzf1/Il15/Il15ra/Il1a/Il1b/Il23a/Irf1/Jun/Kitlg/Lgals9/Lif/Lilrb4/Mafb/Nlrp3/Pglyrp1/Pglyrp4/Prdm1/Myb/Rasgrp1/Rbp1/Ripk2/Sfrp1/Sox12/Syk/Tnf/Tnfsf18/Tox/Vsir	44
GO:0051048	negative regulation of secretion	36/1242	231/18303	2.42E-06	7.88E-05	5.39E-05	Acsl4/Ada/Adora1/Adra2a/Anpep/Cx3cl1/Cyp51/Frmd4a/Hadh/Hdac9/Hmgcr/Htr7/Il11/Il13ra2/Il1b/Il1rn/Il33/Inha/Lgals9/Lif/Npy1r/Nrg1/Pla2r1/Ptger4/Rab11fip1/Rap1b/Rgcc/Rsad2/Sfrp1/Siglec10/Tcp11/Tnf/Tnfrsf1b/Ucn2/Vsnl1/Wnk4	36
GO:0042326	negative regulation of phosphorylation	60/1242	476/18303	2.47E-06	7.99E-05	5.47E-05	Adora2a/Atf3/Bdkrb1/Bdkrb2/Birc3/Bmp4/Bmp7/Cadm4/Drd1/Dusp10/Dusp16/Dusp19/Dusp5/Dusp6/Efna1/Emilin1/Ephb2/Errfi1/Fabp4/Fbln1/Flcn/Gadd45g/Gstp1/Hmgcr/Ier3/Il1b/Irf1/Jun/Itprip/Lif/Mical1/Ndrg2/Pcp4/Pde2a/Pik3ip1/Plpp3/Ppargc1a/Ppif/Ppp1r15a/Prkar2a/Prkar2b/Ptpn5/Ptprb/Ptpro/Ptprt/Pycard/Rgs14/Rnf149/Sema6a/Serpinb3/Sfrp1/Sirpa/Slit2/Smcr8/Socs3/Spry4/Timp3/Tnf/Tnfaip3/Tnip1	60
GO:1903039	positive regulation of leukocyte cell-cell adhesion	35/1242	222/18303	2.54E-06	8.18E-05	5.60E-05	Ada/Blm/Card11/Ccl2/Ccl5/Cd274/Cd44/Cd83/Coro1a/Dock8/Dusp10/Ets1/Has2/Ikzf1/Il15/Il1a/Il1b/Il23a/Itgal/Lgals9/Lilrb4/Nlrp3/Pycard/Myb/Rasal3/Rasgrp1/Ripk2/Selp/Sirpa/Sox12/Syk/Tnf/Vcam1/Vsir/Xcl1	35

ID	Description	GeneRatio	BgRatio	pvalue	p.adjust	qvalue	geneID	Count
GO:0048754	branching morphogenesis of an epithelial tube	31/1242	185/18303	2.57E-06	8.23E-05	5.63E-05	Agtr1a/Areg/Bmp2/Bmp4/Bmp7/Cd44/Col4a1/Csf1/Csmd1/Cxcl12/Cxcr4/Esrp2/Fat4/Fgfr2/Flt1/Gbx2/Gna13/Hmga2/Hnf1b/Mgp/Npnt/Pax8/Pgf/Plxnd1/Rbm15/Sema5a/Slit2/Tbx3/Tnf/Wnt2b/Wnt5a	31
GO:0015718	monocarboxylic acid transport	31/1242	186/18303	2.89E-06	9.17E-05	6.27E-05	Abcd1/Acsl1/Acsl3/Acsl4/Bdkrb2/Cpt1b/Erfe/Fabp3/Fabp4/Fabp5/Il1a/Il1b/Nos2/P2ry2/Pla2g2a/Pla2g5/Pla2r1/Plin2/Ppard/Ptges/Slc16a12/Slc16a14/Slc16a6/Slc27a3/Slc6a12/Slco3a1/Syk/Thbs1/Tnf/Trpc4/Trpv4	31
GO:0032642	regulation of chemokine	18/1242	77/18303	2.89E-06	9.17E-05	6.27E-05	C1qtnf3/Gstp1/Il1a/Il1b/Lbp/Lgals9/Mcoln2/Nod2/P2ry2/Ripk2/Sirpa/Snai2/Syk/Tlr2/Tlr3/Tnf/Trpv4/Tslp	18
GO:0001649	osteoblast differentiation	34/1242	214/18303	2.95E-06	9.30E-05	6.36E-05	Areg/Bmp2/Bmp3/Bmp4/Bmp7/Ccl3/Cebpd/Chrd/Col1a1/Creb3l1/Cthrc1/Fam20c/Fgf9/Gdpd2/Gli1/Hand2/Igfbp5/Junb/Mef2c/Mmp13/Noct/Npnt/Pcp4/Penk/Ptger4/Pth1r/Rorb/Sfrp1/Smoc1/Snai1/Snai2/Tnf/Tnn/Twist2	34
GO:0031663	lipopolysaccharide-mediated signaling pathway	15/1242	56/18303	3.16E-06	9.86E-05	6.74E-05	Ccl2/Ccl3/Ccl5/Cd180/Cx3cl1/Il1b/Irak2/Acod1/Lbp/Nfkbia/Prdm1/Ripk2/Stat1/Tnf/Tnfaip3	15
GO:0032637	interleukin-8 production	19/1242	85/18303	3.17E-06	9.86E-05	6.74E-05	Bcl3/Camp/F3/Hspa1b/Il1b/Lbp/Lgals9/Nod2/Nos2/Ptger4/Pycard/Ssc5d/Syk/Tlr2/Tlr3/Tlr5/Tlr6/Tnf/Wnt5a	19
GO:0090100	positive regulation of transmembrane receptor protein serine/threonine kinase signaling pathway	23/1242	117/18303	3.23E-06	9.98E-05	6.82E-05	Bmp2/Bmp3/Bmp4/Bmp5/Bmp7/Fgf9/Flcn/Furin/Gata6/Gdf15/Gdf6/Hfe/Hipk2/Inha/Inhba/Inhbe/Lgals9/Npnt/Rbpj/Rbpms/Slc2a10/Thbs1/Vsir	23
GO:0042063	gliogenesis	50/1242	374/18303	3.24E-06	9.98E-05	6.82E-05	Adora2a/Areg/Aspa/Bmp2/Bmp4/Ccl2/Ccl3/Clcf1/Csf1/Cspg4/Cspg5/Cx3cl1/Cxcr4/Dll1/Drd1/Dusp10/Egr1/Enpp2/Epha4/Fa2h/Flt1/Gpr37l1/Gstp1/Hmga2/Il1b/Il33/Lama2/Ldlr/Lif/Lrp8/Matn2/Metrn/Mxra8/Myrf/Nfix/Nrg1/Pdgfb/Penk/Plpp3/Pmp22/Prdm8/Myb/Ror1/Sh3tc2/Tenm4/Tlr2/Tnf/Tnfrsf1b/Trpc4/Vim	50
GO:0051147	regulation of muscle cell differentiation	29/1242	169/18303	3.30E-06	0.000101	6.91E-05	Bdnf/Bmp2/Bmp4/Cd53/Cflar/Col14a1/Cth/Cxcl10/Cxcl12/Cyp26b1/Dll1/Ehd2/Ereg/Ripor2/Fdps/Fgf9/Fgfr2/Flot1/Flt3lg/Gdf15/Hdac9/Mef2c/Nrg1/Pdgfb/Pi16/Prdm6/Rbm38/Rbpj/Thra	29
GO:0050715	positive regulation of cytokine secretion	12/1242	37/18303	3.41E-06	0.000104	7.11E-05	Agtr1a/C1qtnf3/Cadm1/Clec4e/Fgr/Il1a/Ptger4/Rgcc/Syk/Tlr2/Tnf/Tnfsf15	12
GO:0032677	regulation of interleukin-8 production	18/1242	78/18303	3.53E-06	0.000107	7.32E-05	Bcl3/Camp/F3/Hspa1b/Il1b/Lbp/Lgals9/Nod2/Ptger4/Pycard/Ssc5d/Syk/Tlr2/Tlr3/Tlr5/Tlr6/Tnf/Wnt5a	18
GO:0050729	positive regulation of inflammatory response	27/1242	152/18303	3.63E-06	0.00011	7.49E-05	Ccl3/Ccl5/Cx3cl1/Ets1/Fabp4/Gbp5/Gpr4/Il1b/Il33/Itga2/Lbp/Ldlr/Nfkbia/Nod2/Pde2a/Ptger4/Ptgs2/Tgm2/Tlr2/Tlr3/Tlr6/Tnf/Tnfsf18/Tnip1/Trpv4/Tslp/Wnt5a	27
GO:0048762	mesenchymal cell differentiation	35/1242	226/18303	3.84E-06	0.000115	7.89E-05	Akna/Bmp2/Bmp4/Bmp7/Col1a1/Ednrb/Efna1/Fgfr2/Foxc1/Frzb/Fuz/Gbx2/Hand2/Has2/Hey2/Hmga2/Il1b/Kitlg/Mef2c/Plaur/Rbpj/Rgcc/Sema3f/Sema4a/Sema4b/Sema5a/Sema6a/Sema6d/Serpinb3/Sfrp1/Snai1/Snai2/Tmem100/Wnt5a/Zeb2	35
GO:0006869	lipid transport	51/1242	387/18303	3.93E-06	0.000117	7.99E-05	Abca8/Abcb1a/Abcb1b/Abcd1/Acat2/Acsl1/Acsl3/Acsl4/Agtr1a/Apol9a/Atp10d/Atp11a/Bdkrb2/C1qtnf1/Cpt1b/Erfe/Fabp3/Fabp4/Fabp5/Fzd4/Il1a/Il1b/Inhba/Itgb3/Kcnn4/Lbp/Ldlr/Lipg/Nfkbia/Nos2/Nrg1/P2ry2/Pcsk9/Pla2g2a/Pla2g5/Pla2r1/Plin2/Pltp/Ppard/Slc66a2/Ptges/Mvb/Slc27a3/Slco3a1/Star/Stard4/Syk/Thbs1/Tnf/Trpv4/T	51
GO:0050678	regulation of epithelial cell proliferation	51/1242	387/18303	3.93E-06	0.000117	7.99E-05	Agtr1a/Bmp2/Bmp4/Bmp5/Cask/Ccl2/Ccl5/Ccnd1/Cdh3/Cflar/Cxcl12/Cyp7b1/Dusp10/Ednrb/Eppk1/Errfi1/Esrp2/F3/Fgf9/Fgfr2/Flt1/Flt4/Gas1/Gata2/Gli1/Has2/Hmga2/Hyal1/Itgb3/Jaml/Jun/Mef2c/Mmp12/Nod2/Pdgfb/Pgf/Plau/Ppard/Rap1gap/Rgcc/Sema5a/Sfrp1/Snai2/Stat1/Thbs1/Thbs4/Tnf/Tnfaip3/Twist2/Wfdc1/Wnt5a	51
GO:1905517	macrophage migration	15/1242	57/18303	4.02E-06	0.000119	8.13E-05	Ccl2/Ccl3/Ccl5/Cd200r1/Cmklr1/Csf1/Cx3cl1/Ednrb/Emilin1/Lgals3/Mcoln2/Rarres2/Thbs1/Tnfsf18/Trpv4	15
GO:0001659	temperature homeostasis	31/1242	189/18303	4.07E-06	0.00012	8.18E-05	Acot11/Acsl1/Adora1/Arrdc3/Ccl5/Cmklr1/Cxcr4/Drd1/Ednrb/Egr1/Fabp4/Fabp5/Flcn/Gadd45g/Hadh/Htr2a/Il15/Il1a/Il1b/Il1rn/Nova1/Npr3/Ppargc1a/Prkab2/Ptgs2/Rbpj/Scd/Syk/Thra/Tnf/Tshr	31
GO:0071605	monocyte chemotactic protein-1	9/1242	21/18303	4.13E-06	0.00012	8.22E-05	C1qtnf3/Gstp1/Il1a/Il1b/Lgals9/Mcoln2/Nod2/Syk/Trpv4	9
GO:0071637	regulation of monocyte chemotactic protein-1	9/1242	21/18303	4.13E-06	0.00012	8.22E-05	C1qtnf3/Gstp1/Il1a/Il1b/Lgals9/Mcoln2/Nod2/Syk/Trpv4	9
GO:0002544	chronic inflammatory response	11/1242	32/18303	4.59E-06	0.000132	9.05E-05	Bdnf/Camp/Ccl2/Ccl5/Il1b/Il1rn/Ptges/Thbs1/Tnf/Tnfaip3/Vcam1	11
GO:2000406	positive regulation of T cell migration	11/1242	32/18303	4.59E-06	0.000132	9.05E-05	Ccl20/Ccl5/Cd99l2/Cxcl10/Cxcl12/Dock8/Itgb3/Lgals9/Pycard/Wnt5a/Xcl1	11
GO:0070528	protein kinase C signaling	12/1242	38/18303	4.68E-06	0.000134	9.17E-05	Adgrv1/Akap12/Ankrd1/Cd40/Dgkg/Fibin/Flot1/Flt4/Pdgfb/Plek/Sez6l/Wnt5a	12

ID	Description	GeneRatio	BgRatio	pvalue	p.adjust	qvalue	geneID	Count
GO:0045619	regulation of lymphocyte differentiation	31/1242	191/18303	5.09E-06	0.000145	9.93E-05	Ada/Bmp4/Card11/Cd44/Cd83/Cyp26b1/Dusp10/Flt3lg/Foxn1/Ikzf1/Il15/Il15ra/Il1a/Il1b/Il23a/Irf1/Lgals9/Lilrb4/Nlrp3/Pglyrp1/Pglyrp4/Prdm1/Myb/Rasgrp1/Ripk2/Sfrp1/Sox12/Syk/Tnfsf18/Tox/Vsir	31
GO:0051928	positive regulation of calcium ion transport	26/1242	146/18303	5.21E-06	0.000147	0.000101	Adcyap1r1/Agtr1a/Ank2/Bdkrb1/Bmp4/Cacnb2/Cask/Casq1/Ccl2/Ccl3/Ccl5/Ccr1/Cemip/Cx3cl1/Cxcl10/Cxcl11/Cxcl12/Cxcr4/Drd1/Lgals3/Mylk/P2ry6/Pdgfb/Ryr2/Trpv3/Xcl1	26
GO:0007565	female pregnancy	42/1242	298/18303	5.22E-06	0.000147	0.000101	A1cf/Acsl4/Adra2a/Akr1b1/Arhgdib/Ccl2/Csmd1/Cyp27b1/Cyp2j4/Ets1/Fbln1/Flt1/Fosl1/Gjb2/Hfe/Hpgd/Hsd17b7/Igfbp5/Il1rn/Irf1/Acod1/Itga2/Itga5/Itgb3/Junb/Lgals9/Lif/Mmp12/Mmp3/Mmp9/Nampt/Pcsk5/Pgf/Plau/Ppard/Prdm1/Prl8a9/Ptger4/Ptgs2/Ramp2/Thbd/Vim	42
GO:0003279	cardiac septum development	24/1242	129/18303	5.45E-06	0.000153	0.000105	Adamts6/Ank2/Bmp4/Bmp5/Bmp7/Cxcr4/Dhrs3/Fgfr2/Fuz/Fzd2/Gata6/Gja5/Hey2/Pax8/Pcsk5/Pde2a/Plxnd1/Prdm1/Rbm15/Rbpj/Slit2/Slit3/Tbx3/Wnt5a	24
GO:0002224	toll-like receptor signaling pathway	21/1242	104/18303	5.50E-06	0.000154	0.000105	Cd180/Cd40/Flot1/Irak2/Irf1/Acod1/Lbp/Lgals9/Nfkbia/Nod2/Peli1/Ripk2/Rsad2/Tlr2/Tlr3/Tlr5/Tlr6/Tnf/Tnfaip3/Tnip1/Tril	21
GO:0007411	axon guidance	37/1242	249/18303	5.52E-06	0.000154	0.000105	Ablim1/Alcam/Ank3/Bdnf/Bmp7/Cdh4/Chn1/Cxcl12/Cxcr4/Efna1/Epha4/Ephb2/Ephb3/Etv1/Flrt3/Gas1/Gbx2/Lama2/Matn2/Ncam1/Nectin1/Plxna2/Plxna3/Plxnd1/Ptpro/Sema3f/Sema4a/Sema4b/Sema5a/Sema6a/Sema6d/Slit2/Slit3/Tubb2b/Tubb3/Wnt5a/Zswim5	37
GO:0019233	sensory perception of pain	27/1242	156/18303	6.02E-06	0.000166	0.000114	Adora1/Aqp1/Bdkrb1/Bdkrb2/Ccl2/Ccl3/Cxcl12/Cxcr4/Ednrb/Fabp5/Grin2a/Hoxb8/Htr2a/Htr7/Il1a/Il1rn/Itga2/Lxn/Mgll/Ncam1/Ngf/Npy1r/P2ry2/Penk/Ptgs2/Tmem1	27
GO:0051249	regulation of lymphocyte activation	57/1242	457/18303	6.06E-06	0.000166	0.000114	Ada/Adora2a/Blm/Bmp4/Card11/Ccl2/Ccl5/Cd274/Cd40/Cd44/Cd83/Clcf1/Coro1a/Cyp26b1/Dock8/Dusp10/Exosc6/Ripor2/Fgr/Flt3lg/Foxn1/Hfe/Ikzf1/Il15/Il15ra/Il1a/Il1b/Il23a/Irf1/Itgal/Lgals3/Lgals9/Lilrb4/Mef2c/Nlrp3/Nod2/Peli1/Pglyrp1/Pglyrp4/Prdm1/Pycard/Myb/Rasal3/Rasgrp1/Ripk2/Sfrp1/Siglec10/Sirpa/Sox12/Syk/Tnfaip3/Tnfrsf1b/Tnfsf18/Tox/Vcam1/Vsir/Xcl1	57
GO:0097485	neuron projection guidance	37/1242	250/18303	6.06E-06	0.000166	0.000114	Ablim1/Alcam/Ank3/Bdnf/Bmp7/Cdh4/Chn1/Cxcl12/Cxcr4/Efna1/Epha4/Ephb2/Ephb3/Etv1/Flrt3/Gas1/Gbx2/Lama2/Matn2/Ncam1/Nectin1/Plxna2/Plxna3/Plxnd1/Ptpro/Sema3f/Sema4a/Sema4b/Sema5a/Sema6a/Sema6d/Slit2/Slit3/Tubb2b/Tubb3/Wnt5a/Zswim5	37
GO:0016045	detection of	8/1242	17/18303	6.15E-06	0.000168	0.000115	Naip6/Nlrc4/Nod2/Pglyrp1/Pglyrp4/Ssc5d/Tlr2/Tlr6	8
GO:0051384	response to glucocorticoid	42/1242	300/18303	6.18E-06	0.000168	0.000115	Abcb1a/Agtr1a/Anxa3/Aqp1/Areg/Ass1/Bmp4/Cacna1g/Ccl2/Ccl5/Ccnd1/Cflar/Crebrf/Cxcl1/Cxcl2/Cyp1b1/Errfi1/Fabp4/Fibin/Fosl1/Gjb2/Gstp1/Htr7/Il1b/Il1rn/Junb/Mgp/Ngf/Nos2/Pcsk1/Plat/Ptgs2/Serpina3n/Sgk1/Slit2/Slit3/Socs3/Star/Tnf/Ucn2/Ugt1a6/Wnt5a	42
GO:0003151	outflow tract morphogenesis	18/1242	81/18303	6.24E-06	0.000168	0.000115	Bmp4/Bmp7/Dhrs3/Fgfr2/Foxc1/Fzd2/Gata6/Gja5/Hand2/Hey2/Jun/Mef2c/Npy1r/Plxnd1/Rbpj/Tbx3/Thbs1/W	18
GO:0032722	positive regulation of chemokine	14/1242	52/18303	6.28E-06	0.000168	0.000115	Il1a/Il1b/Lbp/Lgals9/Mcoln2/Nod2/P2ry2/Ripk2/Syk/Tlr2/Tlr3/Tnf/Trpv4/Tslp	14
GO:0048846	axon extension involved in axon guidance	12/1242	39/18303	6.34E-06	0.000168	0.000115	Alcam/Cxcl12/Plxna3/Sema3f/Sema4a/Sema4b/Sema5a/Sema6a/Sema6d/Slit2/Slit3/Wnt5a	12
GO:1902284	neuron projection extension involved in neuron projection guidance	12/1242	39/18303	6.34E-06	0.000168	0.000115	Alcam/Cxcl12/Plxna3/Sema3f/Sema4a/Sema4b/Sema5a/Sema6a/Sema6d/Slit2/Slit3/Wnt5a	12
GO:2000403	positive regulation of lymphocyte	12/1242	39/18303	6.34E-06	0.000168	0.000115	Ccl20/Ccl3/Ccl5/Cd99l2/Cxcl10/Cxcl12/Dock8/Itgb3/Lgals9/Pycard/Wnt5a/Xcl1	12
GO:0032757	positive regulation of interleukin-8 production	15/1242	59/18303	6.39E-06	0.000169	0.000116	Camp/F3/Hspa1b/Il1b/Lbp/Lgals9/Nod2/Ptger4/Pycard/Syk/Tlr2/Tlr3/Tlr5/Tnf/Wnt5a	15
GO:0045637	regulation of myeloid cell differentiation	34/1242	222/18303	6.74E-06	0.000177	0.000121	Ccl3/Ccl5/Ccr1/Csf1/Csf2/Csf3/Dll1/Ets1/Faxdc2/Gata2/Gpr68/Hoxb8/Hspa1b/Ikzf1/Il23a/Inhba/Isg15/Jun/Kitlg/LOC102551184/Lif/Lilrb4/Lmo2/Mafb/Mef2c/Nfkbia/RGD1562378/Rbm15/Rbp1/Scin/Sfrp1/Stat1/Tnf/Twi	34
GO:0010876	lipid localization	55/1242	438/18303	7.20E-06	0.000189	0.000129	Abca8/Abcb1a/Abcb1b/Abcd1/Acat2/Acsl1/Acsl3/Acsl4/Agtr1a/Apol9a/Atp10d/Atp11a/B4galnt1/Bdkrb2/C1qtnf1/Cpt1a/Cpt1b/Erfe/Fabp3/Fabp4/Fabp5/Fzd4/Hilpda/Il1a/Il1b/Inhba/Itgb3/Kcnn4/Lbp/Ldlr/Lipg/Nfkbia/Nos2/Nrg1/P2ry2/Pcsk9/Pla2g2a/Pla2g5/Pla2r1/Plin2/Pltp/Ppard/Slc66a2/Ptges/Myb/Slc27a3/Slco3a1/Sqle/Star/Stard4/Syk/Thbs1/Tnf/Trpv4/Ttc39b	55
GO:0044706	multi-multicellular organism process	45/1242	333/18303	7.48E-06	0.000195	0.000133	A1cf/Acsl4/Adra2a/Akr1b1/Arhgdib/Ccl2/Csmd1/Cyp27b1/Cyp2j4/Dnm3/Ets1/Fbln1/Flt1/Fosl1/Gjb2/Hfe/Hpgd/Hsd17b7/Igfbp5/Il1rn/Irf1/Acod1/Itga2/Itga5/Itgb3/Junb/Klk14/Lgals9/Lif/Mmp12/Mmp13/Mmp3/Mmp9/Nampt/Pcsk5/Pgf/Plau/Ppard/Prdm1/Prl8a9/Ptger4/Ptgs2/Ramp2/Thbd/Vim	45
GO:0032602	chemokine production	18/1242	82/18303	7.49E-06	0.000195	0.000133	C1qtnf3/Gstp1/Il1a/Il1b/Lbp/Lgals9/Mcoln2/Nod2/P2ry2/Ripk2/Sirpa/Snai2/Syk/Tlr2/Tlr3/Tnf/Trpv4/Tslp	18

ID	Description	GeneRatio	BgRatio	pvalue	p.adjust	qvalue	geneID	Count
GO:2000401	regulation of lymphocyte	16/1242	67/18303	7.61E-06	0.000197	0.000134	Ccl2/Ccl20/Ccl3/Ccl5/Cd200r1/Cd99l2/Cxcl10/Cxcl12/Dock8/Ripor2/Itgb3/LOC290595/Lgals9/Pycard/Wnt5a/	16
GO:0002221	pattern recognition receptor signaling pathway	26/1242	149/18303	7.64E-06	0.000197	0.000134	Cd180/Cd40/Clec4e/Flot1/Hspa1b/Irak2/Irf1/Acod1/Lbp/Lgals9/Nfkbia/Nod2/Peli1/Inava/Riok3/Ripk2/Rsad2/Tifa/Tlr2/Tlr3/Tlr5/Tlr6/Tnf/Tnfaip3/Tnip1/Tril	26
GO:0051251	positive regulation of lymphocyte activation	40/1242	283/18303	8.09E-06	0.000207	0.000142	Ada/Blm/Card11/Ccl2/Ccl5/Cd274/Cd40/Cd83/Clcf1/Coro1a/Dock8/Dusp10/Exosc6/Flt3lg/Ikzf1/Il15/Il15ra/Il1a/Il1b/Il23a/Itgal/Lgals9/Lilrb4/Mef2c/Nlrp3/Nod2/Peli1/Prdm1/Pycard/Myb/Rasal3/Rasgrp1/Ripk2/Sirpa/Sox12/Syk/Tox/Vcam1/Vsir/Xcl1	40
GO:0050863	regulation of T cell activation	44/1242	324/18303	8.30E-06	0.000212	0.000145	Ada/Adora2a/Blm/Bmp4/Card11/Ccl2/Ccl5/Cd274/Cd44/Cd83/Coro1a/Cyp26b1/Dock8/Dusp10/Ripor2/Foxn1/Hfe/Ikzf1/Il15/Il1a/Il1b/Il23a/Irf1/Itgal/Lgals3/Lgals9/Lilrb4/Nlrp3/Peli1/Prdm1/Pycard/Myb/Rasal3/Rasgrp1/Ripk2/Sirpa/Sox12/Syk/Tnfrsf1b/Tnfsf18/Tox/Vcam1/Vs	44
GO:0051767	nitric-oxide synthase biosynthetic process	10/1242	28/18303	8.47E-06	0.000214	0.000146	Akap12/Ccl2/Ccl20/Gstp1/Il33/Nampt/Nod2/Ptger4/Stat1/Tlr2	10
GO:0051769	regulation of nitric-oxide synthase biosynthetic process	10/1242	28/18303	8.47E-06	0.000214	0.000146	Akap12/Ccl2/Ccl20/Gstp1/Il33/Nampt/Nod2/Ptger4/Stat1/Tlr2	10
GO:0003205	cardiac chamber development	31/1242	196/18303	8.74E-06	0.000219	0.00015	Adamts6/Ank2/Bmp4/Bmp5/Bmp7/Col11a1/Cxcr4/Dhrs3/Fgfr2/Foxc1/Fuz/Fzd2/Gata6/Gja5/Hand2/Hey2/Kcnk2/Mef2c/Nrg1/Pax8/Pcsk5/Pde2a/Plxnd1/Prdm1/Rbm15/Rbpj/Ryr2/Slit2/Slit3/Tbx3/Wnt5a	31
GO:0070661	leukocyte proliferation	45/1242	335/18303	8.75E-06	0.000219	0.00015	Ada/Blm/Bmp4/Card11/Ccl5/Cd180/Cd274/Cd40/Cd44/Clcf1/Coro1a/Csf1/Cx3cl1/Cxcl12/Cxcr4/Dock8/Ripor2/Flt3lg/Gstp1/Il15/Il1a/Il1b/Il23a/Il33/Irf1/Itgal/Kitlg/Lgals3/Lgals9/Mef2c/Npr3/Peli1/Prdm1/Pycard/Rasal3/Rasgrp1/Ripk2/Satb1/Siglec10/Syk/Tnfrsf1b/Tnfsf18/V	45
GO:0007162	negative regulation of cell adhesion	41/1242	295/18303	9.50E-06	0.000237	0.000162	Adora2a/Akna/Ass1/Bmp2/Bmp4/C1qtnf1/Cask/Cd274/Cd44/Col1a1/Coro2b/Cxcl12/Cyp1b1/Dact2/Enpp2/Ripor2/Fbln1/Fzd4/Hfe/Il1rn/Irf1/Lgals3/Lgals9/Lpxn/Meltf/Mmp12/Myo1f/Peli1/Plet1/Plxna2/Plxna3/Plxnd1/Rgcc/Sema5a/Sema6a/Snai2/Thbs1/Tnfsf18/Trpv4/Vsir/Xcl	41
GO:0001933	negative regulation of protein phosphorylation	54/1242	432/18303	9.94E-06	0.000246	0.000168	Adora2a/Atf3/Bdkrb1/Bdkrb2/Bmp4/Bmp7/Cadm4/Drd1/Dusp10/Dusp16/Dusp19/Dusp5/Dusp6/Efna1/Emilin1/Ephb2/Errfi1/Fabp4/Fbln1/Flcn/Gadd45g/Gstp1/Hmgcr/Il1b/Irf1/Jun/Itprip/Lif/Mical1/Ndrg2/Pcp4/Plpp3/Ppargc1a/Ppp1r15a/Prkar2a/Prkar2b/Ptpn5/Ptprb/Ptpro/Ptprt/Pycard/Rgs14/Rnf149/Sema6a/Serpinb3/Sfrp1/Sirpa/Slit2/Smcr8/Socs3/Spry4/Timp3/Tnfaip3/Tnip1	54
GO:0050707	regulation of cytokine secretion	15/1242	61/18303	9.94E-06	0.000246	0.000168	Agtr1a/C1qtnf3/Cadm1/Clec4e/Cx3cl1/Fgr/Hdac9/Il1a/Ptger4/Rgcc/Syk/Tlr2/Tlr5/Tnf/Tnfsf15	15
GO:0003012	muscle system process	55/1242	443/18303	1.01E-05	0.000248	0.00017	Ada/Adora1/Adra2a/Ank2/Bdkrb1/Bdkrb2/Cacna1g/Cacnb2/Calcrl/Casq1/Cflar/Col14a1/Cxcr4/Dock4/Drd1/Ednrb/Errfi1/Fdps/Flt1/Gata6/Gja5/Glra1/Hand2/Hey2/Htr2a/Htr7/Igfbp5/Il15/Itga2/Kcnh2/Mef2c/Mybpc1/Myh1/Myl1/Mylk/Npnt/Npy1r/Pde4b/Pi16/Ppargc1a/Ptger4/Ptgs2/Ryr2/Ryr3/Scn2b/Sgca/Sgcd/Sorbs2/Tbx3/Tmod1/Tmod2/Tnf/Tnfrsf1b/Tnnt3/Trpv4	55
GO:0071498	cellular response to fluid shear stress	9/1242	23/18303	1.01E-05	0.000249	0.00017	Ass1/Has2/Klf2/Mef2c/Mmp1/Mmp13/Plau/Ptgs2/Tfpi2	9
GO:2000181	negative regulation of blood vessel morphogenesis	23/1242	125/18303	1.03E-05	0.000251	0.000172	Angpt4/Ccl2/Creb3l1/Cxcl10/Dcn/Emilin1/Fbln5/Flt1/Foxc1/Gpr4/Klf2/Lif/Meox2/Plk2/Ptger4/Rgcc/Sema4a/Sema6a/Stat1/Thbs1/Thbs2/Thbs4/Tnf	23
GO:0098543	detection of other organism	8/1242	18/18303	1.04E-05	0.000253	0.000173	Naip6/Nlrc4/Nod2/Pglyrp1/Pglyrp4/Ssc5d/Tlr2/Tlr6	8
GO:0043409	negative regulation of MAPK cascade	29/1242	179/18303	1.05E-05	0.000255	0.000174	Atf3/Bmp4/Bmp7/Dusp10/Dusp16/Dusp19/Dusp5/Dusp6/Efna1/Emilin1/Ephb2/Errfi1/Fbln1/Flcn/Gstp1/Hmgcr/Il1b/Lif/Ndrg2/Ptpn5/Rgs14/Rnf149/Sema6a/Serpinb3/Sfrp1/Sirpa/Spry4/Timp3/Tnip1	29
GO:0032652	regulation of interleukin-1 production	20/1242	100/18303	1.06E-05	0.000256	0.000175	Ccl20/Ccl3/Cx3cl1/Egr1/Errfi1/Fzd5/Gstp1/Lgals9/Nlrc4/Nlrp3/Nod2/Ptger4/Pycard/Inava/Ripk2/Sirpa/Tlr2/Tnf/Tnfaip3/Wnt5a	20
GO:0048706	embryonic skeletal system development	25/1242	143/18303	1.10E-05	0.000264	0.00018	Bmp4/Bmp7/Col11a1/Col1a1/Col2a1/Dmrt2/Fgf9/Fgfr2/Fuz/Gas1/Hoxb5/Hoxb8/Hoxc6/Hsd17b7/Hyal1/Mdfi/Mef2c/Mmp16/Nkx3-	25
GO:0046632	alpha-beta T cell differentiation	22/1242	117/18303	1.11E-05	0.000266	0.000182	Ada/Bcl3/Blm/Cd83/Ikzf1/Il23a/Irf1/Lgals9/Nlrp3/Pax1/Prdm1/Ptger4/Myb/Relb/Ripk2/Rsad2/Satb1/Sema4a/Syk/Tnfsf18/Tnfsf8/Tox	22
GO:0060485	mesenchyme development	40/1242	287/18303	1.14E-05	0.000271	0.000185	Akna/Bmp2/Bmp4/Bmp5/Bmp7/Col1a1/Ednrb/Efna1/Fgf9/Fgfr2/Foxc1/Frzb/Fuz/Gbx2/Hand2/Has2/Hey2/Hmga2/Il1b/Kitlg/Mef2c/Pax1/Plaur/Rbpj/Rgcc/Scx/Sema3f/Sema4a/Sema4b/Sema5a/Sema6a/Sema6d/Serpinb3/Sfrp1/Snai1/Snai2/Thbs1/Tmem100/Wnt5a/Zeb2	40
GO:0070663	regulation of leukocyte proliferation	37/1242	257/18303	1.15E-05	0.000273	0.000186	Ada/Blm/Bmp4/Card11/Ccl5/Cd274/Cd40/Cd44/Clcf1/Coro1a/Csf1/Ripor2/Flt3lg/Gstp1/Il15/Il1a/Il1b/Il23a/Il33/Irf1/Itgal/Kitlg/Lgals3/Lgals9/Mef2c/Peli1/Prdm1/Pycard/Rasal3/Ripk2/Siglec10/Syk/Tnfrsf1b/Tnfsf18/Vca	37

ID	Description	GeneRatio	BgRatio	pvalue	p.adjust	qvalue	geneID	Count
GO:0042742	defense response to bacterium	41/1242	298/18303	1.22E-05	0.000287	0.000197	Adamts4/Anxa3/Bcl3/Camp/Clec4e/Fgr/Gbp4/Il23a/Isg15/LOC290595/Lbp/Lgals9/Lypd8/Lyz2/Mr1/Myo1f/Naip6/Nlrc4/Nlrp3/Nod2/Nos2/Pglyrp1/Pglyrp4/Pla2g2a/Pycard/Rarres2/Rbpj/Ripk2/Scd/Serpinb9/Ssc5d/Syk/Tfeb/Tlr2/Tlr5/Tnf/Tnfsf8/Trem3/Tslp/Wfdc18/Wfdc21	41
GO:0043507	positive regulation of JUN kinase activity	17/1242	77/18303	1.23E-05	0.000289	0.000198	Dusp19/Epha4/Ern1/Fzd4/Fzd5/Fzd8/Il1b/Il1rn/Map3k9/Mdfi/Ripk2/Syk/Tlr6/Tnf/Tnik/Wnt5a/Zeb2	17
GO:0002697	regulation of immune effector process	54/1242	436/18303	1.30E-05	0.000304	0.000208	Cadm1/Ccl2/Cd40/Cfh/Clcf1/Cxcl1/Dusp10/Exosc6/Fgr/Fzd5/Gata2/Hfe/Hspa1b/Il13ra2/Il15/Il18rap/Il1b/Il1r1/Il23a/Il33/Lbp/Lgals3/Lgals9/Micb/Mmp12/Mr1/Nlrp3/Nod2/Pvr/Pglyrp1/Pglyrp4/Pla2g5/Pycard/Myb/Rasgrp1/Riok3/Ripk2/Rsad2/Serpinb3a/Serpinb9/Serping1/Siglec10/Stat1/Syk/Tlr2/Tlr3/Tnf/Tnfrsf1b/Tnfsf18/Tril/Trim6/Vsir/Wnt5a/Xcl1	54
GO:0051153	regulation of striated muscle cell differentiation	23/1242	127/18303	1.35E-05	0.000314	0.000215	Bdnf/Bmp2/Bmp4/Cd53/Cflar/Col14a1/Cxcl10/Cxcl12/Cyp26b1/Dll1/Ehd2/Ripor2/Fdps/Flot1/Flt3lg/Gdf15/Hdac9/Mef2c/Nrg1/Pi16/Rbm38/Rbpj/Thra	23
GO:0002791	regulation of peptide secretion	52/1242	415/18303	1.35E-05	0.000314	0.000215	A1cf/Aacs/Acsl4/Adcy8/Adora1/Adora2a/Adra2a/Agtr1a/Ankrd1/C1qtnf3/Cadm1/Cask/Ccl5/Cd40/Clec4e/Cpt1a/Cx3cl1/Cyp51/Fgr/Frmd4a/Gja5/Gpr68/Hadh/Hdac9/Hfe/Hmga2/Hmgcr/Il1a/Il1b/Il33/Kcnn4/Lif/Mmp13/Hcar2/Nos2/Pax8/Pcsk1/Ppard/Ptger4/Rab11fip1/Rfx3/Rgcc/Rsad2/Sfrp1/Siglec10/Sybu/Syk/Tlr2/Tlr5/Tnf/Tnfsf	52
GO:0032609	interferon-gamma production	24/1242	136/18303	1.39E-05	0.000322	0.00022	Bcl3/Cd274/Fzd5/Il18rap/Il1b/Il1r1/Il23a/Il33/Isg15/Lgals9/Nod2/Pde4b/Pglyrp1/Pglyrp4/Pycard/Rasgrp1/Ripk2/Rnf19b/Sirpa/Tlr3/Tnf/Vsir/Wnt5a/Xcl1	24
GO:0071639	positive regulation of monocyte chemotactic protein-1	7/1242	14/18303	1.46E-05	0.000337	0.00023	Il1a/Il1b/Lgals9/Mcoln2/Nod2/Syk/Trpv4	7
GO:1903531	negative regulation of secretion by cell	31/1242	201/18303	1.47E-05	0.000337	0.00023	Acsl4/Adora1/Adra2a/Cx3cl1/Cyp51/Frmd4a/Hadh/Hdac9/Hmgcr/Htr7/Il11/Il13ra2/Il1b/Il1rn/Il33/Inha/Lgals9/Lif/Npy1r/Nrg1/Ptger4/Rab11fip1/Rap1b/Rgcc/Rsad2/Sfrp1/Siglec10/Tcp11/Tnfrsf1b/Ucn2/Vsnl1	31
GO:0070373	negative regulation of ERK1 and ERK2 cascade	17/1242	78/18303	1.47E-05	0.000337	0.000231	Atf3/Dusp10/Dusp6/Emilin1/Ephb2/Errfi1/Fbln1/Flcn/Gstp1/Lif/Ndrg2/Rgs14/Sema6a/Sirpa/Spry4/Timp3/Tnip1	17
GO:0010632	regulation of epithelial cell migration	35/1242	240/18303	1.48E-05	0.000338	0.000231	Angpt4/Anxa3/Aqp1/Bmp4/Bmper/Cd40/Dcn/Dusp10/Efna1/Enpp2/Eppk1/Ets1/Flt4/Gata2/Has2/Hdac9/Hyal1/Itga2/Itgb3/Jun/Mef2c/Meox2/Mmp9/Pdgfb/Plk2/Plpp3/Ptgs2/Rgcc/Rhoj/Sema4a/Sema5a/Slit2/Thbs1/Tnf/Wnt	35
GO:0060393	regulation of pathway-restricted SMAD protein phosphorylation	15/1242	63/18303	1.51E-05	0.000342	0.000234	Bmp2/Bmp3/Bmp4/Bmp5/Bmp7/Bmper/Emilin1/Gdf15/Gdf6/Hfe/Inha/Inhba/Inhbe/Lgals9/Rbpms	15
GO:0010759	positive regulation of macrophage chemotaxis	9/1242	24/18303	1.53E-05	0.000342	0.000234	Ccl2/Ccl5/Cmklr1/Csf1/Cx3cl1/Rarres2/Thbs1/Tnfsf18/Trpv4	9
GO:0098581	detection of external biotic stimulus	9/1242	24/18303	1.53E-05	0.000342	0.000234	Lbp/Naip6/Nlrc4/Nod2/Pglyrp1/Pglyrp4/Ssc5d/Tlr2/Tlr6	9
GO:1901739	regulation of myoblast fusion	9/1242	24/18303	1.53E-05	0.000342	0.000234	Cd53/Cflar/Cxcl10/Cxcl12/Ehd2/Ripor2/Flot1/Flt3lg/Gdf15	9
GO:0001704	formation of primary germ layer	23/1242	128/18303	1.54E-05	0.000343	0.000235	Bmp4/Bmp7/Chrd/Col11a1/Col5a1/Col7a1/Dusp5/Fgfr2/Foxc1/Gata6/Hmga2/Hnf1b/Inhba/Itga2/Itga5/Itga7/Itgb3/Lamb3/Mmp9/Scx/Snai1/Txnrd1/Wnt5a	23
GO:0002831	regulation of response to biotic stimulus	41/1242	301/18303	1.55E-05	0.000343	0.000235	Cadm1/Ccr1/Cd180/Cd274/Cx3cl1/Cxcl1/Dusp10/Ereg/Fgr/Gbp5/Il15/Il18rap/Il1b/Il23a/Acod1/Lbp/Lgals9/Lrp8/Micb/Mmp12/Mr1/Myo1f/Nlrc4/Nlrc5/Nod2/Pvr/Pla2g5/Prdm1/Pycard/Rasgrp1/Riok3/Ripk2/Serpinb3a/Serpinb9/Serping1/Stat1/Tlr2/Tnfaip3/Trem3/Trim6/Wnt5a	41
GO:0010631	epithelial cell migration	41/1242	301/18303	1.55E-05	0.000343	0.000235	Angpt4/Anxa3/Aqp1/Bmp4/Bmper/Cd40/Cyp1b1/Dcn/Dusp10/Efna1/Enpp2/Eppk1/Ets1/Flt4/Gata2/Has2/Hdac9/Hyal1/Itga2/Itgb3/Jun/Lpxn/Mef2c/Meox2/Mmp9/Pdgfb/Plk2/Plpp3/Plxnd1/Ppard/Ptgs2/Ptp4a3/Rgcc/Rhoj/Sema4a/Sema5a/Slit2/Thbs1/Tnf/Wnt5a/Zeb2	41
GO:0022612	gland morphogenesis	27/1242	164/18303	1.56E-05	0.000343	0.000235	Areg/Bmp4/Bmp7/Cd44/Cflar/Crip1/Csf1/Csmd1/Cyp7b1/Edar/Elf3/Esrp2/Fgfr2/Gli1/Igfbp5/Ntn4/Pdgfb/Plau/Plxnd1/Sfrp1/Slit2/Snai2/Tbx3/Tgm2/Tnf/Tnfaip3/Wnt	27
GO:0033280	response to vitamin D	14/1242	56/18303	1.60E-05	0.000349	0.000238	Abcb1a/Bmp7/Cxcl10/Cyp27b1/Fzd2/Il15/Il1b/Mmp1/Nos2/Penk/Ptgs2/Sfrp1/Snai2/Vim	14
GO:0010862	positive regulation of pathway-restricted SMAD protein phosphorylation	13/1242	49/18303	1.60E-05	0.000349	0.000238	Bmp2/Bmp3/Bmp4/Bmp5/Bmp7/Gdf15/Gdf6/Hfe/Inha/Inhba/Inhbe/Lgals9/Rbpms	13
GO:1902622	regulation of neutrophil migration	13/1242	49/18303	1.60E-05	0.000349	0.000238	Cd99l2/Dapk2/Ripor2/Il1a/Il1b/Il1r1/Il23a/Lbp/Nod2/Ptger4/Slit2/Thbs4/Xcl1	13
GO:0034599	cellular response to oxidative stress	45/1242	343/18303	1.61E-05	0.000349	0.000239	Abcd1/Akr1b1/Aldh3b1/Aqp1/Bmp4/Casp12/Ccl5/Cfh/Cflar/Cpeb2/Cyp1b1/Ern1/Ets1/F3/Fbln5/Gch1/Ggt1/Gjb2/Gpr37/Gpr37l1/Hk3/Il18rap/Jun/Klf2/Klf6/Mgat3/Mmp3/Mmp9/Nefh/Net1/Nox1/Nqo1/Penk/Pla2r1/Ppargc1a/Ppif/Pycr1/Myb/Sirpa/Slc1a1/Slc7a11/Thbs1/Tlr6/Tn	45

ID	Description	GeneRatio	BgRatio	pvalue	p.adjust	qvalue	geneID	Count
GO:0032649	regulation of interferon-gamma production	22/1242	120/18303	1.69E-05	0.000363	0.000249	Bcl3/Cd274/Fzd5/Il1b/Il1r1/Il23a/Il33/Isg15/Lgals9/Nod2/Pde4b/Pglyrp1/Pglyrp4/Pycard/Rasgrp1/Ripk2/Sirpa/Tlr3/Tnf/Vsir/Wnt5a/Xcl1	22
GO:2000379	positive regulation of reactive oxygen species metabolic process	22/1242	120/18303	1.69E-05	0.000363	0.000249	Agtr1a/Ass1/Cxcl1/Cyp1b1/Ddah2/Gstp1/Hvcn1/Il1b/Acod1/Klf2/Nox1/Olr1/Pdgfb/Plau/Ptgs2/Slc5a3/Syk/Thbs1/Tlr2/Tlr5/Tlr6/Tnf	22
GO:0071636	positive regulation of transforming growth factor beta	8/1242	19/18303	1.69E-05	0.000363	0.000249	Cx3cl1/Furin/Lgals9/Ptgs2/Myb/Serpinf2/Thbs1/Xcl1	8
GO:0062012	regulation of small molecule metabolic process	48/1242	376/18303	1.76E-05	0.000377	0.000258	Abcd1/Adcyap1r1/Bmp2/Bmp5/C1qtnf1/C1qtnf3/Clybl/Cpt1a/Cyp27b1/Cyp51/Egr1/Erfe/Fabp3/Fabp5/Fdps/Flcn/Fmo5/Has2/Hpgd/Htr2a/Ier3/Igfbp4/Il1b/Insig1/Ldlr/Nfkb1/Nos2/P2ry6/Pdgfb/Plek/Ppard/Ppargc1a/Ppp1r3b/Ppp1r3c/Ppp1r3e/Ppp1r3g/Ptger4/Ptgs2/Pth1r/Slc2a6/Slc7a11/Snai1/Snai2/Star/Stard4/Thrb/Tnf/Ttc39b	48
GO:0090132	epithelium migration	41/1242	303/18303	1.82E-05	0.000388	0.000265	Angpt4/Anxa3/Aqp1/Bmp4/Bmper/Cd40/Cyp1b1/Dcn/Dusp10/Efna1/Enpp2/Eppk1/Ets1/Flt4/Gata2/Has2/Hdac9/Hyal1/Itga2/Itgb3/Jun/Lpxn/Mef2c/Meox2/Mmp9/Pdgfb/Plk2/Plpp3/Plxnd1/Ppard/Ptgs2/Ptp4a3/Rgcc/Rhoj/Sema4a/Sema5a/Slit2/Thbs1/Tnf/Wnt5a/Zeb2	41
GO:0002367	cytokine production involved in immune response	21/1242	112/18303	1.83E-05	0.000389	0.000266	Fzd5/Hfe/Il18rap/Il1b/Il1r1/Il31ra/Mr1/Nlrp3/Nod2/Inava/Rsad2/Tlr2/Tlr3/Tnf/Tnfrsf1b/Trem3/Tril/Trim6/Vsir/Wnt5a/Xcl1	21
GO:0007178	transmembrane receptor protein serine/threonine kinase signaling pathway	47/1242	366/18303	1.84E-05	0.00039	0.000267	Bmp2/Bmp3/Bmp4/Bmp5/Bmp7/Bmper/Ccl2/Chrd/Col3a1/Dact2/Egr1/Emilin1/Fam83g/Fgf9/Flcn/Furin/Gata6/Gdf15/Gdf6/Grem2/Hfe/Hipk2/Hpgd/Hspa1b/Htra3/Inha/Inhba/Inhbe/Jun/Lgals9/Lpxn/Ltbp4/Npnt/Nrep/Peg10/Rbpj/Rbpms/Rgma/Scx/Sfrp1/Slc2a10/Tgif1/Thbs1/Tmem100/Vim/Vsir/Wnt5a	47
GO:0090287	regulation of cellular response to growth factor stimulus	39/1242	283/18303	1.89E-05	0.0004	0.000273	Bmp4/Bmper/Cadm4/Cask/Cflar/Chrd/Creb3l1/Dcn/Dll1/Emilin1/Fam20c/Fgf9/Vegfd/Flcn/Flt1/Furin/Fuz/Fzd4/Gata6/Grem2/Hipk2/Hspa1b/Htra3/Ngf/Npnt/Nrep/Pdgfb/Peg10/Ptp4a3/Rbpj/Rgma/Sema6a/Sfrp1/Slc2a10/Slit2/Spry4/Thbs1/Vsir/Wnt5a	39
GO:0009306	protein secretion	57/1242	475/18303	1.92E-05	0.000403	0.000276	A1cf/Aacs/Acsl4/Adcy8/Adora2a/Adra2a/Agtr1a/Ankrd1/C1qtnf3/Cadm1/Cask/Ccl5/Cd40/Clec4e/Cpt1a/Cx3cl1/Cyp51/Fgr/Frmd4a/Gja5/Gpr68/Hadh/Hdac9/Hmgcr/Hnf1b/Il1a/Il1b/Il1rn/Il33/Kcnn4/Mafa/Mmp13/Hcar2/Nlrp3/Nod2/Nos2/Pclo/Pcsk1/Pcsk5/Plek/Ppard/Ptger4/Rab11fip1/Rab3d/Rfx3/Rgcc/Rsad2/Sfrp1/Siglec10/Sybu/Syk/Tlr2/Tlr5/Tnf/Tnfsf15/Trem3/Vsnl1	57
GO:0007157	heterophilic cell-cell adhesion via plasma membrane cell adhesion molecules	12/1242	43/18303	1.93E-05	0.000403	0.000276	Alcam/Cadm1/Cdh4/Fat4/Itga5/Itgal/Jaml/Nectin1/Pvr/Selp/Tenm4/Vcam1	12
GO:0016525	negative regulation of angiogenesis	22/1242	121/18303	1.93E-05	0.000403	0.000276	Angpt4/Ccl2/Creb3l1/Cxcl10/Dcn/Emilin1/Fbln5/Flt1/Foxc1/Gpr4/Klf2/Lif/Meox2/Plk2/Rgcc/Sema4a/Sema6a/Stat1/Thbs1/Thbs2/Thbs4/Tnf	22
GO:0031960	response to corticosteroid	43/1242	325/18303	2.00E-05	0.000416	0.000285	Abcb1a/Agtr1a/Anxa3/Aqp1/Areg/Ass1/Bmp4/Cacna1g/Ccl2/Ccl5/Ccnd1/Cflar/Col1a1/Crebrf/Cxcl1/Cxcl2/Cyp1b1/Errfi1/Fabp4/Fibin/Fosl1/Gjb2/Gstp1/Htr7/Il1b/Il1rn/Junb/Mgp/Ngf/Nos2/Pcsk1/Plat/Ptgs2/Serpina3n/Sgk1/Slit2/Slit3/Socs3/Star/Tnf/Ucn2/Ugt1a6/Wnt5a	43
GO:0033627	cell adhesion mediated by integrin	16/1242	72/18303	2.02E-05	0.000419	0.000286	Ada/Ccl5/Cyp1b1/Efna1/Itga11/Itga2/Itga5/Itgb3/Itgbl1/Lif/Lpxn/Npnt/Plau/Plpp3/Snai2/Syk	16
GO:0045621	positive regulation of lymphocyte differentiation	21/1242	113/18303	2.10E-05	0.000433	0.000296	Ada/Cd83/Dusp10/Flt3lg/Ikzf1/Il15/Il15ra/Il1a/Il1b/Il23a/Lgals9/Lilrb4/Nlrp3/Prdm1/Myb/Rasgrp1/Ripk2/Sox12/Syk/Tox/Vsir	21
GO:0045639	positive regulation of myeloid cell differentiation	21/1242	113/18303	2.10E-05	0.000433	0.000296	Ccl3/Ccl5/Ccr1/Csf1/Csf2/Csf3/Ets1/Faxdc2/Gata2/Gpr68/Hspa1b/Ikzf1/Il23a/Inhba/Isg15/Jun/Kitlg/Lif/Scin/Stat1/Tnf	21
GO:0050708	regulation of protein secretion	47/1242	368/18303	2.12E-05	0.000435	0.000298	A1cf/Aacs/Acsl4/Adcy8/Adora2a/Adra2a/Agtr1a/Ankrd1/C1qtnf3/Cadm1/Cask/Ccl5/Cd40/Clec4e/Cpt1a/Cx3cl1/Cyp51/Fgr/Frmd4a/Gja5/Gpr68/Hadh/Hdac9/Hmgcr/Il1a/Il1b/Il33/Kcnn4/Mmp13/Hcar2/Nos2/Pcsk1/Ppard/Ptger4/Rab11fip1/Rfx3/Rgcc/Rsad2/Sfrp1/Siglec10/Sybu/Syk/Tlr2/Tlr5/Tnf/Tnfsf15/Vsnl1	47
GO:0035592	establishment of protein localization to extracellular region	57/1242	477/18303	2.17E-05	0.000442	0.000302	A1cf/Aacs/Acsl4/Adcy8/Adora2a/Adra2a/Agtr1a/Ankrd1/C1qtnf3/Cadm1/Cask/Ccl5/Cd40/Clec4e/Cpt1a/Cx3cl1/Cyp51/Fgr/Frmd4a/Gja5/Gpr68/Hadh/Hdac9/Hmgcr/Hnf1b/Il1a/Il1b/Il1rn/Il33/Kcnn4/Mafa/Mmp13/Hcar2/Nlrp3/Nod2/Nos2/Pclo/Pcsk1/Pcsk5/Plek/Ppard/Ptger4/Rab11fip1/Rab3d/Rfx3/Rgcc/Rsad2/Sfrp1/Siglec10/Sybu/Syk/Tlr2/Tlr5/Tnf/Tnfsf15/Trem3/Vsnl1	57

ID	Description	GeneRatio	BgRatio	pvalue	p.adjust	qvalue	geneID	Count
GO:0010959	regulation of metal ion transport	54/1242	444/18303	2.17E-05	0.000442	0.000302	Adcyap1r1/Adora1/Adora2a/Adra2a/Agtr1a/Ank2/Ank3/Bdkrb1/Bmp4/Cacna1g/Cacnb2/Cask/Casq1/Ccl2/Ccl3/Ccl5/Ccr1/Cemip/Coro1a/Cx3cl1/Cxcl1/Cxcl10/Cxcl11/Cxcl12/Cxcr4/Drd1/Fhl1/Hfe/Htr2a/Itgb3/Kcnh2/Kcnip4/Lgals3/Lrrc38/Mylk/Ngf/Nkain4/P2ry6/Pcsk9/Pde4b/Pdgfb/Ptgs2/Rgs7/Rgs9/Ryr2/Scn2b/Sgk1/Sptbn4/Stom/Trpc4/Trpv3/Trpv4/Wnk4/Xcl1	54
GO:0002040	sprouting angiogenesis	22/1242	122/18303	2.21E-05	0.000446	0.000305	Agtr1a/Bmp4/Bmper/Creb3l1/Dll1/Esm1/Flt1/Flt4/Gata2/Hdac9/Itga5/Klf2/Meox2/Pgf/Plk2/Ptgs2/Ramp2/Rhoj/Sema5a/Sema6a/Slit2/Thbs1	22
GO:1902624	positive regulation of neutrophil migration	11/1242	37/18303	2.22E-05	0.000446	0.000305	Cd99l2/Dapk2/Ripor2/Il1a/Il1b/Il1r1/Il23a/Lbp/Ptger4/Thbs4/Xcl1	11
GO:2000191	regulation of fatty acid transport	11/1242	37/18303	2.22E-05	0.000446	0.000305	Acsl4/Erfe/Fabp3/Il1a/Il1b/P2ry2/Pla2r1/Ptges/Syk/Thbs1/Trpv4	11
GO:0045088	regulation of innate immune response	29/1242	186/18303	2.22E-05	0.000446	0.000305	Cadm1/Ccr1/Dusp10/Ereg/Fgr/Gbp5/Il18rap/Acod1/Lbp/Lgals9/Lrp8/Mmp12/Myo1f/Nlrc4/Nlrc5/Nod2/Pvr/Pla2g5/Pycard/Rasgrp1/Riok3/Serpinb3a/Serpinb9/Serping1/Tlr2/Tnfaip3/Trem3/Trim6/Wnt5a	29
GO:0045580	regulation of T cell differentiation	26/1242	158/18303	2.24E-05	0.000447	0.000306	Ada/Bmp4/Card11/Cd44/Cd83/Cyp26b1/Dusp10/Foxn1/Ikzf1/Il15/Il1a/Il1b/Il23a/Irf1/Lgals9/Lilrb4/Nlrp3/Prdm1/Myb/Rasgrp1/Ripk2/Sox12/Syk/Tnfsf18/Tox/Vsir	26
GO:0060055	angiogenesis involved in wound	9/1242	25/18303	2.24E-05	0.000447	0.000306	Cx3cl1/Cxcr4/Ets1/Gata2/Gpr4/Hpse/Ndnf/Pik3cb/Tnf	9
GO:0140353	lipid export from cell	14/1242	58/18303	2.45E-05	0.000488	0.000334	Acsl4/Agtr1a/C1qtnf1/Fzd4/Il1a/Il1b/Inhba/Nos2/Nrg1/P2ry2/Ptges/Myb/Tnf/Trpv4	14
GO:0046633	alpha-beta T cell proliferation	12/1242	44/18303	2.49E-05	0.000492	0.000336	Blm/Cd274/Cd44/Il15/Il23a/Irf1/Lgals9/Rasal3/Ripk2/Syk/Vsir/Xcl1	12
GO:0048246	macrophage chemotaxis	12/1242	44/18303	2.49E-05	0.000492	0.000336	Ccl2/Ccl3/Ccl5/Cmklr1/Csf1/Cx3cl1/Ednrb/Lgals3/Rarres2/Thbs1/Tnfsf18/Trpv4	12
GO:0072073	kidney epithelium development	26/1242	159/18303	2.51E-05	0.000493	0.000337	Agtr1a/Aqp1/Bdnf/Bmp2/Bmp4/Bmp7/Bmper/Cd44/Dll1/Epha4/Fat4/Fgfr2/Foxc1/Hnf1b/Irx3/Lif/Mef2c/Npnt/Pax8/Pdgfb/Pgf/Ptpro/Sfrp1/Slit2/Wnk4/Wnt2b	26
GO:0050919	negative chemotaxis	13/1242	51/18303	2.55E-05	0.0005	0.000342	Flrt3/Itgb3/Nrg1/Plxna3/Sema3f/Sema4a/Sema4b/Sema5a/Sema6a/Sema6d/Slit2/Slit3/Wnt5a	13
GO:0001660	fever generation	7/1242	15/18303	2.58E-05	0.000505	0.000345	Ccl5/Ednrb/Il1a/Il1b/Il1rn/Ptgs2/Tnf	7
GO:0009110	vitamin biosynthetic process	8/1242	20/18303	2.65E-05	0.000516	0.000353	Cyp27a1/Cyp27b1/Il1b/Nfkb1/Pltp/Snai1/Snai2/Tnf	8
GO:0060389	pathway-restricted SMAD protein phosphorylation	15/1242	66/18303	2.75E-05	0.000533	0.000364	Bmp2/Bmp3/Bmp4/Bmp5/Bmp7/Bmper/Emilin1/Gdf15/Gdf6/Hfe/Inha/Inhba/Inhbe/Lgals9/Rbpms	15
GO:0034329	cell junction assembly	53/1242	437/18303	2.78E-05	0.000539	0.000368	Ank2/Bdnf/Bsn/Cadm1/Cldn3/Clstn2/Cntn5/Col17a1/Coro2b/Ctnnd2/Dnm3/Drd1/Enpp2/Ephb2/Ephb3/Erc2/F11r/Flcn/Flot1/Flrt3/Fscn1/Fzd5/Gja5/Gjb2/Il1b/Itga2/Itga5/Itgb3/Limch1/Lrfn1/Mef2c/Nectin1/Nptxr/Nrg1/Nrxn3/Obsl1/Pard6b/Pclo/Pdgfb/Plxnd1/Pmp22/Ptpro/Ramp2/Rap1b/Sema4a/Shank1/Snai1/Snai2/Thbs2/Tlr2/Tn	53
GO:0031668	cellular response to extracellular stimulus	39/1242	288/18303	2.83E-05	0.000545	0.000373	Acat2/Asgr1/Atf3/Bmf/Ccl5/Cd68/Col1a1/Col2a1/Cpeb4/Cyp11b2/Cyp27b1/Flcn/Fnip1/Fosl1/Fscn1/Fzd2/Gstp1/Hfe/Il15/Jun/Mmp1/Mmp3/Mmp9/Nampt/Nos2/Pcsk9/Penk/Pik3c2b/Ppp1r15a/Rragd/Sesn3/Sfrp1/Slc1a2/Snai2/Tfeb/Ucn2/Upp1/Vcam1/Wnt2b	39
GO:0042542	response to hydrogen peroxide	30/1242	198/18303	2.84E-05	0.000545	0.000373	Ada/Akr1b1/Aqp1/Areg/Casp12/Cfh/Col1a1/Cyp1b1/Ern1/Ets1/F3/Fosl1/Gpr37/Gpr37l1/Hk3/Il18rap/Jun/Klf2/Klf6/Net1/Nptxr/Nqo1/Olr1/Ppif/Pycr1/Myb/Sirpa/Star/Stat1/Tnfaip3	30
GO:1903035	negative regulation of response to wounding	18/1242	90/18303	2.89E-05	0.000554	0.000379	C1qtnf1/Cask/Cldn3/Dusp10/Epha4/Eppk1/Hmgcr/Pdgfb/Plau/Rgma/Serpinf2/Serping1/Siglec10/Thbd/Thbs1/Tnf/Wfdc1/Xylt1	18
GO:0006775	fat-soluble vitamin metabolic process	11/1242	38/18303	2.94E-05	0.00056	0.000383	Cyp26b1/Cyp27a1/Cyp27b1/Il1b/Nfkb1/Pltp/Ppard/Rbp1/Snai1/Snai2/Tnf	11
GO:0032651	regulation of interleukin-1 beta production	17/1242	82/18303	2.94E-05	0.00056	0.000383	Ccl3/Egr1/Errfi1/Fzd5/Gstp1/Lgals9/Nlrc4/Nlrp3/Nod2/Pycard/Inava/Ripk2/Sirpa/Tlr2/Tnf/Tnfaip3/Wnt5a	17
GO:2000377	regulation of reactive oxygen species metabolic process	32/1242	218/18303	2.97E-05	0.000564	0.000386	Abcd1/Agtr1a/Ass1/Birc3/Cflar/Cxcl1/Cyp1b1/Ddah2/Fbln5/Gch1/Gstp1/Hk2/Hvcn1/Ier3/Il1b/Acod1/Klf2/Mmp3/Nox1/Olr1/Pdgfb/Plau/Ptger4/Ptgs2/Sirpa/Slc5a3/Syk/Thbs1/Tlr2/Tlr5/Tlr6/Tnf	32
GO:0003170	heart valve development	14/1242	59/18303	3.02E-05	0.00057	0.00039	Bmp2/Bmp4/Efna1/Emilin1/Gja5/Hey2/Mef2c/Pde2a/Prdm1/Rbpj/Scx/Slit2/Slit3/Tnfrsf1b	14
GO:0090130	tissue migration	41/1242	310/18303	3.12E-05	0.000587	0.000401	Angpt4/Anxa3/Aqp1/Bmp4/Bmper/Cd40/Cyp1b1/Dcn/Dusp10/Efna1/Enpp2/Eppk1/Ets1/Flt4/Gata2/Has2/Hdac9/Hyal1/Itga2/Itgb3/Jun/Lpxn/Mef2c/Meox2/Mmp9/Pdgfb/Plk2/Plpp3/Plxnd1/Ppard/Ptgs2/Ptp4a3/Rgcc/Rhoj/Sema4a/Sema5a/Slit2/Thbs1/Tnf/Wnt5a/Zeb2	41

ID	Description	GeneRatio	BgRatio	pvalue	p.adjust	qvalue	geneID	Count
GO:0060537	muscle tissue development	56/1242	472/18303	3.13E-05	0.000587	0.000401	Ankrd1/Atf3/Bdnf/Bmp2/Bmp4/Bmp5/Bmp7/Btg2/Bves/Casq1/Cflar/Col11a1/Col14a1/Col3a1/Csrp2/Cyp26b1/Dcn/Dll1/Egr1/Ripor2/Fdps/Fgf9/Fgfr2/Flcn/Flot1/Foxc1/Gata6/Gja5/Gli1/Hdac9/Hey2/Hmgcr/Igfbp5/Itga7/Kcnk2/Maff/Mef2c/Meox2/Mylk/Ncam1/Nrg1/Pi16/Plagl1/Pmp22/Ppargc1a/Rbpj/Ryr2/Scx/Sgcd/Sgcg/Sorbs2/Tbx3/Tenm4/Tiparp/Wnt5a/Xirp1	56
GO:0034405	response to fluid shear stress	12/1242	45/18303	3.19E-05	0.000593	0.000406	Ass1/Csf2/Ets1/F3/Has2/Klf2/Mef2c/Mmp1/Mmp13/Plau/Ptgs2/Tfpi2	12
GO:0043123	positive regulation of I-kappaB kinase/NF-kappaB signaling	28/1242	180/18303	3.19E-05	0.000593	0.000406	Card11/Cd40/Cflar/Cth/Cx3cl1/Edar/Il1a/Il1b/Lgals9/Lurap1l/Map3k14/Nod2/Peli1/Plk2/Inava/Rel/Ripk2/Ror1/Slc20a1/Slc44a2/Tgm2/Tifa/Tlr3/Tlr6/Tnf/Traf5/Trim	28
GO:0032731	positive regulation of interleukin-1 beta production	13/1242	52/18303	3.19E-05	0.000593	0.000406	Ccl3/Egr1/Fzd5/Lgals9/Nlrc4/Nlrp3/Nod2/Pycard/Inava/Ripk2/Tlr2/Tnf/Wnt5a	13
GO:0072593	reactive oxygen species metabolic process	40/1242	300/18303	3.22E-05	0.000597	0.000408	Abcd1/Agtr1a/Ass1/Birc3/Cflar/Cxcl1/Cyp1b1/Ddah2/Fbln5/Gch1/Gstp1/Hk2/Hvcn1/Ier3/Il19/Il1b/Acod1/Klf2/LOC497963/Mmp3/Nos2/Nox1/Nqo1/Olr1/Pdgfb/Pla2r1/Plau/Ptger4/Ptgs2/Inava/Sirpa/Slc5a3/Slc7a2/Syk/Thbs1/Tlr2/Tlr5/Tlr6/Tnf/Txnrd1	40
GO:0001779	natural killer cell differentiation	10/1242	32/18303	3.24E-05	0.000599	0.00041	Flt3lg/Ikzf1/Il15/Il15ra/Lgals9/Pglyrp1/Pglyrp4/Prdm1/Rasgrp1/Tox	10
GO:0090092	regulation of transmembrane receptor protein serine/threonine kinase signaling pathway	35/1242	249/18303	3.28E-05	0.000604	0.000413	Bmp2/Bmp3/Bmp4/Bmp5/Bmp7/Bmper/Chrd/Dact2/Emilin1/Fgf9/Flcn/Furin/Gata6/Gdf15/Gdf6/Grem2/Hfe/Hipk2/Hspa1b/Htra3/Inha/Inhba/Inhbe/Lgals9/Npnt/Nrep/Peg10/Rbpj/Rbpms/Rgma/Sfrp1/Slc2a10/Thbs1/Vsir/Wnt5a	35
GO:0071692	protein localization to extracellular region	57/1242	484/18303	3.29E-05	0.000604	0.000413	A1cf/Aacs/Acsl4/Adcy8/Adora2a/Adra2a/Agtr1a/Ankrd1/C1qtnf3/Cadm1/Cask/Ccl5/Cd40/Clec4e/Cpt1a/Cx3cl1/Cyp51/Fgr/Frmd4a/Gja5/Gpr68/Hadh/Hdac9/Hmgcr/Hnf1b/Il1a/Il1b/Il1rn/Il33/Kcnn4/Mafa/Mmp13/Hcar2/Nlrp3/Nod2/Nos2/Pclo/Pcsk1/Pcsk5/Plek/Ppard/Ptger4/Rab11fip1/Rab3d/Rfx3/Rgcc/Rsad2/Sfrp1/Siglec10/Sybu/Syk/Tlr2/Tlr5/Tnf/Tnfsf15/Trem3/Vsnl1	57
GO:0150076	neuroinflammatory response	15/1242	67/18303	3.32E-05	0.000607	0.000415	Adcy8/Adora2a/Cd200r1/Cx3cl1/Il1b/Il33/Jun/Ldlr/Nampt/Ptgs2/Tlr2/Tlr3/Tlr6/Tnf/Tnfrsf1b	15
GO:0007264	small GTPase mediated signal transduction	52/1242	429/18303	3.36E-05	0.00061	0.000418	Adcyap1r1/Agtr1a/Als2/Arap3/Arhgdib/Arhgef25/Arhgef3/Cadm4/Cdc42ep5/Chn1/Col3a1/Csf1/Dhcr24/Dock4/Dock8/Ephb2/F11r/Ripor2/Flcn/Flot1/Gna13/Gpr4/Jun/Kitlg/LOC691418/Net1/Ngf/Nradd/Nrg1/Plk2/Psd3/RGD1560455/Rab30/Ralgapa2/Ralgps1/Rap1b/Rap1gap/Rapgef5/Rapgefl1/Rasa2/Rasal3/Rasgrp1/Rasgrp3/Rassf1/Rerg/Rhobtb1/Rhoj/Rhou/Sh2b2/Slit2/Spry4/Srgap1	52
GO:0007265	Ras protein signal transduction	43/1242	332/18303	3.36E-05	0.00061	0.000418	Agtr1a/Als2/Arap3/Arhgdib/Arhgef25/Arhgef3/Cadm4/Cdc42ep5/Col3a1/Csf1/Dhcr24/Ephb2/F11r/Ripor2/Flcn/Flot1/Gna13/Gpr4/Jun/Kitlg/LOC691418/Net1/Ngf/Nradd/Nrg1/Plk2/Psd3/RGD1560455/Rab30/Ralgapa2/Ralgps1/Rap1b/Rasa2/Rasal3/Rasgrp1/Rasgrp3/Rassf1/Rerg/Rhoj/Rhou/Sh2b2/Spry4/Srgap1	43
GO:0035690	cellular response to drug	20/1242	108/18303	3.46E-05	0.000625	0.000427	Ankrd1/Blm/Ccl2/Cd69/Cxcr4/Egr1/Hmga2/Il1b/Itgb3/Kcnh2/Mef2c/Mmp1/Mylk/Nos2/Pde2a/Pde4b/Ppp1r15a/Rap1b/Rnf149/Tlr3	20
GO:0046634	regulation of alpha-beta T cell activation	20/1242	108/18303	3.46E-05	0.000625	0.000427	Ada/Adora2a/Blm/Cd274/Cd44/Cd83/Hfe/Ikzf1/Il23a/Irf1/Lgals9/Nlrp3/Prdm1/Myb/Rasal3/Ripk2/Syk/Tnfsf18/Vsir/Xcl1	20
GO:0007409	axonogenesis	57/1242	485/18303	3.49E-05	0.000629	0.00043	Ablim1/Alcam/Als2/Ank3/Bdnf/Bmp7/Cdh4/Chn1/Cobl/Col25a1/Cxcl12/Cxcr4/Efna1/Epha4/Ephb2/Ephb3/Etv1/Fgfr2/Flot1/Flrt3/Gas1/Gbx2/Lama2/Lmtk2/Map6/Matn2/Metrn/Mgll/Ncam1/Nectin1/Nefh/Ngf/Nrg1/Plxna2/Plxna3/Plxnd1/Pmp22/Prdm8/Ptpro/Rgma/Sema3f/Sema4a/Sema4b/Sema5a/Sema6a/Sema6d/Slit2/Slit3/Sptbn4/Tnn/Tubb2b/Tubb3/Twf2/Vim/Wnt5a/Zeb2/Zswim5	57
GO:0007187	G protein-coupled receptor signaling pathway, coupled to cyclic nucleotide second messenger	35/1242	250/18303	3.57E-05	0.000641	0.000439	Adcy8/Adcyap1r1/Adgrd1/Adora1/Adora2a/Adra2a/Akap12/Arrdc3/Calcrl/Cxcl10/Cxcl11/Drd1/Fzd2/Gna13/Gna14/Gpr37/Gpr37l1/Gpr4/Htr2a/Htr7/Itgb3/Lgr5/Nos2/Npr3/Palm/Pde2a/Pde4b/Ptger1/Ptger4/Ptgir/Pth1r/Ramp2/Tshr/Ucn2/Vipr1	35
GO:0019218	regulation of steroid metabolic process	21/1242	117/18303	3.62E-05	0.000648	0.000443	Agtr1a/Bmp2/Bmp5/Cyp27b1/Cyp51/Egr1/Fdps/Fmo5/Il1a/Il1b/Insig1/Ldlr/Nfkb1/Ppargc1a/Snai1/Snai2/Star/Stard4/Thrb/Tnf/Ttc39b	21
GO:0034113	heterotypic cell-cell adhesion	14/1242	60/18303	3.69E-05	0.000659	0.000451	Alox15/Bmp7/Cd200r1/Cd44/Flot1/Il1b/Il1rn/Itga5/Itga7/Itgax/Itgb3/Sirpa/Tnf/Vcam1	14
GO:0071774	response to fibroblast growth factor	22/1242	126/18303	3.70E-05	0.000659	0.000451	Ccl2/Ccl5/Cd44/Col1a1/Col2a1/Creb3l1/Fam20c/Fat4/Fgf9/Fgfr2/Flrt3/Fuz/Gclc/Hyal1/Pdgfb/Sfrp1/Snai2/Spry4/Star/Thbs1/Vim/Wnt5a	22

ID	Description	GeneRatio	BgRatio	pvalue	p.adjust	qvalue	geneID	Count
GO:0009914	hormone transport	48/1242	387/18303	3.72E-05	0.00066	0.000451	Aacs/Abcb1a/Acsl4/Adcy8/Adora1/Adra2a/Agtr1a/Aqp1/C1qtnf1/C1qtnf3/Cask/Ccl5/Cpt1a/Fzd4/Gpr68/Hadh/Hfe/Hmga2/Hmgcr/Hnf1b/Il11/Il1b/Il1rn/Inha/Inhba/Lif/Ltbp4/Mafa/Hcar2/Nos2/Npy1r/Nrg1/Pax8/Pclo/Ppard/Ptger4/Myb/Rab11fip1/Rfx3/Sfrp1/Slc7a8/Slco4a1/Sybu/Tbx3/Tnf/Trpv4/Ucn2/Vsnl1	48
GO:0007611	learning or memory	42/1242	323/18303	3.76E-05	0.000665	0.000455	Adcy8/Bdnf/Btg2/Csmd1/Ctnnd2/Cyp7b1/Drd1/Egr1/Elavl4/Ephb2/Fosl1/Gpr88/Grin2a/Hmgcr/Htr2a/Htr7/Il1b/Il1rn/Itga5/Jun/Kcnk2/Ldlr/Lrrn4/Mef2c/Ncam1/Ngf/Nrg1/Nrxn3/Pde1b/Plcb1/Plk2/Prkar2b/Prrt1/Ptgs2/Rcan2/Rgs14/Sgk1/Shank1/Slc7a11/Thra/Tlr2/Tmod2	42
GO:0050870	positive regulation of T cell activation	30/1242	201/18303	3.79E-05	0.000666	0.000456	Ada/Blm/Card11/Ccl2/Ccl5/Cd274/Cd83/Coro1a/Dock8/Dusp10/Ikzf1/Il15/Il1a/Il1b/Il23a/Itgal/Lgals9/Lilrb4/Nlrp3/Pycard/Myb/Rasal3/Rasgrp1/Ripk2/Sirpa/Sox12/Syk/Vcam1/Vsir/Xcl1	30
GO:0072330	monocarboxylic acid biosynthetic process	30/1242	201/18303	3.79E-05	0.000666	0.000456	Abcd1/Acsl3/Acss1/Alox15/Bdkrb1/Chst14/Cyp27a1/Cyp7b1/Elovl4/Fa2h/Fabp5/Fasn/Gamt/Gstp1/Hacd4/Hoga1/Il1b/Insig1/Lipg/Liph/Mgll/Olah/Prkab2/Ptges/Ptgs2/Rbp1/Scd/Star/Stard4/Thnsl2	30
GO:0010573	vascular endothelial growth factor production	11/1242	39/18303	3.84E-05	0.000673	0.00046	Ccl2/Cyp1b1/Flt1/Flt4/Hpse/Il1a/Il1b/Ndrg2/Nox1/Ptgs2/Tnf	11
GO:2000027	regulation of animal organ morphogenesis	31/1242	211/18303	3.87E-05	0.000675	0.000462	Agtr1a/Ankrd6/Bdnf/Bmp2/Bmp4/Bmp7/Cflar/Csf1/Cthrc1/Fgfr2/Foxc1/Fzd2/Gli1/Hand2/Hmga2/Hnf1b/Lif/Ntn4/Pax8/Pax9/Plau/Jhy/Rbpj/Sfrp1/Snai2/Thrb/Tnf/Tnfaip3/Tnfrsf11b/Wnt2b/Wnt5a	31
GO:0032944	regulation of mononuclear cell proliferation	34/1242	241/18303	3.92E-05	0.00068	0.000465	Ada/Blm/Bmp4/Card11/Ccl5/Cd274/Cd40/Cd44/Clcf1/Coro1a/Csf1/Ripor2/Flt3lg/Il15/Il1a/Il1b/Il23a/Irf1/Itgal/Lgals3/Lgals9/Mef2c/Peli1/Prdm1/Pycard/Rasal3/Ripk2/Siglec10/Syk/Tnfrsf1b/Tnfsf18/Vcam1/Vsir/Xcl1	34
GO:0071248	cellular response to metal ion	34/1242	241/18303	3.92E-05	0.00068	0.000465	A3galt2/Adcy8/Adgrv1/Alox15/Ank3/Aqp1/Atp7b/Casp12/Cpne4/Cpne8/Cyp11b2/Enpp2/Ern1/Fabp4/Glra1/Grin2a/Hfe/Hvcn1/Jun/Junb/Kcnh1/Kcnk3/Mef2c/Mmp1/Mmp9/Mylk/Nos2/Nqo1/Ppargc1a/Ppif/Ptgs2/Star/Txnr	34
GO:0014033	neural crest cell differentiation	18/1242	92/18303	3.94E-05	0.000682	0.000466	Bmp4/Bmp7/Ednrb/Foxc1/Frzb/Gbx2/Hand2/Kitlg/Mef2c/Sema3f/Sema4a/Sema4b/Sema5a/Sema6a/Sema6d/Sfrp1/Snai2/Zeb2	18
GO:0048638	regulation of developmental growth	50/1242	410/18303	4.03E-05	0.000696	0.000476	Bdnf/Bmp4/Cdh4/Col14a1/Csf1/Csf2/Cxcl12/Cxcr4/Dll1/Dusp10/Dusp6/Eppk1/Fdps/Fgf9/Fgfr2/Foxc1/Gamt/Gata6/Gdf15/Gli1/Hey2/Hmga2/Hnf1b/Ikzf1/Kcnk2/Mef2c/Mgll/Ncam1/Ngf/Npy1r/Nrg1/Pi16/Plcb1/Plxna3/Ppard/Ptger4/Rbpj/Rgma/Sema3f/Sema4a/Sema4b/Sema5a/Sema6a/Sema6d/Sfrp1/Slit2/Sptbn4/Tshr/Twf2/Wnt5a	50
GO:0060401	cytosolic calcium ion transport	29/1242	192/18303	4.06E-05	0.000699	0.000478	Adcyap1r1/Agtr1a/Ank2/Atp2a3/Bdkrb1/Bmp4/Cacna1g/Casq1/Ccl3/Cemip/Coro1a/Cx3cl1/Cxcl10/Cxcl11/Drd1/Grin2a/Htr2a/Itgb3/Mcoln2/Mcoln3/Ngf/P2ry6/Rgs9/Ryr2/Ryr3/Slc35g1/Trpv3/Trpv4/Xcl1	29
GO:0031640	killing of cells of other organism	17/1242	84/18303	4.08E-05	0.0007	0.000479	Camp/Ccl20/Ccl22/Cxcl1/Cxcl10/Cxcl11/Cxcl12/Cxcl2/Cxcl3/Lgals3/Lyz2/Nos2/Pglyrp1/Pglyrp4/Syk/Trem3/	17
GO:0044344	cellular response to fibroblast growth factor stimulus	21/1242	118/18303	4.12E-05	0.000705	0.000482	Ccl2/Ccl5/Cd44/Col1a1/Creb3l1/Fam20c/Fat4/Fgf9/Fgfr2/Flrt3/Fuz/Gclc/Hyal1/Pdgfb/Sfrp1/Snai2/Spry4/Star/Thbs1/Vim/Wnt5a	21
GO:0002274	myeloid leukocyte activation	34/1242	242/18303	4.27E-05	0.000728	0.000498	Anxa3/Ccl3/Ccl5/Csf2/Cx3cl1/Fgr/Flt3lg/Gata2/Il13ra2/Il15/Il18rap/Il33/Jun/Lbp/Ldlr/Lgals9/Myo1f/Nampt/Pla2g2a/Pla2g5/Ptpre/Pycard/Rasgrp1/Rbpj/Relb/Slc7a2/Syk/Thbs1/Tlr2/Tlr3/Tlr6/Tnf/Tslp/Wnt5a	34
GO:0030656	regulation of vitamin metabolic process	7/1242	16/18303	4.32E-05	0.000733	0.000501	Clybl/Cyp27b1/Il1b/Nfkb1/Snai1/Snai2/Tnf	7
GO:1901722	regulation of cell proliferation involved in kidney	7/1242	16/18303	4.32E-05	0.000733	0.000501	Bmp4/Bmp7/Cflar/Egr1/Flcn/Itgb3/Pdgfb	7
GO:0046683	response to organophosphorus	29/1242	193/18303	4.47E-05	0.000756	0.000517	Abcc9/Aqp1/Areg/Ass1/Atp7b/Ccl2/Col1a1/Cyp1b1/Cyp27b1/Cyp7b1/Egr1/Fosl1/Gata6/Hcn1/Igfbp5/Il1b/Jun/Junb/P2ry6/Pde2a/Penk/Pla2g5/Plat/Ptgs2/Rap1b/Serpina3n/Star/Stat1/Thbd	29
GO:0032732	positive regulation of interleukin-1 production	14/1242	61/18303	4.50E-05	0.000756	0.000517	Ccl20/Ccl3/Egr1/Fzd5/Lgals9/Nlrc4/Nlrp3/Nod2/Pycard/Inava/Ripk2/Tlr2/Tnf/Wnt5a	14
GO:0090183	regulation of kidney development	14/1242	61/18303	4.50E-05	0.000756	0.000517	Agtr1a/Bmp4/Bmp7/Cflar/Egr1/Fat4/Flcn/Hnf1b/Itgb3/Lif/Mmp9/Pax8/Pdgfb/Wnt2b	14
GO:2000106	regulation of leukocyte apoptotic process	20/1242	110/18303	4.55E-05	0.000762	0.000521	Ada/Blm/Bmp4/Ccl5/Cd274/Cd44/Cxcl12/Dock8/Efna1/Fnip1/Kitlg/Lgals3/Lgals9/Mef2c/Hcar2/Nod2/Pik3cb/Serpinb9/Slc7a11/Wnt5a	20
GO:0006631	fatty acid metabolic process	50/1242	412/18303	4.58E-05	0.000764	0.000523	Aacs/Abcd1/Acat2/Acat2l1/Acot11/Acsl1/Acsl3/Acsl4/Acss1/Alox15/Bdkrb1/Cpt1a/Cpt1b/Cyp1b1/Cyp2j4/Ech1/Echdc2/Elovl4/Erfe/Fa2h/Fabp3/Fabp4/Fabp5/Fasn/Ggt1/Gsta2/Gstp1/Hacd4/Hadh/Hpgd/Il1b/Insig1/Lipg/Liph/Mgll/Olah/Phyh/Pla2g5/Pnpla3/Ppard/Ppargc1a/Prkab2/Prkar2b/Ptges/Ptgr1/Ptgs2/Sc5d/Scd/Slc27a3/Thns	50

ID	Description	GeneRatio	BgRatio	pvalue	p.adjust	qvalue	geneID	Count
GO:0007188	adenylate cyclase-modulating G protein-coupled receptor signaling	32/1242	223/18303	4.68E-05	0.000779	0.000533	Adcy8/Adcyap1r1/Adgrd1/Adora1/Adora2a/Adra2a/Akap12/Arrdc3/Calcrl/Cxcl10/Cxcl11/Drd1/Gna13/Gna14/Gpr37/Gpr37l1/Gpr4/Htr7/Itgb3/Lgr5/Npr3/Palm/Pde2a/Pde4b/Ptger1/Ptger4/Ptgir/Pth1r/Ramp2/Tshr/Ucn2/Vip	32
GO:0001935	endothelial cell proliferation	25/1242	156/18303	5.03E-05	0.000835	0.000571	Agtr1a/Bmp2/Bmp4/Bmper/Ccl2/Cxcl12/Ern1/F3/Flt1/Flt4/Gata2/Itgb3/Jun/Mef2c/Nos2/Pdgfb/Pgf/Pik3cb/Rgcc/Sema5a/Stat1/Thbs1/Thbs4/Tnf/Wnt5a	25
GO:0032946	positive regulation of mononuclear cell proliferation	24/1242	147/18303	5.20E-05	0.000861	0.000589	Ada/Blm/Card11/Ccl5/Cd274/Cd40/Clcf1/Coro1a/Csf1/Flt3lg/Il15/Il1a/Il1b/Il23a/Itgal/Lgals9/Mef2c/Peli1/Pycard/Rasal3/Ripk2/Syk/Vcam1/Xcl1	24
GO:0035296	regulation of tube diameter	28/1242	185/18303	5.27E-05	0.000868	0.000594	Adora1/Adora2a/Adra2a/Agtr1a/Bdkrb2/Cacna1g/Cx3cl1/Dock4/Drd1/Dusp5/Ednrb/Foxc1/Gch1/Gclc/Gja5/Hmgcr/Hspa1b/Htr2a/Htr7/Mgll/Npy1r/Olr1/P2ry2/Ppard/Ptgs2/Serpinf2/Tnf/Trpv4	28
GO:0097746	regulation of blood vessel diameter	28/1242	185/18303	5.27E-05	0.000868	0.000594	Adora1/Adora2a/Adra2a/Agtr1a/Bdkrb2/Cacna1g/Cx3cl1/Dock4/Drd1/Dusp5/Ednrb/Foxc1/Gch1/Gclc/Gja5/Hmgcr/Hspa1b/Htr2a/Htr7/Mgll/Npy1r/Olr1/P2ry2/Ppard/Ptgs2/Serpinf2/Tnf/Trpv4	28
GO:0010830	regulation of myotube	14/1242	62/18303	5.45E-05	0.000891	0.000609	Bdnf/Cd53/Cflar/Cxcl10/Cxcl12/Cyp26b1/Ehd2/Ripor2/Flot1/Flt3lg/Gdf15/Hdac9/Rbm38/Thra	14
GO:0006816	calcium ion transport	53/1242	448/18303	5.45E-05	0.000891	0.000609	Adcyap1r1/Adora2a/Adra2a/Agtr1a/Ank2/Atp2a3/Bdkrb1/Bmp4/Cacna1g/Cacnb2/Calcrl/Cask/Casq1/Ccl2/Ccl3/Ccl5/Ccr1/Cemip/Coro1a/Cx3cl1/Cxcl10/Cxcl11/Cxcl12/Cxcr4/Cyp27b1/Drd1/Grin2a/Htr2a/Itgb3/Kcnn4/Lgals3/Mcoln2/Mcoln3/Mylk/Nalcn/Ngf/P2ry6/Pde4b/Pdgfb/Ptgs2/Myb/Ramp2/Rgs9/Ryr2/Ryr3/Slc24a3/Slc24a4/Slc25a25/Slc35g1/Trpc4/Trpv3/Trpv4/Xcl1	53
GO:1901653	cellular response to peptide	53/1242	448/18303	5.45E-05	0.000891	0.000609	Adcy8/Agtr1a/Akr1b1/Ass1/Ccl2/Cflar/Col2a1/Cpeb2/Cyp11b2/Cyp1b1/Dnai1/Drd1/Egr1/Epha4/Erfe/Errfi1/Fzd2/Gclc/Gdf15/Gjb2/Gstp1/Hdac9/Il1b/Insig1/Irf1/Irs3/Itgb3/Klf2/Klf6/Mmp1/Nampt/Nfkb1/Nod2/Pcsk9/Pdgfb/Plat/Plcb1/Pnpla3/Ptpre/Rap1b/Rarres2/Ripk2/Sesn3/Sgk1/Sh2b2/Socs3/Star/Stat1/Tlr6/Tnf/Tshr/Vcam1/Vi	53
GO:0071453	cellular response to oxygen levels	32/1242	225/18303	5.58E-05	0.000908	0.000621	Angpt4/Ankrd1/Aqp1/Bach1/Bdkrb2/Cflar/Cox4i2/Cpeb2/Cpeb4/Drd1/Egln3/Egr1/Flt1/Fndc1/Gata6/Hilpda/Kcnk2/Kcnk3/Nampt/Ndnf/Nox1/Plau/Ppard/Ppargc1a/Ppp1r15a/Ptgs2/Rbpj/Rgcc/Sfrp1/Txnrd1/Uck2/Ucn2	32
GO:0035150	regulation of tube size	28/1242	186/18303	5.81E-05	0.000939	0.000642	Adora1/Adora2a/Adra2a/Agtr1a/Bdkrb2/Cacna1g/Cx3cl1/Dock4/Drd1/Dusp5/Ednrb/Foxc1/Gch1/Gclc/Gja5/Hmgcr/Hspa1b/Htr2a/Htr7/Mgll/Npy1r/Olr1/P2ry2/Ppard/Ptgs2/Serpinf2/Tnf/Trpv4	28
GO:0010574	regulation of vascular endothelial growth factor production	10/1242	34/18303	5.82E-05	0.000939	0.000642	Ccl2/Cyp1b1/Flt1/Flt4/Hpse/Il1a/Il1b/Ndrg2/Nox1/Ptgs2	10
GO:0060142	regulation of syncytium formation by plasma membrane fusion	10/1242	34/18303	5.82E-05	0.000939	0.000642	Cd53/Cflar/Cxcl10/Cxcl12/Ehd2/Ripor2/Flot1/Flt3lg/Gdf15/Stat1	10
GO:0072111	cell proliferation involved in kidney development	8/1242	22/18303	5.95E-05	0.000958	0.000655	Bmp2/Bmp4/Bmp7/Cflar/Egr1/Flcn/Itgb3/Pdgfb	8
GO:0010001	glial cell differentiation	37/1242	277/18303	6.04E-05	0.000968	0.000662	Adora2a/Aspa/Bmp2/Bmp4/Clcf1/Cspg5/Cxcr4/Dll1/Drd1/Dusp10/Egr1/Enpp2/Epha4/Fa2h/Gpr37l1/Gstp1/Hmga2/Il1b/Lama2/Ldlr/Lif/Metrn/Mxra8/Myrf/Nfix/Nrg1/Pdgfb/Plpp3/Pmp22/Prdm8/Ror1/Sh3tc2/Tenm4/Tlr2/Tnfrsf1b/Trpc4/Vim	37
GO:0006939	smooth muscle contraction	21/1242	121/18303	6.04E-05	0.000968	0.000662	Ada/Adora1/Adra2a/Bdkrb1/Bdkrb2/Cacna1g/Calcrl/Dock4/Drd1/Ednrb/Flt1/Htr2a/Htr7/Itga2/Mylk/Npnt/Npy1r/Ptger4/Ptgs2/Tbx3/Trpv4	21
GO:0070665	positive regulation of leukocyte proliferation	25/1242	158/18303	6.23E-05	0.000993	0.000679	Ada/Blm/Card11/Ccl5/Cd274/Cd40/Clcf1/Coro1a/Csf1/Flt3lg/Il15/Il1a/Il1b/Il23a/Itgal/Kitlg/Lgals9/Mef2c/Peli1/Pycard/Rasal3/Ripk2/Syk/Vcam1/Xcl1	25
GO:0072124	regulation of glomerular mesangial cell proliferation	6/1242	12/18303	6.24E-05	0.000993	0.000679	Bmp4/Bmp7/Cflar/Egr1/Itgb3/Pdgfb	6
GO:0045662	negative regulation of myoblast differentiation	9/1242	28/18303	6.29E-05	0.000993	0.000679	Bmp4/Cxcl10/Dll1/Mbnl3/Mkx/Ppard/Prickle1/Tbx3/Tnf	9
GO:0060045	positive regulation of cardiac muscle cell proliferation	9/1242	28/18303	6.29E-05	0.000993	0.000679	Fgf9/Fgfr2/Gata6/Gli1/Hey2/Mef2c/Ncam1/Nrg1/Rbpj	9
GO:0060445	branching involved in salivary gland morphogenesis	9/1242	28/18303	6.29E-05	0.000993	0.000679	Bmp7/Esrp2/Fgfr2/Ntn4/Pdgfb/Plxnd1/Snai2/Tgm2/Tnf	9
GO:0046640	regulation of alpha-beta T cell proliferation	11/1242	41/18303	6.39E-05	0.001004	0.000687	Blm/Cd274/Cd44/Il23a/Irf1/Lgals9/Rasal3/Ripk2/Syk/Vsir/Xcl1	11
GO:1902667	regulation of axon guidance	12/1242	48/18303	6.39E-05	0.001004	0.000687	Cxcl12/Plxna3/Sema3f/Sema4a/Sema4b/Sema5a/Sema6a/Sema6d/Slit2/Tubb2b/Wnt5a/Zswim5	12

ID	Description	GeneRatio	BgRatio	pvalue	p.adjust	qvalue	geneID	Count
GO:0051591	response to cAMP	24/1242	149/18303	6.49E-05	0.001016	0.000695	Aqp1/Areg/Ass1/Atp7b/Col1a1/Cyp1b1/Cyp27b1/Cyp7b1/Egr1/Fosl1/Gata6/Hcn1/Igfbp5/Jun/Junb/Pde2a/Penk/Pla2g5/Plat/Rap1b/Serpina3n/Star/Stat1/Thbd	24
GO:0010769	regulation of cell morphogenesis involved in differentiation	45/1242	363/18303	6.52E-05	0.001017	0.000695	Bdnf/Cask/Cdh4/Chn1/Cspg5/Cxcl12/Dnm3/Efna1/Enpp2/Epha4/Ephb2/Ephb3/Fbln1/Has2/Itgb3/Kif1a/Lrp8/Map6/Meltf/Metrn/Mgll/Net1/Ngf/Nrg1/Obsl1/Plxna2/Plxna3/Plxnd1/Rgma/Sema3f/Sema4a/Sema4b/Sema5a/Sema6a/Sema6d/Sgk1/Slit2/Tnik/Tubb2b/Twf2/Vim/Wnt5a/Zdhhc15/Zeb2/Zswim5	45
GO:0035710	CD4-positive, alpha-beta T cell activation	19/1242	104/18303	6.53E-05	0.001017	0.000695	Bcl3/Cd274/Cd44/Cd83/Il23a/Lgals9/Nlrp3/Pax1/Ptger4/Myb/Relb/Ripk2/Rsad2/Satb1/Sema4a/Tnfsf18/Tox/Vsir/Xcl1	19
GO:0042098	T cell proliferation	31/1242	217/18303	6.65E-05	0.001033	0.000707	Blm/Bmp4/Card11/Ccl5/Cd274/Cd44/Coro1a/Cxcl12/Cxcr4/Dock8/Ripor2/Il15/Il1a/Il1b/Il23a/Irf1/Itgal/Lgals3/Lgals9/Peli1/Pycard/Rasal3/Rasgrp1/Ripk2/Satb1/Syk/Tnfrsf1b/Tnfsf18/Vcam1/Vsir/Xcl1	31
GO:0015909	long-chain fatty acid transport	15/1242	71/18303	6.79E-05	0.00105	0.000718	Abcd1/Acsl1/Acsl3/Bdkrb2/Cpt1b/Fabp3/Fabp4/Fabp5/Pla2g2a/Pla2g5/Pla2r1/Plin2/Slc27a3/Syk/Thbs1	15
GO:0050918	positive chemotaxis	15/1242	71/18303	6.79E-05	0.00105	0.000718	Bmp4/Ccl3/Ccl5/Ccl9/Coro1a/Cx3cl1/Cxcl10/Cxcl12/Vegfd/Itga2/Lgals3/Pdgfb/Pgf/Sema5a/Wnt5a	15
GO:0042359	vitamin D metabolic process	7/1242	17/18303	6.92E-05	0.001063	0.000727	Cyp27a1/Cyp27b1/Il1b/Nfkb1/Snai1/Snai2/Tnf	7
GO:0072672	neutrophil extravasation	7/1242	17/18303	6.92E-05	0.001063	0.000727	Cd99l2/Ripor2/Il1a/Il1r1/Jaml/Ptger4/Trem3	7
GO:0060021	roof of mouth development	18/1242	96/18303	7.09E-05	0.001086	0.000743	Col2a1/Csrnp1/Dhrs3/Ephb2/Ephb3/Fuz/Fzd2/Gas1/Hand2/Inhba/Insig1/Mef2c/Meox2/Snai1/Snai2/Tbx3/Tiparp/Wnt5a	18
GO:0050670	regulation of lymphocyte proliferation	33/1242	238/18303	7.15E-05	0.001093	0.000747	Ada/Blm/Bmp4/Card11/Ccl5/Cd274/Cd40/Cd44/Clcf1/Coro1a/Ripor2/Flt3lg/Il15/Il1a/Il1b/Il23a/Irf1/Itgal/Lgals3/Lgals9/Mef2c/Peli1/Prdm1/Pycard/Rasal3/Ripk2/Siglec10/Syk/Tnfrsf1b/Tnfsf18/Vcam1/Vsir/Xcl1	33
GO:0001906	cell killing	31/1242	218/18303	7.26E-05	0.001107	0.000757	Cadm1/Camp/Ccl2/Ccl20/Ccl22/Cfh/Coro1a/Cxcl1/Cxcl10/Cxcl11/Cxcl12/Cxcl2/Cxcl3/Hspa1b/Il18rap/Il23a/Lgals3/Lgals9/Lyz2/Mr1/Nos2/Pvr/Pglyrp1/Pglyrp4/Rasgrp1/Rnf19b/Serpinb3a/Serpinb9/Syk/Trem3/Xcl1	31
GO:0010594	regulation of endothelial cell migration	26/1242	169/18303	7.28E-05	0.001108	0.000758	Angpt4/Anxa3/Bmp4/Bmper/Cd40/Dcn/Efna1/Ets1/Flt4/Gata2/Hdac9/Itgb3/Mef2c/Meox2/Pdgfb/Plk2/Plpp3/Ptgs2/Rgcc/Rhoj/Sema4a/Sema5a/Slit2/Thbs1/Tnf/Wnt5a	26
GO:0032368	regulation of lipid transport	23/1242	141/18303	7.49E-05	0.001136	0.000777	Acat2/Acsl4/Agtr1a/C1qtnf1/Erfe/Fabp3/Il1a/Il1b/Itgb3/Lipg/Nfkbia/Nrg1/P2ry2/Pcsk9/Pla2r1/Pltp/Ptges/Myb/Syk/Thbs1/Tnf/Trpv4/Ttc39b	23
GO:1905952	regulation of lipid localization	27/1242	179/18303	7.56E-05	0.001143	0.000782	Acat2/Acsl4/Agtr1a/C1qtnf1/Cpt1a/Erfe/Fabp3/Hilpda/Il1a/Il1b/Itgb3/Lipg/Nfkbia/Nrg1/P2ry2/Pcsk9/Pla2r1/Plin2/Pltp/Ppard/Ptges/Myb/Syk/Thbs1/Tnf/Trpv4/Ttc39b	27
GO:0032943	mononuclear cell proliferation	40/1242	312/18303	7.81E-05	0.001179	0.000806	Ada/Blm/Bmp4/Card11/Ccl5/Cd180/Cd274/Cd40/Cd44/Clcf1/Coro1a/Csf1/Cxcl12/Cxcr4/Dock8/Ripor2/Flt3lg/Il15/Il1a/Il1b/Il23a/Irf1/Itgal/Lgals3/Lgals9/Mef2c/Peli1/Prdm1/Pycard/Rasal3/Rasgrp1/Ripk2/Satb1/Siglec10/Syk/Tnfrsf1b/Tnfsf18/Vcam1/Vsir/Xcl1	40
GO:0007596	blood coagulation	27/1242	180/18303	8.33E-05	0.001253	0.000857	Adra2a/Anxa8/C1qtnf1/Cfh/Cx3cl1/Ephb2/F11r/F13a1/F3/Fbln1/Gna13/Hpse/Itgb3/Mmrn1/Pdgfb/Pik3cb/Plau/Plek/Procr/Selp/Serpinf2/Serping1/Slc7a11/Syk/Tfpi2/Thbd/Thbs1	27
GO:0030856	regulation of epithelial cell differentiation	25/1242	161/18303	8.53E-05	0.001275	0.000872	Abcb1a/Bmp4/Cyp27b1/Dll1/Eppk1/Errfi1/F11r/Fat4/Foxc1/Foxn1/Frzb/Macroh2a2/Hey2/Il1a/Il1b/Lif/Maff/Mmp9/Mycl/Pax8/Rfx3/Tbx3/Tmem100/Tnf/Zeb2	25
GO:1902668	negative regulation of axon guidance	9/1242	29/18303	8.57E-05	0.001275	0.000872	Plxna3/Sema3f/Sema4a/Sema4b/Sema5a/Sema6a/Sema6d/Slit2/Wnt5a	9
GO:0010818	T cell chemotaxis	8/1242	23/18303	8.58E-05	0.001275	0.000872	Ccl3/Ccl5/Cxcl10/Cxcl11/Cxcl12/Cxcl16/Wnt5a/Xcl1	8
GO:0045986	negative regulation of smooth muscle contraction	8/1242	23/18303	8.58E-05	0.001275	0.000872	Adora1/Adra2a/Bdkrb1/Calcrl/Dock4/Ptger4/Ptgs2/Trpv4	8
GO:0051770	positive regulation of nitric-oxide synthase biosynthetic process	8/1242	23/18303	8.58E-05	0.001275	0.000872	Akap12/Ccl2/Ccl20/Il33/Nampt/Nod2/Stat1/Tlr2	8
GO:0070542	response to fatty acid	22/1242	133/18303	8.60E-05	0.001275	0.000872	Aacs/Acat2/Acsl1/Ass1/Ccl2/Cpt1a/Cyp27b1/Fabp3/Fdps/Il1b/Insig1/Ldlr/P2ry6/Pcsk1/Plat/Plcb1/Ppargc1a/Ptger4/Ptgs2/Scd/Sfrp1/Tlr2	22
GO:0030199	collagen fibril organization	13/1242	57/18303	8.96E-05	0.001319	0.000902	Col11a1/Col13a1/Col14a1/Col1a1/Col2a1/Col3a1/Col5a1/Cyp1b1/Emilin1/Foxc1/Mkx/Scx/Serpinf2	13
GO:1902893	regulation of pri-miRNA transcription by RNA polymerase II	13/1242	57/18303	8.96E-05	0.001319	0.000902	Bmp2/Bmp4/Egr1/Ets1/Fosl1/Gata2/Gata6/Jun/Lilrb4/Nfkb1/Pdgfb/Ppard/Tnf	13
GO:0010634	positive regulation of epithelial cell migration	24/1242	152/18303	8.97E-05	0.001319	0.000902	Angpt4/Anxa3/Aqp1/Bmp4/Cd40/Enpp2/Ets1/Flt4/Gata2/Has2/Hdac9/Hyal1/Itga2/Itgb3/Jun/Mmp9/Pdgfb/Plk2/Plpp3/Ptgs2/Rhoj/Sema5a/Thbs1/Wnt5a	24
GO:0045682	regulation of epidermis	16/1242	81/18303	9.25E-05	0.001357	0.000928	Bmp4/Cdh3/Cyp27b1/Dll1/Errfi1/Foxc1/Foxn1/Macroh2a2/Hey2/Hpse/Inhba/Maff/Mycl/Ppard/Tnf/Wnt5a	16

ID	Description	GeneRatio	BgRatio	pvalue	p.adjust	qvalue	geneID	Count
GO:0045667	regulation of osteoblast differentiation	23/1242	143/18303	9.35E-05	0.001368	0.000935	Areg/Bmp2/Bmp4/Bmp7/Cebpd/Chrd/Cthrc1/Fam20c/Gdpd2/Gli1/Hand2/Igfbp5/Mef2c/Noct/Npnt/Ptger4/Rorb/Sfrp1/Smoc1/Snai2/Tnf/Tnn/Twist2	23
GO:0002833	positive regulation of response to biotic stimulus	25/1242	162/18303	9.45E-05	0.001379	0.000943	Cadm1/Cd180/Cd274/Cxcl1/Ereg/Gbp5/Il18rap/Acod1/Lbp/Lgals9/Mmp12/Mr1/Nlrc4/Nlrc5/Nod2/Pvr/Pla2g5/Pycard/Rasgrp1/Riok3/Ripk2/Tlr2/Trem3/Trim6/Wnt5a	25
GO:0032635	interleukin-6 production	26/1242	172/18303	9.82E-05	0.001425	0.000975	C1qtnf3/Cd200r1/Ereg/Vegfd/Il18rap/Il19/Il1a/Il1b/Il33/Klf2/Lbp/Lgals9/Nod2/Nos2/Pycard/Inava/Ripk2/Sirpa/Syk/Tlr2/Tlr3/Tnf/Tnfaip3/Trpv4/Tslp/Wnt5a	26
GO:0060402	calcium ion transport into cytosol	26/1242	172/18303	9.82E-05	0.001425	0.000975	Adcyap1r1/Agtr1a/Ank2/Bdkrb1/Bmp4/Cacna1g/Casq1/Ccl3/Cemip/Coro1a/Cx3cl1/Cxcl10/Cxcl11/Drd1/Grin2a/Htr2a/Itgb3/Mcoln2/Mcoln3/Ngf/P2ry6/Ryr2/Ryr3/Trpv3/Trpv4/Xcl1	26
GO:0003007	heart morphogenesis	36/1242	273/18303	9.85E-05	0.001425	0.000975	Ankrd1/Bmp2/Bmp4/Bmp5/Bmp7/Col11a1/Col2a1/Col5a1/Dhrs3/Dll1/Efna1/Fat4/Fgfr2/Foxc1/Fzd2/Gata6/Gja5/Hand2/Has2/Hey2/Jun/Mef2c/Npy1r/Nrg1/Plxnd1/Rbm15/Rbpj/Ryr2/Slit2/Slit3/Snai1/Snai2/Tbx3/Thbs1/Tmem100/Wnt5a	36
GO:0042472	inner ear morphogenesis	20/1242	116/18303	9.86E-05	0.001425	0.000975	Clic5/Col11a1/Col2a1/Cthrc1/Ephb2/Ripor2/Fgf9/Fgfr2/Frzb/Fzd2/Gata2/Gbx2/Insig1/Mafb/Myo15a/Myo7a/Pax8/Tshr/Whrn/Wnt5a	20
GO:0007599	hemostasis	27/1242	182/18303	0.000101	0.001452	0.000993	Adra2a/Anxa8/C1qtnf1/Cfh/Cx3cl1/Ephb2/F11r/F13a1/F3/Fbln1/Gna13/Hpse/Itgb3/Mmrn1/Pdgfb/Pik3cb/Plau/Plek/Procr/Selp/Serpinf2/Serping1/Slc7a11/Syk/Tfpi2/Thbd/Thbs1	27
GO:0097191	extrinsic apoptotic signaling pathway	32/1242	232/18303	0.000101	0.001452	0.000993	Atf3/Bcl2a1/Bmp4/Cflar/Col2a1/Csf2/Cx3cl1/Gas1/Gclc/Gstp1/Hspa1b/Il19/Il1a/Il1b/Il33/Inhba/Kitlg/Itprip/Lgals3/Mal/Ngf/Nrg1/Pycard/Sfrp1/Snai2/Thbs1/Timp3/Tlr3/Tnf/Tnfaip3/Tnfrsf1b/Traf1	32
GO:0030203	glycosaminoglycan metabolic process	17/1242	90/18303	0.000101	0.001454	0.000995	Cd44/Cemip/Chst14/Dcn/Foxc1/Has2/Hyal1/Il15/Il1b/Mamdc2/Ndnf/Nfkb1/Pdgfb/Pglyrp1/Pglyrp4/Ptger4/Xy	17
GO:0007517	muscle organ development	49/1242	414/18303	0.000102	0.001455	0.000995	Ankrd1/Ass1/Atf3/Bdnf/Bmp2/Bmp4/Btg2/Bves/Casq1/Cflar/Col11a1/Col14a1/Col3a1/Cyp26b1/Dcn/Dll1/Egr1/Etv1/Ripor2/Fdps/Fgf9/Fgfr2/Fhl1/Flot1/Foxc1/Fzd2/Gata6/Gli1/Hdac9/Hey2/Hmgcr/Itga7/Jph1/Kcnk2/Lif/Maff/Mef2c/Meox2/Mkx/Mylk/Ncam1/Nrg1/Pi16/Plagl1/Pmp22/Rbpj/Ryr2/Scx/Wnt5a	49
GO:0070232	regulation of T cell apoptotic process	11/1242	43/18303	0.000103	0.00146	0.000998	Ada/Blm/Bmp4/Ccl5/Cd274/Dock8/Efna1/Lgals3/Lgals9/Serpinb9/Wnt5a	11
GO:1903053	regulation of extracellular matrix organization	11/1242	43/18303	0.000103	0.00146	0.000998	Cflar/Emilin1/Ets1/Fscn1/Has2/Itgb3/Angptl7/Meltf/Rgcc/Slc2a10/Tnfrsf1b	11
GO:0097305	response to alcohol	53/1242	459/18303	0.000103	0.001461	0.000999	Aacs/Adcy8/Adcyap1r1/Agtr1a/Blm/Ca3/Casp12/Ccl2/Ccl3/Ccl5/Ccnd1/Cldn3/Cpt1a/Csf3/Cyp1b1/Cyp27b1/Drd1/Egr1/Fdps/Fgfr2/Flt1/Fosl1/Ggt1/Glra1/Grin2a/Gstp1/Hmgcr/Hmgcs1/Hpgd/Htr7/Il1b/Inhba/Junb/Klf2/Klf6/Ldlr/Lrp8/Mmp1/Mmp9/Nfkb1/Nfkbia/Nlrp3/Nqo1/P2ry6/Penk/Ptger4/Rgs7/Sfrp1/Slit2/Slit3/Star/Tnf/Vca	53
GO:0050714	positive regulation of protein secretion	29/1242	202/18303	0.000103	0.001461	0.000999	A1cf/Aacs/Acsl4/Adcy8/Adora2a/Agtr1a/Ankrd1/C1qtnf3/Cadm1/Cask/Clec4e/Fgr/Frmd4a/Gpr68/Il1a/Il33/Kcnn4/Mmp13/Hcar2/Pcsk1/Ppard/Ptger4/Rgcc/Sybu/Syk/Tlr2/Tnf/Tnfsf15/Vsnl1	29
GO:0072109	glomerular mesangium	7/1242	18/18303	0.000107	0.001506	0.00103	Bmp4/Bmp7/Cflar/Egr1/Gpr4/Itgb3/Pdgfb	7
GO:0043087	regulation of GTPase activity	49/1242	415/18303	0.000108	0.001519	0.001039	Adprhl1/Als2/Arap3/Arhgef19/Bves/Ccl2/Ccl20/Ccl22/Ccl3/Ccl5/Ccl9/Cd40/Chn1/Cpeb2/Cx3cl1/Dock10/Dock4/Dock8/Elmod1/Epha4/Ephb3/F11r/Flcn/Gpsm1/Jun/Net1/Plcb1/Plekhg4/Plxna2/Plxna3/Plxnd1/Pycard/Ralgapa2/Rap1gap/Rasa2/Rasal3/Rasgrp1/Rasgrp3/Rgma/Rgs14/Rgs16/Rgs7/Rgs9/Slit2/Snx18/Srgap1/Tbc1d9/Wn	49
GO:0071715	icosanoid transport	13/1242	58/18303	0.000108	0.001519	0.001039	Acsl4/Bdkrb2/Il1a/Il1b/Nos2/P2ry2/Pla2g2a/Pla2g5/Pla2r1/Ptges/Slco3a1/Syk/Trpv4	13
GO:1901571	fatty acid derivative transport	13/1242	58/18303	0.000108	0.001519	0.001039	Acsl4/Bdkrb2/Il1a/Il1b/Nos2/P2ry2/Pla2g2a/Pla2g5/Pla2r1/Ptges/Slco3a1/Syk/Trpv4	13
GO:0072110	glomerular mesangial cell proliferation	6/1242	13/18303	0.000109	0.001529	0.001046	Bmp4/Bmp7/Cflar/Egr1/Itgb3/Pdgfb	6
GO:0009266	response to temperature stimulus	32/1242	233/18303	0.00011	0.00153	0.001047	Acot11/Adora1/Casp12/Casq1/Ccl2/Cxcl1/Cxcl10/Cxcl12/Cxcl2/Cxcr4/F3/Gclc/Hspa1b/Htr2a/Ier5/Il1a/Il1b/Il1r1/Irf1/Lxn/Micb/Mmp9/Pde4b/Pmp22/Ppargc1a/Ptgs2/Rgs7/Socs3/Thra/Trpv3/Trpv4/Xylt1	32
GO:0060986	endocrine hormone secretion	14/1242	66/18303	0.000112	0.001565	0.00107	Agtr1a/Aqp1/C1qtnf1/C1qtnf3/Fzd4/Il1b/Inha/Inhba/Lif/Hcar2/Nrg1/Rab11fip1/Tbx3/Ucn2	14
GO:0048562	embryonic organ morphogenesis	41/1242	328/18303	0.000113	0.001565	0.00107	Bmp4/Bmp7/Clic5/Col11a1/Col2a1/Cthrc1/Dll1/Efna1/Ephb2/Ripor2/Fgf9/Fgfr2/Frzb/Fuz/Fzd2/Fzd5/Gas1/Gata2/Gbx2/Gli1/Hand2/Hipk2/Hnf1b/Hoxb5/Hoxb8/Hyal1/Insig1/Mafb/Mdfi/Mef2c/Mmp16/Myo15a/Myo7a/Nkx3-2/Pax8/Ryr2/Tbx3/Tshr/Twist2/Whrn/Wnt5a	41
GO:1905523	positive regulation of macrophage	9/1242	30/18303	0.000115	0.001596	0.001091	Ccl2/Ccl5/Cmklr1/Csf1/Cx3cl1/Rarres2/Thbs1/Tnfsf18/Trpv4	9

ID	Description	GeneRatio	BgRatio	pvalue	p.adjust	qvalue	geneID	Count
GO:0072006	nephron development	25/1242	164/18303	0.000116	0.001598	0.001093	Agtr1a/Aqp1/Bmp2/Bmp4/Bmp7/Cd44/Cfh/Cflar/Dll1/Egr1/Fat4/Foxc1/Gpr4/Hnf1b/Irx3/Itgb3/Lif/Mef2c/Npnt/Pax8/Pdgfb/Pgf/Ptpro/Wnk4/Wnt2b	25
GO:0050671	positive regulation of lymphocyte proliferation	23/1242	145/18303	0.000116	0.001601	0.001095	Ada/Blm/Card11/Ccl5/Cd274/Cd40/Clcf1/Coro1a/Flt3lg/Il15/Il1a/Il1b/Il23a/Itgal/Lgals9/Mef2c/Peli1/Pycard/Rasal3/Ripk2/Syk/Vcam1/Xcl1	23
GO:0006936	muscle contraction	40/1242	318/18303	0.000119	0.001632	0.001116	Ada/Adora1/Adra2a/Ank2/Bdkrb1/Bdkrb2/Cacna1g/Cacnb2/Calcrl/Casq1/Cxcr4/Dock4/Drd1/Ednrb/Flt1/Gja5/Glra1/Htr2a/Htr7/Itga2/Kcnh2/Mybpc1/Myh1/Myl1/Mylk/Npnt/Npy1r/Pde4b/Ptger4/Ptgs2/Ryr2/Ryr3/Scn2b/Sgcd/Tbx3/Tmod1/Tmod2/Tnf/Tnnt3/Trpv4	40
GO:0031649	heat generation	8/1242	24/18303	0.000121	0.001662	0.001137	Arrdc3/Ccl5/Ednrb/Il1a/Il1b/Il1rn/Ptgs2/Tnf	8
GO:0048705	skeletal system morphogenesis	34/1242	255/18303	0.000122	0.001669	0.001141	Barx2/Bmp4/Bmp7/Col11a1/Col13a1/Col1a1/Col2a1/Csrnp1/Cyp26b1/Dhrs3/Fgfr2/Fgr/Foxc1/Frem1/Fuz/Gas1/Has2/Hoxb5/Hoxb8/Hyal1/Insig1/Mdfi/Mef2c/Mmp13/Mmp16/Nkx3-	34
GO:0050770	regulation of axonogenesis	30/1242	214/18303	0.000122	0.001669	0.001141	Bdnf/Cdh4/Chn1/Cxcl12/Efna1/Epha4/Ephb2/Ephb3/Map6/Metrn/Mgll/Ngf/Nrg1/Plxna2/Plxna3/Plxnd1/Rgma/Sema3f/Sema4a/Sema4b/Sema5a/Sema6a/Sema6d/Slit2/Tubb2b/Twf2/Vim/Wnt5a/Zeb2/Zswim5	30
GO:0014013	regulation of gliogenesis	24/1242	155/18303	0.000123	0.001669	0.001141	Aspa/Bmp2/Bmp4/Clcf1/Csf1/Cx3cl1/Cxcr4/Dusp10/Enpp2/Epha4/Flt1/Gpr37l1/Hmga2/Il1b/Ldlr/Lif/Nrg1/Pmp22/Myb/Tenm4/Tlr2/Tnf/Tnfrsf1b/Vim	24
GO:0032092	positive regulation of protein binding	18/1242	100/18303	0.000123	0.001669	0.001141	Bdnf/Bmp2/Bmp4/Csf3/Cthrc1/Epha4/Flot1/Hfe/Hip1r/Hipk2/Mmp9/Ngf/Plk2/Plxnd1/Ramp2/Ripk2/Spon1/W	18
GO:0001892	embryonic placenta development	19/1242	109/18303	0.000126	0.001703	0.001165	Bmp5/Bmp7/Csf2/Fgfr2/Fzd5/Gata2/Hey2/Junb/Lif/Mdfi/Nsdhl/Pdgfb/Prdm1/Rbpj/Snai1/Socs3/St14/Tfeb/Vca	19
GO:0071295	cellular response to vitamin	11/1242	44/18303	0.000128	0.001738	0.001188	Ccl5/Col1a1/Col2a1/Cyp27b1/Fzd2/Il15/Mmp1/Nos2/Penk/Sfrp1/Snai2	11
GO:0001755	neural crest cell migration	13/1242	59/18303	0.00013	0.001748	0.001196	Bmp4/Bmp7/Ednrb/Gbx2/Hand2/Kitlg/Sema3f/Sema4a/Sema4b/Sema5a/Sema6a/Sema6d/Zeb2	13
GO:0035924	cellular response to vascular endothelial growth factor	13/1242	59/18303	0.00013	0.001748	0.001196	Cadm4/Dcn/Dll1/Ern1/Flt1/Flt4/Foxc1/Gas1/Pgf/Ptp4a3/Ramp2/Sema6a/Vcam1	13
GO:0045661	regulation of myoblast	13/1242	59/18303	0.00013	0.001748	0.001196	Bmp4/Cxcl10/Dll1/Ripor2/Flt3lg/Mbnl3/Mef2c/Mkx/Plcb1/Ppard/Prickle1/Tbx3/Tnf	13
GO:0061614	pri-miRNA transcription by RNA polymerase II	13/1242	59/18303	0.00013	0.001748	0.001196	Bmp2/Bmp4/Egr1/Ets1/Fosl1/Gata2/Gata6/Jun/Lilrb4/Nfkb1/Pdgfb/Ppard/Tnf	13
GO:0050890	cognition	44/1242	363/18303	0.000132	0.001759	0.001203	Adcy8/Adora1/Bdnf/Btg2/Csmd1/Ctnnd2/Cyp7b1/Drd1/Egr1/Elavl4/Ephb2/Fosl1/Gpr88/Grin2a/Hmgcr/Htr2a/Htr7/Il1b/Il1rn/Itga5/Jun/Kcnk2/Ldlr/Lrrn4/Mef2c/Mgat3/Ncam1/Ngf/Nrg1/Nrxn3/Pde1b/Plcb1/Plk2/Prkar2b/Prrt1/Ptgs2/Rcan2/Rgs14/Sgk1/Shank1/Slc7a11/Thra/Tlr2	44
GO:0043542	endothelial cell migration	30/1242	215/18303	0.000133	0.001769	0.00121	Angpt4/Anxa3/Bmp4/Bmper/Cd40/Cyp1b1/Dcn/Efna1/Ets1/Flt4/Gata2/Hdac9/Itgb3/Lpxn/Mef2c/Meox2/Pdgfb/Plk2/Plpp3/Plxnd1/Ptgs2/Ptp4a3/Rgcc/Rhoj/Sema4a/Sema5a/Slit2/Thbs1/Tnf/Wnt5a	30
GO:0031638	zymogen activation	14/1242	67/18303	0.000133	0.001769	0.00121	C1rl/Dhcr24/Furin/Ggt1/LOC100910418/Meltf/Nlrc4/Plat/Plau/Pycard/Ripk2/Serpinf2/Thbs1/Vsir	14
GO:0060688	regulation of morphogenesis of a branching structure	14/1242	67/18303	0.000133	0.001769	0.00121	Agtr1a/Bmp4/Bmp7/Fgfr2/Hnf1b/Il1b/Ntn4/Pax8/Pgf/Sfrp1/Snai2/Tnf/Wnt2b/Wnt5a	14
GO:0046651	lymphocyte proliferation	39/1242	309/18303	0.000134	0.001769	0.00121	Ada/Blm/Bmp4/Card11/Ccl5/Cd180/Cd274/Cd40/Cd44/Clcf1/Coro1a/Cxcl12/Cxcr4/Dock8/Ripor2/Flt3lg/Il15/Il1a/Il1b/Il23a/Irf1/Itgal/Lgals3/Lgals9/Mef2c/Peli1/Prdm1/Pycard/Rasal3/Rasgrp1/Ripk2/Satb1/Siglec10/Syk/Tnfrsf1b/Tnfsf18/Vcam1/Vsir/Xcl1	39
GO:0036294	cellular response to decreased oxygen levels	29/1242	205/18303	0.000134	0.001771	0.001211	Angpt4/Ankrd1/Aqp1/Bach1/Bdkrb2/Cflar/Cox4i2/Cpeb2/Cpeb4/Drd1/Egln3/Egr1/Flt1/Fndc1/Gata6/Hilpda/Kcnk2/Kcnk3/Nampt/Ndnf/Plau/Ppard/Ppargc1a/Ppp1r15a/Ptgs2/Rbpj/Rgcc/Sfrp1/Ucn2	29
GO:0014015	positive regulation of gliogenesis	17/1242	92/18303	0.000135	0.001771	0.001211	Aspa/Bmp2/Clcf1/Csf1/Cx3cl1/Cxcr4/Enpp2/Flt1/Il1b/Lif/Nrg1/Myb/Tenm4/Tlr2/Tnf/Tnfrsf1b/Vim	17
GO:0042490	mechanoreceptor differentiation	17/1242	92/18303	0.000135	0.001771	0.001211	Adgrv1/Bdnf/Bmp4/Clic5/Cthrc1/Dll1/Ripor2/Fat4/Fzd2/Hey2/Mcoln3/Mycl/Myo7a/Rbpj/Slc4a7/Tshr/Whrn	17
GO:0002793	positive regulation of peptide secretion	32/1242	236/18303	0.00014	0.001834	0.001254	A1cf/Aacs/Acsl4/Adcy8/Adora1/Adora2a/Agtr1a/Ankrd1/C1qtnf3/Cadm1/Cask/Clec4e/Fgr/Frmd4a/Gpr68/Hfe/Il1a/Il33/Kcnn4/Lif/Mmp13/Hcar2/Pcsk1/Ppard/Ptger4/Rgcc/Sybu/Syk/Tlr2/Tnf/Tnfsf15/Vsnl1	32
GO:0008544	epidermis development	38/1242	299/18303	0.00014	0.001836	0.001255	Bmp4/Cdh3/Clic5/Cyp26b1/Cyp27b1/Dll1/Edar/Errfi1/Fa2h/Ripor2/Fgfr2/Foxc1/Foxn1/Fuz/Gli1/Macroh2a2/Hey2/Hpse/Igfbp5/Il1a/Inhba/Klk14/Lgr5/Maff/Mcoln3/Mycl/Myo7a/Nsdhl/Ppard/Rbpj/Satb1/Slc4a7/Snai1/St14/Svep1/Tnf/Whrn/Wnt5a	38
GO:0061326	renal tubule development	19/1242	110/18303	0.000142	0.00186	0.001272	Agtr1a/Aqp1/Bmp2/Bmp4/Cd44/Col4a1/Dll1/Fat4/Hnf1b/Irx3/Lgr5/Lif/Mef2c/Npnt/Pax8/Pgf/Stat1/Wnk4/Wn	19
GO:0050810	regulation of steroid biosynthetic process	16/1242	84/18303	0.000145	0.001884	0.001288	Bmp2/Bmp5/Cyp27b1/Cyp51/Egr1/Fdps/Il1a/Il1b/Insig1/Nfkb1/Ppargc1a/Snai1/Snai2/Star/Stard4/Tnf	16

ID	Description	GeneRatio	BgRatio	pvalue	p.adjust	qvalue	geneID	Count
GO:0060113	inner ear receptor cell differentiation	16/1242	84/18303	0.000145	0.001884	0.001288	Adgrv1/Bmp4/Clic5/Cthrc1/Dll1/Ripor2/Fat4/Fzd2/Hey2/Mcoln3/Mycl/Myo7a/Rbpj/Slc4a7/Tshr/Whrn	16
GO:1901863	positive regulation of muscle tissue development	16/1242	84/18303	0.000145	0.001884	0.001288	Bmp4/Cyp26b1/Dll1/Fdps/Fgf9/Fgfr2/Flot1/Gata6/Gli1/Hey2/Hmgcr/Mef2c/Ncam1/Nrg1/Ppargc1a/Rbpj	16
GO:0042692	muscle cell differentiation	48/1242	409/18303	0.000146	0.001889	0.001292	Ank2/Barx2/Bdnf/Bmp2/Bmp4/Bves/Casq1/Cd53/Cflar/Col14a1/Csrp2/Cth/Cxcl10/Cxcl12/Cyp26b1/Dll1/Ednrb/Ehd2/Ereg/Ripor2/Fdps/Fgf9/Fgfr2/Flot1/Flt3lg/Gata6/Gdf15/Hdac9/Hey2/Igfbp5/Mef2c/Npnt/Nrg1/Pdgfb/Pi16/Pmp22/Prdm6/Ramp2/Rbm38/Rbpj/Sgcd/Sorbs2/Tbx3/Thra/Tmod1/Tmod2/Tnnt3/Xirp1	48
GO:0050817	coagulation	27/1242	186/18303	0.000146	0.001889	0.001292	Adra2a/Anxa8/C1qtnf1/Cfh/Cx3cl1/Ephb2/F11r/F13a1/F3/Fbln1/Gna13/Hpse/Itgb3/Mmrn1/Pdgfb/Pik3cb/Plau/Plek/Procr/Selp/Serpinf2/Serping1/Slc7a11/Syk/Tfpi2/Thbd/Thbs1	27
GO:0060760	positive regulation of response to cytokine stimulus	12/1242	52/18303	0.000147	0.001897	0.001298	Crebrf/Csf1/Cxcr4/Hspa1b/Il1r1/Mmp12/Nlrc5/Ripk2/Tlr2/Trim6/Tslp/Wnt5a	12
GO:0050768	negative regulation of neurogenesis	42/1242	343/18303	0.000148	0.001906	0.001304	Bdnf/Bmp4/Bmp7/Col3a1/Cspg4/Dll1/Dnm3/Dusp10/Ednrb/Efna1/Epha4/Ephb2/Fat3/Fuom/Gpr37l1/Hmga2/Il15ra/Il1b/Irx3/Ldlr/Nr2f1/Nrg1/Plk2/Plxna3/Pmp22/Rap1gap/Rbpj/Rgma/Rtn4rl2/Sema3f/Sema4a/Sema4b/Sema5a/Sema6a/Sema6d/Slit2/Thrb/Tnn/Trpv4/Vim/Wnt5	42
GO:0046879	hormone secretion	45/1242	376/18303	0.000149	0.001911	0.001307	Aacs/Acsl4/Adcy8/Adora1/Adra2a/Agtr1a/Aqp1/C1qtnf1/C1qtnf3/Cask/Ccl5/Cpt1a/Fzd4/Gpr68/Hadh/Hfe/Hmga2/Hmgcr/Hnf1b/Il11/Il1b/Il1rn/Inha/Inhba/Lif/Ltbp4/Mafa/Hcar2/Nos2/Npy1r/Nrg1/Pax8/Pclo/Ppard/Ptger4/Myb/Rab11fip1/Rfx3/Sfrp1/Sybu/Tbx3/Tnf/Trpv4/Ucn	45
GO:0042471	ear morphogenesis	22/1242	138/18303	0.00015	0.001924	0.001316	Clic5/Col11a1/Col2a1/Cthrc1/Ephb2/Ripor2/Fgf9/Fgfr2/Frzb/Fzd2/Gas1/Gata2/Gbx2/Insig1/Mafb/Myo15a/Myo7a/Nkx3-2/Pax8/Tshr/Whrn/Wnt5a	22
GO:0030212	hyaluronan metabolic process	9/1242	31/18303	0.000153	0.00195	0.001333	Cd44/Cemip/Has2/Hyal1/Il15/Il1b/Nfkb1/Pdgfb/Ptger4	9
GO:0006469	negative regulation of protein kinase activity	31/1242	227/18303	0.000155	0.001973	0.001349	Adora2a/Bmp4/Bmp7/Drd1/Dusp10/Dusp16/Dusp19/Dusp5/Dusp6/Ephb2/Errfi1/Fabp4/Gadd45g/Gstp1/Hmgcr/Il1b/Itprip/Pcp4/Prkar2a/Prkar2b/Ptpn5/Ptprb/Ptpro/Ptprt/Pycard/Rgs14/Serpinb3/Sfrp1/Smcr8/Spry4/Tnfaip3	31
GO:2001236	regulation of extrinsic apoptotic signaling pathway	25/1242	167/18303	0.000155	0.001974	0.00135	Atf3/Bmp4/Cflar/Col2a1/Csf2/Cx3cl1/Gas1/Gclc/Gstp1/Hspa1b/Il19/Il1b/Inhba/Itprip/Lgals3/Mal/Nrg1/Pycard/Sfrp1/Snai2/Thbs1/Timp3/Tnf/Tnfaip3/Traf1	25
GO:0010721	negative regulation of cell development	47/1242	399/18303	0.000156	0.001985	0.001358	Bdnf/Bmp4/Bmp7/Col3a1/Cspg4/Dll1/Dnm3/Dusp10/Ednrb/Efna1/Epha4/Ephb2/Fat3/Fbln1/Frzb/Fuom/Gpr37l1/Hmga2/Il15ra/Il1a/Il1b/Irx3/Ldlr/Meltf/Nr2f1/Nrg1/Pi16/Plk2/Plxna3/Pmp22/Rap1gap/Rbpj/Rgma/Rtn4rl2/Sema3f/Sema4a/Sema4b/Sema5a/Sema6a/Sema6d/Slit2/Thrb/Tnn/Trpv4/Vim/Wnt5a/Xylt1	47
GO:0001558	regulation of cell growth	51/1242	444/18303	0.000157	0.001994	0.001364	Acsl4/Bdkrb1/Bdnf/Ccdc85b/Cd44/Cdh4/Col14a1/Cth/Cxcl12/Cxcl16/Cxcr4/Cyp27b1/Extl3/Fbln5/Fdps/Fhl1/Flcn/Flt4/Frzb/Gas1/Hspa1b/Hyal1/Igfbp4/Igfbp5/Inhba/Kazald1/Mgll/Ngf/Nrg1/Osgin1/Pi16/Plxna3/Ppard/Cavin3/Rerg/Rgma/Sema3f/Sema4a/Sema4b/Sema5a/Sema6a/Sema6d/Sfrp1/Sgk1/Slit2/Slit3/Socs3/Twf2/Wfdc1/	51
GO:0097553	calcium ion transmembrane import into cytosol	23/1242	148/18303	0.000159	0.002001	0.001368	Agtr1a/Ank2/Bdkrb1/Casq1/Ccl3/Cemip/Coro1a/Cx3cl1/Cxcl10/Cxcl11/Drd1/Grin2a/Htr2a/Itgb3/Mcoln2/Mcoln3/Ngf/P2ry6/Ryr2/Ryr3/Trpv3/Trpv4/Xcl1	23
GO:0051216	cartilage development	29/1242	207/18303	0.000159	0.002001	0.001368	Barx2/Bmp2/Bmp3/Bmp4/Cd44/Col11a1/Col1a1/Col2a1/Fgf9/Frzb/Gdf6/Hand2/Hmga2/Hyal1/Mef2c/Mgp/Mmp13/Nkx3-2/Pth1r/Snorc/Scx/Snai1/Snai2/Thbs1/Thra/Thrb/Trpv4/Wnt2b/Wnt5a	29
GO:0002369	T cell cytokine production	11/1242	45/18303	0.00016	0.002001	0.001368	Fzd5/Hfe/Il18rap/Il1b/Il1r1/Il31ra/Nlrp3/Rsad2/Tnfrsf1b/Vsir/Xcl1	11
GO:0002686	negative regulation of leukocyte migration	11/1242	45/18303	0.00016	0.002001	0.001368	Ada/Adora1/Ccl2/Cd200r1/Cxcl12/Emilin1/Ripor2/Il33/Plcb1/Ptger4/Slit2	11
GO:1905521	regulation of macrophage	11/1242	45/18303	0.00016	0.002001	0.001368	Ccl2/Ccl5/Cd200r1/Cmklr1/Csf1/Cx3cl1/Emilin1/Rarres2/Thbs1/Tnfsf18/Trpv4	11
GO:0060840	artery development	19/1242	111/18303	0.000161	0.002013	0.001377	Adamts6/Bmp4/Col3a1/Cxcr4/Foxc1/Fuz/Gja5/Hand2/Hey2/Hpgd/Ldlr/Mylk/Pde2a/Plxnd1/Prdm1/Prickle1/Ptger4/Rbpj/Slc2a10	19
GO:0071634	regulation of transforming growth factor beta	10/1242	38/18303	0.000163	0.002039	0.001395	Cdh3/Cx3cl1/Furin/Gata6/Lgals9/Ptgs2/Myb/Serpinf2/Thbs1/Xcl1	10
GO:0031099	regeneration	40/1242	323/18303	0.000166	0.002065	0.001412	Anxa3/Atp2a3/Bdnf/Ccl2/Ccnd1/Cflar/Cpt1a/Cspg5/Cxcl12/Dusp10/Elavl4/Eno3/Epha4/Eppk1/Flt1/Ggt1/Gli1/Gstp1/Hfe/Jun/Lif/Matn2/Ncam1/Nefh/Nfkbia/Nrep/Nrg1/Pgf/Plau/Plaur/Ppard/Rgma/Scarf1/Sgca/Socs3/Timp3/Tnf/Ucn2/Vim/Xylt1	40
GO:0009408	response to heat	22/1242	139/18303	0.000167	0.002073	0.001418	Casq1/Ccl2/Cxcl1/Cxcl10/Cxcl12/Cxcl2/Gclc/Hspa1b/Ier5/Il1a/Il1b/Il1r1/Irf1/Micb/Mmp9/Pde4b/Pmp22/Ptgs2/Socs3/Trpv3/Trpv4/Xylt1	22

ID	Description	GeneRatio	BgRatio	pvalue	p.adjust	qvalue	geneID	Count
GO:0015732	prostaglandin	8/1242	25/18303	0.000168	0.002073	0.001418	Acsl4/Il1a/Il1b/Nos2/P2ry2/Ptges/Slco3a1/Trpv4	8
GO:2000193	positive regulation of fatty acid transport	8/1242	25/18303	0.000168	0.002073	0.001418	Erfe/Fabp3/Il1a/Il1b/P2ry2/Pla2r1/Ptges/Trpv4	8
GO:0007160	cell-matrix adhesion	30/1242	218/18303	0.000171	0.002111	0.001444	Bcam/Cask/Col13a1/Col3a1/Col5a3/Coro2b/Csf1/Emilin1/Enpp2/Frem1/Hpse/Itga11/Itga2/Itgal/Itgb3/Itgbl1/Limch1/Mmp12/Msln/Nid2/Npnt/Pcsk5/Pik3cb/Plau/Plet1/Sirpa/Sned1/Thbs1/Tnn/Vcam1	30
GO:0052548	regulation of endopeptidase activity	52/1242	457/18303	0.000173	0.002125	0.001454	Adora2a/Ambp/Anxa8/Aqp1/Atp2a3/Birc3/Casp12/Cd44/Cflar/Dhcr24/Efna1/Egln3/Epha4/F3/Fnip1/Furin/Hip1r/Hspa1b/LOC103689965/Lgals9/Lxn/Mical1/Mmp9/Naip6/Ngf/Nlrc4/Nlrp3/Plaur/Prr7/Ptgs2/Pycard/Ripk2/Serpina3n/Serpinb10/Serpinb2/Serpinb3/Serpinb3a/Serpinb6b/Serpinb9/Serpinf2/Serping1/Stat1/Syk/Tfpi2/Thbs1/Timp3/Tnf/Tnfsf15/Vsir/Wfdc1/Wfdc18/Wfdc21	52
GO:0071456	cellular response to hypoxia	26/1242	178/18303	0.000174	0.002135	0.00146	Angpt4/Ankrd1/Aqp1/Bach1/Bdkrb2/Cflar/Cox4i2/Cpeb2/Drd1/Egln3/Egr1/Flt1/Fndc1/Gata6/Hilpda/Kcnk2/Kcnk3/Ndnf/Plau/Ppard/Ppargc1a/Ptgs2/Rbpj/Rgcc/Sfrp1	26
GO:0070301	cellular response to hydrogen peroxide	21/1242	130/18303	0.000174	0.002135	0.00146	Akr1b1/Aqp1/Casp12/Cfh/Cyp1b1/Ern1/Ets1/F3/Gpr37/Gpr37l1/Hk3/Il18rap/Klf2/Klf6/Net1/Nqo1/Ppif/Pycr1/Myb/Sirpa/Tnfaip3	21
GO:0032309	icosanoid secretion	12/1242	53/18303	0.000179	0.002177	0.001489	Acsl4/Bdkrb2/Il1a/Il1b/Nos2/P2ry2/Pla2g2a/Pla2g5/Pla2r1/Ptges/Syk/Trpv4	12
GO:0043407	negative regulation of MAP kinase activity	15/1242	77/18303	0.000179	0.002177	0.001489	Bmp4/Bmp7/Dusp10/Dusp16/Dusp19/Dusp5/Dusp6/Gstp1/Hmgcr/Il1b/Ptpn5/Rgs14/Serpinb3/Sfrp1/Spry4	15
GO:1903670	regulation of sprouting	15/1242	77/18303	0.000179	0.002177	0.001489	Agtr1a/Bmper/Creb3l1/Dll1/Flt1/Gata2/Hdac9/Itga5/Klf2/Meox2/Plk2/Ptgs2/Rhoj/Sema6a/Thbs1	15
GO:0030213	hyaluronan biosynthetic process	6/1242	14/18303	0.00018	0.002192	0.001499	Has2/Hyal1/Il1b/Nfkb1/Pdgfb/Ptger4	6
GO:0052547	regulation of peptidase activity	54/1242	481/18303	0.000184	0.002233	0.001527	Adora2a/Ambp/Anxa8/Aqp1/Atp2a3/Birc3/Casp12/Cd44/Cflar/Cldn3/Dhcr24/Efna1/Egln3/Epha4/F3/Fbln1/Fnip1/Furin/Hip1r/Hspa1b/LOC103689965/Lgals9/Lxn/Mical1/Mmp9/Naip6/Ngf/Nlrc4/Nlrp3/Plaur/Prr7/Ptgs2/Pycard/Ripk2/Serpina3n/Serpinb10/Serpinb2/Serpinb3/Serpinb3a/Serpinb6b/Serpinb9/Serpinf2/Serping1/Stat1/Syk/Tfpi2/Thbs1/Timp3/Tnf/Tnfsf15/Vsir/Wfdc1/Wfdc18/	54
GO:1903510	mucopolysaccharide metabolic process	14/1242	69/18303	0.000186	0.002248	0.001537	Cd44/Cemip/Chst14/Dcn/Has2/Hyal1/Il15/Il1b/Mamdc2/Ndnf/Nfkb1/Pdgfb/Ptger4/Xylt1	14
GO:0032655	regulation of interleukin-12 production	13/1242	61/18303	0.000186	0.002248	0.001538	Cd40/Cmklr1/Il23a/Irf1/Lgals9/Nfkb1/Nod2/Plcb1/Rel/Syk/Thbs1/Tlr2/Tlr3	13
GO:0090303	positive regulation of wound healing	13/1242	61/18303	0.000186	0.002248	0.001538	Adra2a/Ccl2/Cldn3/Cxcr4/F11r/Hpse/Mylk/Plek/Plpp3/Ptger4/Selp/Serpinf2/Thbs1	13
GO:0019935	cyclic-nucleotide-mediated signaling	31/1242	230/18303	0.000197	0.002352	0.001609	Adcy8/Adcyap1r1/Adgrd1/Adora2a/Adra2a/Aqp1/Arrdc3/Calcrl/Cxcl10/Cxcl11/Drd1/Ednrb/Gna13/Gpr4/Htr7/Lgr5/Nos2/Pclo/Pde2a/Pde4b/Prkar2a/Prkar2b/Ptger1/Ptger4/Ptgir/Pth1r/Ramp2/Tcp11/Thbs1/Tshr/Ucn2	31
GO:0001961	positive regulation of cytokine-mediated signaling pathway	11/1242	46/18303	0.000197	0.002352	0.001609	Crebrf/Csf1/Cxcr4/Hspa1b/Il1r1/Mmp12/Nlrc5/Ripk2/Trim6/Tslp/Wnt5a	11
GO:0032814	regulation of natural killer cell activation	11/1242	46/18303	0.000197	0.002352	0.001609	Fgr/Flt3lg/Il15/Il15ra/Il23a/Lgals9/Pglyrp1/Pglyrp4/Prdm1/Rasgrp1/Tox	11
GO:0035987	endodermal cell differentiation	11/1242	46/18303	0.000197	0.002352	0.001609	Col11a1/Col5a1/Col7a1/Gata6/Hmga2/Hnf1b/Inhba/Itga5/Itga7/Lamb3/Mmp9	11
GO:0071526	semaphorin-plexin signaling pathway	11/1242	46/18303	0.000197	0.002352	0.001609	Hand2/Ncam1/Plxna2/Plxna3/Plxnd1/Sema3f/Sema4a/Sema4b/Sema5a/Sema6a/Sema6d	11
GO:0001958	endochondral ossification	9/1242	32/18303	0.0002	0.002367	0.001619	Bmp4/Col13a1/Col1a1/Col2a1/Foxc1/Mef2c/Mmp13/Mmp16/Scx	9
GO:0036037	CD8-positive, alpha-beta T cell activation	9/1242	32/18303	0.0002	0.002367	0.001619	Cd274/Hfe/Irf1/Pax1/Satb1/Tnfsf8/Tox/Vsir/Xcl1	9
GO:0036075	replacement ossification	9/1242	32/18303	0.0002	0.002367	0.001619	Bmp4/Col13a1/Col1a1/Col2a1/Foxc1/Mef2c/Mmp13/Mmp16/Scx	9
GO:0010976	positive regulation of neuron projection development	45/1242	381/18303	0.000201	0.002382	0.001629	Ankrd1/Bdnf/Bmp4/Bmp5/Bmp7/Cask/Cdh4/Cflar/Cobl/Cx3cl1/Cxcl12/Cxcr4/Elavl4/Epha4/Fez1/Fzd4/Htr7/Lif/Lrp8/Map6/Metrn/Ndnf/Ngf/Nrg1/Obsl1/P2ry2/Palm/Pcp4/Plxna2/Plxna3/Plxnd1/Ptpn5/Rgma/Ror1/Scarf1/Sema5a/Sgk1/Shank1/Tnik/Tox/Tubb2b/Twf2/Wnt5a/Zd	45
GO:0045666	positive regulation of neuron differentiation	54/1242	483/18303	0.000204	0.002414	0.001651	Ankrd1/Bdnf/Bmp2/Bmp4/Bmp5/Bmp7/Cask/Ccl5/Cdh4/Cflar/Cobl/Cx3cl1/Cxcl12/Cxcr4/Elavl4/Epha4/Fez1/Fzd4/Gata2/Gdf6/Htr7/Irx3/Lif/Lrp8/Map6/Mef2c/Metrn/Mmd/Ndnf/Ngf/Nrg1/Obsl1/P2ry2/Palm/Pcp4/Plxna2/Plxna3/Plxnd1/Ptpn5/Rgma/Ror1/Scarf1/Sema5a/Sgk1/Sh3gl3/Shank1/Tgif1/Tnik/Tox/Tubb2b/Twf2/Wnt5a/Z	54
GO:0032755	positive regulation of interleukin-6 production	18/1242	104/18303	0.000205	0.002422	0.001656	Ereg/Vegfd/Il1a/Il1b/Il33/Lbp/Lgals9/Nod2/Pycard/Inava/Ripk2/Syk/Tlr2/Tlr3/Tnf/Trpv4/Tslp/Wnt5a	18
GO:0022410	circadian sleep/wake cycle process	10/1242	39/18303	0.000207	0.002425	0.001659	Ada/Adora1/Adora2a/Csf2/Drd1/Hcrtr2/Htr7/Pglyrp1/Ptger4/Star	10
GO:0071371	cellular response to gonadotropin	10/1242	39/18303	0.000207	0.002425	0.001659	Cyp1b1/Egr1/Gata6/Gclc/Hmgcs1/Inhba/Pax8/Plat/Ppargc1a/Star	10

ID	Description	GeneRatio	BgRatio	pvalue	p.adjust	qvalue	geneID	Count
GO:0042129	regulation of T cell proliferation	26/1242	180/18303	0.000208	0.002442	0.00167	Blm/Bmp4/Card11/Ccl5/Cd274/Cd44/Coro1a/Ripor2/Il15/Il1a/Il1b/Il23a/Irf1/Itgal/Lgals3/Lgals9/Peli1/Pycard/Rasal3/Ripk2/Syk/Tnfrsf1b/Tnfsf18/Vcam1/Vsir/Xcl1	26
GO:0051961	negative regulation of nervous system development	44/1242	371/18303	0.000215	0.002514	0.00172	Bdnf/Bmp4/Bmp7/Col3a1/Cspg4/Dll1/Dnm3/Dusp10/Ednrb/Efna1/Epha4/Ephb2/Fat3/Fuom/Gpr37l1/Hmga2/Il15ra/Il1b/Irx3/Ldlr/Nr2f1/Nrg1/Plk2/Plxna3/Pmp22/Rap1gap/Rbpj/Rgma/Rtn4rl2/Sema3f/Sema4a/Sema4b/Sema5a/Sema6a/Sema6d/Slit2/Thrb/Tlr2/Tnf/Tnn/Trpv4/	44
GO:2000351	regulation of endothelial cell apoptotic process	12/1242	54/18303	0.000216	0.002516	0.001721	Angptl4/Bmp4/Ccl2/Cd40/Gata2/Ndnf/Ramp2/Rgcc/Sema5a/Thbs1/Tnf/Tnfaip3	12
GO:0031670	cellular response to nutrient	13/1242	62/18303	0.000221	0.002578	0.001763	Acat2/Ccl5/Col1a1/Col2a1/Cyp11b2/Cyp27b1/Fzd2/Il15/Mmp1/Nos2/Penk/Sfrp1/Snai2	13
GO:0043281	regulation of cysteine-type endopeptidase activity involved in	30/1242	222/18303	0.000236	0.002745	0.001878	Adora2a/Aqp1/Atp2a3/Birc3/Casp12/Cd44/Cflar/Dhcr24/Egln3/F3/Fnip1/Hip1r/Hspa1b/Lgals9/Mical1/Mmp9/Naip6/Ngf/Nlrc4/Nlrp3/Plaur/Prr7/Ptgs2/Pycard/Serpinb9/Stat1/Syk/Thbs1/Tnf/Tnfsf15	30
GO:0007369	gastrulation	28/1242	202/18303	0.000246	0.00285	0.001949	Bmp4/Bmp7/Chrd/Col11a1/Col5a1/Col7a1/Dusp5/Fgfr2/Foxc1/Gata6/Hmga2/Hnf1b/Il1rn/Inhba/Itga2/Itga5/Itga7/Itgb3/Lamb3/Mmp9/Plpp3/Scx/Sfrp1/Snai1/Tenm4/Tgif1/Txnrd1/Wnt5a	28
GO:0050830	defense response to Gram-positive bacterium	20/1242	124/18303	0.000251	0.002909	0.001989	Camp/Fgr/Gbp4/LOC290595/Lbp/Lyz2/Mr1/Myo1f/Nlrp3/Nod2/Pglyrp1/Pglyrp4/Pla2g2a/Rarres2/Ripk2/Scd/Ssc5d/Tlr2/Tnf/Tnfsf8	20
GO:0014823	response to activity	22/1242	143/18303	0.000254	0.00293	0.002004	Agtr1a/Bdnf/Ccl2/Ccl5/Cxcr4/Drd1/Flt1/Gclc/Hadh/Il1rn/Itga2/Itgb3/Ncam1/Nos2/Plau/Ppard/Ppargc1a/Rarres2/Ryr2/Slc25a25/Star/Tnf	22
GO:0061045	negative regulation of wound healing	14/1242	71/18303	0.000255	0.002934	0.002007	C1qtnf1/Cask/Cldn3/Dusp10/Eppk1/Hmgcr/Pdgfb/Plau/Serpinf2/Serping1/Thbd/Thbs1/Tnf/Wfdc1	14
GO:0045665	negative regulation of neuron differentiation	34/1242	265/18303	0.000257	0.002947	0.002015	Bmp7/Cspg4/Dll1/Dnm3/Ednrb/Efna1/Epha4/Ephb2/Fat3/Fuom/Gpr37l1/Il15ra/Il1b/Irx3/Nr2f1/Plk2/Plxna3/Pmp22/Rap1gap/Rbpj/Rgma/Rtn4rl2/Sema3f/Sema4a/Sema4b/Sema5a/Sema6a/Sema6d/Slit2/Thrb/Trpv4/Vim/	34
GO:0002724	regulation of T cell cytokine production	9/1242	33/18303	0.000258	0.002947	0.002015	Fzd5/Hfe/Il1b/Il1r1/Nlrp3/Rsad2/Tnfrsf1b/Vsir/Xcl1	9
GO:0033687	osteoblast	9/1242	33/18303	0.000258	0.002947	0.002015	Bmp2/Cthrc1/Fbln5/Hpse/Itgb3/Junb/Npr3/Sfrp1/Tnn	9
GO:1905314	semi-lunar valve development	9/1242	33/18303	0.000258	0.002947	0.002015	Bmp4/Efna1/Emilin1/Gja5/Hey2/Rbpj/Slit2/Slit3/Tnfrsf1b	9
GO:0030099	myeloid cell differentiation	48/1242	419/18303	0.000258	0.002947	0.002015	Bmp4/Ccl3/Ccl5/Ccr1/Csf1/Csf2/Csf3/Dll1/Ets1/Fam20c/Faxdc2/Fli1/Gata2/Gpr68/Hoxb8/Hspa1b/Ikzf1/Il15/Il23a/Il31ra/Inhba/Isg15/Jun/Junb/Kitlg/Klf2/LOC102551184/Lif/Lilrb4/Lmo2/Mafb/Mef2c/Nfkbia/Pde1b/Pde2a/Myb/RGD1562378/Rbm15/Rbp1/Rbpj/Relb/Scin/Sfrp1/Stat1/Thra/Tlr2/Tnf/Twist2	48
GO:0071604	transforming growth factor beta	10/1242	40/18303	0.000259	0.002949	0.002017	Cdh3/Cx3cl1/Furin/Gata6/Lgals9/Ptgs2/Myb/Serpinf2/Thbs1/Xcl1	10
GO:0050886	endocrine process	18/1242	106/18303	0.000263	0.002986	0.002042	Agtr1a/Aqp1/C1qtnf1/C1qtnf3/Fzd4/Gja5/Il1b/Inha/Inhba/Lif/Hcar2/Nox1/Nrg1/Pcsk5/Rab11fip1/Serpinf2/Tbx3/Ucn2	18
GO:0002366	leukocyte activation involved in immune response	35/1242	276/18303	0.000263	0.002986	0.002042	Ada/Anxa3/Bcl3/Ccl3/Cd180/Cd40/Clcf1/Clec4e/Coro1a/Dll1/Dock10/Ercc1/Exosc6/Fgr/Gata2/Il13ra2/Il23a/Il33/Itgal/Lbp/Lgals3/Lgals9/Myo1f/Nlrp3/Pglyrp1/Pglyrp4/Ptger4/Pycard/Myb/Rasgrp1/Relb/Ripk2/Sema4a/Sy	35
GO:0007613	memory	24/1242	163/18303	0.000269	0.003041	0.00208	Adcy8/Bdnf/Csmd1/Cyp7b1/Drd1/Egr1/Grin2a/Htr2a/Htr7/Il1b/Il1rn/Itga5/Kcnk2/Ldlr/Lrrn4/Ngf/Nrg1/Plcb1/Plk2/Ptgs2/Rcan2/Rgs14/Sgk1/Shank1	24
GO:2000391	positive regulation of neutrophil extravasation	5/1242	10/18303	0.00027	0.003041	0.00208	Cd99l2/Ripor2/Il1a/Il1r1/Ptger4	5
GO:2000562	negative regulation of CD4-positive, alpha-beta T cell proliferation	5/1242	10/18303	0.00027	0.003041	0.00208	Cd274/Cd44/Lgals9/Vsir/Xcl1	5
GO:1903036	positive regulation of response to	15/1242	80/18303	0.000278	0.003124	0.002137	Adra2a/Ccl2/Cldn3/Cxcr4/F11r/Hpse/Mylk/Nrg1/Plek/Plpp3/Ptger4/Scarf1/Selp/Serpinf2/Thbs1	15
GO:0098742	cell-cell adhesion via plasma-membrane adhesion molecules	32/1242	245/18303	0.000279	0.003134	0.002144	Alcam/Bmp2/Cadm1/Cdh3/Cdh4/Cldn3/Clstn2/Cx3cl1/Fat3/Fat4/Flrt3/Igsf9/Igsf9b/Il1rn/Itga5/Itgal/Jaml/Pcdhb6l/Ncam1/Nectin1/Nrg1/Obsl1/Pvr/Pcdh18/Pcdhb14/Pcdhb3/Pcdhb5/Pik3cb/Ptprt/Selp/Tenm4/Vcam1	32
GO:0032494	response to peptidoglycan	6/1242	15/18303	0.000283	0.003164	0.002164	Camp/Nlrp3/Nod2/Inava/Ripk2/Tlr2	6
GO:0034116	positive regulation of heterotypic cell-cell adhesion	6/1242	15/18303	0.000283	0.003164	0.002164	Alox15/Bmp7/Cd44/Flot1/Il1b/Tnf	6
GO:0070431	nucleotide-binding oligomerization domain containing 2 signaling pathway	6/1242	15/18303	0.000283	0.003164	0.002164	Hspa1b/Nfkbia/Nod2/Inava/Ripk2/Tnfaip3	6

ID	Description	GeneRatio	BgRatio	pvalue	p.adjust	qvalue	geneID	Count
GO:0002819	regulation of adaptive immune response	28/1242	204/18303	0.00029	0.003223	0.002204	Ada/Alox15/Cd274/Cd40/Clcf1/Dusp10/Exosc6/Fzd5/Hfe/Hspa1b/Il1b/Il1r1/Il23a/Il33/Mef2c/Mr1/Nlrp3/Nod2/Pvr/Pycard/Ripk2/Rsad2/Tnf/Tnfaip3/Tnfrsf1b/Tnfsf18/Vsir/Xcl1	28
GO:0045089	positive regulation of innate immune response	19/1242	116/18303	0.00029	0.003223	0.002204	Cadm1/Ereg/Gbp5/Il18rap/Lbp/Lgals9/Mmp12/Nlrc4/Nlrc5/Nod2/Pvr/Pla2g5/Pycard/Rasgrp1/Riok3/Tlr2/Trem3/Trim6/Wnt5a	19
GO:0097756	negative regulation of blood vessel diameter	19/1242	116/18303	0.00029	0.003223	0.002204	Adora1/Adra2a/Agtr1a/Bdkrb2/Cacna1g/Cx3cl1/Dock4/Drd1/Dusp5/Ednrb/Gja5/Hmgcr/Hspa1b/Htr2a/Htr7/Mgll/Npy1r/Olr1/Ptgs2	19
GO:0002718	regulation of cytokine production involved in immune	16/1242	89/18303	0.000291	0.003228	0.002208	Fzd5/Hfe/Il1b/Il1r1/Nlrp3/Nod2/Rsad2/Tlr2/Tlr3/Tnf/Tnfrsf1b/Tril/Trim6/Vsir/Wnt5a/Xcl1	16
GO:0030431	sleep	11/1242	48/18303	0.000295	0.003256	0.002227	Ada/Adora1/Adora2a/Csf2/Drd1/Grin2a/Htr2a/Htr7/Pglyrp1/Ptger4/Star	11
GO:1901568	fatty acid derivative metabolic process	24/1242	164/18303	0.000295	0.003256	0.002227	Abcd1/Acat2/Acsl1/Acsl3/Acsl4/Alox15/Bdkrb1/Cyp1b1/Cyp2j4/Elovl4/Fabp5/Ggt1/Gsta2/Gstp1/Hpgd/Il1b/Mgll/Mgst3/Pla2g5/Ptges/Ptgr1/Ptgs2/Syk/Tlr2	24
GO:0051145	smooth muscle cell differentiation	14/1242	72/18303	0.000297	0.003266	0.002234	Bmp4/Cth/Ednrb/Ereg/Fgf9/Fgfr2/Gata6/Hey2/Mef2c/Npnt/Pdgfb/Prdm6/Ramp2/Tbx3	14
GO:0045445	myoblast differentiation	17/1242	98/18303	0.000297	0.003266	0.002234	Bmp4/Csrp2/Cxcl10/Dll1/Ripor2/Flt3lg/Hmgcr/Mbnl3/Mef2c/Mkx/Nrg1/Plcb1/Ppard/Prickle1/Rbpj/Tbx3/Tnf	17
GO:0045995	regulation of embryonic development	21/1242	135/18303	0.000297	0.003266	0.002234	Bmp4/Bmp7/Col5a1/Csf2/Dll1/Dmrt2/Fuz/Fzd2/Gata2/Il1rn/Lama2/Noct/Pdgfb/Plcb1/Scx/Sfrp1/Snai1/Tenm4/Tgif1/Wnt2b/Wnt5a	21
GO:0003071	renal system process involved in regulation of systemic arterial	8/1242	27/18303	0.000305	0.003318	0.002269	Adora1/Agtr1a/Coro2b/Gja5/Pcsk5/Pdgfb/Ptpro/Serpinf2	8
GO:0014912	negative regulation of smooth muscle cell migration	8/1242	27/18303	0.000305	0.003318	0.002269	Gstp1/Igfbp5/Mef2c/Ppard/Ppargc1a/Rbpj/Sema6d/Slit2	8
GO:0032303	regulation of icosanoid secretion	8/1242	27/18303	0.000305	0.003318	0.002269	Acsl4/Il1a/Il1b/P2ry2/Pla2r1/Ptges/Syk/Trpv4	8
GO:0090280	positive regulation of calcium ion import	8/1242	27/18303	0.000305	0.003318	0.002269	Agtr1a/Cask/Ccl2/Cxcl12/Cxcr4/Lgals3/Pdgfb/Trpv3	8
GO:0032615	interleukin-12 production	13/1242	64/18303	0.000309	0.003347	0.002289	Cd40/Cmklr1/Il23a/Irf1/Lgals9/Nfkb1/Nod2/Plcb1/Rel/Syk/Thbs1/Tlr2/Tlr3	13
GO:0001706	endoderm formation	12/1242	56/18303	0.00031	0.003347	0.002289	Col11a1/Col5a1/Col7a1/Dusp5/Gata6/Hmga2/Hnf1b/Inhba/Itga5/Itga7/Lamb3/Mmp9	12
GO:0043331	response to dsRNA	12/1242	56/18303	0.00031	0.003347	0.002289	Ciita/Flot1/Grin2a/Irf1/Nfkb1/Nfkbia/Nod2/Peli1/Riok3/Ripk2/Stat1/Tlr3	12
GO:0050873	brown fat cell differentiation	12/1242	56/18303	0.00031	0.003347	0.002289	Arl4a/Bmp7/Dusp10/Fabp4/Flcn/Lamb3/Ppargc1a/Ptgs2/Rarres2/Scd/Sh2b2/Trpv4	12
GO:0007416	synapse assembly	27/1242	195/18303	0.00032	0.003455	0.002363	Bdnf/Bsn/Cadm1/Clstn2/Cntn5/Dnm3/Drd1/Ephb2/Ephb3/Erc2/Flrt3/Fzd5/Lrfn1/Mef2c/Nectin1/Nptxr/Nrg1/Nrxn3/Obsl1/Pclo/Pdgfb/Plxnd1/Sema4a/Shank1/Thbs2/Tlr2/Wnt5a	27
GO:0007435	salivary gland morphogenesis	10/1242	41/18303	0.000322	0.00346	0.002366	Bmp7/Edar/Esrp2/Fgfr2/Ntn4/Pdgfb/Plxnd1/Snai2/Tgm2/Tnf	10
GO:0032735	positive regulation of interleukin-12 production	10/1242	41/18303	0.000322	0.00346	0.002366	Cd40/Il23a/Irf1/Lgals9/Nod2/Plcb1/Rel/Syk/Tlr2/Tlr3	10
GO:0006959	humoral immune response	32/1242	247/18303	0.000323	0.003468	0.002372	Bcl3/C1rl/Camp/Ccl20/Ccl22/Cd69/Cfh/Colec11/Cxcl1/Cxcl10/Cxcl11/Cxcl12/Cxcl2/Cxcl3/Il1b/Acod1/LOC103689965/Lgals3/Mef2c/Nod2/Pglyrp1/Pglyrp4/Rarres2/Rbpj/Rgcc/Serping1/Tfeb/Tnf/Tslp/Wfdc18/Wfdc21/Xc	32
GO:0090036	regulation of protein kinase C signaling	7/1242	21/18303	0.000325	0.003478	0.002378	Adgrv1/Akap12/Cd40/Dgkg/Flt4/Sez6l/Wnt5a	7
GO:1901741	positive regulation of myoblast fusion	7/1242	21/18303	0.000325	0.003478	0.002378	Cd53/Cxcl12/Ehd2/Ripor2/Flot1/Flt3lg/Gdf15	7
GO:0051924	regulation of calcium ion transport	36/1242	290/18303	0.000328	0.003503	0.002396	Adcyap1r1/Adora2a/Adra2a/Agtr1a/Ank2/Bdkrb1/Bmp4/Cacna1g/Cacnb2/Cask/Casq1/Ccl2/Ccl3/Ccl5/Ccr1/Cemip/Coro1a/Cx3cl1/Cxcl10/Cxcl11/Cxcl12/Cxcr4/Drd1/Itgb3/Lgals3/Mylk/Ngf/P2ry6/Pde4b/Pdgfb/Ptgs2/Rgs9/Ryr2/Trpc4/Trpv3/Xcl1	36
GO:0009595	detection of biotic stimulus	9/1242	34/18303	0.00033	0.003503	0.002396	Lbp/Naip6/Nlrc4/Nod2/Pglyrp1/Pglyrp4/Ssc5d/Tlr2/Tlr6	9
GO:0010758	regulation of macrophage chemotaxis	9/1242	34/18303	0.00033	0.003503	0.002396	Ccl2/Ccl5/Cmklr1/Csf1/Cx3cl1/Rarres2/Thbs1/Tnfsf18/Trpv4	9
GO:0048841	regulation of axon extension involved in axon guidance	9/1242	34/18303	0.00033	0.003503	0.002396	Cxcl12/Plxna3/Sema3f/Sema4a/Sema4b/Sema5a/Sema6a/Sema6d/Wnt5a	9
GO:0014031	mesenchymal cell development	16/1242	90/18303	0.000332	0.003507	0.002399	Bmp4/Bmp7/Ednrb/Foxc1/Gbx2/Hand2/Hey2/Kitlg/Sema3f/Sema4a/Sema4b/Sema5a/Sema6a/Sema6d/Snai2/	16

ID	Description	GeneRatio	BgRatio	pvalue	p.adjust	qvalue	geneID	Count
GO:0043154	negative regulation of cysteine-type endopeptidase activity involved in apoptotic process	16/1242	90/18303	0.000332	0.003507	0.002399	Adora2a/Aqp1/Birc3/Cd44/Cflar/Dhcr24/Fnip1/Hspa1b/Mical1/Mmp9/Naip6/Plaur/Ptgs2/Serpinb9/Thbs1/Tnf	16
GO:0045685	regulation of glial cell differentiation	16/1242	90/18303	0.000332	0.003507	0.002399	Aspa/Bmp2/Bmp4/Clcf1/Cxcr4/Dusp10/Enpp2/Epha4/Gpr37l1/Hmga2/Il1b/Ldlr/Lif/Tenm4/Tlr2/Tnfrsf1b	16
GO:0006022	aminoglycan metabolic process	18/1242	108/18303	0.000333	0.003511	0.002401	B3gnt9/Cd44/Cemip/Chst14/Dcn/Foxc1/Has2/Hyal1/Il15/Il1b/Mamdc2/Ndnf/Nfkb1/Pdgfb/Pglyrp1/Pglyrp4/Ptger4/Xylt1	18
GO:0051235	maintenance of location	40/1242	334/18303	0.000334	0.003516	0.002405	Ank2/Ank3/Ankrd13c/Atp7b/B4galnt1/Bdkrb1/Casq1/Ccl3/Cemip/Coro1a/Cpt1a/Cx3cl1/Cxcl10/Cxcl11/Drd1/Hilpda/Hk2/Htr2a/Il1b/Insig1/Itgb3/Lgals9/Mcoln2/Mcoln3/Nfkbia/Nfkbib/Ngf/P2ry6/Plin2/Ppard/Ryr2/Ryr3/Scin/Sqle/Stard4/Thra/Tnf/Ttc39b/Twf2/Xcl1	40
GO:2001234	negative regulation of apoptotic signaling pathway	31/1242	237/18303	0.000336	0.003523	0.00241	Bdkrb2/Bdnf/Bmf/Bmp4/Cd44/Cflar/Col2a1/Creb3l1/Csf2/Cth/Cx3cl1/Cxcl12/Gas1/Gclc/Gstp1/Hspa1b/Ier3/Il19/Il1b/Itprip/Lgals3/Mmp9/Ngf/Nrg1/Plaur/Ppif/Ptgs2/Snai1/Snai2/Tnf/Tnfaip3	31
GO:0042116	macrophage activation	17/1242	99/18303	0.000336	0.003523	0.00241	Csf2/Cx3cl1/Il33/Jun/Lbp/Ldlr/Lgals9/Nampt/Pla2g5/Slc7a2/Syk/Thbs1/Tlr2/Tlr3/Tlr6/Tnf/Wnt5a	17
GO:0031346	positive regulation of cell projection organization	54/1242	493/18303	0.00034	0.003557	0.002432	Ankrd1/Aqp1/Bdnf/Bmp4/Bmp5/Bmp7/Cask/Cdc42ep5/Cdh4/Cflar/Cobl/Cx3cl1/Cxcl12/Cxcr4/Dnm3/Elavl4/Enpp2/Epha4/Ripor2/Fez1/Fscn1/Fuz/Fzd4/Htr7/Itga2/LOC691418/Lif/Lrp8/Map6/Metrn/Ndnf/Ngf/Nrg1/Obsl1/P2ry2/Palm/Pcp4/Plxna2/Plxna3/Plxnd1/Ptpn5/Rgma/Ror1/Scarf1/Sema5a/Sgk1/Shank1/Tnik/Tox/Tubb2b/Twf2/Wnt5a/Zdhhc15/Zeb2	54
GO:0034614	cellular response to reactive oxygen species	28/1242	206/18303	0.000341	0.00356	0.002435	Akr1b1/Aqp1/Casp12/Cfh/Cflar/Cyp1b1/Ern1/Ets1/F3/Fbln5/Gch1/Gpr37/Gpr37l1/Hk3/Il18rap/Jun/Klf2/Klf6/Mmp9/Net1/Nqo1/Ppif/Pycr1/Myb/Sirpa/Thbs1/Tnf/Tnf	28
GO:0003231	cardiac ventricle development	22/1242	146/18303	0.000342	0.003566	0.002439	Bmp4/Col11a1/Cxcr4/Fgfr2/Foxc1/Fzd2/Gja5/Hand2/Hey2/Kcnk2/Mef2c/Nrg1/Pax8/Pde2a/Prdm1/Rbm15/Rbpj/Ryr2/Slit2/Slit3/Tbx3/Wnt5a	22
GO:0042509	regulation of tyrosine phosphorylation of STAT protein	14/1242	73/18303	0.000344	0.003576	0.002446	Ccl5/Cd40/Clcf1/Csf2/Il15/Il23a/Il31ra/Irf1/Lif/Ptger4/Socs3/Tnf/Tnfsf18/Tslp	14
GO:0051155	positive regulation of striated muscle cell differentiation	14/1242	73/18303	0.000344	0.003576	0.002446	Bmp4/Cd53/Cxcl12/Cyp26b1/Ehd2/Ripor2/Fdps/Flot1/Flt3lg/Gdf15/Mef2c/Nrg1/Rbpj/Thra	14
GO:0033673	negative regulation of kinase activity	32/1242	248/18303	0.000347	0.003592	0.002457	Adora2a/Bmp4/Bmp7/Drd1/Dusp10/Dusp16/Dusp19/Dusp5/Dusp6/Ephb2/Errfi1/Fabp4/Gadd45g/Gstp1/Hmgcr/Il1b/Itprip/Pcp4/Pik3ip1/Prkar2a/Prkar2b/Ptpn5/Ptprb/Ptpro/Ptprt/Pycard/Rgs14/Serpinb3/Sfrp1/Smcr8/Spry4/	32
GO:0002526	acute inflammatory response	20/1242	127/18303	0.000348	0.003592	0.002457	Adora1/Adora2a/Ass1/Bdkrb2/Ccl5/Cxcl1/Ednrb/F3/Hfe/Il1a/Il1b/Il1rn/Il31ra/Lbp/Nlrp3/Ptges/Ptgs2/Serpinb9/Tnf/Vcam1	20
GO:0003014	renal system process	20/1242	127/18303	0.000348	0.003592	0.002457	Adora1/Adora2a/Agtr1a/Akr1b1/Anpep/Aqp1/Bdkrb1/Bmp4/Coro2b/Ednrb/Gja5/Has2/Htr7/Npr3/Pcsk5/Pdgfb/Ptger4/Ptpro/Serpinf2/Wnk4	20
GO:0019216	regulation of lipid metabolic process	45/1242	391/18303	0.000358	0.003696	0.002528	Abcd1/Acsl3/Adora1/Adra2a/Agtr1a/Angptl4/Bmp2/Bmp5/Cpt1a/Cyp27b1/Cyp51/Egr1/Erfe/Fabp3/Fabp5/Fdps/Fgr/Flt1/Fmo5/Hpgd/Htr2a/Il1a/Il1b/Insig1/Ldlr/Nfkb1/Hcar2/Nod2/Pdgfb/Pik3ip1/Pik3r5/Ppard/Ppargc1a/Ptgs2/Rab38/Rarres2/Snai1/Snai2/Socs3/Star/Stard4/Thra/Thrb/Tnf/Ttc39b	45
GO:0071470	cellular response to osmotic stress	12/1242	57/18303	0.000368	0.00379	0.002592	Abcb1a/Akr1b1/Aqp1/Bdkrb2/Errfi1/Lrrc8c/Lrrc8d/Mylk/Ptgs2/Relb/Slc6a12/Trpv4	12
GO:0002263	cell activation involved in immune response	35/1242	281/18303	0.00037	0.003805	0.002603	Ada/Anxa3/Bcl3/Ccl3/Cd180/Cd40/Clcf1/Clec4e/Coro1a/Dll1/Dock10/Ercc1/Exosc6/Fgr/Gata2/Il13ra2/Il23a/Il33/Itgal/Lbp/Lgals3/Lgals9/Myo1f/Nlrp3/Pglyrp1/Pglyrp4/Ptger4/Pycard/Myb/Rasgrp1/Relb/Ripk2/Sema4a/Sy	35
GO:0070838	divalent metal ion transport	54/1242	495/18303	0.000375	0.003849	0.002633	Adcyap1r1/Adora2a/Adra2a/Agtr1a/Ank2/Atp2a3/Bdkrb1/Bmp4/Cacna1g/Cacnb2/Calcrl/Cask/Casq1/Ccl2/Ccl3/Ccl5/Ccr1/Cemip/Coro1a/Cx3cl1/Cxcl10/Cxcl11/Cxcl12/Cxcr4/Cyp27b1/Drd1/Grin2a/Htr2a/Itgb3/Kcnn4/Lgals3/Mcoln2/Mcoln3/Mylk/Nalcn/Ngf/Nipal1/P2ry6/Pde4b/Pdgfb/Ptgs2/Myb/Ramp2/Rgs9/Ryr2/Ryr3/Slc24a3/Slc24a4/Slc25a25/Slc35g1/Trpc4/Trpv3/Trpv4/Xcl1	54
GO:0030111	regulation of Wnt signaling pathway	37/1242	303/18303	0.000377	0.003864	0.002642	Ankrd6/Bmp2/Cdh3/Cmahp/Col1a1/Cthrc1/Ctnnd2/Egr1/Fgf9/Fgfr2/Frzb/Fuz/Fzd4/Gli1/Hmga2/Hnf1b/Lgr5/Mdfi/Nfkb1/Notum/Plpp3/Prickle1/Ptpro/Rbpj/Rnf43/Rspo1/Rspo4/Sema5a/Sfrp1/Snai2/Tlr2/Tmem88/Tnfaip3/Tnn/Trabd2b/Wnt5a/Zeb2	37
GO:0033688	regulation of osteoblast	8/1242	28/18303	0.000401	0.004093	0.002799	Bmp2/Cthrc1/Fbln5/Hpse/Itgb3/Npr3/Sfrp1/Tnn	8
GO:0048843	negative regulation of axon extension involved in axon guidance	8/1242	28/18303	0.000401	0.004093	0.002799	Plxna3/Sema3f/Sema4a/Sema4b/Sema5a/Sema6a/Sema6d/Wnt5a	8

ID	Description	GeneRatio	BgRatio	pvalue	p.adjust	qvalue	geneID	Count
GO:0001936	regulation of endothelial cell proliferation	21/1242	138/18303	0.000404	0.00411	0.002811	Agtr1a/Bmp2/Bmp4/Ccl2/Cxcl12/F3/Flt1/Flt4/Gata2/Itgb3/Jun/Mef2c/Pdgfb/Pgf/Rgcc/Sema5a/Stat1/Thbs1/Thbs4/Tnf/Wnt5a	21
GO:0008360	regulation of cell shape	22/1242	148/18303	0.000415	0.004196	0.002869	Arap3/Bves/Ccl2/Ccl3/Cdc42ep5/Coro1a/F11r/Fgr/Gas7/Gna13/Itga7/LOC691418/Palm/Palmd/Plxna2/Plxna3/Plxnd1/Rhobtb1/Rhoj/Rhou/Sema4a/Strip2	22
GO:0042476	odontogenesis	22/1242	148/18303	0.000415	0.004196	0.002869	Adamts4/Aqp1/Bmp2/Bmp4/Bmp7/Col1a1/Csf1/Dlx3/Edar/Fam20c/Fgfr2/Foxc1/Gas1/Gata6/Hand2/Inhba/Klk4/Nectin1/Pax9/Relt/Slc24a4/Tnfrsf11b	22
GO:0030224	monocyte differentiation	9/1242	35/18303	0.000418	0.004196	0.002869	Bmp4/Csf1/Csf2/Gpr68/Il31ra/Jun/Mef2c/Pde1b/Pde2a	9
GO:0030574	collagen catabolic process	9/1242	35/18303	0.000418	0.004196	0.002869	Mmp1/Mmp10/Mmp12/Mmp13/Mmp16/Mmp1b/Mmp3/Mmp9/Vsir	9
GO:0042749	regulation of circadian sleep/wake	9/1242	35/18303	0.000418	0.004196	0.002869	Ada/Adora1/Adora2a/Csf2/Drd1/Hcrtr2/Htr7/Pglyrp1/Ptger4	9
GO:0090022	regulation of neutrophil	9/1242	35/18303	0.000418	0.004196	0.002869	Dapk2/Ripor2/Il1b/Il23a/Lbp/Nod2/Slit2/Thbs4/Xcl1	9
GO:1903131	mononuclear cell differentiation	9/1242	35/18303	0.000418	0.004196	0.002869	Bmp4/Csf1/Csf2/Gpr68/Il31ra/Jun/Mef2c/Pde1b/Pde2a	9
GO:0060349	bone morphogenesis	18/1242	110/18303	0.00042	0.004196	0.002869	Bmp4/Col13a1/Col1a1/Col2a1/Cyp26b1/Dhrs3/Fgfr2/Foxc1/Frem1/Has2/Insig1/Mef2c/Mmp13/Mmp16/Pax1/Scx/Thbs1/Trpv4	18
GO:0043367	CD4-positive, alpha-beta T cell differentiation	15/1242	83/18303	0.00042	0.004196	0.002869	Bcl3/Cd83/Il23a/Lgals9/Nlrp3/Pax1/Ptger4/Myb/Relb/Ripk2/Rsad2/Satb1/Sema4a/Tnfsf18/Tox	15
GO:0045844	positive regulation of striated muscle tissue development	15/1242	83/18303	0.00042	0.004196	0.002869	Bmp4/Cyp26b1/Dll1/Fdps/Fgf9/Fgfr2/Flot1/Gata6/Gli1/Hey2/Hmgcr/Mef2c/Ncam1/Nrg1/Rbpj	15
GO:0048636	positive regulation of muscle organ development	15/1242	83/18303	0.00042	0.004196	0.002869	Bmp4/Cyp26b1/Dll1/Fdps/Fgf9/Fgfr2/Flot1/Gata6/Gli1/Hey2/Hmgcr/Mef2c/Ncam1/Nrg1/Rbpj	15
GO:0051047	positive regulation of secretion	50/1242	451/18303	0.000421	0.004196	0.002869	A1cf/Aacs/Acsl3/Acsl4/Adcy8/Adora1/Adora2a/Agtr1a/Ankrd1/Aqp1/C1qtnf1/C1qtnf3/Cacna1g/Cacnb2/Cadm1/Cask/Clec4e/Cxcl12/Ednrb/Fgr/Frmd4a/Gata2/Gpr68/Hfe/Il1a/Il1b/Il33/Kcnn4/Lgals3/Lif/Mmp13/Hcar2/P2ry2/Pcp4/Pcsk1/Pla2r1/Ppard/Prl8a9/Ptger4/Ptges/Myb/Rab3d/Rgcc/Sybu/Syk/Tlr2/Tnf/Tnfsf15/Trpv4/Vsnl1	50
GO:0045861	negative regulation of proteolysis	45/1242	394/18303	0.000424	0.004216	0.002883	Adora2a/Ambp/Anxa8/Aqp1/Birc3/Cd44/Cflar/Derl3/Dhcr24/Efna1/Epha4/Fnip1/Furin/Gas1/Hfe/Hipk2/Hspa1b/LOC103689965/Lxn/Mical1/Mmp9/Naip6/Ngf/Plat/Plaur/Ppp1r15a/Ptgs2/Rybp/Serpina3n/Serpinb10/Serpinb2/Serpinb3/Serpinb3a/Serpinb6b/Serpinb9/Serpinf2/Serping1/Tfpi2/Thbs1/Timp3/Tnf/Wfdc1/Wfdc18/Wfdc21/	45
GO:0031650	regulation of heat generation	6/1242	16/18303	0.000427	0.004232	0.002894	Arrdc3/Ccl5/Ednrb/Il1b/Ptgs2/Tnf	6
GO:0060973	cell migration involved in heart	6/1242	16/18303	0.000427	0.004232	0.002894	Bmp4/Bmp7/Bves/Flrt3/Hand2/Snai2	6
GO:0070423	nucleotide-binding oligomerization domain containing signaling pathway	6/1242	16/18303	0.000427	0.004232	0.002894	Hspa1b/Nfkbia/Nod2/Inava/Ripk2/Tnfaip3	6
GO:0051208	sequestering of calcium ion	20/1242	129/18303	0.000429	0.004238	0.002899	Ank2/Atp7b/Bdkrb1/Casq1/Ccl3/Cemip/Coro1a/Cx3cl1/Cxcl10/Cxcl11/Drd1/Htr2a/Itgb3/Mcoln2/Mcoln3/Ngf/P2ry6/Ryr2/Ryr3/Xcl1	20
GO:0072577	endothelial cell apoptotic process	12/1242	58/18303	0.000436	0.004296	0.002938	Angptl4/Bmp4/Ccl2/Cd40/Gata2/Ndnf/Ramp2/Rgcc/Sema5a/Thbs1/Tnf/Tnfaip3	12
GO:1902930	regulation of alcohol biosynthetic process	12/1242	58/18303	0.000436	0.004296	0.002938	Adcyap1r1/Bmp2/Bmp5/Cyp27b1/Cyp51/Fdps/Il1b/Nfkb1/P2ry6/Plek/Pth1r/Tnf	12
GO:0032310	prostaglandin secretion	7/1242	22/18303	0.000449	0.004403	0.003011	Acsl4/Il1a/Il1b/Nos2/P2ry2/Ptges/Trpv4	7
GO:0072215	regulation of metanephros development	7/1242	22/18303	0.000449	0.004403	0.003011	Bmp4/Egr1/Fat4/Hnf1b/Lif/Pax8/Pdgfb	7
GO:0072677	eosinophil migration	7/1242	22/18303	0.000449	0.004403	0.003011	Ccl2/Ccl3/Ccl5/Cx3cl1/Dapk2/Lgals3/Ptger4	7
GO:0016055	Wnt signaling pathway	47/1242	418/18303	0.000453	0.004433	0.003032	Ankrd6/Bmp2/Ccnd1/Cd44/Cdh3/Cmahp/Col1a1/Cpz/Cthrc1/Ctnnd2/Egr1/Fgf9/Fgfr2/Frzb/Fuz/Fzd2/Fzd4/Fzd5/Fzd8/Gli1/Hmga2/Hnf1b/Lgr5/Mdfi/Mgat3/Ndrg2/Nfkb1/Notum/Plpp3/Prickle1/Ptpro/Rbpj/Rnf43/Ror1/Rspo1/Rspo4/Sema5a/Sfrp1/Snai2/Tlr2/Tmem88/Tnfaip3/Tnn/Trabd2b/Wnt2b/Wnt5a/Zeb2	47
GO:0071241	cellular response to inorganic substance	38/1242	317/18303	0.000457	0.00446	0.003051	A3galt2/Adcy8/Adgrv1/Alox15/Ank3/Aqp1/Atp7b/Blm/Casp12/Cflar/Cpne4/Cpne8/Cyp11b2/Enpp2/Ern1/Fabp4/Glra1/Grin2a/Hfe/Hvcn1/Jun/Junb/Kcnh1/Kcnk3/Mef2c/Mmp1/Mmp9/Mylk/Ncam1/Nos2/Nqo1/Ppargc1a/Ppif/Ptgs2/Star/Thbs1/Txnrd1/Wnt5a	38
GO:0031620	regulation of fever generation	5/1242	11/18303	0.000467	0.004505	0.003081	Ccl5/Ednrb/Il1b/Ptgs2/Tnf	5
GO:0032621	interleukin-18 production	5/1242	11/18303	0.000467	0.004505	0.003081	Gbp5/Nlrp3/Nod2/Tlr2/Tnf	5

ID	Description	GeneRatio	BgRatio	pvalue	p.adjust	qvalue	geneID	Count
GO:0032825	positive regulation of natural killer cell differentiation	5/1242	11/18303	0.000467	0.004505	0.003081	Flt3lg/Il15/Il15ra/Rasgrp1/Tox	5
GO:0061299	retina vasculature morphogenesis in camera-type eye	5/1242	11/18303	0.000467	0.004505	0.003081	Col4a1/Cyp1b1/Fzd4/Rhoj/Slc4a7	5
GO:0086016	AV node cell action potential	5/1242	11/18303	0.000467	0.004505	0.003081	Ank2/Cacna1g/Cacnb2/Gja5/Ryr2	5
GO:0086027	AV node cell to bundle of His cell signaling	5/1242	11/18303	0.000467	0.004505	0.003081	Ank2/Cacna1g/Cacnb2/Gja5/Ryr2	5
GO:2000389	regulation of neutrophil extravasation	5/1242	11/18303	0.000467	0.004505	0.003081	Cd99l2/Ripor2/Il1a/Il1r1/Ptger4	5
GO:0032370	positive regulation of lipid transport	15/1242	84/18303	0.00048	0.004628	0.003165	Acat2/C1qtnf1/Erfe/Fabp3/Il1a/Il1b/Lipg/Nfkbia/P2ry2/Pla2r1/Pltp/Ptges/Myb/Tnf/Trpv4	15
GO:2001237	negative regulation of extrinsic apoptotic signaling pathway	17/1242	102/18303	0.000482	0.004635	0.00317	Bmp4/Cflar/Col2a1/Csf2/Cx3cl1/Gas1/Gclc/Gstp1/Hspa1b/Il19/Il1b/Itprip/Lgals3/Nrg1/Snai2/Tnf/Tnfaip3	17
GO:0010951	negative regulation of endopeptidase activity	35/1242	285/18303	0.000482	0.004635	0.00317	Adora2a/Ambp/Anxa8/Aqp1/Birc3/Cd44/Cflar/Dhcr24/Fnip1/Furin/Hspa1b/LOC103689965/Lxn/Mical1/Mmp9/Naip6/Ngf/Plaur/Ptgs2/Serpina3n/Serpinb10/Serpinb2/Serpinb3/Serpinb3a/Serpinb6b/Serpinb9/Serpinf2/Serping1/Tfpi2/Thbs1/Timp3/Tnf/Wfdc1/Wfdc18/Wfdc21	35
GO:0045137	development of primary sexual characteristics	39/1242	329/18303	0.000483	0.004636	0.003171	Abcb1a/Adcyap1r1/Bmp4/Capn5/Casp12/Ccnd1/Col9a3/Csmd1/Cyp1b1/Ercc1/Ereg/Fdps/Fgf9/Foxc1/Fzd4/Gata6/Hmga2/Hmgcs1/Il1a/Inha/Inhba/Kif18a/Kitlg/Map7/Mmp1/Mmp13/Nefh/Pdgfb/Ptger4/Sfrp1/Slit2/Slit3/Spr2d/Star/Tiparp/Tlr3/Tlr5/Wnt2b/Wnt5a	39
GO:0042745	circadian sleep/wake cycle	10/1242	43/18303	0.000486	0.004654	0.003183	Ada/Adora1/Adora2a/Csf2/Drd1/Hcrtr2/Htr7/Pglyrp1/Ptger4/Star	10
GO:0002065	columnar/cuboidal epithelial cell differentiation	22/1242	150/18303	0.000501	0.004774	0.003265	Bmp2/Bmp4/Bmp5/Clic5/Dll1/Dlx3/Fam20c/Ripor2/Fgfr2/Gata6/Hey2/Il31ra/Mcoln3/Mycl/Myo7a/Nkx3-2/Prdm1/Rbpj/Rfx3/Slc4a7/Whrn/Wnt5a	22
GO:0006633	fatty acid biosynthetic process	22/1242	150/18303	0.000501	0.004774	0.003265	Abcd1/Acsl3/Acss1/Alox15/Bdkrb1/Elovl4/Fa2h/Fabp5/Fasn/Gstp1/Hacd4/Il1b/Insig1/Lipg/Liph/Mgll/Olah/Prkab2/Ptges/Ptgs2/Scd/Thnsl2	22
GO:0045598	regulation of fat cell differentiation	22/1242	150/18303	0.000501	0.004774	0.003265	Bmp2/Bmp7/Ccdc85b/Cmklr1/Dusp10/Flcn/Frzb/Gata2/Htr2a/Insig1/Medag/Noct/Ppard/Ptgs2/Rarres2/Sfrp1/Snai2/Tnf/Trib2/Trpv4/Wnt5a/Zbtb7c	22
GO:0071385	cellular response to glucocorticoid stimulus	19/1242	121/18303	0.000502	0.004774	0.003265	Abcb1a/Agtr1a/Aqp1/Ass1/Bmp4/Cacna1g/Ccl2/Cflar/Crebrf/Cyp1b1/Errfi1/Gjb2/Gstp1/Plat/Serpina3n/Sgk1/Star/Ucn2/Ugt1a6	19
GO:0008217	regulation of blood pressure	28/1242	211/18303	0.000505	0.004788	0.003274	Adora1/Agtr1a/Anpep/Bdkrb1/Bdkrb2/Coro2b/Cyp11b2/Dll1/Ednrb/F11r/Gch1/Gja5/Gpr37l1/Ier3/Lvrn/Nampt/Nos2/Nox1/Npr3/Npy1r/Pcsk5/Pdgfb/Ptgs2/Ptpro/Ramp2/Rarres2/Serpinf2/Tnf	28
GO:1901861	regulation of muscle tissue development	24/1242	170/18303	0.000505	0.004788	0.003274	Bdnf/Bmp2/Bmp4/Col14a1/Cyp26b1/Dll1/Fdps/Fgf9/Fgfr2/Flcn/Flot1/Gata6/Gli1/Hdac9/Hey2/Hmgcr/Igfbp5/Kcnk2/Mef2c/Ncam1/Nrg1/Pi16/Ppargc1a/Rbpj	24
GO:0042531	positive regulation of tyrosine phosphorylation of STAT protein	12/1242	59/18303	0.000514	0.004865	0.003327	Ccl5/Cd40/Clcf1/Csf2/Il15/Il23a/Il31ra/Lif/Ptger4/Tnf/Tnfsf18/Tslp	12
GO:0060412	ventricular septum morphogenesis	11/1242	51/18303	0.000515	0.004865	0.003327	Bmp4/Fgfr2/Fzd2/Gja5/Hey2/Rbm15/Rbpj/Slit2/Slit3/Tbx3/Wnt5a	11
GO:0019933	cAMP-mediated signaling	27/1242	201/18303	0.00052	0.004872	0.003332	Adcy8/Adcyap1r1/Adgrd1/Adora2a/Adra2a/Arrdc3/Calcrl/Cxcl10/Cxcl11/Drd1/Gna13/Gpr4/Htr7/Lgr5/Pclo/Pde2a/Pde4b/Prkar2a/Prkar2b/Ptger1/Ptger4/Ptgir/Pth1r/Ramp2/Tcp11/Tshr/Ucn2	27
GO:0071305	cellular response to vitamin D	8/1242	29/18303	0.000521	0.004872	0.003332	Cyp27b1/Fzd2/Il15/Mmp1/Nos2/Penk/Sfrp1/Snai2	8
GO:0071677	positive regulation of mononuclear cell migration	8/1242	29/18303	0.000521	0.004872	0.003332	Ccl2/Ccl5/Ccr1/Cxcl10/Cxcl12/Lgals3/Pla2g7/Tnfsf18	8
GO:0072012	glomerulus vasculature development	8/1242	29/18303	0.000521	0.004872	0.003332	Aqp1/Bmp4/Bmp7/Cflar/Egr1/Gpr4/Itgb3/Pdgfb	8
GO:0033032	regulation of myeloid cell apoptotic process	9/1242	36/18303	0.000524	0.004872	0.003332	Ccl5/Cd44/Kitlg/Mef2c/Hcar2/Nod2/Pik3cb/Slc7a11/Thra	9
GO:0050802	circadian sleep/wake cycle, sleep	9/1242	36/18303	0.000524	0.004872	0.003332	Ada/Adora1/Adora2a/Csf2/Drd1/Htr7/Pglyrp1/Ptger4/Star	9
GO:1901099	negative regulation of signal transduction in absence of ligand	9/1242	36/18303	0.000524	0.004872	0.003332	Col2a1/Csf2/Cx3cl1/Gas1/Hspa1b/Il1b/Nrg1/Snai2/Tnf	9
GO:2001240	negative regulation of extrinsic apoptotic signaling pathway in absence of ligand	9/1242	36/18303	0.000524	0.004872	0.003332	Col2a1/Csf2/Cx3cl1/Gas1/Hspa1b/Il1b/Nrg1/Snai2/Tnf	9

ID	Description	GeneRatio	BgRatio	pvalue	p.adjust	qvalue	geneID	Count
GO:0001959	regulation of cytokine-mediated signaling pathway	18/1242	112/18303	0.000525	0.004872	0.003332	Ccl5/Crebrf/Csf1/Cxcr4/Hspa1b/Il1r1/Il1rn/Irak2/Mmp12/Nlrc5/Pycard/Ripk2/Slit2/Slit3/Syk/Trim6/Tslp/Wnt5a	18
GO:0006720	isoprenoid metabolic process	18/1242	112/18303	0.000525	0.004872	0.003332	Acat2/Akr1b1/Cyp1b1/Cyp26b1/Cyp2j4/Dhrs3/Fdft1/Fdps/Hmgcr/Hmgcs1/Idi1/Mvd/Phyh/Ppard/Rarres2/Rbp1/Rdh5/Star	18
GO:0007229	integrin-mediated signaling pathway	18/1242	112/18303	0.000525	0.004872	0.003332	Adamts15/Col3a1/Fgr/Ibsp/Isg15/Itga11/Itga2/Itga5/Itga7/Itgal/Itgax/Itgb3/Itgbl1/Pcsk5/Plek/Plpp3/Slc2a10/Sy	18
GO:0198738	cell-cell signaling by wnt	47/1242	421/18303	0.00053	0.004915	0.003362	Ankrd6/Bmp2/Ccnd1/Cd44/Cdh3/Cmahp/Col1a1/Cpz/Cthrc1/Ctnnd2/Egr1/Fgf9/Fgfr2/Frzb/Fuz/Fzd2/Fzd4/Fzd5/Fzd8/Gli1/Hmga2/Hnf1b/Lgr5/Mdfi/Mgat3/Ndrg2/Nfkb1/Notum/Plpp3/Prickle1/Ptpro/Rbpj/Rnf43/Ror1/Rspo1/Rspo4/Sema5a/Sfrp1/Snai2/Tlr2/Tmem88/Tnfaip3/Tnn/Trabd2b/Wnt2b/Wnt5a/Zeb2	47
GO:0007548	sex differentiation	44/1242	387/18303	0.000537	0.004971	0.0034	Abcb1a/Adcyap1r1/Bmp4/Bmp5/Capn5/Casp12/Ccnd1/Col9a3/Csmd1/Cyp1b1/Dhcr24/Dmrt2/Ercc1/Ereg/Fdps/Fgf9/Foxc1/Fzd4/Gata6/Hmga2/Hmgcs1/Hnf1b/Il1a/Inha/Inhba/Kif18a/Kitlg/Map7/Mmp1/Mmp13/Nefh/Pdgfb/Ptger4/Sfrp1/Slit2/Slit3/Sprr2d/Star/Tbx3/Tiparp/Tlr3/Tlr5/Wnt2b/Wnt5a	44
GO:0051056	regulation of small GTPase mediated signal transduction	30/1242	233/18303	0.000543	0.005015	0.00343	Adcyap1r1/Arap3/Arhgdib/Arhgef25/Arhgef3/Cadm4/Chn1/Col3a1/Csf1/Ephb2/F11r/Ripor2/Flcn/Flot1/Gpr4/Kitlg/Net1/Ngf/Nrg1/Psd3/RGD1560455/Ralgapa2/Ralgps1/Rap1gap/Rasa2/Rasal3/Rasgrp1/Sh2b2/Slit2/Spry4	30
GO:0010977	negative regulation of neuron projection development	25/1242	181/18303	0.000544	0.005015	0.00343	Cspg4/Dnm3/Efna1/Epha4/Ephb2/Fat3/Il15ra/Nr2f1/Plk2/Plxna3/Pmp22/Rbpj/Rgma/Rtn4rl2/Sema3f/Sema4a/Sema4b/Sema5a/Sema6a/Sema6d/Slit2/Trpv4/Vim/Wnt	25
GO:0014032	neural crest cell development	15/1242	85/18303	0.000547	0.005029	0.003439	Bmp4/Bmp7/Ednrb/Foxc1/Gbx2/Hand2/Kitlg/Sema3f/Sema4a/Sema4b/Sema5a/Sema6a/Sema6d/Snai2/Zeb2	15
GO:0097755	positive regulation of blood vessel diameter	15/1242	85/18303	0.000547	0.005029	0.003439	Adora1/Adora2a/Adra2a/Bdkrb2/Cx3cl1/Dock4/Drd1/Dusp5/Ednrb/Gch1/Gja5/Hspa1b/Ppard/Tnf/Trpv4	15
GO:0032675	regulation of interleukin-6 production	23/1242	161/18303	0.000553	0.005075	0.003471	C1qtnf3/Cd200r1/Ereg/Vegfd/Il1a/Il1b/Il33/Klf2/Lbp/Lgals9/Nod2/Pycard/Inava/Ripk2/Sirpa/Syk/Tlr2/Tlr3/Tnf/Tnfaip3/Trpv4/Tslp/Wnt5a	23
GO:0060759	regulation of response to cytokine	19/1242	122/18303	0.000557	0.005108	0.003494	Ccl5/Crebrf/Csf1/Cxcr4/Hspa1b/Il1r1/Il1rn/Irak2/Mmp12/Nlrc5/Pycard/Ripk2/Slit2/Slit3/Syk/Tlr2/Trim6/Tslp/	19
GO:2001233	regulation of apoptotic signaling pathway	47/1242	422/18303	0.000559	0.005112	0.003496	Adora2a/Atf3/Bdkrb2/Bdnf/Bmf/Bmp4/Cd44/Cflar/Col2a1/Creb3l1/Csf2/Cth/Cx3cl1/Cxcl12/Dapk2/Flcn/Gas1/Gclc/Gstp1/Hip1r/Hspa1b/Ier3/Il19/Il1b/Inhba/Itprip/Lgals3/Lgals9/Mal/Mmp9/Ngf/Nox1/Nrg1/Plaur/Pmp22/Ppif/Prr7/Ptgs2/Pycard/Sfrp1/Snai1/Snai2/Thbs1/Timp3/Tnf/Tnfaip3/Traf1	47
GO:0043491	protein kinase B signaling	27/1242	202/18303	0.000563	0.005132	0.00351	C1qtnf1/C1qtnf3/Ccl2/Ccl3/Ccl5/Cd40/Csf2/Csf3/Cx3cl1/F3/Fam110c/Flcn/Gdf15/Hpse/Igfbp5/Il1b/Mmp3/Mst1r/Nrg1/Pik3c2b/Pik3r5/Cavin3/Sema5a/Sesn3/Thbs1/Tmem100/Tnf	27
GO:0051099	positive regulation of binding	27/1242	202/18303	0.000563	0.005132	0.00351	Bdnf/Bmp2/Bmp4/Csf3/Cthrc1/Epha4/Ripor2/Flot1/Foxc1/Gpsm1/Hand2/Hfe/Hip1r/Hipk2/Itga2/Larp6/Lgals9/Mmp9/Ngf/Plaur/Plk2/Plxnd1/Ramp2/Ripk2/Spon1/Tri	27
GO:0043588	skin development	32/1242	255/18303	0.000568	0.005174	0.003538	Arrdc3/Cdh3/Col1a1/Col3a1/Col5a1/Cyp26b1/Cyp27b1/Dact2/Dhcr24/Dll1/Edar/Errfi1/Fa2h/Fgfr2/Foxc1/Foxn1/Fuz/Macroh2a2/Hpse/Igfbp5/Il1a/Inhba/Itga2/Lgr5/Nsdhl/Rbpj/Scd/Slc2a10/Snai1/St14/Tnf/Wnt5a	32
GO:0034698	response to gonadotropin	13/1242	68/18303	0.000572	0.005203	0.003559	Cyp1b1/Egr1/Gata6/Gclc/Gjb2/Hmgcs1/Inhba/Pax8/Pcsk5/Pdgfb/Plat/Ppargc1a/Star	13
GO:0030258	lipid modification	33/1242	266/18303	0.000576	0.005232	0.003578	A3galt2/Abcd1/Acat2/Acat2l1/Agtr1a/Alox15/B4galnt1/Cpt1a/Cpt1b/Dgkg/Ech1/Echdc2/Efr3b/Fa2h/Fabp3/Hadh/Mboat1/Mtmr10/Mtmr11/Mtmr7/Phyh/Pik3c2b/Pik3cb/Pip5k1b/Pla2g7/Plpp3/Ppard/Ppargc1a/Scd/Socs3/St3gal2/Stard4/Pip4p1	33
GO:0021782	glial cell development	20/1242	132/18303	0.000581	0.005263	0.003599	Adora2a/Aspa/Cspg5/Dll1/Drd1/Fa2h/Gstp1/Il1b/Ldlr/Mxra8/Myrf/Nrg1/Pdgfb/Pmp22/Prdm8/Ror1/Sh3tc2/Tenm4/Tlr2/Vim	20
GO:0043279	response to alkaloid	25/1242	182/18303	0.000591	0.005325	0.003642	Abcb1a/Ada/Adcy8/Adora2a/Blm/Ccl5/Cpt1a/Cxcr4/Drd1/Egr1/Elavl4/Grin2a/Htr2a/Il1b/Mmp1/Ncam1/Nefh/Nqo1/Pcsk1/Penk/Plau/Ppargc1a/Ppp1r15a/Ryr2/Star	25
GO:0048010	vascular endothelial growth factor receptor signaling	10/1242	44/18303	0.000591	0.005325	0.003642	Cadm4/Ccl2/Emilin1/Fgf9/Vegfd/Flt1/Flt4/Foxc1/Fzd4/Pgf	10
GO:0055023	positive regulation of cardiac muscle tissue growth	10/1242	44/18303	0.000591	0.005325	0.003642	Fdps/Fgf9/Fgfr2/Gata6/Gli1/Hey2/Mef2c/Ncam1/Nrg1/Rbpj	10
GO:1902895	positive regulation of pri-miRNA transcription by RNA polymerase II	10/1242	44/18303	0.000591	0.005325	0.003642	Bmp2/Bmp4/Egr1/Ets1/Fosl1/Gata2/Jun/Nfkb1/Pdgfb/Tnf	10
GO:0060976	coronary vasculature development	12/1242	60/18303	0.000603	0.005417	0.003705	Adamts6/Bmp4/Fuz/Gata6/Hand2/Hey2/Pcsk5/Pde2a/Plxnd1/Prdm1/Prickle1/Sgcd	12

ID	Description	GeneRatio	BgRatio	pvalue	p.adjust	qvalue	geneID	Count
GO:1990874	vascular associated smooth muscle cell proliferation	12/1242	60/18303	0.000603	0.005417	0.003705	Ern1/Fgf9/Gstp1/Hpgd/Igfbp5/Jun/Mef2c/Mmp9/P2ry6/Pcsk5/Pdgfb/Tnf	12
GO:0007260	tyrosine phosphorylation of STAT protein	14/1242	77/18303	0.000607	0.005435	0.003717	Ccl5/Cd40/Clcf1/Csf2/Il15/Il23a/Il31ra/Irf1/Lif/Ptger4/Socs3/Tnf/Tnfsf18/Tslp	14
GO:0010595	positive regulation of endothelial cell migration	17/1242	104/18303	0.000607	0.005435	0.003717	Angpt4/Anxa3/Bmp4/Cd40/Ets1/Flt4/Gata2/Hdac9/Itgb3/Pdgfb/Plk2/Plpp3/Ptgs2/Rhoj/Sema5a/Thbs1/Wnt5a	17
GO:0090026	positive regulation of monocyte chemotaxis	7/1242	23/18303	0.000608	0.005435	0.003717	Ccl2/Ccl5/Ccr1/Cxcl10/Cxcl12/Pla2g7/Tnfsf18	7
GO:0072009	nephron epithelium development	19/1242	123/18303	0.000618	0.005504	0.003764	Agtr1a/Aqp1/Bmp2/Bmp4/Cd44/Dll1/Fat4/Foxc1/Hnf1b/Irx3/Lif/Mef2c/Npnt/Pax8/Pdgfb/Pgf/Ptpro/Wnk4/Wn	19
GO:0030279	negative regulation of ossification	16/1242	95/18303	0.000621	0.005504	0.003764	Areg/Ccl3/Ccr1/Chrd/Gfra4/Hand2/Igfbp5/Mef2c/Noct/P2ry2/Rbpj/Rorb/Sfrp1/Tnf/Tnn/Twist2	16
GO:0003281	ventricular septum development	15/1242	86/18303	0.000622	0.005504	0.003764	Bmp4/Cxcr4/Fgfr2/Fzd2/Gja5/Hey2/Pax8/Pde2a/Prdm1/Rbm15/Rbpj/Slit2/Slit3/Tbx3/Wnt5a	15
GO:0060411	cardiac septum morphogenesis	15/1242	86/18303	0.000622	0.005504	0.003764	Bmp4/Bmp5/Bmp7/Dhrs3/Fgfr2/Fzd2/Gata6/Gja5/Hey2/Rbm15/Rbpj/Slit2/Slit3/Tbx3/Wnt5a	15
GO:0035872	nucleotide-binding domain, leucine rich repeat containing receptor signaling pathway	6/1242	17/18303	0.000623	0.005504	0.003764	Hspa1b/Nfkbia/Nod2/Inava/Ripk2/Tnfaip3	6
GO:0090185	negative regulation of kidney development	6/1242	17/18303	0.000623	0.005504	0.003764	Bmp4/Bmp7/Flcn/Hnf1b/Mmp9/Pax8	6
GO:0090192	regulation of glomerulus development	6/1242	17/18303	0.000623	0.005504	0.003764	Bmp4/Bmp7/Cflar/Egr1/Itgb3/Pdgfb	6
GO:0014706	striated muscle tissue development	49/1242	448/18303	0.000648	0.005698	0.003897	Ankrd1/Atf3/Bdnf/Bmp2/Bmp4/Bmp5/Bmp7/Btg2/Bves/Casq1/Cflar/Col11a1/Col14a1/Cyp26b1/Dcn/Dll1/Egr1/Ripor2/Fdps/Fgf9/Fgfr2/Flot1/Foxc1/Gata6/Gja5/Gli1/Hdac9/Hey2/Hmgcr/Itga7/Kcnk2/Maff/Mef2c/Meox2/Ncam1/Nrg1/Pi16/Plagl1/Pmp22/Rbpj/Ryr2/Scx/Sgcd/Sgcg/Sorbs2/Tbx3/Tenm4/Wnt5a/Xirp1	49
GO:0010831	positive regulation of myotube differentiation	9/1242	37/18303	0.000651	0.005698	0.003897	Cd53/Cxcl12/Cyp26b1/Ehd2/Ripor2/Flot1/Flt3lg/Gdf15/Thra	9
GO:0030728	ovulation	9/1242	37/18303	0.000651	0.005698	0.003897	Ereg/Hpgd/Il1b/Il1r1/Inhba/Plat/Plau/Ptgs2/Tnfaip6	9
GO:0071402	cellular response to lipoprotein particle stimulus	9/1242	37/18303	0.000651	0.005698	0.003897	Ccl2/Ccl5/Cd68/F3/Hmgcs1/Ldlr/Mmp9/Syk/Tlr6	9
GO:2000108	positive regulation of leukocyte apoptotic process	9/1242	37/18303	0.000651	0.005698	0.003897	Ccl5/Cd274/Cd44/Fnip1/Lgals9/Mef2c/Hcar2/Pik3cb/Wnt5a	9
GO:0050764	regulation of phagocytosis	18/1242	114/18303	0.000652	0.005698	0.003897	Alox15/Ccl2/Fgr/Gata2/Il15/Il15ra/Il1b/Itga2/Lbp/Nod2/Pla2g5/Pycard/Rap1gap/Sirpa/Syk/Tlr2/Tnf/Tub	18
GO:1905330	regulation of morphogenesis of an epithelium	18/1242	114/18303	0.000652	0.005698	0.003897	Agtr1a/Ankrd6/Bmp4/Bmp7/Cthrc1/Cxcl10/Fzd2/Hnf1b/Itgax/Lif/Ntn4/Pax8/Jhy/Sfrp1/Snai2/Tnf/Wnt2b/Wnt5a	18
GO:0003206	cardiac chamber morphogenesis	21/1242	143/18303	0.000654	0.005698	0.003897	Bmp4/Bmp5/Bmp7/Col11a1/Dhrs3/Fgfr2/Foxc1/Fzd2/Gata6/Gja5/Hand2/Hey2/Mef2c/Nrg1/Rbm15/Rbpj/Ryr2/Slit2/Slit3/Tbx3/Wnt5a	21
GO:0007189	adenylate cyclase-activating G protein-coupled receptor signaling pathway	21/1242	143/18303	0.000654	0.005698	0.003897	Adcy8/Adgrd1/Adora2a/Adra2a/Arrdc3/Calcrl/Cxcl10/Cxcl11/Drd1/Gna13/Gpr4/Htr7/Lgr5/Pde4b/Ptger1/Ptger4/Ptgir/Pth1r/Ramp2/Tshr/Ucn2	21
GO:0019730	antimicrobial humoral response	21/1242	143/18303	0.000654	0.005698	0.003897	Bcl3/Camp/Ccl20/Ccl22/Colec11/Cxcl1/Cxcl10/Cxcl11/Cxcl12/Cxcl2/Cxcl3/Acod1/Lgals3/Nod2/Pglyrp1/Pglyrp4/Rarres2/Tslp/Wfdc18/Wfdc21/Xcl1	21
GO:0008406	gonad development	38/1242	323/18303	0.000656	0.005704	0.003901	Abcb1a/Bmp4/Capn5/Casp12/Ccnd1/Col9a3/Csmd1/Cyp1b1/Ercc1/Ereg/Fdps/Fgf9/Foxc1/Fzd4/Gata6/Hmga2/Hmgcs1/Il1a/Inha/Inhba/Kif18a/Kitlg/Map7/Mmp1/Mmp13/Nefh/Pdgfb/Ptger4/Sfrp1/Slit2/Slit3/Sprr2d/Star/Tiparp/Tlr3/Tlr5/Wnt2b/Wnt5a	38
GO:0048511	rhythmic process	44/1242	391/18303	0.000667	0.005786	0.003957	Abcb1a/Ada/Adora1/Adora2a/Arntl2/Ass1/Atp7b/Bdnf/Bmp2/Capn5/Casp12/Csf2/Cyp1b1/Cyp7b1/Drd1/Egr1/Enpp2/Ereg/Ets1/Fzd4/Grin2a/Has2/Hcrtr2/Hnf1b/Htr7/Inhba/Jun/Mmp1/Mmp13/Nampt/Ngf/Noct/Nos2/Pglyrp1/Ppargc1a/Cavin3/Ptger4/Relb/Rorb/Slit2/Slit3/Star/Tn	44
GO:0042634	regulation of hair cycle	8/1242	30/18303	0.000669	0.00579	0.00396	Cdh3/Fa2h/Foxn1/Hpse/Inhba/Tnf/Trpv3/Wnt5a	8
GO:0090025	regulation of monocyte chemotaxis	8/1242	30/18303	0.000669	0.00579	0.00396	Ccl2/Ccl5/Ccr1/Cxcl10/Cxcl12/Pla2g7/Slit2/Tnfsf18	8
GO:0035282	segmentation	17/1242	105/18303	0.00068	0.005875	0.004018	Bmp4/Cobl/Dll1/Dmrt2/Foxc1/Fzd5/Irx3/Mafb/Meox2/Pax1/Plxna2/Rbpj/Sema3f/Sfrp1/Tbx3/Wnt5a/Zeb2	17
GO:2000116	regulation of cysteine-type endopeptidase activity	31/1242	247/18303	0.000686	0.005917	0.004047	Adora2a/Aqp1/Atp2a3/Birc3/Casp12/Cd44/Cflar/Dhcr24/Egln3/F3/Fnip1/Hip1r/Hspa1b/Lgals9/Mical1/Mmp9/Naip6/Ngf/Nlrc4/Nlrp3/Plaur/Prr7/Ptgs2/Pycard/Ripk2/Serpinb9/Stat1/Syk/Thbs1/Tnf/Tnfsf15	31

ID	Description	GeneRatio	BgRatio	pvalue	p.adjust	qvalue	geneID	Count
GO:0006766	vitamin metabolic process	14/1242	78/18303	0.000694	0.005956	0.004074	Clybl/Cyp26b1/Cyp27a1/Cyp27b1/Gclc/Gsto2/Il1b/Nfkb1/Pltp/Ppard/Rbp1/Snai1/Snai2/Tnf	14
GO:0014855	striated muscle cell proliferation	14/1242	78/18303	0.000694	0.005956	0.004074	Cflar/Fgf9/Fgfr2/Foxc1/Gata6/Gli1/Hey2/Kcnk2/Mef2c/Ncam1/Nrg1/Ppard/Rbpj/Tenm4	14
GO:0038034	signal transduction in absence of ligand	14/1242	78/18303	0.000694	0.005956	0.004074	Bcl2a1/Col2a1/Csf2/Cx3cl1/Gas1/Hspa1b/Il1a/Il1b/Inhba/Kitlg/Ngf/Nrg1/Snai2/Tnf	14
GO:0097192	extrinsic apoptotic signaling pathway in absence of ligand	14/1242	78/18303	0.000694	0.005956	0.004074	Bcl2a1/Col2a1/Csf2/Cx3cl1/Gas1/Hspa1b/Il1a/Il1b/Inhba/Kitlg/Ngf/Nrg1/Snai2/Tnf	14
GO:0071559	response to transforming growth factor beta	32/1242	258/18303	0.000696	0.005958	0.004075	Ankrd1/Ccl2/Ccl5/Cflar/Col1a1/Col3a1/Emilin1/Fgfr2/Flcn/Furin/Hipk2/Hpgd/Hspa1b/Htra3/Il1r1/Jun/Lpxn/Ltbp4/Mef2c/Npnt/Nrep/Pde2a/Peg10/Penk/Ppargc1a/Scx/Sfrp1/Slc2a10/Star/Thbs1/Wnt5a/Xcl1	32
GO:1904035	regulation of epithelial cell	16/1242	96/18303	0.000699	0.005976	0.004087	Angptl4/Bmp4/Ccl2/Cd40/Cflar/Gata2/Ndnf/Ngf/Pla2r1/Ppargc1a/Ramp2/Rgcc/Sema5a/Thbs1/Tnf/Tnfaip3	16
GO:0002699	positive regulation of immune effector process	33/1242	269/18303	0.000702	0.005983	0.004092	Cadm1/Ccl2/Cd40/Clcf1/Cxcl1/Exosc6/Fgr/Fzd5/Gata2/Hspa1b/Il13ra2/Il18rap/Il1b/Il1r1/Il23a/Il33/Lbp/Lgals9/Mr1/Nlrp3/Nod2/Pvr/Pla2g5/Myb/Rasgrp1/Ripk2/Rsad2/Syk/Tlr2/Tnf/Trim6/Wnt5a/Xcl1	33
GO:0034121	regulation of toll-like receptor signaling pathway	12/1242	61/18303	0.000706	0.005983	0.004092	Flot1/Irf1/Acod1/Lbp/Nod2/Peli1/Rsad2/Tlr2/Tlr3/Tlr5/Tlr6/Tnfaip3	12
GO:0044060	regulation of endocrine process	12/1242	61/18303	0.000706	0.005983	0.004092	Agtr1a/C1qtnf1/C1qtnf3/Gja5/Il1b/Inha/Inhba/Lif/Hcar2/Nrg1/Rab11fip1/Ucn2	12
GO:0050922	negative regulation of chemotaxis	12/1242	61/18303	0.000706	0.005983	0.004092	Ccl2/Gstp1/Plxna3/Sema3f/Sema4a/Sema4b/Sema5a/Sema6a/Sema6d/Slit2/Thbs1/Wnt5a	12
GO:0060038	cardiac muscle cell proliferation	12/1242	61/18303	0.000706	0.005983	0.004092	Fgf9/Fgfr2/Foxc1/Gata6/Gli1/Hey2/Kcnk2/Mef2c/Ncam1/Nrg1/Rbpj/Tenm4	12
GO:0032147	activation of protein kinase activity	34/1242	280/18303	0.000706	0.005983	0.004092	Adcy8/Adora1/Adra2a/Agtr1a/Angpt4/Bmp2/Bmp4/Cspg4/Dusp19/Ern1/Flt1/Gadd45g/Gdf15/Gprc5c/Gprc5d/Igfbp6/Il1b/Il23a/Itgb3/Map3k14/Map3k9/Mdfi/Ngf/Nrg1/Pdgfb/Inava/Rgcc/Ripk2/Syk/Thbs1/Tnf/Tnfsf15/Tni	34
GO:0010466	negative regulation of peptidase activity	35/1242	291/18303	0.000707	0.005983	0.004092	Adora2a/Ambp/Anxa8/Aqp1/Birc3/Cd44/Cflar/Dhcr24/Fnip1/Furin/Hspa1b/LOC103689965/Lxn/Mical1/Mmp9/Naip6/Ngf/Plaur/Ptgs2/Serpina3n/Serpinb10/Serpinb2/Serpinb3/Serpinb3a/Serpinb6b/Serpinb9/Serpinf2/Serping1/Tfpi2/Thbs1/Timp3/Tnf/Wfdc1/Wfdc18/Wfdc21	35
GO:0030308	negative regulation of cell growth	27/1242	205/18303	0.000709	0.005988	0.004095	Bdkrb1/Ccdc85b/Cth/Cyp27b1/Fhl1/Flcn/Frzb/Gas1/Hspa1b/Hyal1/Inhba/Osgin1/Pi16/Plxna3/Ppard/Rerg/Rgma/Sema3f/Sema4a/Sema4b/Sema5a/Sema6a/Sema6d/Sfrp1/Slit2/Slit3/Wnt5a	27
GO:0048863	stem cell differentiation	30/1242	237/18303	0.000721	0.006084	0.004161	Bmp4/Bmp7/Ednrb/Fgfr2/Foxc1/Frzb/Gata6/Gbx2/Ggt1/Hand2/Hmga2/Hnf1b/Kitlg/Lbh/Lif/Mef2c/Nrg1/Prickle1/Rbpj/Sema3f/Sema4a/Sema4b/Sema5a/Sema6a/Sema6d/Sfrp1/Snai2/Trim6/Vsir/Zeb2	30
GO:0009615	response to virus	36/1242	303/18303	0.00075	0.006297	0.004307	Abcc9/Bcl3/Ccl5/Cd40/Cxcl10/Hmga1/Hmga2/Hyal1/Ifi27l2b/Ifit3/Il15/Il1b/Il23a/Il33/Irf1/Acod1/Isg15/Itgax/Lgals9/Map3k14/Micb/Mmp12/Mst1r/Nlrc5/Nlrp3/Oasl/Penk/Pycard/Riok3/Rsad2/Slfn13/Stat1/Tlr3/Tnf/Trim6	36
GO:0001554	luteolysis	5/1242	12/18303	0.000755	0.006297	0.004307	Casp12/Mmp1/Mmp13/Slit2/Slit3	5
GO:0016338	calcium-independent cell-cell adhesion via plasma membrane cell-adhesion molecules	5/1242	12/18303	0.000755	0.006297	0.004307	Bmp2/Cadm1/Cldn3/Cx3cl1/Ncam1	5
GO:0032490	detection of molecule of bacterial origin	5/1242	12/18303	0.000755	0.006297	0.004307	Lbp/Nod2/Ssc5d/Tlr2/Tlr6	5
GO:0036005	response to macrophage colony-stimulating factor	5/1242	12/18303	0.000755	0.006297	0.004307	Ccl2/Csf1/Pde1b/Pde2a/Tlr2	5
GO:0036006	cellular response to macrophage colony-stimulating factor stimulus	5/1242	12/18303	0.000755	0.006297	0.004307	Ccl2/Csf1/Pde1b/Pde2a/Tlr2	5
GO:0071863	regulation of cell proliferation in bone marrow	5/1242	12/18303	0.000755	0.006297	0.004307	Fgfr2/Flt3lg/Hmga2/Lgals3/Mef2c	5
GO:0006909	phagocytosis	29/1242	227/18303	0.000756	0.006297	0.004307	Alox15/Anxa3/Ccl2/Coro1a/Fgr/Gata2/Il15/Il15ra/Il1b/Itga2/Itgb3/Lbp/Ldlr/Mst1r/Myo7a/Nod2/P2ry6/Pla2g5/Pycard/Rab20/Rap1gap/Rhobtb1/Sirpa/Syk/Tgm2/Thbs1/Tlr2/Tnf/Tub	29
GO:0030282	bone mineralization	19/1242	125/18303	0.000757	0.006297	0.004307	Adgrv1/Alox15/Bmp2/Bmp4/Bmp7/Ccl3/Ccr1/Cyp27b1/Fam20c/Fgfr2/Fgr/Ibsp/Isg15/Mef2c/Mgp/Mmp13/Ptgs2/Pth1r/Slc24a3	19
GO:0051209	release of sequestered calcium ion into cytosol	19/1242	125/18303	0.000757	0.006297	0.004307	Ank2/Bdkrb1/Casq1/Ccl3/Cemip/Coro1a/Cx3cl1/Cxcl10/Cxcl11/Drd1/Htr2a/Itgb3/Mcoln2/Mcoln3/Ngf/P2ry6/Ryr2/Ryr3/Xcl1	19
GO:1905954	positive regulation of lipid localization	17/1242	106/18303	0.00076	0.006309	0.004315	Acat2/C1qtnf1/Erfe/Fabp3/Hilpda/Il1a/Il1b/Lipg/Nfkbia/P2ry2/Pla2r1/Plin2/Pltp/Ptges/Myb/Tnf/Trpv4	17

ID	Description	GeneRatio	BgRatio	pvalue	p.adjust	qvalue	geneID	Count
GO:0050777	negative regulation of immune response	23/1242	165/18303	0.000782	0.006489	0.004438	Alox15/Ccr1/Col3a1/Dusp10/Hfe/Il13ra2/Il33/Acod1/Lgals3/Lgals9/Mmp12/Nlrc5/Nod2/Pglyrp1/Pglyrp4/Serpinb3a/Serpinb9/Serping1/Tnf/Tnfaip3/Tnfsf18/Vsir/Xcl	23
GO:0014075	response to amine	14/1242	79/18303	0.000792	0.006552	0.004481	Adora2a/Ass1/Cxcl1/Cxcl2/Drd1/Egr1/Grin2a/Hdac9/Itga2/Nqo1/Pde1b/Ptpn5/Rgs7/Rgs9	14
GO:0048332	mesoderm morphogenesis	14/1242	79/18303	0.000792	0.006552	0.004481	Bmp4/Bmp7/Chrd/Fgfr2/Foxc1/Hmga2/Inhba/Itga2/Itgb3/Scx/Snai1/Tbx3/Txnrd1/Wnt5a	14
GO:0002703	regulation of leukocyte mediated immunity	32/1242	260/18303	0.000794	0.006557	0.004484	Cadm1/Ccl2/Cd40/Clcf1/Cxcl1/Exosc6/Fgr/Fzd5/Gata2/Hfe/Hspa1b/Il13ra2/Il18rap/Il1b/Il1r1/Il23a/Lgals9/Mr1/Nlrp3/Nod2/Pvr/Rasgrp1/Rsad2/Serpinb3a/Serpinb9/Syk/Tlr2/Tlr3/Tnf/Tnfrsf1b/Vsir/Xcl1	32
GO:0051149	positive regulation of muscle cell differentiation	15/1242	88/18303	0.000798	0.006577	0.004498	Bmp4/Cd53/Cth/Cxcl12/Cyp26b1/Ehd2/Ripor2/Fdps/Flot1/Flt3lg/Gdf15/Mef2c/Nrg1/Rbpj/Thra	15
GO:0002691	regulation of cellular extravasation	9/1242	38/18303	0.000802	0.006587	0.004505	Ccl2/Cd99l2/Cxcl12/Ripor2/Il1a/Il1r1/Plcb1/Ptger4/Selp	9
GO:0045684	positive regulation of epidermis development	9/1242	38/18303	0.000802	0.006587	0.004505	Bmp4/Cyp27b1/Foxc1/Foxn1/Macroh2a2/Hpse/Ppard/Tnf/Wnt5a	9
GO:1904707	positive regulation of vascular associated smooth muscle cell proliferation	9/1242	38/18303	0.000802	0.006587	0.004505	Ern1/Fgf9/Hpgd/Igfbp5/Jun/Mmp9/P2ry6/Pdgfb/Tnf	9
GO:0035634	response to stilbenoid	7/1242	24/18303	0.000809	0.006612	0.004522	Gsta2/Gsta5/Idi1/Ifit3/Il1b/Ppargc1a/Usp18	7
GO:0070233	negative regulation of T cell apoptotic process	7/1242	24/18303	0.000809	0.006612	0.004522	Ada/Blm/Bmp4/Ccl5/Dock8/Efna1/Serpinb9	7
GO:0070498	interleukin-1-mediated signaling	7/1242	24/18303	0.000809	0.006612	0.004522	Egr1/Il1a/Il1b/Il1r1/Il1rn/Irak2/Plcb1	7
GO:1904062	regulation of cation transmembrane transport	42/1242	372/18303	0.000818	0.006675	0.004565	Adra2a/Agtr1a/Ank2/Ank3/Bdkrb1/Bmp4/Cacnb2/Casq1/Ccl2/Cemip/Cnih2/Coro1a/Cx3cl1/Cxcl10/Cxcl11/Drd1/Ephb2/Fhl1/Grin2a/Hcn1/Itgb3/Kcnh2/Kcnip4/Lrrc38/Mef2c/Mmp9/Ngf/P2ry6/Pcsk9/Pde4b/Ppargc1a/Ppif/Prrt1/Rgs7/Rgs9/Ryr2/Scn2b/Shank1/Stom/Trpv4/Wnk	42
GO:0051283	negative regulation of sequestering of calcium ion	19/1242	126/18303	0.000836	0.006816	0.004661	Ank2/Bdkrb1/Casq1/Ccl3/Cemip/Coro1a/Cx3cl1/Cxcl10/Cxcl11/Drd1/Htr2a/Itgb3/Mcoln2/Mcoln3/Ngf/P2ry6/Ryr2/Ryr3/Xcl1	19
GO:0042102	positive regulation of T cell proliferation	17/1242	107/18303	0.000847	0.00687	0.004699	Blm/Card11/Ccl5/Cd274/Coro1a/Il15/Il1a/Il1b/Il23a/Ital/Lgals9/Pycard/Rasal3/Ripk2/Syk/Vcam1/Xcl1	17
GO:0006929	substrate-dependent cell migration	8/1242	31/18303	0.000849	0.00687	0.004699	Abcc9/Cspg4/Fbln1/Itga11/Itga2/Pdgfb/Slit2/Snai2	8
GO:0045187	regulation of circadian sleep/wake cycle, sleep	8/1242	31/18303	0.000849	0.00687	0.004699	Ada/Adora1/Adora2a/Csf2/Drd1/Htr7/Pglyrp1/Ptger4	8
GO:0061437	renal system vasculature development	8/1242	31/18303	0.000849	0.00687	0.004699	Aqp1/Bmp4/Bmp7/Cflar/Egr1/Gpr4/Itgb3/Pdgfb	8
GO:0061440	kidney vasculature development	8/1242	31/18303	0.000849	0.00687	0.004699	Aqp1/Bmp4/Bmp7/Cflar/Egr1/Gpr4/Itgb3/Pdgfb	8
GO:0031214	biomineral tissue development	23/1242	166/18303	0.000851	0.006873	0.0047	Adgrv1/Alox15/Bmp2/Bmp4/Bmp7/Ccl3/Ccr1/Col1a1/Cyp27b1/Fam20c/Fgfr2/Fgr/Hey2/Ibsp/Isg15/Mef2c/Mgp/Mmp13/Nectin1/Ptgs2/Pth1r/Slc20a1/Slc24a3	23
GO:0110148	biomineralization	23/1242	166/18303	0.000851	0.006873	0.0047	Adgrv1/Alox15/Bmp2/Bmp4/Bmp7/Ccl3/Ccr1/Col1a1/Cyp27b1/Fam20c/Fgfr2/Fgr/Hey2/Ibsp/Isg15/Mef2c/Mgp/Mmp13/Nectin1/Ptgs2/Pth1r/Slc20a1/Slc24a3	23
GO:0001975	response to amphetamine	11/1242	54/18303	0.000858	0.00691	0.004726	Adora2a/Cxcl1/Cxcl2/Drd1/Egr1/Grin2a/Hdac9/Pde1b/Ptpn5/Rgs7/Rgs9	11
GO:0042088	T-helper 1 type immune response	10/1242	46/18303	0.000858	0.00691	0.004726	Bcl3/Il18rap/Il1b/Il1r1/Il23a/Il33/Relb/Ripk2/Sema4a/Xcl1	10
GO:0001837	epithelial to mesenchymal transition	21/1242	146/18303	0.000862	0.006931	0.00474	Akna/Bmp2/Bmp4/Bmp7/Col1a1/Efna1/Fgfr2/Foxc1/Fuz/Has2/Hey2/Hmga2/Il1b/Rbpj/Rgcc/Serpinb3/Sfrp1/Snai1/Snai2/Tmem100/Wnt5a	21
GO:0016485	protein processing	29/1242	229/18303	0.00087	0.006987	0.004778	Birc3/C1rl/Casp12/Cpd/Cpz/Dhcr24/Ero1b/F3/Furin/Fuz/Gas1/Ggt1/LOC100910418/Meltf/Mmp16/Myrf/Nlrc4/Pcsk1/Pcsk5/Pcsk9/Plat/Plau/Ppp1r15a/Pycard/Ripk2/Serpinf2/Spon1/Thbs1/Vsir	29
GO:0045582	positive regulation of T cell differentiation	16/1242	98/18303	0.00088	0.00704	0.004815	Ada/Cd83/Dusp10/Ikzf1/Il1a/Il1b/Il23a/Lgals9/Lilrb4/Nlrp3/Myb/Rasgrp1/Ripk2/Sox12/Syk/Vsir	16
GO:2000117	negative regulation of cysteine-type endopeptidase	16/1242	98/18303	0.00088	0.00704	0.004815	Adora2a/Aqp1/Birc3/Cd44/Cflar/Dhcr24/Fnip1/Hspa1b/Mical1/Mmp9/Naip6/Plaur/Ptgs2/Serpinb9/Thbs1/Tnf	16
GO:0048245	eosinophil	6/1242	18/18303	0.000881	0.00704	0.004815	Ccl2/Ccl3/Ccl5/Cx3cl1/Dapk2/Lgals3	6
GO:0071560	cellular response to transforming growth factor beta stimulus	31/1242	251/18303	0.000897	0.007164	0.0049	Ankrd1/Ccl2/Ccl5/Cflar/Col1a1/Col3a1/Emilin1/Fgfr2/Flcn/Furin/Hipk2/Hpgd/Hspa1b/Htra3/Jun/Lpxn/Ltbp4/Mef2c/Npnt/Nrep/Pde2a/Peg10/Penk/Ppargc1a/Scx/Sfrp1/Slc2a10/Star/Thbs1/Wnt5a/Xcl1	31
GO:0061333	renal tubule morphogenesis	15/1242	89/18303	0.0009	0.007177	0.004909	Agtr1a/Bmp2/Bmp4/Cd44/Col4a1/Fat4/Hnf1b/Irx3/Lgr5/Mef2c/Npnt/Pax8/Pgf/Wnk4/Wnt2b	15

ID	Description	GeneRatio	BgRatio	pvalue	p.adjust	qvalue	geneID	Count
GO:0031669	cellular response to nutrient levels	31/1242	252/18303	0.000959	0.007626	0.005216	Acat2/Atf3/Bmf/Ccl5/Cd68/Col1a1/Col2a1/Cpeb4/Cyp11b2/Cyp27b1/Flcn/Fnip1/Fzd2/Hfe/Il15/Jun/Mmp1/Nampt/Nos2/Pcsk9/Penk/Pik3c2b/Ppp1r15a/Rragd/Sesn3/Sfrp1/Snai2/Tfeb/Ucn2/Upp1/Wnt2b	31
GO:0071375	cellular response to peptide hormone stimulus	42/1242	375/18303	0.000959	0.007626	0.005216	Adcy8/Agtr1a/Ass1/Ccl2/Cflar/Col2a1/Cpeb2/Cyp11b2/Cyp1b1/Dnai1/Drd1/Egr1/Erfe/Errfi1/Fzd2/Gclc/Gdf15/Gjb2/Gstp1/Hdac9/Il1b/Insig1/Irf1/Irs3/Itgb3/Mmp1/Nfkb1/Pcsk9/Pdgfb/Plat/Plcb1/Pnpla3/Ptpre/Rap1b/Rarres2/Sesn3/Sgk1/Sh2b2/Socs3/Star/Stat1/Tshr	42
GO:0038084	vascular endothelial growth factor signaling pathway	9/1242	39/18303	0.000981	0.007773	0.005316	Cadm4/Dcn/Dll1/Flt1/Flt4/Foxc1/Pgf/Ptp4a3/Sema6a	9
GO:0110111	negative regulation of animal organ morphogenesis	9/1242	39/18303	0.000981	0.007773	0.005316	Bmp4/Bmp7/Foxc1/Hnf1b/Pax8/Thrb/Tnf/Tnfrsf11b/Wnt5a	9
GO:1904064	positive regulation of cation transmembrane	24/1242	178/18303	0.000982	0.007773	0.005316	Agtr1a/Ank2/Ank3/Bdkrb1/Bmp4/Cacnb2/Casq1/Ccl2/Cemip/Cx3cl1/Cxcl10/Cxcl11/Drd1/Ephb2/Hcn1/Kcnh2/Lrrc38/P2ry6/Rgs7/Rgs9/Ryr2/Trpv4/Wnk4/Xcl1	24
GO:0051146	striated muscle cell differentiation	38/1242	330/18303	0.000983	0.007774	0.005317	Barx2/Bdnf/Bmp2/Bmp4/Bves/Casq1/Cd53/Cflar/Col14a1/Csrp2/Cxcl10/Cxcl12/Cyp26b1/Dll1/Ehd2/Ripor2/Fdps/Flot1/Flt3lg/Gata6/Gdf15/Hdac9/Hey2/Igfbp5/Mef2c/Nrg1/Pi16/Pmp22/Rbm38/Rbpj/Sgcd/Sorbs2/Tbx3/Thra/Tmod1/Tmod2/Tnnt3/Xirp1	38
GO:1901570	fatty acid derivative biosynthetic process	13/1242	72/18303	0.001006	0.00794	0.00543	Acsl1/Acsl4/Alox15/Bdkrb1/Elovl4/Fabp5/Ggt1/Il1b/Mgst3/Pla2g5/Ptges/Ptgs2/Syk	13
GO:0055025	positive regulation of cardiac muscle tissue development	11/1242	55/18303	0.001008	0.00795	0.005437	Bmp4/Fdps/Fgf9/Fgfr2/Gata6/Gli1/Hey2/Mef2c/Ncam1/Nrg1/Rbpj	11
GO:0051282	regulation of sequestering of calcium ion	19/1242	128/18303	0.001015	0.007983	0.00546	Ank2/Bdkrb1/Casq1/Ccl3/Cemip/Coro1a/Cx3cl1/Cxcl10/Cxcl11/Drd1/Htr2a/Itgb3/Mcoln2/Mcoln3/Ngf/P2ry6/Ryr2/Ryr3/Xcl1	19
GO:0071384	cellular response to corticosteroid stimulus	19/1242	128/18303	0.001015	0.007983	0.00546	Abcb1a/Agtr1a/Aqp1/Ass1/Bmp4/Cacna1g/Ccl2/Cflar/Crebrf/Cyp1b1/Errfi1/Gjb2/Gstp1/Plat/Serpina3n/Sgk1/Star/Ucn2/Ugt1a6	19
GO:0050766	positive regulation of phagocytosis	14/1242	81/18303	0.001023	0.008006	0.005475	Ccl2/Gata2/Il15/Il15ra/Il1b/Itga2/Lbp/Nod2/Pla2g5/Pycard/Rap1gap/Sirpa/Tnf/Tub	14
GO:0048738	cardiac muscle tissue development	31/1242	253/18303	0.001023	0.008006	0.005475	Ankrd1/Bmp2/Bmp4/Bmp5/Bmp7/Bves/Col11a1/Col14a1/Dll1/Fdps/Fgf9/Fgfr2/Foxc1/Gata6/Gja5/Gli1/Hey2/Kcnk2/Mef2c/Ncam1/Nrg1/Pi16/Rbpj/Ryr2/Sgcd/Sgcg/Sorbs2/Tbx3/Tenm4/Wnt5a/Xirp1	31
GO:0007431	salivary gland development	10/1242	47/18303	0.001025	0.008006	0.005475	Bmp7/Edar/Esrp2/Fgfr2/Ntn4/Pdgfb/Plxnd1/Snai2/Tgm2/Tnf	10
GO:0060421	positive regulation of heart growth	10/1242	47/18303	0.001025	0.008006	0.005475	Fdps/Fgf9/Fgfr2/Gata6/Gli1/Hey2/Mef2c/Ncam1/Nrg1/Rbpj	10
GO:0071312	cellular response to alkaloid	10/1242	47/18303	0.001025	0.008006	0.005475	Abcb1a/Adcy8/Blm/Ccl5/Egr1/Mmp1/Plau/Ppargc1a/Ryr2/Star	10
GO:0003176	aortic valve development	7/1242	25/18303	0.001058	0.008186	0.005599	Bmp4/Efna1/Emilin1/Rbpj/Slit2/Slit3/Tnfrsf1b	7
GO:0010894	negative regulation of steroid biosynthetic process	7/1242	25/18303	0.001058	0.008186	0.005599	Bmp2/Bmp5/Cyp27b1/Insig1/Nfkb1/Snai1/Snai2	7
GO:0033622	integrin activation	7/1242	25/18303	0.001058	0.008186	0.005599	Cx3cl1/Cxcl12/Pcsk5/Plek/Ptger4/Rap1b/Selp	7
GO:0034123	positive regulation of toll-like receptor signaling pathway	7/1242	25/18303	0.001058	0.008186	0.005599	Flot1/Lbp/Peli1/Rsad2/Tlr2/Tlr3/Tlr5	7
GO:0035743	CD4-positive, alpha-beta T cell cytokine production	7/1242	25/18303	0.001058	0.008186	0.005599	Il18rap/Il1b/Il1r1/Il31ra/Nlrp3/Rsad2/Xcl1	7
GO:0045939	negative regulation of steroid metabolic process	7/1242	25/18303	0.001058	0.008186	0.005599	Bmp2/Bmp5/Cyp27b1/Insig1/Nfkb1/Snai1/Snai2	7
GO:0046641	positive regulation of alpha-beta T cell proliferation	7/1242	25/18303	0.001058	0.008186	0.005599	Blm/Il23a/Lgals9/Rasal3/Ripk2/Syk/Xcl1	7
GO:1903532	positive regulation of secretion by cell	44/1242	400/18303	0.001063	0.008213	0.005617	A1cf/Aacs/Acsl4/Adcy8/Adora2a/Agtr1a/Ankrd1/C1qtnf1/C1qtnf3/Cacna1g/Cacnb2/Cadm1/Cask/Clec4e/Cxcl12/Fgr/Frmd4a/Gata2/Gpr68/Hfe/Il1a/Il1b/Il33/Kcnn4/Lgals3/Lif/Mmp13/Hcar2/P2ry2/Pcp4/Pcsk1/Ppard/Ptger4/Ptges/Myb/Rab3d/Rgcc/Sybu/Syk/Tlr2/Tnf/Tnfsf15/T	44
GO:0001964	startle response	8/1242	32/18303	0.001065	0.008218	0.00562	Adora2a/Csmd1/Glra1/Glrb/Grin2a/Kcnh1/Nrg1/Penk	8
GO:0002460	adaptive immune response based on somatic recombination of immune receptors built from immunoglobulin	36/1242	309/18303	0.00107	0.008245	0.005639	Ada/Bcl3/C1rl/Cd274/Cd40/Clcf1/Ercc1/Exosc6/Fzd5/Hfe/Hspa1b/Il13ra2/Il18rap/Il1b/Il1r1/Il23a/Il31ra/Il33/Mef2c/Mr1/Nfkb2/Nlrp3/Nod2/Pvr/Relb/Ripk2/Rsad2/Sema4a/Serpinb9/Serping1/Tnf/Tnfaip3/Tnfrsf1b/Tnfsf18/Vsir/Xcl1	36
GO:0014009	glial cell proliferation	12/1242	64/18303	0.001102	0.008471	0.005793	Areg/Csf1/Cx3cl1/Flt1/Il1b/Il33/Nrg1/Penk/Pmp22/Myb/Tnf/Vim	12

ID	Description	GeneRatio	BgRatio	pvalue	p.adjust	qvalue	geneID	Count
GO:1905207	regulation of cardiocyte differentiation	12/1242	64/18303	0.001102	0.008471	0.005793	Bmp2/Bmp4/Bmp7/Col14a1/Dll1/Fdps/Gata6/Mef2c/Nrg1/Pi16/Prickle1/Rbpj	12
GO:1904951	positive regulation of establishment of protein localization	44/1242	401/18303	0.001118	0.008579	0.005867	A1cf/Aacs/Acsl3/Acsl4/Adcy8/Adora2a/Agtr1a/Ank3/Ankrd1/C1qtnf3/Cadm1/Cask/Ccl2/Cemip/Clec4e/Coro2b/Crebrf/Fgr/Frmd4a/Fzd5/Gpr68/Il1a/Il33/Ipo5/Kcnn4/Mmp13/Hcar2/Pcsk1/Ppard/Ptger4/Ptgs2/Ptp4a3/Ptpn5/Rgcc/Rhou/Sec16b/Stom/Sybu/Syk/Tlr2/Tnf/Tnfsf15/Tr	44
GO:0032526	response to retinoic acid	21/1242	149/18303	0.001124	0.008618	0.005894	Aqp1/Bmp2/Bmp4/Ccl2/Col1a1/Col2a1/Cyp26b1/Drd1/Fgfr2/Gjb2/Hand2/Irf1/Map7/Mef2c/Micb/Mmp9/Ptges/Myb/Rorb/Tnf/Twf2	21
GO:0071887	leukocyte apoptotic process	20/1242	139/18303	0.001127	0.008625	0.005899	Ada/Blm/Bmp4/Ccl5/Cd274/Cd44/Cxcl12/Dock8/Efna1/Fnip1/Kitlg/Lgals3/Lgals9/Mef2c/Hcar2/Nod2/Pik3cb/Serpinb9/Slc7a11/Wnt5a	20
GO:0048708	astrocyte differentiation	15/1242	91/18303	0.001139	0.008698	0.005949	Adora2a/Bmp2/Clcf1/Dll1/Drd1/Epha4/Gpr37l1/Hmga2/Il1b/Ldlr/Lif/Nfix/Plpp3/Ror1/Vim	15
GO:0048864	stem cell development	15/1242	91/18303	0.001139	0.008698	0.005949	Bmp4/Bmp7/Ednrb/Foxc1/Gbx2/Hand2/Kitlg/Sema3f/Sema4a/Sema4b/Sema5a/Sema6a/Sema6d/Snai2/Zeb2	15
GO:0006029	proteoglycan metabolic process	13/1242	73/18303	0.001149	0.008749	0.005984	Bmp2/Chst14/Col11a1/Col2a1/Dcn/Extl3/Hpse/Mamdc2/Ndnf/Ndst3/Ppard/Slc2a10/Xylt1	13
GO:0009913	epidermal cell differentiation	24/1242	180/18303	0.001149	0.008749	0.005984	Bmp4/Cdh3/Clic5/Cyp26b1/Cyp27b1/Dll1/Errfi1/Fa2h/Ripor2/Foxc1/Foxn1/Gli1/Macroh2a2/Hey2/Il1a/Maff/Mcoln3/Mycl/Myo7a/Rbpj/Slc4a7/St14/Whrn/Wnt5a	24
GO:0031334	positive regulation of protein-containing complex assembly	30/1242	244/18303	0.001157	0.008779	0.006004	Alox15/Bmf/Cdc42ep5/Coro1a/Csf2/Csf3/Fnip1/Fnip2/Fscn1/Gbp5/Gda/Gmfg/Hip1r/Hspa1b/Ikzf1/LOC691418/Lgals3/Mmp1/Mmp3/Nrg1/Pcsk5/Plek/Pycard/Cracd/Rap1b/Scin/Syk/Tlr6/Tnf/Trabd2b	30
GO:0003093	regulation of glomerular filtration	5/1242	13/18303	0.001159	0.008779	0.006004	Adora1/Coro2b/Gja5/Pdgfb/Ptpro	5
GO:0071838	cell proliferation in bone marrow	5/1242	13/18303	0.001159	0.008779	0.006004	Fgfr2/Flt3lg/Hmga2/Lgals3/Mef2c	5
GO:0086067	AV node cell to bundle of His cell communication	5/1242	13/18303	0.001159	0.008779	0.006004	Ank2/Cacna1g/Cacnb2/Gja5/Ryr2	5
GO:0001657	ureteric bud development	17/1242	110/18303	0.001163	0.008795	0.006015	Agtr1a/Bdnf/Bmp2/Bmp4/Bmp7/Bmper/Cd44/Fat4/Fgfr2/Foxc1/Hnf1b/Npnt/Pax8/Pgf/Sfrp1/Slit2/Wnt2b	17
GO:0007626	locomotory behavior	31/1242	255/18303	0.001164	0.008795	0.006015	Adcy8/Adora2a/Als2/Arrdc3/Astn1/Cxcl12/Drd1/Egr1/Elavl4/Epha4/Fzd4/Glra1/Glrb/Gpr37/Gpr88/Hipk2/Hoxb8/Mcoln3/Myo15a/Ngf/Npy1r/Nrg1/Pde1b/Penk/Pmp22/Rcan2/Selenop/Sez6l/Sptbn4/Tmod1/Tshr	31
GO:0061900	glial cell activation	11/1242	56/18303	0.001179	0.008895	0.006083	Adora2a/Cx3cl1/Il1b/Il33/Jun/Ldlr/Nampt/Tlr2/Tlr3/Tlr6/Tnf	11
GO:0048771	tissue remodeling	27/1242	212/18303	0.001183	0.008913	0.006096	Abcb1b/Cspg4/Cthrc1/Elf3/Flt4/Foxc1/Gja5/Hand2/Igfbp5/Il1a/Il23a/Itgb3/Klf6/LOC24906/Lif/Mef2c/Mmp9/Nos2/Ptger4/Pth1r/Rab3d/Rbpj/Sfrp1/Syk/Tgm2/Thbs4/	27
GO:0090322	regulation of superoxide metabolic process	9/1242	40/18303	0.001191	0.008958	0.006127	Agtr1a/Cxcl1/Fbln5/Gch1/Gstp1/Hvcn1/Olr1/Syk/Tnf	9
GO:0002822	regulation of adaptive immune response based on somatic recombination of immune receptors built from immunoglobulin	25/1242	191/18303	0.001198	0.009004	0.006158	Ada/Cd274/Cd40/Clcf1/Exosc6/Fzd5/Hfe/Hspa1b/Il1b/Il1r1/Il23a/Il33/Mef2c/Mr1/Nlrp3/Nod2/Pvr/Ripk2/Rsad2/Tnf/Tnfaip3/Tnfrsf1b/Tnfsf18/Vsir/Xcl1	25
GO:0006970	response to osmotic stress	18/1242	120/18303	0.001204	0.009026	0.006173	Abcb1a/Agtr1a/Akr1b1/Aqp1/Bdkrb2/Ccl5/Cyp11b2/Errfi1/Itga2/Lrrc8c/Lrrc8d/Map7/Mylk/Pcp4/Ptgs2/Relb/Slc6a12/Trpv4	18
GO:0048678	response to axon injury	18/1242	120/18303	0.001204	0.009026	0.006173	Cspg5/Epha4/Flrt3/Jun/Kcnk2/Matn2/Naip6/Ncam1/Nefh/Nrep/Nrg1/Pcsk1/Pdgfb/Rgma/Scarf1/Txnrd1/Uck2/	18
GO:0002700	regulation of production of molecular mediator of immune response	22/1242	160/18303	0.001208	0.009044	0.006185	Cd40/Clcf1/Exosc6/Fzd5/Hfe/Il13ra2/Il1b/Il1r1/Il33/Nlrp3/Nod2/Rsad2/Siglec10/Tlr2/Tlr3/Tnf/Tnfrsf1b/Tril/Trim6/Vsir/Wnt5a/Xcl1	22
GO:0032306	regulation of prostaglandin secretion	6/1242	19/18303	0.001214	0.009062	0.006198	Acsl4/Il1a/Il1b/P2ry2/Ptges/Trpv4	6
GO:0050872	white fat cell differentiation	6/1242	19/18303	0.001214	0.009062	0.006198	Aacs/Fabp4/Pnpla3/Scd/Snai2/Wfdc21	6
GO:0046685	response to arsenic-containing substance	10/1242	48/18303	0.001217	0.009077	0.006208	Abcb1b/As3mt/Cpeb2/Cyp1b1/Gclc/Gsto2/Nefh/Ppif/Tnfrsf11b/Zfand2a	10
GO:0002440	production of molecular mediator of immune response	29/1242	234/18303	0.001225	0.009119	0.006237	Card11/Cd40/Clcf1/Ercc1/Exosc6/Fzd5/Hfe/Il13ra2/Il18rap/Il1b/Il1r1/Il31ra/Il33/Mr1/Nlrp3/Nod2/Inava/Rsad2/Siglec10/Tlr2/Tlr3/Tnf/Tnfrsf1b/Trem3/Tril/Trim6/Vsir/Wnt5a/Xcl1	29
GO:0072163	mesonephric epithelium development	17/1242	111/18303	0.001289	0.009572	0.006546	Agtr1a/Bdnf/Bmp2/Bmp4/Bmp7/Bmper/Cd44/Fat4/Fgfr2/Foxc1/Hnf1b/Npnt/Pax8/Pgf/Sfrp1/Slit2/Wnt2b	17
GO:0072164	mesonephric tubule development	17/1242	111/18303	0.001289	0.009572	0.006546	Agtr1a/Bdnf/Bmp2/Bmp4/Bmp7/Bmper/Cd44/Fat4/Fgfr2/Foxc1/Hnf1b/Npnt/Pax8/Pgf/Sfrp1/Slit2/Wnt2b	17

ID	Description	GeneRatio	BgRatio	pvalue	p.adjust	qvalue	geneID	Count
GO:0042908	xenobiotic transport	8/1242	33/18303	0.001323	0.009804	0.006705	Abcb1a/Abcb1b/Lrrc8c/Lrrc8d/Slc17a3/Slc22a4/Slc47a1/Slc47a2	8
GO:0060317	cardiac epithelial to mesenchymal transition	8/1242	33/18303	0.001323	0.009804	0.006705	Bmp2/Efna1/Has2/Hey2/Rbpj/Snai1/Snai2/Tmem100	8
GO:0031639	plasminogen	7/1242	26/18303	0.001364	0.010079	0.006893	Dhcr24/LOC100910418/Meltf/Plat/Plau/Serpinf2/Thbs1	7
GO:0034114	regulation of heterotypic cell-cell adhesion	7/1242	26/18303	0.001364	0.010079	0.006893	Alox15/Bmp7/Cd44/Flot1/Il1b/Il1rn/Tnf	7
GO:0046470	phosphatidylcholine metabolic process	11/1242	57/18303	0.001373	0.010132	0.00693	Acsl3/Chka/Enpp2/Fabp3/Fabp5/Plaat1/Ldlr/Pla2g2a/Pla2g5/Pla2g7/Rab38	11
GO:0060395	SMAD protein signal transduction	15/1242	93/18303	0.00143	0.010531	0.007202	Bmp2/Bmp3/Bmp4/Bmp5/Bmp7/Gdf15/Gdf6/Hipk2/Inha/Inhba/Inhbe/Jun/Rbpms/Slc2a10/Vim	15
GO:0001569	branching involved in blood vessel morphogenesis	9/1242	41/18303	0.001435	0.010531	0.007202	Col4a1/Cxcl12/Cxcr4/Flt1/Gbx2/Gna13/Plxnd1/Rbm15/Sema5a	9
GO:0033028	myeloid cell apoptotic process	9/1242	41/18303	0.001435	0.010531	0.007202	Ccl5/Cd44/Kitlg/Mef2c/Hcar2/Nod2/Pik3cb/Slc7a11/Thra	9
GO:0042398	cellular modified amino acid biosynthetic process	9/1242	41/18303	0.001435	0.010531	0.007202	Carns1/Chdh/Ckb/Gamt/Gch1/Gclc/Ggt1/Plod2/Slc7a11	9
GO:0003179	heart valve morphogenesis	10/1242	49/18303	0.001438	0.010531	0.007202	Bmp2/Bmp4/Efna1/Emilin1/Gja5/Hey2/Mef2c/Scx/Slit2/Slit3	10
GO:0033628	regulation of cell adhesion mediated by integrin	10/1242	49/18303	0.001438	0.010531	0.007202	Ada/Ccl5/Cyp1b1/Efna1/Itgb3/Lif/Lpxn/Plau/Snai2/Syk	10
GO:0006949	syncytium formation	12/1242	66/18303	0.001458	0.010664	0.007294	Cd44/Cd53/Cflar/Cxcl10/Cxcl12/Ehd2/Ercc1/Ripor2/Flot1/Flt3lg/Gdf15/Stat1	12
GO:0006809	nitric oxide biosynthetic process	14/1242	84/18303	0.001473	0.010733	0.007341	Ass1/Cyp1b1/Ddah2/Il1b/Klf2/LOC497963/Nos2/Ptgs2/Sirpa/Slc7a2/Tlr2/Tlr5/Tlr6/Tnf	14
GO:0003158	endothelium development	19/1242	132/18303	0.001474	0.010733	0.007341	Abcb1b/Bmp4/Cldn3/Cxcl10/Cxcr4/Dll1/F11r/Gja5/Hey2/Hoxb5/Il1b/Itgax/Nrg1/Pde2a/Pdgfb/Rap1b/Rbpj/Tmem100/Tnf	19
GO:0071901	negative regulation of protein serine/threonine kinase activity	19/1242	132/18303	0.001474	0.010733	0.007341	Bmp4/Bmp7/Dusp10/Dusp16/Dusp19/Dusp5/Dusp6/Gstp1/Hmgcr/Il1b/Prkar2a/Prkar2b/Ptpn5/Pycard/Rgs14/Serpinb3/Sfrp1/Spry4/Tnfaip3	19
GO:0002443	leukocyte mediated immunity	43/1242	395/18303	0.001477	0.010733	0.007341	Anxa3/Bcl3/C1rl/Cadm1/Ccl2/Ccl3/Cd40/Clcf1/Coro1a/Cxcl1/Ercc1/Exosc6/Fgr/Fzd5/Gata2/Hfe/Hspa1b/Il13ra2/Il18rap/Il1b/Il1r1/Il23a/Il31ra/Lgals9/Mr1/Myo1f/Nlrp3/Nod2/Pvr/Rasgrp1/Rnf19b/Rsad2/Serpinb3a/Serpinb9/Serping1/Syk/Tlr2/Tlr3/Tnf/Tnfrsf1b/Trem3/Vsir/Xc	43
GO:0006644	phospholipid metabolic process	43/1242	395/18303	0.001477	0.010733	0.007341	Acsl3/Alox15/Chka/Dgkg/Efr3b/Enpp2/Fabp3/Fabp5/Fdft1/Fdps/Fgr/Flt1/Gata6/Hmgcr/Hmgcs1/Plaat1/Htr2a/Idi1/Ldlr/Mtmr10/Mtmr11/Mtmr7/Mvd/Nod2/Pcsk9/Pdgfb/Pik3c2b/Pik3cb/Pik3ip1/Pik3r5/Pip5k1b/Pla2g2a/Pla2g4e/Pla2g5/Pla2g7/Plcb1/Plek/Plpp3/Pnpla3/Ppard/Rab38/Socs3/Pip4p1	43
GO:0001707	mesoderm formation	13/1242	75/18303	0.001487	0.010793	0.007381	Bmp4/Bmp7/Chrd/Fgfr2/Foxc1/Hmga2/Inhba/Itga2/Itgb3/Scx/Snai1/Txnrd1/Wnt5a	13
GO:0046890	regulation of lipid biosynthetic process	25/1242	194/18303	0.001495	0.010841	0.007415	Abcd1/Acsl3/Bmp2/Bmp5/Cyp27b1/Cyp51/Egr1/Fabp3/Fabp5/Fdps/Htr2a/Il1a/Il1b/Insig1/Ldlr/Nfkb1/Pdgfb/Ppargc1a/Ptgs2/Rab38/Snai1/Snai2/Star/Stard4/Tnf	25
GO:0071548	response to dexamethasone	16/1242	103/18303	0.001517	0.010987	0.007514	Abcb1a/Agtr1a/Aqp1/Ass1/Bmp4/Cacna1g/Ccl2/Cflar/Cyp1b1/Errfi1/Fibin/Gjb2/Il1b/Nos2/Plat/Star	16
GO:0046883	regulation of hormone secretion	36/1242	316/18303	0.00159	0.011499	0.007865	Aacs/Acsl4/Adcy8/Adora1/Adra2a/Agtr1a/C1qtnf1/C1qtnf3/Cask/Ccl5/Cpt1a/Gpr68/Hadh/Hfe/Hmga2/Hmgcr/Il11/Il1b/Inha/Inhba/Lif/Hcar2/Nos2/Npy1r/Nrg1/Pax8/Ppard/Ptger4/Myb/Rab11fip1/Rfx3/Sfrp1/Sybu/Tnf/Ucn2	36
GO:0061098	positive regulation of protein tyrosine kinase activity	11/1242	58/18303	0.001592	0.0115	0.007865	Adora1/Adra2a/Areg/Ccl5/Efna1/Epha4/Ereg/Lrp8/Nrg1/Pdgfb/Trpv4	11
GO:0051346	negative regulation of hydrolase activity	50/1242	479/18303	0.001594	0.011502	0.007866	Adora2a/Ambp/Angptl4/Anxa8/Aqp1/Birc3/Ccdc8/Cd44/Cflar/Cpeb2/Csrnp3/Dhcr24/F11r/Fnip1/Furin/Gpsm1/Hdac9/Hspa1b/LOC103689965/LOC497940/Lmtk2/Lxn/Mical1/Mmp9/Naip6/Ngf/Phactr1/Pla2r1/Plaur/Ppif/Ppp1r15a/Ptgs2/Serpina3n/Serpinb10/Serpinb2/Serpinb3/Serpinb3a/Serpinb6b/Serpinb9/Serpinf2/Serping1/Sh2d4a/Slit2/Tfpi2/Thbs1/Timp3/Tnf/Wfdc1/Wfdc18/Wfdc2	50
GO:0002250	adaptive immune response	42/1242	385/18303	0.0016	0.01151	0.007872	Ada/Alcam/Alox15/Bcl3/C1rl/Cd274/Cd40/Clcf1/Dusp10/Ercc1/Exosc6/Fzd5/Hfe/Hspa1b/Il13ra2/Il18rap/Il1b/Il1r1/Il23a/Il31ra/Il33/Mef2c/Mr1/Nfkb2/Nlrp3/Nod2/Pvr/Prr7/Pycard/Relb/Ripk2/Rsad2/Sema4a/Serpinb9/Serping1/Syk/Tnf/Tnfaip3/Tnfrsf1b/Tnfsf18/Vsir/Xcl1	42
GO:0051222	positive regulation of protein transport	42/1242	385/18303	0.0016	0.01151	0.007872	A1cf/Aacs/Acsl3/Acsl4/Adcy8/Adora2a/Agtr1a/Ank3/Ankrd1/C1qtnf3/Cadm1/Cask/Ccl2/Cemip/Clec4e/Crebrf/Fgr/Frmd4a/Fzd5/Gpr68/Il1a/Il33/Ipo5/Kcnn4/Mmp13/Hcar2/Pcsk1/Ppard/Ptger4/Ptgs2/Ptpn5/Rgcc/Rhou/Sec16b/Stom/Sybu/Syk/Tlr2/Tnf/Tnfsf15/Trpv4/Vsnl1	42

ID	Description	GeneRatio	BgRatio	pvalue	p.adjust	qvalue	geneID	Count
GO:0010770	positive regulation of cell morphogenesis involved in differentiation	25/1242	195/18303	0.001607	0.011539	0.007892	Bdnf/Cask/Cdh4/Cspg5/Cxcl12/Enpp2/Epha4/Has2/Itgb3/Lrp8/Map6/Metrn/Net1/Ngf/Nrg1/Obsl1/Plxna2/Plxna3/Plxnd1/Sema5a/Sgk1/Tnik/Tubb2b/Twf2/Zeb2	25
GO:0034767	positive regulation of ion transmembrane transport	25/1242	195/18303	0.001607	0.011539	0.007892	Abcb1a/Agtr1a/Ank2/Ank3/Bdkrb1/Bmp4/Cacnb2/Casq1/Ccl2/Cemip/Cx3cl1/Cxcl10/Cxcl11/Drd1/Ephb2/Hcn1/Kcnh2/Lrrc38/P2ry6/Rgs7/Rgs9/Ryr2/Trpv4/Wnk4/	25
GO:0008277	regulation of G protein-coupled receptor signaling pathway	19/1242	133/18303	0.001613	0.011561	0.007907	Ada/Arrdc3/Ccl5/Fzd2/Gpsm1/Itgb3/Klk14/LOC681355/Mgll/Pde4b/Plcb1/Plek/Ramp2/Rgs14/Rgs7/Rgs9/Tmod2/Tub/Wnt5a	19
GO:0045932	negative regulation of muscle contraction	8/1242	34/18303	0.001629	0.011649	0.007967	Adora1/Adra2a/Bdkrb1/Calcrl/Dock4/Ptger4/Ptgs2/Trpv4	8
GO:0048710	regulation of astrocyte	8/1242	34/18303	0.001629	0.011649	0.007967	Bmp2/Clcf1/Epha4/Gpr37l1/Hmga2/Il1b/Ldlr/Lif	8
GO:0002755	MyD88-dependent toll-like receptor signaling pathway	6/1242	20/18303	0.001635	0.011665	0.007978	Irak2/Irf1/Tlr2/Tlr5/Tlr6/Tnip1	6
GO:0034138	toll-like receptor 3 signaling pathway	6/1242	20/18303	0.001635	0.011665	0.007978	Flot1/Lgals9/Peli1/Tlr3/Tnf/Tnfaip3	6
GO:0007492	endoderm development	14/1242	85/18303	0.001656	0.011784	0.008059	Bmp4/Col11a1/Col5a1/Col7a1/Dusp5/Gata6/Hmga2/Hnf1b/Inhba/Itga5/Itga7/Lamb3/Mmp9/Pax9	14
GO:0050818	regulation of coagulation	14/1242	85/18303	0.001656	0.011784	0.008059	C1qtnf1/Ephb2/F11r/Hpse/Pdgfb/Plau/Plek/Procr/Selp/Serpinf2/Serping1/Syk/Thbd/Thbs1	14
GO:0002064	epithelial cell development	32/1242	272/18303	0.001682	0.011922	0.008154	Abcb1a/Abcb1b/Akr1b1/Bfsp1/Bmp4/Bmp5/Cldn3/Clic5/Cxcr4/Dact2/Dll1/F11r/Ripor2/Fgfr2/Gpr4/Il1a/Il1b/Map7/Myo7a/Nkx3-2/Pde2a/Pdgfb/Prdm1/Rap1b/Rfx3/Slc4a7/St14/Tmod1/Tnf/Vim/Whrn/Wnt5a	32
GO:0055024	regulation of cardiac muscle tissue development	16/1242	104/18303	0.001683	0.011922	0.008154	Bmp2/Bmp4/Col14a1/Dll1/Fdps/Fgf9/Fgfr2/Gata6/Gli1/Hey2/Kcnk2/Mef2c/Ncam1/Nrg1/Pi16/Rbpj	16
GO:0072080	nephron tubule development	16/1242	104/18303	0.001683	0.011922	0.008154	Agtr1a/Aqp1/Bmp2/Bmp4/Cd44/Dll1/Fat4/Hnf1b/Irx3/Lif/Mef2c/Npnt/Pax8/Pgf/Wnk4/Wnt2b	16
GO:0002573	myeloid leukocyte differentiation	28/1242	228/18303	0.001689	0.011922	0.008154	Bmp4/Ccl3/Ccl5/Ccr1/Csf1/Csf2/Fam20c/Gata2/Gpr68/Ikzf1/Il15/Il23a/Il31ra/Jun/Junb/Kitlg/Lif/Lilrb4/Mafb/Mef2c/Pde1b/Pde2a/Rbp1/Rbpj/Relb/Sfrp1/Tlr2/Tnf	28
GO:0006953	acute-phase response	10/1242	50/18303	0.00169	0.011922	0.008154	Ass1/Ccl5/Ednrb/Hfe/Il1a/Il1b/Il1rn/Lbp/Ptgs2/Tnf	10
GO:0050974	detection of mechanical stimulus involved in sensory perception	10/1242	50/18303	0.00169	0.011922	0.008154	Adgrv1/Col11a1/Cxcl12/Cxcr4/Htr2a/Htr7/Itga2/Piezo2/Tnf/Whrn	10
GO:2001239	regulation of extrinsic apoptotic signaling pathway in absence of ligand	10/1242	50/18303	0.00169	0.011922	0.008154	Col2a1/Csf2/Cx3cl1/Gas1/Hspa1b/Il1b/Inhba/Nrg1/Snai2/Tnf	10
GO:0030522	intracellular receptor signaling pathway	29/1242	239/18303	0.001698	0.011925	0.008156	Alox15/Bdnf/Cyp26b1/Cyp27b1/Cyp2j4/Cyp7b1/Dhrs3/Fabp5/Flt1/Hmga2/Hspa1b/Klf2/Lbh/Nfkbia/Nod2/Nos2/Ppard/Inava/Riok3/Ripk2/Rorb/Sfrp1/Snai2/Tgif1/Thra/Thrb/Tifa/Tnfaip3/Trim68	29
GO:0007379	segment specification	5/1242	14/18303	0.001703	0.011925	0.008156	Cobl/Dll1/Irx3/Mafb/Meox2	5
GO:0035739	CD4-positive, alpha-beta T cell proliferation	5/1242	14/18303	0.001703	0.011925	0.008156	Cd274/Cd44/Lgals9/Vsir/Xcl1	5
GO:0072683	T cell extravasation	5/1242	14/18303	0.001703	0.011925	0.008156	Ccl2/Ccl5/Cd99l2/F11r/Itgal	5
GO:1902931	negative regulation of alcohol biosynthetic process	5/1242	14/18303	0.001703	0.011925	0.008156	Bmp2/Bmp5/Cyp27b1/Nfkb1/Plek	5
GO:2000561	regulation of CD4-positive, alpha-beta T cell proliferation	5/1242	14/18303	0.001703	0.011925	0.008156	Cd274/Cd44/Lgals9/Vsir/Xcl1	5
GO:0051098	regulation of binding	43/1242	398/18303	0.001709	0.011958	0.008178	Atp2a3/Bcl3/Bdnf/Blm/Bmp2/Bmp4/Csf3/Cthrc1/Epha4/Ripor2/Flot1/Foxc1/Gpsm1/Grem2/Hand2/Hey2/Hfe/Hip1r/Hipk2/Hmga2/Itga2/Jun/Larp6/Lgals9/Lif/Mdfi/Mmp9/Ngf/Pcsk9/Pdgfb/Plaur/Plk2/Plxnd1/Ramp2/Rap1gap/Rgma/Ripk2/Spon1/Trim6/Twist2/Wnt5a/Xirp1/Z	43
GO:0098801	regulation of renal system process	9/1242	42/18303	0.001718	0.012003	0.008209	Adora1/Adora2a/Anpep/Coro2b/Ednrb/Gja5/Pdgfb/Ptger4/Ptpro	9
GO:0010575	positive regulation of vascular endothelial growth factor production	7/1242	27/18303	0.001735	0.012087	0.008266	Cyp1b1/Flt4/Hpse/Il1a/Il1b/Nox1/Ptgs2	7
GO:0060713	labyrinthine layer morphogenesis	7/1242	27/18303	0.001735	0.012087	0.008266	Bmp5/Bmp7/Fgfr2/Fzd5/Socs3/St14/Vcam1	7
GO:0016042	lipid catabolic process	37/1242	329/18303	0.001736	0.012087	0.008266	Abcd1/Acat2/Acat2l1/Adora1/Adra2a/Ces1a/Cpt1a/Cpt1b/Cyp1b1/Cyp26b1/Cyp27a1/Cyp27b1/Ech1/Echdc2/Enpp2/Fabp3/Hadh/Hpgd/Plaat1/Il1b/Ldlr/Lipg/Liph/Mgll/Neu3/Hcar2/Phyh/Pla2g2a/Pla2g4e/Pla2g5/Pla2g7/Plcb1/Pnpla3/Ppard/Rarres2/Thra/Tnf	37

ID	Description	GeneRatio	BgRatio	pvalue	p.adjust	qvalue	geneID	Count
GO:0007219	Notch signaling pathway	23/1242	175/18303	0.001746	0.012138	0.008302	Angpt4/Bmp2/Bmp7/Dll1/Dtx4/Fat4/Foxc1/Gata2/Hey2/Hnf1b/Maml3/Nfkbia/Nod2/Nrip2/Ptp4a3/Rbm15/Rbpj/Rcan2/Snai1/Snai2/Sorbs2/Tmem100/Xirp1	23
GO:0007266	Rho protein signal transduction	19/1242	134/18303	0.001762	0.012239	0.008371	Agtr1a/Arap3/Arhgdib/Arhgef25/Arhgef3/Cdc42ep5/Col3a1/F11r/Ripor2/Flcn/Flot1/Gna13/Gpr4/LOC691418/Net1/Nradd/RGD1560455/Rhoj/Srgap1	19
GO:0045926	negative regulation of growth	32/1242	273/18303	0.001784	0.012377	0.008465	Bdkrb1/Bmp4/Ccdc85b/Cth/Cyp27b1/Dusp10/Fhl1/Flcn/Frzb/Gas1/Gdf15/Hspa1b/Hyal1/Igfbp5/Inhba/Kcnk2/Osgin1/Pi16/Plxna3/Ppard/Rerg/Rgma/Sema3f/Sema4a/Sema4b/Sema5a/Sema6a/Sema6d/Sfrp1/Slit2/Slit3/Wnt	32
GO:0019722	calcium-mediated signaling	28/1242	229/18303	0.001803	0.01247	0.008529	Ada/Agtr1a/Ank2/Calml4/Casq1/Ccl20/Ccl3/Ccr1/Cmklr1/Cxcr4/Drd1/Ednrb/Grin2a/Itgal/Ncam1/Nrg1/P2ry6/RGD1564899/Rcan2/Ryr2/Selp/Sgcd/Siglec10/Syk/Tmem100/Tnf/Vcam1/Vsnl1	28
GO:0030509	BMP signaling pathway	22/1242	165/18303	0.001806	0.01247	0.008529	Bmp2/Bmp4/Bmp5/Bmp7/Bmper/Chrd/Egr1/Fam83g/Gata6/Gdf15/Gdf6/Grem2/Hfe/Hipk2/Htra3/Rbpj/Rgma/Scx/Sfrp1/Tmem100/Vsir/Wnt5a	22
GO:0062013	positive regulation of small molecule metabolic process	22/1242	165/18303	0.001806	0.01247	0.008529	Abcd1/Adcyap1r1/Clybl/Cpt1a/Fdps/Has2/Htr2a/Il1b/Nfkb1/Nos2/P2ry6/Pdgfb/Ppard/Ppargc1a/Ppp1r3e/Ppp1r3g/Ptger4/Ptgs2/Pth1r/Star/Stard4/Tnf	22
GO:1990845	adaptive thermogenesis	22/1242	165/18303	0.001806	0.01247	0.008529	Acot11/Acsl1/Arrdc3/Clic5/Cmklr1/Cxcr4/Fabp4/Fabp5/Flcn/Gadd45g/Hadh/Il15/Nova1/Npr3/Ppargc1a/Prkab2/Rbpj/Scd/Syk/Thra/Trpv4/Tshr	22
GO:0060711	labyrinthine layer development	11/1242	59/18303	0.001839	0.012651	0.008652	Bmp5/Bmp7/Fgfr2/Fzd5/Hey2/Junb/Nsdhl/Rbpj/Socs3/St14/Vcam1	11
GO:0070231	T cell apoptotic process	11/1242	59/18303	0.001839	0.012651	0.008652	Ada/Blm/Bmp4/Ccl5/Cd274/Dock8/Efna1/Lgals3/Lgals9/Serpinb9/Wnt5a	11
GO:1904705	regulation of vascular associated smooth muscle cell proliferation	11/1242	59/18303	0.001839	0.012651	0.008652	Ern1/Fgf9/Gstp1/Hpgd/Igfbp5/Jun/Mef2c/Mmp9/P2ry6/Pdgfb/Tnf	11
GO:0032606	type I interferon production	14/1242	86/18303	0.001857	0.012738	0.008711	Csf2/Flot1/Irf1/Acod1/Mmp12/Pycard/Rel/Relb/Riok3/Sirpa/Stat1/Syk/Tlr2/Tlr3	14
GO:0048644	muscle organ morphogenesis	14/1242	86/18303	0.001857	0.012738	0.008711	Ankrd1/Bmp2/Col11a1/Col3a1/Fgfr2/Foxc1/Fzd2/Hey2/Lif/Mylk/Nrg1/Rbpj/Ryr2/Wnt5a	14
GO:0090288	negative regulation of cellular response to growth factor	21/1242	155/18303	0.001858	0.012738	0.008711	Bmper/Cadm4/Cask/Cflar/Chrd/Creb3l1/Dcn/Emilin1/Fuz/Grem2/Hipk2/Hspa1b/Htra3/Peg10/Sema6a/Sfrp1/Slc2a10/Slit2/Spry4/Thbs1/Wnt5a	21
GO:0070588	calcium ion transmembrane transport	36/1242	319/18303	0.001873	0.012822	0.008769	Adra2a/Agtr1a/Ank2/Atp2a3/Bdkrb1/Bmp4/Cacna1g/Cacnb2/Casq1/Ccl3/Cemip/Coro1a/Cx3cl1/Cxcl10/Cxcl11/Drd1/Grin2a/Htr2a/Itgb3/Mcoln2/Mcoln3/Nalcn/Ngf/P2ry6/Pde4b/Rgs9/Ryr2/Ryr3/Slc24a3/Slc24a4/Slc25a25/Slc35g1/Trpc4/Trpv3/Trpv4/Xcl1	36
GO:0045862	positive regulation of proteolysis	40/1242	365/18303	0.001876	0.012825	0.008772	Adra2a/Atp2a3/Casp12/Cflar/Cldn3/Efna1/Egln3/Epha4/F3/Fbln1/Fuz/Gclc/Hip1r/Hspa1b/Il1b/Il33/Lgals9/Meltf/Ngf/Nlrc4/Nlrp3/Plk2/Prickle1/Prr7/Pycard/Rgma/Ripk2/Rnf144a/Rnf19b/Serpinb3/Spon1/Stat1/Syk/Tnf/Tnfrsf1b/Tnfsf15/Tnip1/Trib2/Vsir/Zfand2a	40
GO:0006575	cellular modified amino acid metabolic process	23/1242	176/18303	0.001883	0.012857	0.008793	Ass1/Carns1/Chdh/Ckb/Cpt1a/Cpt1b/Cth/Gamt/Gch1/Gclc/Ggt1/Gsta2/Gsta5/Gstp1/Hoga1/Mthfd2l/Pax8/Plod2/Sardh/Slc22a4/Slc7a11/Slco4a1/Tg	23
GO:0070228	regulation of lymphocyte apoptotic process	13/1242	77/18303	0.001903	0.012937	0.008848	Ada/Blm/Bmp4/Ccl5/Cd274/Cd44/Dock8/Efna1/Fnip1/Lgals3/Lgals9/Serpinb9/Wnt5a	13
GO:0071300	cellular response to retinoic acid	13/1242	77/18303	0.001903	0.012937	0.008848	Aqp1/Ccl2/Col1a1/Col2a1/Cyp26b1/Fgfr2/Hand2/Irf1/Mef2c/Myb/Rorb/Tnf/Twf2	13
GO:0010524	positive regulation of calcium ion transport into cytosol	12/1242	68/18303	0.001903	0.012937	0.008848	Adcyap1r1/Agtr1a/Bdkrb1/Bmp4/Casq1/Cemip/Cx3cl1/Cxcl10/Cxcl11/Drd1/P2ry6/Xcl1	12
GO:0030858	positive regulation of epithelial cell differentiation	12/1242	68/18303	0.001903	0.012937	0.008848	Abcb1a/Bmp4/Cyp27b1/F11r/Foxc1/Foxn1/Macroh2a2/Lif/Pax8/Rfx3/Tmem100/Zeb2	12
GO:0001823	mesonephros development	17/1242	115/18303	0.001911	0.012944	0.008852	Agtr1a/Bdnf/Bmp2/Bmp4/Bmp7/Bmper/Cd44/Fat4/Fgfr2/Foxc1/Hnf1b/Npnt/Pax8/Pgf/Sfrp1/Slit2/Wnt2b	17
GO:0010522	regulation of calcium ion transport into cytosol	17/1242	115/18303	0.001911	0.012944	0.008852	Adcyap1r1/Agtr1a/Ank2/Bdkrb1/Bmp4/Casq1/Cemip/Coro1a/Cx3cl1/Cxcl10/Cxcl11/Drd1/Itgb3/Ngf/P2ry6/Ryr2/Xcl1	17
GO:0048639	positive regulation of developmental growth	27/1242	219/18303	0.001911	0.012944	0.008852	Bdnf/Cdh4/Csf1/Csf2/Cxcl12/Cxcr4/Dll1/Fdps/Fgf9/Fgfr2/Gata6/Gli1/Hey2/Hmga2/Ikzf1/Mef2c/Ncam1/Ngf/Nrg1/Plcb1/Ppard/Ptger4/Rbpj/Sema5a/Sptbn4/Tshr/Tw	27
GO:0060043	regulation of cardiac muscle cell proliferation	10/1242	51/18303	0.001976	0.013323	0.009112	Fgf9/Fgfr2/Gata6/Gli1/Hey2/Kcnk2/Mef2c/Ncam1/Nrg1/Rbpj	10
GO:0001938	positive regulation of endothelial cell proliferation	15/1242	96/18303	0.00198	0.013323	0.009112	Agtr1a/Bmp2/Bmp4/Ccl2/Cxcl12/F3/Flt4/Gata2/Itgb3/Jun/Pdgfb/Pgf/Sema5a/Thbs4/Wnt5a	15
GO:0043535	regulation of blood vessel endothelial cell migration	15/1242	96/18303	0.00198	0.013323	0.009112	Angpt4/Cd40/Efna1/Ets1/Gata2/Hdac9/Mef2c/Meox2/Pdgfb/Plk2/Ptgs2/Rgcc/Rhoj/Thbs1/Tnf	15

ID	Description	GeneRatio	BgRatio	pvalue	p.adjust	qvalue	geneID	Count
GO:0008299	isoprenoid biosynthetic process	8/1242	35/18303	0.001988	0.013323	0.009112	Acat2/Fdft1/Fdps/Hmgcr/Hmgcs1/Idi1/Mvd/Rbp1	8
GO:0032354	response to follicle-stimulating hormone	8/1242	35/18303	0.001988	0.013323	0.009112	Cyp1b1/Egr1/Gclc/Hmgcs1/Inhba/Plat/Ppargc1a/Star	8
GO:0035458	cellular response to interferon-beta	8/1242	35/18303	0.001988	0.013323	0.009112	Gbp4/Hcn1/Ifit3/Irf1/Acod1/Stat1/Tlr3/Trim6	8
GO:0045066	regulatory T cell differentiation	8/1242	35/18303	0.001988	0.013323	0.009112	Cd44/Dusp10/Irf1/Lgals9/Lilrb4/Sox12/Tox/Vsir	8
GO:0060512	prostate gland morphogenesis	8/1242	35/18303	0.001988	0.013323	0.009112	Bmp4/Bmp7/Cd44/Crip1/Cyp7b1/Fgfr2/Sfrp1/Wnt5a	8
GO:1901186	positive regulation of ERBB signaling pathway	8/1242	35/18303	0.001988	0.013323	0.009112	Adora1/Adra2a/Areg/Ereg/Hip1r/Mmp9/Plaur/Rbpj	8
GO:0045471	response to ethanol	31/1242	264/18303	0.002028	0.013563	0.009276	Aacs/Adcyap1r1/Ca3/Casp12/Ccl2/Ccl5/Ccnd1/Cldn3/Cpt1a/Csf3/Drd1/Egr1/Fgfr2/Flt1/Glra1/Grin2a/Gstp1/Hmgcr/Hpgd/Il1b/Ldlr/Mmp9/Nfkb1/Nfkbia/Nlrp3/Nqo1/Penk/Rgs7/Star/Tnf/Vcam1	31
GO:0097696	receptor signaling pathway via STAT	23/1242	177/18303	0.002028	0.013563	0.009276	Agtr1a/Akr1b1/Bcl3/Ccl5/Cd40/Clcf1/Csf2/Cyp1b1/Hmga2/Il15/Il15ra/Il23a/Il31ra/Irf1/Lif/Prl8a9/Ptger4/Ptprt/Socs3/Stat1/Tnf/Tnfsf18/Tslp	23
GO:0036314	response to sterol	9/1242	43/18303	0.002044	0.013652	0.009337	Aacs/Ccl3/Ccl5/Fdps/Hmgcr/Hmgcs1/Inhba/Insig1/Lrp	9
GO:0046427	positive regulation of receptor signaling pathway via JAK-STAT	16/1242	106/18303	0.002059	0.013721	0.009384	Agtr1a/Akr1b1/Ccl5/Cd40/Clcf1/Csf2/Cyp1b1/Il15/Il23a/Il31ra/Lif/Prl8a9/Ptger4/Tnf/Tnfsf18/Tslp	16
GO:0050709	negative regulation of protein secretion	16/1242	106/18303	0.002059	0.013721	0.009384	Adra2a/Cx3cl1/Cyp51/Frmd4a/Hadh/Hdac9/Hmgcr/Il1b/Il33/Ptger4/Rab11fip1/Rgcc/Rsad2/Sfrp1/Siglec10/Vsn	16
GO:0062207	regulation of pattern recognition receptor signaling pathway	14/1242	87/18303	0.002078	0.013831	0.009459	Flot1/Hspa1b/Irf1/Acod1/Lbp/Nod2/Peli1/Riok3/Rsad2/Tlr2/Tlr3/Tlr5/Tlr6/Tnfaip3	14
GO:0003002	regionalization	41/1242	379/18303	0.002109	0.014024	0.009591	Bmp2/Bmp4/Btg2/Chrd/Cobl/Cyp26b1/Dll1/Dmrt2/Fgfr2/Foxc1/Fuz/Fzd5/Gas1/Gbx2/Gli1/Grem2/Hey2/Hipk2/Hnf1b/Hoxb5/Hoxb8/Hoxc6/Irx3/Mafb/Mdfi/Mef2c/Meox2/Nr2f1/Pax1/Pax8/Pcsk5/Plxna2/Rbpj/Sema3f/Sfrp1/Snai1/Tbx3/Tgif1/Wnt2b/Wnt5a/Zeb2	41
GO:0048662	negative regulation of smooth muscle cell proliferation	11/1242	60/18303	0.002117	0.014056	0.009613	Gstp1/Igfbp5/Il15/Mef2c/Ndrg2/Npr3/Ogn/Ppard/Ppargc1a/Ptgir/Tnfaip3	11
GO:0046578	regulation of Ras protein signal transduction	25/1242	199/18303	0.002131	0.014138	0.009669	Arap3/Arhgdib/Arhgef25/Arhgef3/Cadm4/Col3a1/Csf1/Ephb2/F11r/Ripor2/Flcn/Flot1/Gpr4/Kitlg/Net1/Ngf/Nrg1/Psd3/RGD1560455/Ralgps1/Rasa2/Rasal3/Rasgrp1/Sh2b2/Spry4	25
GO:0009743	response to carbohydrate	39/1242	356/18303	0.002135	0.014148	0.009676	Aacs/Adcy8/Adra2a/Calcrl/Ccl2/Col6a3/Egr1/Ercc1/Ern1/Gclc/Gpr68/Grin2a/Gstp1/Hmgcr/Hnf1b/Il1a/Il1b/Il1r1/Itga2/Mafa/Mef2c/Nampt/Nfkb1/Nqo1/Pcsk1/Plau/Plcb1/Pnpla3/Ppard/Ppargc1a/Ppp1r15a/Ptger4/Ptgs2/Rap1b/Star/Sybu/Thbs1/Vcam1/Vsnl1	39
GO:0002043	blood vessel endothelial cell proliferation involved in sprouting angiogenesis	6/1242	21/18303	0.002159	0.014191	0.009706	Agtr1a/Bmp4/Bmper/Gata2/Sema5a/Thbs1	6
GO:0002726	positive regulation of T cell cytokine production	6/1242	21/18303	0.002159	0.014191	0.009706	Fzd5/Il1b/Il1r1/Nlrp3/Rsad2/Xcl1	6
GO:0003177	pulmonary valve development	6/1242	21/18303	0.002159	0.014191	0.009706	Bmp4/Gja5/Hey2/Rbpj/Slit2/Tnfrsf1b	6
GO:0032305	positive regulation of icosanoid secretion	6/1242	21/18303	0.002159	0.014191	0.009706	Il1a/Il1b/P2ry2/Pla2r1/Ptges/Trpv4	6
GO:0032495	response to muramyl dipeptide	6/1242	21/18303	0.002159	0.014191	0.009706	Nfkbia/Nod2/Inava/Ripk2/Tnfaip3/Vim	6
GO:0051797	regulation of hair follicle development	6/1242	21/18303	0.002159	0.014191	0.009706	Cdh3/Foxn1/Hpse/Inhba/Tnf/Wnt5a	6
GO:0060039	pericardium development	6/1242	21/18303	0.002159	0.014191	0.009706	Bmp2/Bmp5/Bmp7/Flrt3/Hand2/Wnt5a	6
GO:0046635	positive regulation of alpha-beta T cell activation	12/1242	69/18303	0.002165	0.01421	0.009718	Ada/Blm/Cd83/Ikzf1/Il23a/Lgals9/Nlrp3/Myb/Rasal3/Ripk2/Syk/Xcl1	12
GO:0002693	positive regulation of cellular extravasation	7/1242	28/18303	0.002179	0.01421	0.009718	Ccl2/Cd99l2/Ripor2/Il1a/Il1r1/Ptger4/Selp	7
GO:0006925	inflammatory cell apoptotic process	7/1242	28/18303	0.002179	0.01421	0.009718	Ccl5/Cd44/Mef2c/Hcar2/Nod2/Pik3cb/Slc7a11	7
GO:0071359	cellular response to dsRNA	7/1242	28/18303	0.002179	0.01421	0.009718	Ciita/Flot1/Grin2a/Irf1/Nfkb1/Riok3/Tlr3	7
GO:0071372	cellular response to follicle-stimulating hormone stimulus	7/1242	28/18303	0.002179	0.01421	0.009718	Egr1/Gclc/Hmgcs1/Inhba/Plat/Ppargc1a/Star	7
GO:0090023	positive regulation of neutrophil	7/1242	28/18303	0.002179	0.01421	0.009718	Dapk2/Ripor2/Il1b/Il23a/Lbp/Thbs4/Xcl1	7

ID	Description	GeneRatio	BgRatio	pvalue	p.adjust	qvalue	geneID	Count
GO:1900120	regulation of receptor binding	7/1242	28/18303	0.002179	0.01421	0.009718	Atp2a3/Bdnf/Grem2/Hfe/Mmp9/Pcsk9/Rgma	7
GO:0043547	positive regulation of GTPase activity	38/1242	345/18303	0.002198	0.014303	0.009782	Adprhl1/Als2/Arap3/Arhgef19/Ccl2/Ccl20/Ccl22/Ccl3/Ccl5/Ccl9/Cd40/Chn1/Cx3cl1/Dock10/Dock4/Dock8/Elmod1/F11r/Flcn/Jun/Net1/Plcb1/Plekhg4/Ralgapa2/Rap1gap/Rasa2/Rasgrp1/Rasgrp3/Rgma/Rgs14/Rgs16/Rgs7/Rgs9/Snx18/Srgap1/Tbc1d9/Wnt5a/Xcl1	38
GO:0006835	dicarboxylic acid transport	15/1242	97/18303	0.002199	0.014303	0.009782	Adora1/Adora2a/Bdnf/Il1b/Il1rn/Lrrc8c/Lrrc8d/Slc17a6/Slc1a1/Slc1a2/Slc26a10/Slc26a4/Slc26a9/Slc7a11/Tnf	15
GO:0002285	lymphocyte activation involved in immune response	24/1242	189/18303	0.002241	0.014566	0.009962	Ada/Bcl3/Cd180/Cd40/Clcf1/Clec4e/Coro1a/Dll1/Dock10/Ercc1/Exosc6/Il23a/Itgal/Lgals3/Lgals9/Nlrp3/Pglyrp1/Pglyrp4/Ptger4/Myb/Relb/Ripk2/Sema4a/Tnfsf18	24
GO:0034765	regulation of ion transmembrane transport	51/1242	499/18303	0.00225	0.014599	0.009985	Abcb1a/Adra2a/Agtr1a/Ank2/Ank3/Bdkrb1/Bmp4/Cacna1g/Cacnb2/Casq1/Ccl2/Cemip/Clic5/Cnih2/Coro1a/Cx3cl1/Cxcl10/Cxcl11/Drd1/Ephb2/Fhl1/Gja5/Grin2a/Hcn1/Itgb3/Kcna3/Kcnh1/Kcnh2/Kcnip4/Lrrc38/Mef2c/Mmp9/Nalcn/Ngf/P2ry6/Pcsk9/Pde4b/Ppargc1a/Ppif/Prrt1/Rgs7/Rgs9/Ryr2/Scn2b/Shank1/Stom/Thbs1/Tnf/Trpv4	51
GO:0060560	developmental growth involved in morphogenesis	32/1242	277/18303	0.002252	0.014599	0.009985	Alcam/Areg/Bdnf/Bmp4/Cadm1/Cdh4/Cobl/Csf1/Cxcl12/Cxcr4/Fgfr2/Flrt3/Hnf1b/Mgll/Ngf/Nrg1/Nrn1/Plxna3/Rgma/Sema3f/Sema4a/Sema4b/Sema5a/Sema6a/Sema6d/Sfrp1/Slit2/Slit3/Tnn/Twf2/Wnt5a/Zeb2	32
GO:0110110	positive regulation of animal organ morphogenesis	16/1242	107/18303	0.002272	0.014715	0.010064	Agtr1a/Bmp2/Bmp4/Bmp7/Cflar/Csf1/Fgfr2/Hand2/Hmga2/Lif/Pax8/Rbpj/Tnf/Tnfaip3/Wnt2b/Wnt5a	16
GO:0032941	secretion by tissue	10/1242	52/18303	0.002301	0.014869	0.010169	Ada/Adora1/Aqp1/Kcnn4/Mmp13/Npr3/P2ry2/Ptger4/Jhy/Wnk4	10
GO:0045687	positive regulation of glial cell	10/1242	52/18303	0.002301	0.014869	0.010169	Aspa/Bmp2/Clcf1/Cxcr4/Enpp2/Il1b/Lif/Tenm4/Tlr2/Tnfrsf1b	10
GO:1903555	regulation of tumor necrosis factor superfamily cytokine production	18/1242	127/18303	0.002308	0.014898	0.010189	Ccl2/Ccl3/Cd274/Gstp1/Il23a/Lbp/Lgals9/Nod2/Ptger4/Pycard/Rasgrp1/Ripk2/Sirpa/Tlr2/Tlr3/Tnfaip3/Twist2/Vsir	18
GO:0051279	regulation of release of sequestered calcium ion into cytosol	14/1242	88/18303	0.00232	0.014926	0.010208	Ank2/Bdkrb1/Casq1/Cemip/Coro1a/Cx3cl1/Cxcl10/Cxcl11/Drd1/Itgb3/Ngf/P2ry6/Ryr2/Xcl1	14
GO:0060420	regulation of heart growth	14/1242	88/18303	0.00232	0.014926	0.010208	Col14a1/Dusp6/Fdps/Fgf9/Fgfr2/Gata6/Gli1/Hey2/Kcnk2/Mef2c/Ncam1/Nrg1/Pi16/Rbpj	14
GO:0072028	nephron morphogenesis	14/1242	88/18303	0.00232	0.014926	0.010208	Agtr1a/Bmp2/Bmp4/Bmp7/Cd44/Fat4/Hnf1b/Irx3/Lif/Npnt/Pax8/Pgf/Wnk4/Wnt2b	14
GO:0002705	positive regulation of leukocyte mediated immunity	23/1242	179/18303	0.002349	0.015094	0.010323	Cadm1/Ccl2/Cd40/Clcf1/Cxcl1/Exosc6/Fgr/Fzd5/Gata2/Hspa1b/Il18rap/Il1b/Il1r1/Il23a/Mr1/Nlrp3/Nod2/Pvr/Rasgrp1/Rsad2/Syk/Tnf/Xcl1	23
GO:0048566	embryonic digestive tract development	8/1242	36/18303	0.002406	0.015331	0.010485	Ada/Fgf9/Fgfr2/Hnf1b/Pcsk5/Myb/Rarres2/Tnf	8
GO:0062149	detection of stimulus involved in sensory perception of pain	8/1242	36/18303	0.002406	0.015331	0.010485	Adora1/Cxcl12/Cxcr4/Htr2a/Htr7/Itga2/Lxn/Tnf	8
GO:0060415	muscle tissue morphogenesis	13/1242	79/18303	0.002411	0.015331	0.010485	Ankrd1/Bmp2/Col11a1/Col3a1/Fgfr2/Foxc1/Fzd2/Hey2/Mylk/Nrg1/Rbpj/Ryr2/Wnt5a	13
GO:0060563	neuroepithelial cell differentiation	13/1242	79/18303	0.002411	0.015331	0.010485	Bmp2/Clic5/Dll1/Dlx3/Fam20c/Ripor2/Hey2/Mcoln3/Mycl/Myo7a/Rbpj/Slc4a7/Whrn	13
GO:0001977	renal system process involved in regulation of blood	5/1242	15/18303	0.002412	0.015331	0.010485	Adora1/Coro2b/Gja5/Pdgfb/Ptpro	5
GO:0002830	positive regulation of type 2 immune response	5/1242	15/18303	0.002412	0.015331	0.010485	Il33/Nlrp3/Nod2/Rsad2/Xcl1	5
GO:0033690	positive regulation of osteoblast	5/1242	15/18303	0.002412	0.015331	0.010485	Bmp2/Cthrc1/Fbln5/Hpse/Itgb3	5
GO:0043508	negative regulation of JUN kinase activity	5/1242	15/18303	0.002412	0.015331	0.010485	Dusp10/Dusp19/Gstp1/Serpinb3/Sfrp1	5
GO:0046642	negative regulation of alpha-beta T cell proliferation	5/1242	15/18303	0.002412	0.015331	0.010485	Cd274/Cd44/Lgals9/Vsir/Xcl1	5
GO:0060442	branching involved in prostate gland morphogenesis	5/1242	15/18303	0.002412	0.015331	0.010485	Bmp4/Bmp7/Cd44/Fgfr2/Sfrp1	5
GO:1903672	positive regulation of sprouting	9/1242	44/18303	0.002418	0.015347	0.010496	Agtr1a/Bmper/Dll1/Gata2/Hdac9/Itga5/Plk2/Ptgs2/Rhoj	9
GO:0035272	exocrine system development	11/1242	61/18303	0.002427	0.015391	0.010526	Bmp7/Edar/Esrp2/Fgfr2/Foxc1/Ntn4/Pdgfb/Plxnd1/Snai2/Tgm2/Tnf	11
GO:0032890	regulation of organic acid transport	15/1242	98/18303	0.002437	0.015435	0.010556	Acsl4/Adora1/Adora2a/Erfe/Fabp3/Il1a/Il1b/Il1rn/P2ry2/Pla2r1/Ptges/Syk/Thbs1/Tnf/Trpv4	15
GO:0006865	amino acid transport	22/1242	169/18303	0.002452	0.015496	0.010598	Adora1/Adora2a/Bdnf/Il1b/Il1rn/Lrrc8c/Lrrc8d/Nfkbie/Slc17a6/Slc1a1/Slc1a2/Slc22a4/Slc25a48/Slc47a1/Slc6a12/Slc6a17/Slc7a11/Slc7a2/Slc7a3/Slc7a8/Tnf/Trpc4	22

ID	Description	GeneRatio	BgRatio	pvalue	p.adjust	qvalue	geneID	Count
GO:0002763	positive regulation of myeloid leukocyte differentiation	12/1242	70/18303	0.002455	0.015496	0.010598	Ccl3/Ccl5/Ccr1/Csf1/Csf2/Gpr68/Ikzf1/Il23a/Jun/Kitlg/Lif/Tnf	12
GO:0006940	regulation of smooth muscle contraction	12/1242	70/18303	0.002455	0.015496	0.010598	Ada/Adora1/Adra2a/Bdkrb1/Calcrl/Dock4/Flt1/Itga2/Npnt/Ptger4/Ptgs2/Trpv4	12
GO:0071772	response to BMP	23/1242	180/18303	0.002525	0.015904	0.010877	Bmp2/Bmp4/Bmp5/Bmp7/Bmper/Chrd/Col2a1/Egr1/Fam83g/Gata6/Gdf15/Gdf6/Grem2/Hfe/Hipk2/Htra3/Rbpj/Rgma/Scx/Sfrp1/Tmem100/Vsir/Wnt5a	23
GO:0071773	cellular response to BMP stimulus	23/1242	180/18303	0.002525	0.015904	0.010877	Bmp2/Bmp4/Bmp5/Bmp7/Bmper/Chrd/Col2a1/Egr1/Fam83g/Gata6/Gdf15/Gdf6/Grem2/Hfe/Hipk2/Htra3/Rbpj/Rgma/Scx/Sfrp1/Tmem100/Vsir/Wnt5a	23
GO:0038061	NIK/NF-kappaB signaling	17/1242	118/18303	0.002528	0.015909	0.01088	Bcl3/Birc3/C1qtnf3/Edar/Il1b/Lgals9/Map3k14/Nfkb2/Nlrp3/Nod2/Ptp4a3/Rel/Relb/Tlr2/Tlr3/Tnf/Tnfsf15	17
GO:1904892	regulation of receptor signaling pathway via STAT	21/1242	159/18303	0.002547	0.016009	0.010949	Agtr1a/Akr1b1/Bcl3/Ccl5/Cd40/Clcf1/Csf2/Cyp1b1/Hmga2/Il15/Il23a/Il31ra/Irf1/Lif/Prl8a9/Ptger4/Ptprt/Socs3/Tnf/Tnfsf18/Tslp	21
GO:0002706	regulation of lymphocyte mediated immunity	24/1242	191/18303	0.002578	0.016189	0.011072	Cadm1/Cd40/Clcf1/Exosc6/Fzd5/Hfe/Hspa1b/Il18rap/Il1b/Il1r1/Il23a/Lgals9/Mr1/Nlrp3/Nod2/Pvr/Rasgrp1/Rsad2/Serpinb3a/Serpinb9/Tnf/Tnfrsf1b/Vsir/Xcl1	24
GO:0046209	nitric oxide metabolic process	14/1242	89/18303	0.002585	0.016198	0.011078	Ass1/Cyp1b1/Ddah2/Il1b/Klf2/LOC497963/Nos2/Ptgs2/Sirpa/Slc7a2/Tlr2/Tlr5/Tlr6/Tnf	14
GO:1904427	positive regulation of calcium ion transmembrane transport	14/1242	89/18303	0.002585	0.016198	0.011078	Agtr1a/Ank2/Bdkrb1/Bmp4/Cacnb2/Casq1/Cemip/Cx3cl1/Cxcl10/Cxcl11/Drd1/P2ry6/Ryr2/Xcl1	14
GO:0001889	liver development	30/1242	257/18303	0.002595	0.01624	0.011107	Aacs/Acat2/Ada/Ass1/Atp2a3/Bmp4/Cadm1/Ccnd1/Cflar/Cobl/Cpt1a/Flt1/Frzb/Fscn1/Gata6/Ggt1/Gli1/Hfe/Hmgcs1/Hnf1b/Itga2/Jun/Lbp/Nfkbia/Pcsk9/Plau/Thra/Tnf/Tnfaip3/Ugt1a6	30
GO:0010810	regulation of cell-substrate adhesion	27/1242	224/18303	0.002641	0.016513	0.011294	Alox15/Cask/Ccdc80/Col1a1/Coro2b/Csf1/Cspg5/Edil3/Emilin1/Enpp2/Fbln1/Fzd4/Has2/Itga5/Itgb3/Limch1/Meltf/Mmp12/Ndnf/Net1/Npnt/Pcsk5/Pik3cb/Plau/Plet1/Smoc1/Thbs1	27
GO:0007618	mating	10/1242	53/18303	0.002667	0.016601	0.011354	Ada/Dnm3/Drd1/Ednrb/Fuom/Klk14/P2ry2/Pmp22/Thra/Thrb	10
GO:0042491	inner ear auditory receptor cell differentiation	10/1242	53/18303	0.002667	0.016601	0.011354	Clic5/Dll1/Ripor2/Hey2/Mcoln3/Mycl/Myo7a/Rbpj/Slc4a7/Whrn	10
GO:0046456	icosanoid biosynthetic process	10/1242	53/18303	0.002667	0.016601	0.011354	Alox15/Bdkrb1/Fabp5/Ggt1/Il1b/Mgst3/Pla2g5/Ptges/Ptgs2/Syk	10
GO:1905953	negative regulation of lipid localization	10/1242	53/18303	0.002667	0.016601	0.011354	Acsl4/Itgb3/Nfkbia/Nrg1/Pcsk9/Pla2r1/Ppard/Thbs1/Tnf/Ttc39b	10
GO:0002698	negative regulation of immune effector process	19/1242	139/18303	0.002699	0.01674	0.011449	Dusp10/Hfe/Il13ra2/Il33/Lgals3/Lgals9/Micb/Nod2/Pglyrp1/Pglyrp4/Riok3/Serpinb3a/Serpinb9/Serping1/Siglec10/Tnf/Tnfsf18/Vsir/Xcl1	19
GO:0010893	positive regulation of steroid biosynthetic process	7/1242	29/18303	0.002707	0.01674	0.011449	Fdps/Il1a/Il1b/Ppargc1a/Star/Stard4/Tnf	7
GO:0021952	central nervous system projection neuron axonogenesis	7/1242	29/18303	0.002707	0.01674	0.011449	Epha4/Ephb2/Ephb3/Prdm8/Slit2/Sptbn4/Zeb2	7
GO:0033081	regulation of T cell differentiation in thymus	7/1242	29/18303	0.002707	0.01674	0.011449	Ada/Bmp4/Foxn1/Il1a/Il1b/Rasgrp1/Tox	7
GO:0060143	positive regulation of syncytium formation by plasma membrane fusion	7/1242	29/18303	0.002707	0.01674	0.011449	Cd53/Cxcl12/Ehd2/Ripor2/Flot1/Flt3lg/Gdf15	7
GO:2000310	regulation of NMDA receptor activity	7/1242	29/18303	0.002707	0.01674	0.011449	Ccl2/Cnih2/Ephb2/Grin2a/Mef2c/Ppargc1a/Rgs9	7
GO:0030178	negative regulation of Wnt signaling pathway	21/1242	160/18303	0.002749	0.016984	0.011616	Ankrd6/Bmp2/Cthrc1/Egr1/Fgf9/Frzb/Fuz/Fzd4/Gli1/Hmga2/Mdfi/Notum/Prickle1/Ptpro/Rnf43/Sfrp1/Snai2/Tmem88/Tnn/Trabd2b/Wnt5a	21
GO:0007389	pattern specification process	49/1242	480/18303	0.002779	0.01715	0.011729	Armc4/Bmp2/Bmp4/Bmp5/Bmp7/Btg2/Chrd/Cobl/Cyp26b1/Dll1/Dmrt2/Dnai1/Dnai2/Fgfr2/Foxc1/Fuz/Fzd5/Gas1/Gbx2/Gli1/Grem2/Hand2/Hey2/Hipk2/Hnf1b/Hoxb5/Hoxb8/Hoxc6/Irx3/Mafb/Mdfi/Mef2c/Meox2/Nkx3-2/Nr2f1/Pax1/Pax8/Pcsk5/Plxna2/Rbpj/Rfx3/Sema3f/Sfrp1/Snai1/Tbx3/Tgif1/Wnt2b/Wnt5a/Zeb2	49
GO:0030903	notochord development	6/1242	22/18303	0.0028	0.017246	0.011795	Cobl/Col13a1/Col2a1/Efna1/Gli1/Wnt5a	6
GO:2000353	positive regulation of endothelial cell apoptotic process	6/1242	22/18303	0.0028	0.017246	0.011795	Bmp4/Ccl2/Cd40/Rgcc/Thbs1/Tnf	6
GO:0099173	postsynapse organization	26/1242	214/18303	0.002811	0.017291	0.011826	Abhd17c/Cadm1/Cask/Ctnnd2/Dnm3/Dock10/Efna1/Epha4/Ephb2/Ephb3/Glrb/Grin2a/Kif1a/Lrfn1/Lrp8/Mpp2/Ndrg2/Nefh/Nptxr/Rapsn/Sema3f/Shank1/Slc7a11/Sorbs2/Wnt5a/Zdhhc15	26

ID	Description	GeneRatio	BgRatio	pvalue	p.adjust	qvalue	geneID	Count
GO:0003254	regulation of membrane depolarization	9/1242	45/18303	0.002843	0.017421	0.011915	Abcd1/Ank3/Cacna1g/Dcn/Fhl1/Gclc/Gja5/Hcn1/Scn2b	9
GO:0043114	regulation of vascular permeability	9/1242	45/18303	0.002843	0.017421	0.011915	Adora2a/Akap12/Flt1/Gpr4/Pde2a/Ptp4a3/Ramp2/Slit2/Trpv4	9
GO:0089718	amino acid import across plasma membrane	9/1242	45/18303	0.002843	0.017421	0.011915	Slc1a1/Slc1a2/Slc22a4/Slc47a1/Slc7a11/Slc7a2/Slc7a3/Slc7a8/Tnf	9
GO:0106106	cold-induced thermogenesis	20/1242	150/18303	0.002847	0.017421	0.011915	Acot11/Acsl1/Arrdc3/Cmklr1/Cxcr4/Fabp4/Fabp5/Flcn/Gadd45g/Hadh/Il15/Nova1/Npr3/Ppargc1a/Prkab2/Rbpj/Scd/Syk/Thra/Tshr	20
GO:0120161	regulation of cold-induced thermogenesis	20/1242	150/18303	0.002847	0.017421	0.011915	Acot11/Acsl1/Arrdc3/Cmklr1/Cxcr4/Fabp4/Fabp5/Flcn/Gadd45g/Hadh/Il15/Nova1/Npr3/Ppargc1a/Prkab2/Rbpj/Scd/Syk/Thra/Tshr	20
GO:0006721	terpenoid metabolic process	14/1242	90/18303	0.002875	0.017554	0.012005	Akr1b1/Cyp1b1/Cyp26b1/Cyp2j4/Dhrs3/Fdft1/Fdps/Hmgcr/Hmgcs1/Ppard/Rarres2/Rbp1/Rdh5/Star	14
GO:0045638	negative regulation of myeloid cell differentiation	14/1242	90/18303	0.002875	0.017554	0.012005	Ccl3/Dll1/Gata2/Gpr68/Hoxb8/LOC102551184/Lilrb4/Lmo2/Mafb/Nfkbia/RGD1562378/Rbm15/Sfrp1/Twist2	14
GO:0008045	motor neuron axon guidance	8/1242	37/18303	0.00289	0.017573	0.012019	Alcam/Chn1/Cxcl12/Cxcr4/Epha4/Plxna3/Sema3f/Slit2	8
GO:0045940	positive regulation of steroid metabolic process	8/1242	37/18303	0.00289	0.017573	0.012019	Agtr1a/Fdps/Il1a/Il1b/Ppargc1a/Star/Stard4/Tnf	8
GO:0060674	placenta blood vessel development	8/1242	37/18303	0.00289	0.017573	0.012019	Fosl1/Fzd5/Hey2/Junb/Nsdhl/Rbm15/Rbpj/Socs3	8
GO:2000273	positive regulation of signaling receptor activity	8/1242	37/18303	0.00289	0.017573	0.012019	Adora1/Adra2a/Areg/Ccl2/Ephb2/Ereg/Hfe/Rgs9	8
GO:0046660	female sex differentiation	23/1242	182/18303	0.00291	0.017674	0.012087	Adcyap1r1/Bmp4/Capn5/Casp12/Col9a3/Csmd1/Ereg/Foxc1/Fzd4/Inha/Inhba/Kitlg/Mmp1/Mmp13/Nefh/Ptger4/Sfrp1/Slit2/Slit3/Sprr2d/Tbx3/Tiparp/Wnt5a	23
GO:0002821	positive regulation of adaptive immune response	19/1242	140/18303	0.00293	0.017776	0.012157	Ada/Cd274/Cd40/Clcf1/Exosc6/Fzd5/Hspa1b/Il1b/Il1r1/Il23a/Mr1/Nlrp3/Nod2/Pvr/Pycard/Ripk2/Rsad2/Tnf/Xcl1	19
GO:0006813	potassium ion transport	29/1242	248/18303	0.002951	0.017889	0.012234	Abcc9/Adora1/Adra2a/Ank2/Ank3/Aqp1/Cxcl1/Fhl1/Gja5/Hcn1/Htr2a/Kcna3/Kcnh1/Kcnh2/Kcnip4/Kcnk2/Kcnk3/Kcnk5/Kcnn4/Kcnq5/Kcnt2/Lrrc38/Nalcn/Rgs7/Slc24a3/Slc24a4/Slc9a9/Trpv4/Wnk4	29
GO:0046545	development of primary female sexual characteristics	21/1242	161/18303	0.002965	0.017931	0.012264	Adcyap1r1/Bmp4/Capn5/Casp12/Col9a3/Csmd1/Ereg/Foxc1/Fzd4/Inha/Inhba/Kitlg/Mmp1/Mmp13/Nefh/Ptger4/Sfrp1/Slit2/Slit3/Sprr2d/Tiparp	21
GO:0048565	digestive tract development	21/1242	161/18303	0.002965	0.017931	0.012264	Ada/Ass1/Bmp4/Cobl/Col3a1/Ednrb/Ephb3/Fat4/Fgf9/Fgfr2/Gata6/Hnf1b/Nkx3-2/Pcsk5/Prdm1/Myb/Rarres2/Sfrp1/Thra/Tnf/Wnt5a	21
GO:0007200	phospholipase C-activating G protein-coupled receptor signaling pathway	15/1242	100/18303	0.002978	0.017971	0.012291	Adra2a/Agtr1a/Cmklr1/Drd1/Ednrb/Gna14/Gpr4/Hcrtr2/Htr2a/Npr3/P2ry2/Pth1r/RGD1560455/Rxfp3/Tgm2	15
GO:0051348	negative regulation of transferase activity	32/1242	282/18303	0.002981	0.017971	0.012291	Adora2a/Bmp4/Bmp7/Drd1/Dusp10/Dusp16/Dusp19/Dusp5/Dusp6/Ephb2/Errfi1/Fabp4/Gadd45g/Gstp1/Hmgcr/Il1b/Itprip/Pcp4/Pik3ip1/Prkar2a/Prkar2b/Ptpn5/Ptprb/Ptpro/Ptprt/Pycard/Rgs14/Serpinb3/Sfrp1/Smcr8/Spry4/	32
GO:0060070	canonical Wnt signaling pathway	32/1242	282/18303	0.002981	0.017971	0.012291	Ankrd6/Bmp2/Cdh3/Col1a1/Cthrc1/Ctnnd2/Egr1/Fgf9/Fgfr2/Frzb/Fuz/Fzd2/Fzd4/Fzd5/Fzd8/Gli1/Lgr5/Mgat3/Nfkb1/Notum/Plpp3/Prickle1/Ptpro/Rbpj/Rspo1/Sema5a/Sfrp1/Snai2/Tmem88/Tnn/Wnt2b/Wnt5a	32
GO:1904894	positive regulation of receptor signaling pathway via STAT	16/1242	110/18303	0.003024	0.018167	0.012425	Agtr1a/Akr1b1/Ccl5/Cd40/Clcf1/Csf2/Cyp1b1/Il15/Il23a/Il31ra/Lif/Prl8a9/Ptger4/Tnf/Tnfsf18/Tslp	16
GO:0030193	regulation of blood coagulation	13/1242	81/18303	0.003026	0.018167	0.012425	C1qtnf1/Ephb2/F11r/Hpse/Pdgfb/Plau/Plek/Selp/Serpinf2/Serping1/Syk/Thbd/Thbs1	13
GO:0055021	regulation of cardiac muscle tissue growth	13/1242	81/18303	0.003026	0.018167	0.012425	Col14a1/Fdps/Fgf9/Fgfr2/Gata6/Gli1/Hey2/Kcnk2/Mef2c/Ncam1/Nrg1/Pi16/Rbpj	13
GO:0042698	ovulation cycle	17/1242	120/18303	0.003026	0.018167	0.012425	Bmp2/Capn5/Casp12/Cyp1b1/Egr1/Enpp2/Ereg/Ets1/Fzd4/Has2/Inhba/Mmp1/Mmp13/Ptger4/Slit2/Slit3/Wnt5a	17
GO:0007259	receptor signaling pathway via JAK-STAT	22/1242	172/18303	0.003055	0.018323	0.012531	Agtr1a/Akr1b1/Bcl3/Ccl5/Cd40/Clcf1/Csf2/Cyp1b1/Hmga2/Il15/Il15ra/Il23a/Il31ra/Irf1/Lif/Prl8a9/Ptger4/Socs3/Stat1/Tnf/Tnfsf18/Tslp	22
GO:1903707	negative regulation of hemopoiesis	20/1242	151/18303	0.003078	0.018423	0.0126	Bmp4/Ccl3/Cd44/Dll1/Gata2/Gpr68/Hoxb8/Irf1/LOC102551184/Lilrb4/Lmo2/Mafb/Nfkbia/Pglyrp1/Pglyrp4/RGD1562378/Rbm15/Sfrp1/Tnfsf18/Twist2	20
GO:0043268	positive regulation of potassium ion transport	10/1242	54/18303	0.003078	0.018423	0.0126	Adora1/Adra2a/Ank2/Cxcl1/Fhl1/Kcnh2/Lrrc38/Rgs7/Trpv4/Wnk4	10
GO:0061008	hepaticobiliary system development	30/1242	260/18303	0.003088	0.018463	0.012627	Aacs/Acat2/Ada/Ass1/Atp2a3/Bmp4/Cadm1/Ccnd1/Cflar/Cobl/Cpt1a/Flt1/Frzb/Fscn1/Gata6/Ggt1/Gli1/Hfe/Hmgcs1/Hnf1b/Itga2/Jun/Lbp/Nfkbia/Pcsk9/Plau/Thra/Tnf/Tnfaip3/Ugt1a6	30

ID	Description	GeneRatio	BgRatio	pvalue	p.adjust	qvalue	geneID	Count
GO:0048592	eye morphogenesis	23/1242	183/18303	0.00312	0.018637	0.012747	Aqp1/Bdnf/Bmp4/Bmp7/Cfh/Col5a1/Col8a2/Dll1/Ephb2/Fat3/Flt1/Fzd5/Gas1/Hcn1/Hipk2/Ikzf1/Nectin1/Obsl1/Pdgfb/Prdm1/Rorb/Slc4a7/Thrb	23
GO:0006801	superoxide metabolic process	12/1242	72/18303	0.003129	0.018652	0.012757	Agtr1a/Cxcl1/Fbln5/Gch1/Gstp1/Hvcn1/Nos2/Nox1/Nqo1/Olr1/Syk/Tnf	12
GO:0032835	glomerulus development	12/1242	72/18303	0.003129	0.018652	0.012757	Aqp1/Bmp4/Bmp7/Cfh/Cflar/Egr1/Foxc1/Gpr4/Itgb3/Mef2c/Pdgfb/Ptpro	12
GO:0031622	positive regulation of fever generation	4/1242	10/18303	0.003181	0.018844	0.012887	Ccl5/Il1b/Ptgs2/Tnf	4
GO:0035425	autocrine signaling	4/1242	10/18303	0.003181	0.018844	0.012887	Cd68/Cx3cl1/Hilpda/Serpinb3	4
GO:0035744	T-helper 1 cell cytokine production	4/1242	10/18303	0.003181	0.018844	0.012887	Il18rap/Il1b/Il1r1/Xcl1	4
GO:0070244	negative regulation of thymocyte apoptotic process	4/1242	10/18303	0.003181	0.018844	0.012887	Ada/Blm/Bmp4/Efna1	4
GO:1901724	positive regulation of cell proliferation involved in kidney development	4/1242	10/18303	0.003181	0.018844	0.012887	Cflar/Egr1/Itgb3/Pdgfb	4
GO:1990264	peptidyl-tyrosine dephosphorylation involved in inactivation of protein kinase	4/1242	10/18303	0.003181	0.018844	0.012887	Dusp10/Ptprb/Ptpro/Ptprt	4
GO:0048015	phosphatidylinositol-mediated signaling	22/1242	173/18303	0.003282	0.019254	0.013168	Ca8/Ccl5/Csf2/Csf3/Dcn/Fgr/Flt1/Htr2a/Kcnh1/Npr3/Nrg1/Pdgfb/Pik3c2b/Pik3cb/Pik3ip1/Pik3r5/Plcb1/Ppard/Rasgrp1/Ror1/Selp/Tnf	22
GO:0018958	phenol-containing compound metabolic process	17/1242	121/18303	0.003303	0.019254	0.013168	Agtr1a/Akr1b1/Cdh3/Drd1/Fah/Gch1/Gpr37/Grin2a/Hand2/Pax8/Pde1b/Slc7a11/Slco4a1/Sncaip/Star/Tg/Wnt5a	17
GO:0060419	heart growth	17/1242	121/18303	0.003303	0.019254	0.013168	Col14a1/Dusp6/Fdps/Fgf9/Fgfr2/Foxc1/Gata6/Gli1/Hey2/Kcnk2/Mef2c/Ncam1/Nrg1/Pi16/Rbpj/Sorbs2/Tenm4	17
GO:0032308	positive regulation of prostaglandin secretion	5/1242	16/18303	0.003315	0.019254	0.013168	Il1a/Il1b/P2ry2/Ptges/Trpv4	5
GO:0033623	regulation of integrin activation	5/1242	16/18303	0.003315	0.019254	0.013168	Pcsk5/Plek/Ptger4/Rap1b/Selp	5
GO:0034134	toll-like receptor 2 signaling pathway	5/1242	16/18303	0.003315	0.019254	0.013168	Acod1/Nod2/Ripk2/Tlr2/Tlr6	5
GO:0035810	positive regulation of urine volume	5/1242	16/18303	0.003315	0.019254	0.013168	Adora2a/Ednrb/Has2/Npr3/Ptger4	5
GO:0038065	collagen-activated signaling pathway	5/1242	16/18303	0.003315	0.019254	0.013168	Col1a1/Col4a1/Itga11/Itga2/Syk	5
GO:0043173	nucleotide salvage	5/1242	16/18303	0.003315	0.019254	0.013168	Ada/Ampd3/Naprt/Uck2/Upp1	5
GO:0043217	myelin maintenance	5/1242	16/18303	0.003315	0.019254	0.013168	Abcd1/Cxcr4/Fa2h/Myrf/Sh3tc2	5
GO:0051044	positive regulation of membrane protein ectodomain	5/1242	16/18303	0.003315	0.019254	0.013168	Adra2a/Il1b/Rgma/Tnf/Tnfrsf1b	5
GO:0072498	embryonic skeletal joint development	5/1242	16/18303	0.003315	0.019254	0.013168	Bmp4/Bmp7/Col2a1/Hyal1/Slc2a10	5
GO:1902043	positive regulation of extrinsic apoptotic signaling pathway via death domain receptors	5/1242	16/18303	0.003315	0.019254	0.013168	Atf3/Mal/Sfrp1/Thbs1/Timp3	5
GO:1902337	regulation of apoptotic process involved in	5/1242	16/18303	0.003315	0.019254	0.013168	Bmp7/Foxc1/Hnf1b/Pax8/Tnfrsf1b	5
GO:0055017	cardiac muscle tissue growth	16/1242	111/18303	0.003317	0.019254	0.013168	Col14a1/Fdps/Fgf9/Fgfr2/Foxc1/Gata6/Gli1/Hey2/Kcnk2/Mef2c/Ncam1/Nrg1/Pi16/Rbpj/Sorbs2/Tenm4	16
GO:0098657	import into cell	29/1242	250/18303	0.003318	0.019254	0.013168	Abcc9/Acsl1/Acsl3/Agtr1a/Dnm3/Fabp3/Flot1/Hfe/Itgb3/Kcnh2/Kcnk5/Nat8l/Nrg1/Slc17a6/Slc1a1/Slc1a2/Slc22a4/Slc2a10/Slc47a1/Slc7a11/Slc7a2/Slc7a3/Slc7a8/Slc9a9/Thbs1/Tnf/Trpv3/Trpv4/Wnk4	29
GO:0001774	microglial cell activation	9/1242	46/18303	0.003326	0.019254	0.013168	Cx3cl1/Il33/Jun/Ldlr/Nampt/Tlr2/Tlr3/Tlr6/Tnf	9
GO:0002269	leukocyte activation involved in inflammatory	9/1242	46/18303	0.003326	0.019254	0.013168	Cx3cl1/Il33/Jun/Ldlr/Nampt/Tlr2/Tlr3/Tlr6/Tnf	9
GO:0036230	granulocyte	9/1242	46/18303	0.003326	0.019254	0.013168	Anxa3/Ccl3/Ccl5/Il15/Il18rap/Myo1f/Pla2g2a/Syk/Tnf	9
GO:0090184	positive regulation of kidney development	9/1242	46/18303	0.003326	0.019254	0.013168	Agtr1a/Bmp4/Cflar/Egr1/Itgb3/Lif/Pax8/Pdgfb/Wnt2b	9
GO:1902742	apoptotic process involved in development	9/1242	46/18303	0.003326	0.019254	0.013168	Bmp7/Foxc1/Fzd5/Hand2/Hnf1b/Pax8/Slit2/Slit3/Tnfrsf1b	9
GO:0045652	regulation of megakaryocyte differentiation	7/1242	30/18303	0.003327	0.019254	0.013168	Faxdc2/Gata2/LOC102551184/Mef2c/RGD1562378/Rbm15/Scin	7

ID	Description	GeneRatio	BgRatio	pvalue	p.adjust	qvalue	geneID	Count
GO:0071624	positive regulation of granulocyte chemotaxis	7/1242	30/18303	0.003327	0.019254	0.013168	Dapk2/Ripor2/Il1b/Il23a/Lbp/Thbs4/Xcl1	7
GO:0007612	learning	23/1242	184/18303	0.003343	0.019329	0.013219	Bdnf/Btg2/Csmd1/Ctnnd2/Drd1/Elavl4/Ephb2/Fosl1/Gpr88/Grin2a/Hmgcr/Jun/Lrrn4/Nrxn3/Pde1b/Plcb1/Prkar2b/Ptgs2/Rgs14/Sgk1/Shank1/Slc7a11/Tlr2	23
GO:0046486	glycerolipid metabolic process	40/1242	377/18303	0.003348	0.019334	0.013223	Acsl1/Acsl3/Acsl4/Alox15/Chka/Cpt1a/Dgkg/Efr3b/Enpp2/Fabp3/Fabp5/Plaat1/Htr2a/Insig1/Ldlr/Lipg/Liph/Mgll/Mogat2/Mtmr10/Mtmr11/Mtmr7/Pcsk9/Pdgfb/Pik3c2b/Pik3cb/Pip5k1b/Pla2g2a/Pla2g4e/Pla2g5/Pla2g7/Plcb1/Plek/Pnpla3/Rab38/Scd/Slc22a4/Socs3/Thrb/Pip4p1	40
GO:0002532	production of molecular mediator involved in inflammatory	13/1242	82/18303	0.003378	0.019449	0.013302	Abcd1/Chid1/Dusp10/Gbp5/Lbp/Nod2/Nos2/Pycard/Sirpa/Slc7a2/Syk/Tlr6/Tnf	13
GO:0032479	regulation of type I interferon production	13/1242	82/18303	0.003378	0.019449	0.013302	Flot1/Irf1/Acod1/Mmp12/Pycard/Rel/Relb/Riok3/Sirpa/Stat1/Syk/Tlr2/Tlr3	13
GO:1900046	regulation of hemostasis	13/1242	82/18303	0.003378	0.019449	0.013302	C1qtnf1/Ephb2/F11r/Hpse/Pdgfb/Plau/Plek/Selp/Serpinf2/Serping1/Syk/Thbd/Thbs1	13
GO:0031345	negative regulation of cell projection organization	26/1242	217/18303	0.0034	0.019561	0.013378	Cspg4/Dnm3/Efna1/Epha4/Ephb2/Fat3/Il15ra/Nr2f1/Plk2/Plxna3/Pmp22/Rap1gap/Rbpj/Rgma/Rtn4rl2/Sema3f/Sema4a/Sema4b/Sema5a/Sema6a/Sema6d/Slit2/Trpv4/Vim/Wnt5a/Xylt1	26
GO:0032689	negative regulation of interferon-gamma production	8/1242	38/18303	0.003447	0.01977	0.013521	Cd274/Il33/Lgals9/Nod2/Pglyrp1/Pglyrp4/Vsir/Xcl1	8
GO:0032885	regulation of polysaccharide biosynthetic process	8/1242	38/18303	0.003447	0.01977	0.013521	Has2/Nfkb1/Pdgfb/Ppp1r3b/Ppp1r3c/Ppp1r3e/Ppp1r3g/Ptger4	8
GO:0070723	response to cholesterol	8/1242	38/18303	0.003447	0.01977	0.013521	Aacs/Ccl3/Ccl5/Fdps/Hmgcr/Hmgcs1/Inhba/Lrp8	8
GO:1903522	regulation of blood circulation	30/1242	262/18303	0.003459	0.019819	0.013555	Abcc9/Ada/Adora1/Adra2a/Agtr1a/Ank2/Bves/Cacna1g/Cacnb2/Cx3cl1/Dock4/Drd1/Dusp5/Gch1/Gja5/Hey2/Hspa1b/Htr2a/Kcnh2/Nos2/Npy1r/Pde4b/Ptgs2/Ryr2/Scn2b/Slc1a1/Sptbn4/Thra/Thrb/Tnf	30
GO:0045055	regulated exocytosis	32/1242	285/18303	0.003508	0.020062	0.01372	Adora2a/Adra2a/Anxa3/Cacna1g/Cacnb2/Cask/Ccl3/Coro1a/Cplx4/Cspg5/Doc2a/Drd1/Erc2/Fgr/Gata2/Htr2a/Htr7/Il13ra2/Kcnh1/Lgals9/Mical1/Myo1f/Nrn1/Nrxn3/P2ry2/Pclo/Plek/Rab11fip1/Rab3d/Rap1b/Rasgrp1/Syk	32
GO:0015800	acidic amino acid transport	12/1242	73/18303	0.003519	0.020062	0.01372	Adora1/Adora2a/Bdnf/Il1b/Il1rn/Lrrc8c/Lrrc8d/Slc17a6/Slc1a1/Slc1a2/Slc7a11/Tnf	12
GO:0045600	positive regulation of fat cell differentiation	12/1242	73/18303	0.003519	0.020062	0.01372	Bmp2/Bmp7/Cmklr1/Frzb/Htr2a/Medag/Noct/Ptgs2/Rarres2/Sfrp1/Snai2/Zbtb7c	12
GO:0071398	cellular response to fatty acid	12/1242	73/18303	0.003519	0.020062	0.01372	Acat2/Ass1/Ccl2/Cpt1a/Fdps/Il1b/Ldlr/P2ry6/Plcb1/Ppargc1a/Ptger4/Sfrp1	12
GO:0071549	cellular response to dexamethasone stimulus	12/1242	73/18303	0.003519	0.020062	0.01372	Abcb1a/Agtr1a/Aqp1/Ass1/Bmp4/Cacna1g/Ccl2/Cflar/Errfi1/Gjb2/Plat/Star	12
GO:0006690	icosanoid metabolic process	18/1242	132/18303	0.003535	0.020131	0.013768	Alox15/Bdkrb1/Cyp1b1/Cyp2j4/Fabp5/Ggt1/Gsta2/Gstp1/Hpgd/Il1b/Mgll/Mgst3/Pla2g5/Ptges/Ptgr1/Ptgs2/Syk	18
GO:0043620	regulation of DNA-templated transcription in	10/1242	55/18303	0.003538	0.020131	0.013768	Atf3/Bach1/Creb3l1/Egr1/Hspa1b/Jun/Klf2/Ppp1r15a/Rbpj/Rgs14	10
GO:0050966	detection of mechanical stimulus involved in sensory perception of pain	6/1242	23/18303	0.003574	0.02026	0.013856	Cxcl12/Cxcr4/Htr2a/Htr7/Itga2/Tnf	6
GO:0051043	regulation of membrane protein ectodomain	6/1242	23/18303	0.003574	0.02026	0.013856	Adra2a/Il1b/Rgma/Timp3/Tnf/Tnfrsf1b	6
GO:0060252	positive regulation of glial cell proliferation	6/1242	23/18303	0.003574	0.02026	0.013856	Flt1/Il1b/Nrg1/Myb/Tnf/Vim	6
GO:0000768	syncytium formation by plasma membrane fusion	11/1242	64/18303	0.003585	0.02026	0.013856	Cd44/Cd53/Cflar/Cxcl10/Cxcl12/Ehd2/Ripor2/Flot1/Flt3lg/Gdf15/Stat1	11
GO:0015695	organic cation transport	11/1242	64/18303	0.003585	0.02026	0.013856	Abcb1a/Adora2a/Htr7/Itgb3/Lgals3/Slc22a4/Slc25a48/Slc44a2/Slc47a1/Slc7a8/Syk	11
GO:0032760	positive regulation of tumor necrosis factor production	11/1242	64/18303	0.003585	0.02026	0.013856	Ccl2/Ccl3/Il23a/Lbp/Lgals9/Nod2/Pycard/Rasgrp1/Ripk2/Tlr2/Tlr3	11
GO:0140253	cell-cell fusion	11/1242	64/18303	0.003585	0.02026	0.013856	Cd44/Cd53/Cflar/Cxcl10/Cxcl12/Ehd2/Ripor2/Flot1/Flt3lg/Gdf15/Stat1	11
GO:0042303	molting cycle	17/1242	122/18303	0.003602	0.020295	0.01388	Cdh3/Edar/Fa2h/Fgfr2/Foxn1/Fuz/Hpse/Igfbp5/Inhba/Lgr5/Nsdhl/Ptgs2/Rbpj/Snai1/Tnf/Trpv3/Wnt5a	17
GO:0042633	hair cycle	17/1242	122/18303	0.003602	0.020295	0.01388	Cdh3/Edar/Fa2h/Fgfr2/Foxn1/Fuz/Hpse/Igfbp5/Inhba/Lgr5/Nsdhl/Ptgs2/Rbpj/Snai1/Tnf/Trpv3/Wnt5a	17
GO:1904019	epithelial cell apoptotic process	17/1242	122/18303	0.003602	0.020295	0.01388	Angptl4/Bmp4/Ccl2/Cd40/Cflar/Gata2/Ndnf/Ngf/Pla2r1/Ppargc1a/Ramp2/Rgcc/Ryr2/Sema5a/Thbs1/Tnf/Tnfaip	17
GO:0042475	odontogenesis of dentin-containing	15/1242	102/18303	0.003613	0.02034	0.013911	Bmp2/Bmp4/Bmp7/Csf1/Dlx3/Edar/Fam20c/Foxc1/Gata6/Hand2/Klk4/Nectin1/Relt/Slc24a4/Tnfrsf11b	15

ID	Description	GeneRatio	BgRatio	pvalue	p.adjust	qvalue	geneID	Count
GO:0032729	positive regulation of interferon-gamma production	13/1242	83/18303	0.003762	0.021155	0.014468	Bcl3/Fzd5/Il1b/Il1r1/Il23a/Lgals9/Pde4b/Pycard/Rasgrp1/Ripk2/Tlr3/Tnf/Wnt5a	13
GO:0071706	tumor necrosis factor superfamily cytokine production	18/1242	133/18303	0.003836	0.021549	0.014738	Ccl2/Ccl3/Cd274/Gstp1/Il23a/Lbp/Lgals9/Nod2/Ptger4/Pycard/Rasgrp1/Ripk2/Sirpa/Tlr2/Tlr3/Tnfaip3/Twist2/Vsir	18
GO:0050866	negative regulation of cell activation	26/1242	219/18303	0.003849	0.021602	0.014774	Adora2a/Bmp4/C1qtnf1/Cd274/Cd44/Cx3cl1/Emilin1/Ripor2/Fgr/Gclc/Hfe/Il13ra2/Irf1/Ldlr/Lgals3/Lgals9/Pdgfb/Peli1/Pglyrp1/Pglyrp4/Prdm1/Sfrp1/Tnfaip3/Tnfsf1	26
GO:0046425	regulation of receptor signaling pathway via JAK-STAT	20/1242	154/18303	0.003866	0.021621	0.014787	Agtr1a/Akr1b1/Bcl3/Ccl5/Cd40/Clcf1/Csf2/Cyp1b1/Hmga2/Il15/Il23a/Il31ra/Irf1/Lif/Prl8a9/Ptger4/Socs3/Tnf/Tnfsf18/Tslp	20
GO:0003197	endocardial cushion development	9/1242	47/18303	0.003871	0.021621	0.014787	Bmp2/Bmp4/Bmp5/Bmp7/Rbpj/Snai1/Snai2/Thbs1/Tmem100	9
GO:0035850	epithelial cell differentiation involved in kidney	9/1242	47/18303	0.003871	0.021621	0.014787	Bmp4/Fat4/Gpr4/Lif/Mef2c/Mmp9/Pax8/Pdgfb/Ptpro	9
GO:0043616	keratinocyte proliferation	9/1242	47/18303	0.003871	0.021621	0.014787	Cask/Cdh3/Eppk1/Ereg/Fgfr2/Has2/Ppard/Snai2/Twist2	9
GO:0051281	positive regulation of release of sequestered calcium ion into cytosol	9/1242	47/18303	0.003871	0.021621	0.014787	Bdkrb1/Casq1/Cemip/Cx3cl1/Cxcl10/Cxcl11/Drd1/P2ry6/Xcl1	9
GO:0045927	positive regulation of growth	34/1242	310/18303	0.003892	0.021715	0.014851	Acsl4/Bdnf/Cdh4/Csf1/Csf2/Cxcl12/Cxcl16/Cxcr4/Dll1/Extl3/Fdps/Fgf9/Fgfr2/Flt4/Gata6/Gli1/Hey2/Hmga2/Hyal1/Ikzf1/Mef2c/Ncam1/Ngf/Nrg1/Plcb1/Ppard/Ptger4/Rbpj/Sema5a/Sfrp1/Sgk1/Sptbn4/Tshr/Twf2	34
GO:0002709	regulation of T cell mediated immunity	14/1242	93/18303	0.003907	0.021777	0.014894	Fzd5/Hfe/Hspa1b/Il1b/Il1r1/Il23a/Mr1/Nlrp3/Nod2/Pvr/Rsad2/Tnfrsf1b/Vsir/Xcl1	14
GO:0060997	dendritic spine morphogenesis	12/1242	74/18303	0.003947	0.021979	0.015032	Cask/Ctnnd2/Dnm3/Dock10/Efna1/Epha4/Ephb2/Ephb3/Kif1a/Lrp8/Shank1/Zdhhc15	12
GO:0048017	inositol lipid-mediated signaling	22/1242	176/18303	0.004049	0.022334	0.015275	Ca8/Ccl5/Csf2/Csf3/Dcn/Fgr/Flt1/Htr2a/Kcnh1/Npr3/Nrg1/Pdgfb/Pik3c2b/Pik3cb/Pik3ip1/Pik3r5/Plcb1/Ppard/Rasgrp1/Ror1/Selp/Tnf	22
GO:0055123	digestive system development	22/1242	176/18303	0.004049	0.022334	0.015275	Ada/Ass1/Bmp4/Cobl/Col3a1/Ednrb/Ephb3/Fat4/Fgf9/Fgfr2/Gata6/Hip1r/Hnf1b/Nkx3-2/Pcsk5/Prdm1/Myb/Rarres2/Sfrp1/Thra/Tnf/Wnt5a	22
GO:0002523	leukocyte migration involved in inflammatory	7/1242	31/18303	0.00405	0.022334	0.015275	Ccl2/Cx3cl1/Lbp/Nfkb1/Nfkbia/Nlrp3/Tnf	7
GO:0045589	regulation of regulatory T cell differentiation	7/1242	31/18303	0.00405	0.022334	0.015275	Cd44/Dusp10/Irf1/Lgals9/Lilrb4/Sox12/Vsir	7
GO:0045672	positive regulation of osteoclast differentiation	7/1242	31/18303	0.00405	0.022334	0.015275	Ccl3/Ccl5/Ccr1/Csf1/Gpr68/Il23a/Tnf	7
GO:0060669	embryonic placenta morphogenesis	7/1242	31/18303	0.00405	0.022334	0.015275	Bmp5/Bmp7/Fgfr2/Fzd5/Socs3/St14/Vcam1	7
GO:0060706	cell differentiation involved in embryonic placenta	7/1242	31/18303	0.00405	0.022334	0.015275	Fzd5/Lif/Mdfi/Prdm1/Snai1/Socs3/St14	7
GO:0035567	non-canonical Wnt signaling pathway	10/1242	56/18303	0.004051	0.022334	0.015275	Ankrd6/Cthrc1/Frzb/Fzd2/Fzd4/Fzd5/Fzd8/Rspo1/Sfrp1/Wnt5a	10
GO:0043030	regulation of macrophage	10/1242	56/18303	0.004051	0.022334	0.015275	Cx3cl1/Il33/Lbp/Ldlr/Lgals9/Pla2g5/Slc7a2/Thbs1/Tlr6/Wnt5a	10
GO:0048286	lung alveolus development	11/1242	65/18303	0.004057	0.022334	0.015275	Ada/Bmp4/Errfi1/Fgfr2/Flt4/Gata6/Igfbp5/Lif/Slc7a11/Slfn4/Wnt5a	11
GO:0060350	endochondral bone morphogenesis	11/1242	65/18303	0.004057	0.022334	0.015275	Bmp4/Col13a1/Col1a1/Col2a1/Foxc1/Mef2c/Mmp13/Mmp16/Scx/Thbs1/Trpv4	11
GO:1903557	positive regulation of tumor necrosis factor superfamily cytokine production	11/1242	65/18303	0.004057	0.022334	0.015275	Ccl2/Ccl3/Il23a/Lbp/Lgals9/Nod2/Pycard/Rasgrp1/Ripk2/Tlr2/Tlr3	11
GO:0042119	neutrophil activation	8/1242	39/18303	0.004083	0.022416	0.015331	Anxa3/Ccl5/Il15/Il18rap/Myo1f/Pla2g2a/Syk/Tnf	8
GO:0062208	positive regulation of pattern recognition receptor signaling pathway	8/1242	39/18303	0.004083	0.022416	0.015331	Flot1/Hspa1b/Lbp/Peli1/Rsad2/Tlr2/Tlr3/Tlr5	8
GO:0120178	steroid hormone biosynthetic process	8/1242	39/18303	0.004083	0.022416	0.015331	Bmp2/Bmp5/Cyp11b2/Cyp27a1/Egr1/Hsd17b7/Ppargc1a/Star	8
GO:1903409	reactive oxygen species biosynthetic process	18/1242	134/18303	0.004157	0.022801	0.015594	Abcd1/Ass1/Cflar/Cyp1b1/Ddah2/Il1b/Klf2/LOC497963/Nos2/Ptgs2/Inava/Sirpa/Slc5a3/Slc7a2/Tlr2/Tlr5/Tlr6/Tnf	18
GO:0008585	female gonad development	20/1242	155/18303	0.004164	0.022814	0.015603	Bmp4/Capn5/Casp12/Col9a3/Csmd1/Ereg/Foxc1/Fzd4/Inha/Inhba/Kitlg/Mmp1/Mmp13/Nefh/Ptger4/Sfrp1/Slit2/Slit3/Sprr2d/Tiparp	20
GO:0051899	membrane depolarization	13/1242	84/18303	0.004181	0.022887	0.015653	Abcd1/Adora2a/Ank3/Cacna1g/Cacnb2/Dcn/Fhl1/Gclc/Gja5/Hcn1/Jun/Kcnh2/Scn2b	13

ID	Description	GeneRatio	BgRatio	pvalue	p.adjust	qvalue	geneID	Count
GO:0010469	regulation of signaling receptor activity	21/1242	166/18303	0.004267	0.023335	0.01596	Adora1/Adra2a/Areg/Ccl2/Cnih2/Dlgap2/Ephb2/Ereg/Errfi1/Grem2/Grin2a/Hfe/Lynx1/Mef2c/Nptxr/Pcsk9/Plau/Ppargc1a/Prrt1/Rgs9/Shank1	21
GO:2001057	reactive nitrogen species metabolic process	14/1242	94/18303	0.004311	0.023552	0.016107	Ass1/Cyp1b1/Ddah2/Il1b/Klf2/LOC497963/Nos2/Ptgs2/Sirpa/Slc7a2/Tlr2/Tlr5/Tlr6/Tnf	14
GO:0061844	antimicrobial humoral immune response mediated by antimicrobial peptide	15/1242	104/18303	0.004356	0.023756	0.016247	Camp/Ccl20/Ccl22/Cxcl1/Cxcl10/Cxcl11/Cxcl12/Cxcl2/Cxcl3/Lgals3/Nod2/Pglyrp1/Pglyrp4/Tslp/Xcl1	15
GO:0098773	skin epidermis development	15/1242	104/18303	0.004356	0.023756	0.016247	Cdh3/Dll1/Edar/Fgfr2/Foxn1/Fuz/Hpse/Igfbp5/Inhba/Lgr5/Nsdhl/Rbpj/Snai1/Tnf/Wnt5a	15
GO:0010952	positive regulation of peptidase activity	23/1242	188/18303	0.004374	0.023831	0.016298	Atp2a3/Casp12/Cflar/Cldn3/Efna1/Egln3/Epha4/F3/Fbln1/Hip1r/Lgals9/Ngf/Nlrc4/Nlrp3/Prr7/Pycard/Ripk2/Serpinb3/Stat1/Syk/Tnf/Tnfsf15/Vsir	23
GO:0048645	animal organ formation	12/1242	75/18303	0.004415	0.023967	0.016391	Bmp2/Bmp4/Bmp7/Fgfr2/Gata6/Hand2/Mef2c/Nkx3-2/Pax8/Rbpj/Wnt2b/Wnt5a	12
GO:0051057	positive regulation of small GTPase mediated signal transduction	12/1242	75/18303	0.004415	0.023967	0.016391	Adcyap1r1/Arhgef3/Col3a1/Csf1/F11r/Gpr4/Kitlg/Net1/Ngf/Nrg1/RGD1560455/Rasgrp1	12
GO:0042107	cytokine metabolic process	5/1242	17/18303	0.004437	0.023967	0.016391	Ereg/Rnf128/Syk/Tnfsf15/Trem3	5
GO:0043374	CD8-positive, alpha-beta T cell differentiation	5/1242	17/18303	0.004437	0.023967	0.016391	Irf1/Pax1/Satb1/Tnfsf8/Tox	5
GO:0045591	positive regulation of regulatory T cell differentiation	5/1242	17/18303	0.004437	0.023967	0.016391	Dusp10/Lgals9/Lilrb4/Sox12/Vsir	5
GO:0061042	vascular wound healing	5/1242	17/18303	0.004437	0.023967	0.016391	Cxcr4/Gata2/Hpse/Ndnf/Tnf	5
GO:0070243	regulation of thymocyte apoptotic process	5/1242	17/18303	0.004437	0.023967	0.016391	Ada/Blm/Bmp4/Efna1/Wnt5a	5
GO:0086012	membrane depolarization during cardiac muscle cell action potential	5/1242	17/18303	0.004437	0.023967	0.016391	Ank3/Cacna1g/Cacnb2/Gja5/Scn2b	5
GO:1904748	regulation of apoptotic process involved in	5/1242	17/18303	0.004437	0.023967	0.016391	Bmp7/Foxc1/Hnf1b/Pax8/Tnfrsf1b	5
GO:0007520	myoblast fusion	9/1242	48/18303	0.004484	0.02413	0.016503	Cd53/Cflar/Cxcl10/Cxcl12/Ehd2/Ripor2/Flot1/Flt3lg/G	9
GO:0010718	positive regulation of epithelial to mesenchymal transition	9/1242	48/18303	0.004484	0.02413	0.016503	Bmp2/Bmp4/Bmp7/Col1a1/Foxc1/Il1b/Rgcc/Serpinb3/Snai1	9
GO:0046638	positive regulation of alpha-beta T cell differentiation	9/1242	48/18303	0.004484	0.02413	0.016503	Ada/Cd83/Ikzf1/Il23a/Lgals9/Nlrp3/Myb/Ripk2/Syk	9
GO:0003272	endocardial cushion formation	6/1242	24/18303	0.004496	0.02413	0.016503	Bmp5/Bmp7/Rbpj/Snai1/Snai2/Tmem100	6
GO:0015701	bicarbonate transport	6/1242	24/18303	0.004496	0.02413	0.016503	Ca4/Slc26a10/Slc26a4/Slc26a9/Slc4a11/Slc4a7	6
GO:0022038	corpus callosum development	6/1242	24/18303	0.004496	0.02413	0.016503	Ephb2/Ephb3/Herc1/Prdm8/Rtn4rl2/Zeb2	6
GO:1901623	regulation of lymphocyte chemotaxis	6/1242	24/18303	0.004496	0.02413	0.016503	Ccl2/Ccl3/Ccl5/Cxcl10/Wnt5a/Xcl1	6
GO:0002708	positive regulation of lymphocyte mediated immunity	18/1242	135/18303	0.004501	0.024134	0.016506	Cadm1/Cd40/Clcf1/Exosc6/Fzd5/Hspa1b/Il18rap/Il1b/Il1r1/Il23a/Mr1/Nlrp3/Nod2/Pvr/Rasgrp1/Rsad2/Tnf/Xcl1	18
GO:0046661	male sex differentiation	27/1242	233/18303	0.004558	0.024417	0.0167	Abcb1a/Bmp5/Ccnd1/Col9a3/Csmd1/Cyp1b1/Dhcr24/Ercc1/Fdps/Fgf9/Gata6/Hmga2/Hmgcs1/Il1a/Inha/Inhba/Kif18a/Kitlg/Map7/Pdgfb/Sfrp1/Star/Tbx3/Tlr3/Tlr5/Wnt2b/Wnt5a	27
GO:2000514	regulation of CD4-positive, alpha-beta T cell activation	11/1242	66/18303	0.004577	0.024476	0.01674	Cd274/Cd44/Cd83/Il23a/Lgals9/Nlrp3/Myb/Ripk2/Tnfsf18/Vsir/Xcl1	11
GO:0016202	regulation of striated muscle tissue development	21/1242	167/18303	0.004578	0.024476	0.01674	Bdnf/Bmp2/Bmp4/Col14a1/Cyp26b1/Dll1/Fdps/Fgf9/Fgfr2/Flot1/Gata6/Gli1/Hdac9/Hey2/Hmgcr/Kcnk2/Mef2c/Ncam1/Nrg1/Pi16/Rbpj	21
GO:0010463	mesenchymal cell proliferation	10/1242	57/18303	0.00462	0.024683	0.016881	Bmp2/Bmp4/Bmp7/Chrd/Fat4/Fgf9/Fgfr2/Gas1/Hand2/Wnt5a	10
GO:0032680	regulation of tumor necrosis factor production	17/1242	125/18303	0.004634	0.024702	0.016894	Ccl2/Ccl3/Gstp1/Il23a/Lbp/Lgals9/Nod2/Ptger4/Pycard/Rasgrp1/Ripk2/Sirpa/Tlr2/Tlr3/Tnfaip3/Twist2/Vsir	17
GO:0030101	natural killer cell activation	13/1242	85/18303	0.004637	0.024702	0.016894	Coro1a/Fgr/Flt3lg/Ikzf1/Il15/Il15ra/Il23a/Lgals9/Pglyrp1/Pglyrp4/Prdm1/Rasgrp1/Tox	13
GO:0072088	nephron epithelium morphogenesis	13/1242	85/18303	0.004637	0.024702	0.016894	Agtr1a/Bmp2/Bmp4/Cd44/Fat4/Hnf1b/Irx3/Lif/Npnt/Pax8/Pgf/Wnk4/Wnt2b	13

ID	Description	GeneRatio	BgRatio	pvalue	p.adjust	qvalue	geneID	Count
GO:0008584	male gonad development	24/1242	200/18303	0.004673	0.024872	0.01701	Abcb1a/Ccnd1/Col9a3/Csmd1/Cyp1b1/Ercc1/Fdps/Fgf9/Gata6/Hmga2/Hmgcs1/Il1a/Inha/Inhba/Kif18a/Kitlg/Map7/Pdgfb/Sfrp1/Star/Tlr3/Tlr5/Wnt2b/Wnt5a	24
GO:0051897	positive regulation of protein kinase B signaling	16/1242	115/18303	0.004728	0.025	0.017098	C1qtnf1/C1qtnf3/Ccl3/Csf3/Cx3cl1/F3/Fam110c/Gdf15/Hpse/Igfbp5/Mst1r/Nrg1/Pik3r5/Sema5a/Thbs1/Tnf	16
GO:0003139	secondary heart field specification	4/1242	11/18303	0.004732	0.025	0.017098	Bmp4/Mef2c/Rbpj/Wnt5a	4
GO:0010692	regulation of alkaline phosphatase activity	4/1242	11/18303	0.004732	0.025	0.017098	Itga2/Mef2c/Npnt/Tnf	4
GO:0033029	regulation of neutrophil apoptotic process	4/1242	11/18303	0.004732	0.025	0.017098	Cd44/Hcar2/Pik3cb/Slc7a11	4
GO:0033034	positive regulation of myeloid cell apoptotic process	4/1242	11/18303	0.004732	0.025	0.017098	Cd44/Mef2c/Hcar2/Pik3cb	4
GO:0034699	response to luteinizing hormone	4/1242	11/18303	0.004732	0.025	0.017098	Cyp1b1/Pcsk5/Plat/Star	4
GO:0071864	positive regulation of cell proliferation in bone marrow	4/1242	11/18303	0.004732	0.025	0.017098	Fgfr2/Flt3lg/Hmga2/Mef2c	4
GO:0090037	positive regulation of protein kinase C signaling	4/1242	11/18303	0.004732	0.025	0.017098	Adgrv1/Cd40/Flt4/Wnt5a	4
GO:0010771	negative regulation of cell morphogenesis involved in differentiation	15/1242	105/18303	0.004772	0.025188	0.017227	Dnm3/Efna1/Ephb2/Fbln1/Meltf/Plxna3/Rgma/Sema3f/Sema4a/Sema4b/Sema5a/Sema6a/Sema6d/Slit2/Wnt5a	15
GO:0002824	positive regulation of adaptive immune response based on somatic recombination of immune receptors built from immunoglobulin	18/1242	136/18303	0.004868	0.025647	0.01754	Ada/Cd274/Cd40/Clcf1/Exosc6/Fzd5/Hspa1b/Il1b/Il1r1/Il23a/Mr1/Nlrp3/Nod2/Pvr/Ripk2/Rsad2/Tnf/Xcl1	18
GO:0014065	phosphatidylinositol 3-kinase signaling	18/1242	136/18303	0.004868	0.025647	0.01754	Ccl5/Csf2/Csf3/Dcn/Fgr/Flt1/Htr2a/Nrg1/Pdgfb/Pik3c2b/Pik3cb/Pik3ip1/Pik3r5/Ppard/Rasgrp1/Ror1/Selp/Tnf	18
GO:0032891	negative regulation of organic acid transport	7/1242	32/18303	0.004886	0.025694	0.017573	Acsl4/Adora1/Il1b/Il1rn/Pla2r1/Thbs1/Tnf	7
GO:0060561	apoptotic process involved in morphogenesis	7/1242	32/18303	0.004886	0.025694	0.017573	Bmp7/Foxc1/Fzd5/Hand2/Hnf1b/Pax8/Tnfrsf1b	7
GO:0051651	maintenance of location in cell	26/1242	223/18303	0.004896	0.025725	0.017594	Ank2/Ank3/Ankrd13c/Atp7b/Bdkrb1/Casq1/Ccl3/Cemip/Coro1a/Cx3cl1/Cxcl10/Cxcl11/Drd1/Hk2/Htr2a/Insig1/Itgb3/Mcoln2/Mcoln3/Ngf/P2ry6/Ryr2/Ryr3/Scin/Twf	26
GO:0051588	regulation of neurotransmitter transport	21/1242	168/18303	0.004907	0.025758	0.017616	Adora2a/Adra2a/Cacnb2/Cask/Cplx4/Cspg5/Drd1/Flot1/Htr2a/Htr7/Itgb3/Kcnh1/Mef2c/Nat8l/Ngf/Nrn1/Nrxn3/P2ry2/Ptger4/Rap1b/Sncaip	21
GO:0051592	response to calcium ion	22/1242	179/18303	0.00496	0.026015	0.017792	Adcy8/Adgrv1/Alox15/Ccnd1/Cpne4/Cpne8/Cyp27b1/Grin2a/Hcn1/Jun/Junb/Kcnh1/Mef2c/Mgp/Mylk/Pcsk1/Penk/Ppif/Ptges/Ryr2/Thbs1/Wnt5a	22
GO:0046546	development of primary male sexual characteristics	24/1242	201/18303	0.004975	0.026072	0.017831	Abcb1a/Ccnd1/Col9a3/Csmd1/Cyp1b1/Ercc1/Fdps/Fgf9/Gata6/Hmga2/Hmgcs1/Il1a/Inha/Inhba/Kif18a/Kitlg/Map7/Pdgfb/Sfrp1/Star/Tlr3/Tlr5/Wnt2b/Wnt5a	24
GO:0031644	regulation of nervous system process	23/1242	190/18303	0.004982	0.02608	0.017837	Adora1/Ccl3/Cnih2/Dlgap2/Ednrb/Fabp5/Glra1/Grin2a/Igsf9b/Il1a/Itga2/Itgax/Mgll/Myrf/Ncam1/Nptxr/Nrg1/Shank1/Tenm4/Tmem100/Tmem25/Tnf/Tnfrsf1b	23
GO:0035904	aorta development	11/1242	67/18303	0.005148	0.026877	0.018382	Adamts6/Col3a1/Cxcr4/Fuz/Hey2/Mylk/Pde2a/Plxnd1/Prdm1/Prickle1/Rbpj	11
GO:0046579	positive regulation of Ras protein signal transduction	11/1242	67/18303	0.005148	0.026877	0.018382	Arhgef3/Col3a1/Csf1/F11r/Gpr4/Kitlg/Net1/Ngf/Nrg1/RGD1560455/Rasgrp1	11
GO:2000107	negative regulation of leukocyte apoptotic process	11/1242	67/18303	0.005148	0.026877	0.018382	Ada/Blm/Bmp4/Ccl5/Cd44/Cxcl12/Dock8/Efna1/Kitlg/Nod2/Serpinb9	11
GO:0021983	pituitary gland development	9/1242	49/18303	0.005169	0.026889	0.01839	Bmp2/Bmp4/Ets1/Gata2/Gli1/Hmga2/Pcsk1/Rbpj/Wnt5a	9
GO:0030517	negative regulation of axon extension	9/1242	49/18303	0.005169	0.026889	0.01839	Plxna3/Sema3f/Sema4a/Sema4b/Sema5a/Sema6a/Sema6d/Slit2/Wnt5a	9
GO:0043090	amino acid import	9/1242	49/18303	0.005169	0.026889	0.01839	Slc1a1/Slc1a2/Slc22a4/Slc47a1/Slc7a11/Slc7a2/Slc7a3/Slc7a8/Tnf	9
GO:1902041	regulation of extrinsic apoptotic signaling pathway via death domain	9/1242	49/18303	0.005169	0.026889	0.01839	Atf3/Cflar/Itprip/Lgals3/Mal/Sfrp1/Thbs1/Timp3/Tnfaip3	9
GO:0060993	kidney morphogenesis	15/1242	106/18303	0.005219	0.02711	0.018541	Agtr1a/Bmp2/Bmp4/Bmp7/Cd44/Fat4/Gcnt1/Hnf1b/Irx3/Lif/Npnt/Pax8/Pgf/Wnk4/Wnt2b	15
GO:0046888	negative regulation of hormone secretion	14/1242	96/18303	0.005221	0.02711	0.018541	Adora1/Adra2a/Hadh/Hmgcr/Il11/Il1b/Inha/Lif/Npy1r/Nrg1/Rab11fip1/Sfrp1/Ucn2/Vsnl1	14

ID	Description	GeneRatio	BgRatio	pvalue	p.adjust	qvalue	geneID	Count
GO:1901654	response to ketone	41/1242	399/18303	0.005251	0.027171	0.018583	Aacs/Abcb1a/Abcb1b/Adcy8/Agtr1a/Akr1b1/Aqp1/Ass1/Bmp4/Cacna1g/Ccl2/Ccnd1/Cflar/Cyp1b1/Cyp27b1/Errfi1/Fdps/Fibin/Fosl1/Gjb2/Htr7/Il1b/Acod1/Junb/Klf2/Klf6/Nos2/Nqo1/Nrg1/P2ry6/Plat/Ptger4/Ramp2/Sfrp1/Slit2/Slit3/Socs3/Star/Thbs1/Tlr2/Wnt5a	41
GO:0006636	unsaturated fatty acid biosynthetic process	10/1242	58/18303	0.005251	0.027171	0.018583	Abcd1/Alox15/Bdkrb1/Elovl4/Fabp5/Gstp1/Il1b/Ptges/Ptgs2/Scd	10
GO:0043536	positive regulation of blood vessel endothelial cell migration	10/1242	58/18303	0.005251	0.027171	0.018583	Angpt4/Cd40/Ets1/Gata2/Hdac9/Pdgfb/Plk2/Ptgs2/Rhoj/Thbs1	10
GO:1903556	negative regulation of tumor necrosis factor superfamily cytokine production	10/1242	58/18303	0.005251	0.027171	0.018583	Cd274/Gstp1/Lbp/Lgals9/Nod2/Ptger4/Sirpa/Tnfaip3/Twist2/Vsir	10
GO:0048872	homeostasis of number of cells	35/1242	328/18303	0.005308	0.027441	0.018767	Ada/Ampd3/Bmp4/Card11/Cd44/Cfh/Col14a1/Coro1a/Csf1/Dock10/Ets1/Flt3lg/Foxn1/Gata2/Hspa1b/Ikzf1/Inhba/Isg15/Kitlg/Klf2/Lgals9/Lmo2/Mafb/Map7/Mef2c/Hcar2/Pde4b/Pik3cb/Pla2g2a/Myb/Sh2b2/Slc7a11/Stat1	35
GO:0021537	telencephalon development	36/1242	340/18303	0.005365	0.027708	0.01895	Anxa3/Aqp1/Bcl2a1/Bmp2/Bmp4/Btg2/Cask/Col3a1/Cxcl12/Cxcr4/Drd1/Ephb2/Ephb3/Fat4/Fez1/Grin2a/Herc1/Hsd17b7/Igf2bp1/Inhba/Lrp8/Mef2c/Nefh/Nr2f1/Nrg1/Phactr1/Plcb1/Plxna3/Prdm8/Rtn4rl2/Slc17a6/Slc1a2/Slc7a11/Slit2/Wnt5a/Zeb2	36
GO:0060348	bone development	27/1242	236/18303	0.005413	0.027932	0.019103	Bmp2/Bmp4/Cadm1/Col13a1/Col1a1/Col2a1/Cyp26b1/Dhrs3/Fam20c/Fat4/Fgfr2/Fli1/Foxc1/Frem1/Gpr68/Has2/Insig1/Mef2c/Mmp13/Mmp16/Pax1/Ptger4/Scx/Thbs1/Trpv4/Ttc9/Xylt1	27
GO:0014902	myotube differentiation	17/1242	127/18303	0.005447	0.028083	0.019206	Barx2/Bdnf/Cd53/Cflar/Cxcl10/Cxcl12/Cyp26b1/Ehd2/Ripor2/Flot1/Flt3lg/Gdf15/Hdac9/Mef2c/Pmp22/Rbm3	17
GO:0048844	artery morphogenesis	12/1242	77/18303	0.005485	0.028255	0.019324	Bmp4/Col3a1/Foxc1/Gja5/Hand2/Hey2/Hpgd/Ldlr/Mylk/Prdm1/Ptger4/Rbpj	12
GO:0009895	negative regulation of catabolic process	34/1242	317/18303	0.005497	0.028291	0.019349	Adora1/Adra2a/Bmf/Cyp51/Derl3/Efna1/Elavl4/Epha4/Tent5b/Tent5c/Fez1/Furin/Grin2a/Herc1/Hfe/Hipk2/Hmgcr/Ier3/Igf2bp1/Il1b/Mgat3/Nampt/Hcar2/Noct/Nos2/Nrg1/Ppargc1a/Rbm38/Rilp/Rybp/Smcr8/Timp3/Tnf/Yod	34
GO:0021700	developmental maturation	35/1242	329/18303	0.005565	0.028547	0.019524	Akr1b1/Ank3/Bfsp1/Bmp2/Cabyr/Cd44/Cdh3/Cspg4/Ednrb/Ereg/Fam20c/Fat4/Fzd5/Gata2/Il15/Klf2/Ngf/Ntn4/Palm/Pdgfb/Pmp22/Prkar2a/Pth1r/Rab38/Rbpj/Rfx3/Scarf1/Sez6l/Shank1/Sptbn4/Sybu/Tcp11/Wnt5a/Xylt1/Zd	35
GO:0006700	C21-steroid hormone biosynthetic process	6/1242	25/18303	0.005582	0.028547	0.019524	Bmp2/Bmp5/Cyp11b2/Cyp27a1/Egr1/Ppargc1a	6
GO:0010742	macrophage derived foam cell differentiation	6/1242	25/18303	0.005582	0.028547	0.019524	Csf1/Csf2/Itgb3/Nfkbia/Pla2g5/Stat1	6
GO:0032930	positive regulation of superoxide anion generation	6/1242	25/18303	0.005582	0.028547	0.019524	Agtr1a/Cxcl1/Gstp1/Hvcn1/Olr1/Syk	6
GO:0061298	retina vasculature development in camera-type eye	6/1242	25/18303	0.005582	0.028547	0.019524	Col4a1/Cyp1b1/Fzd4/Pdgfb/Rhoj/Slc4a7	6
GO:0090077	foam cell differentiation	6/1242	25/18303	0.005582	0.028547	0.019524	Csf1/Csf2/Itgb3/Nfkbia/Pla2g5/Stat1	6
GO:2000810	regulation of bicellular tight	6/1242	25/18303	0.005582	0.028547	0.019524	Cldn3/F11r/Fzd5/Snai1/Snai2/Tnf	6
GO:0051604	protein maturation	32/1242	294/18303	0.00559	0.028567	0.019537	Atp7b/Birc3/C1rl/Casp12/Cpd/Cpz/Dhcr24/Ero1b/F3/Furin/Fuz/Gas1/Ggt1/LOC100910418/Meltf/Mmp16/Myrf/Nlrc4/Pcsk1/Pcsk5/Pcsk9/Plat/Plau/Ppp1r15a/Pycard/Ripk2/Serpinf2/Spon1/Tasp1/Thbs1/Vsir/Yae1	32
GO:0030516	regulation of axon extension	16/1242	117/18303	0.005599	0.028585	0.01955	Bdnf/Cdh4/Cxcl12/Mgll/Ngf/Nrg1/Plxna3/Sema3f/Sema4a/Sema4b/Sema5a/Sema6a/Sema6d/Slit2/Twf2/Wnt5	16
GO:0045834	positive regulation of lipid metabolic process	21/1242	170/18303	0.005623	0.028663	0.019603	Abcd1/Acsl3/Adora1/Agtr1a/Cpt1a/Fdps/Fgr/Flt1/Htr2a/Il1a/Il1b/Ldlr/Nod2/Pdgfb/Ppard/Ppargc1a/Ptgs2/Rab38/Star/Stard4/Tnf	21
GO:1904037	positive regulation of epithelial cell apoptotic process	8/1242	41/18303	0.005624	0.028663	0.019603	Bmp4/Ccl2/Cd40/Pla2r1/Ppargc1a/Rgcc/Thbs1/Tnf	8
GO:0001654	eye development	45/1242	449/18303	0.005635	0.028693	0.019624	Aqp1/Bdnf/Bfsp1/Bmp4/Bmp7/Cfh/Col4a1/Col5a1/Col8a2/Cyp1b1/Dll1/Ephb2/Fat3/Fgf9/Fgfr2/Flt1/Foxc1/Fzd4/Fzd5/Gas1/Hcn1/Hipk2/Ikzf1/Inhba/Jun/Angptl7/Lif/Nectin1/Obsl1/Pdgfb/Prdm1/Rhoj/Rorb/Slc17a6/Slc25a25/Slc4a7/Slc7a11/Smoc1/Tgif1/Thrb/Tmod1/Tub/Twis	45
GO:0033555	multicellular organismal response to stress	15/1242	107/18303	0.0057	0.028996	0.019831	Adcyap1r1/Adra2a/Als2/Asic4/Bdnf/Drd1/Ednrb/Gch1/Htr7/Mef2c/Ncam1/Penk/Thbs1/Thbs4/Vwa1	15
GO:0021954	central nervous system neuron development	14/1242	97/18303	0.005731	0.029128	0.019921	Btg2/Drd1/Epha4/Ephb2/Ephb3/Fgfr2/Gata2/Gbx2/Plxna3/Prdm8/Slit2/Sptbn4/Wnt5a/Zeb2	14
GO:0002931	response to ischemia	11/1242	68/18303	0.005774	0.029219	0.019983	Bves/Cpeb4/Csf1/Cx3cl1/Egr1/Gjb2/Hk2/Ndnf/Nqo1/Ppargc1a/Ppif	11

ID	Description	GeneRatio	BgRatio	pvalue	p.adjust	qvalue	geneID	Count
GO:0046637	regulation of alpha-beta T cell differentiation	11/1242	68/18303	0.005774	0.029219	0.019983	Ada/Cd83/Ikzf1/Il23a/Lgals9/Nlrp3/Prdm1/Myb/Ripk2/Syk/Tnfsf18	11
GO:0050994	regulation of lipid catabolic process	11/1242	68/18303	0.005774	0.029219	0.019983	Abcd1/Adora1/Adra2a/Cpt1a/Hpgd/Il1b/Ldlr/Hcar2/Rarres2/Thra/Tnf	11
GO:0002467	germinal center formation	5/1242	18/18303	0.005804	0.029219	0.019983	Ada/Bcl3/Mef2c/Nfkb2/Tnfaip3	5
GO:0003128	heart field specification	5/1242	18/18303	0.005804	0.029219	0.019983	Bmp2/Bmp4/Mef2c/Rbpj/Wnt5a	5
GO:0003159	morphogenesis of an endothelium	5/1242	18/18303	0.005804	0.029219	0.019983	Bmp4/Cxcl10/Cxcr4/Itgax/Rbpj	5
GO:0010623	programmed cell death involved in cell development	5/1242	18/18303	0.005804	0.029219	0.019983	Bdnf/Il1a/Il1b/Kitlg/Slc4a7	5
GO:0060572	morphogenesis of an epithelial bud	5/1242	18/18303	0.005804	0.029219	0.019983	Bmp4/Bmp7/Fgfr2/Wnt2b/Wnt5a	5
GO:0060707	trophoblast giant cell differentiation	5/1242	18/18303	0.005804	0.029219	0.019983	Lif/Mdfi/Prdm1/Snai1/Socs3	5
GO:0061154	endothelial tube morphogenesis	5/1242	18/18303	0.005804	0.029219	0.019983	Bmp4/Cxcl10/Cxcr4/Itgax/Rbpj	5
GO:1902894	negative regulation of pri-miRNA transcription by RNA polymerase II	5/1242	18/18303	0.005804	0.029219	0.019983	Bmp4/Gata6/Lilrb4/Pdgfb/Ppard	5
GO:0018149	peptide cross-linking	7/1242	33/18303	0.005845	0.029244	0.02	Col3a1/Dcn/F13a1/Mamdc2/Ndnf/Tgm2/Thbs1	7
GO:0035590	purinergic nucleotide receptor signaling pathway	7/1242	33/18303	0.005845	0.029244	0.02	Ada/Adora1/Adora2a/Hcar2/P2ry14/P2ry2/P2ry6	7
GO:0040036	regulation of fibroblast growth factor receptor	7/1242	33/18303	0.005845	0.029244	0.02	Creb3l1/Fam20c/Fuz/Pdgfb/Spry4/Thbs1/Wnt5a	7
GO:0045742	positive regulation of epidermal growth factor receptor signaling pathway	7/1242	33/18303	0.005845	0.029244	0.02	Adora1/Adra2a/Areg/Ereg/Hip1r/Mmp9/Plaur	7
GO:0051150	regulation of smooth muscle cell differentiation	7/1242	33/18303	0.005845	0.029244	0.02	Bmp4/Cth/Ereg/Fgf9/Fgfr2/Pdgfb/Prdm6	7
GO:0055094	response to lipoprotein particle	7/1242	33/18303	0.005845	0.029244	0.02	Cd68/F3/Hmgcs1/Ldlr/Mmp9/Syk/Tlr6	7
GO:0060740	prostate gland epithelium morphogenesis	7/1242	33/18303	0.005845	0.029244	0.02	Bmp4/Bmp7/Cd44/Cyp7b1/Fgfr2/Sfrp1/Wnt5a	7
GO:0060996	dendritic spine development	17/1242	128/18303	0.005895	0.029472	0.020156	Acsl4/Cask/Ctnnd2/Dnm3/Dock10/Efna1/Epha4/Ephb2/Ephb3/Kif1a/Lrp8/Mef2c/Nrg1/Palm/Plk2/Shank1/Zdh	17
GO:0045429	positive regulation of nitric oxide biosynthetic process	9/1242	50/18303	0.005933	0.029554	0.020213	Ass1/Ddah2/Il1b/Klf2/Ptgs2/Tlr2/Tlr5/Tlr6/Tnf	9
GO:0002720	positive regulation of cytokine production involved in immune response	10/1242	59/18303	0.005948	0.029554	0.020213	Fzd5/Il1b/Il1r1/Nlrp3/Nod2/Rsad2/Tlr2/Trim6/Wnt5a/Xcl1	10
GO:0010517	regulation of phospholipase	10/1242	59/18303	0.005948	0.029554	0.020213	Adcyap1r1/Agtr1a/Anxa8/Ccl5/Fgfr2/Flt1/Gna13/Htr2a/Pla2g5/Pla2r1	10
GO:0030042	actin filament depolymerization	10/1242	59/18303	0.005948	0.029554	0.020213	Mical1/Plek/Cracd/Scin/Sema5a/Shroom2/Sptbn4/Tmod1/Tmod2/Twf2	10
GO:0035315	hair cell differentiation	10/1242	59/18303	0.005948	0.029554	0.020213	Clic5/Dll1/Ripor2/Hey2/Mcoln3/Mycl/Myo7a/Rbpj/Slc4a7/Whrn	10
GO:0046622	positive regulation of organ growth	10/1242	59/18303	0.005948	0.029554	0.020213	Fdps/Fgf9/Fgfr2/Gata6/Gli1/Hey2/Mef2c/Ncam1/Nrg1/Rbpj	10
GO:1900449	regulation of glutamate receptor signaling pathway	10/1242	59/18303	0.005948	0.029554	0.020213	Ccl2/Cnih2/Cx3cl1/Ephb2/Grin2a/Mef2c/Ppargc1a/Prrt1/Rgs9/Shank1	10
GO:0042391	regulation of membrane potential	47/1242	475/18303	0.006002	0.029776	0.020364	Abcd1/Adora1/Adora2a/Alox15/Ank2/Ank3/Bdnf/Bves/Cacna1g/Cacnb2/Cnih2/Cx3cl1/Dcn/Drd1/Fhl1/Gclc/Gja5/Glra1/Glrb/Gna14/Gpr88/Grin2a/Hcn1/Igsf9b/Il1rn/Jun/Kcnh1/Kcnh2/Kcnk2/Kcnk3/Kcnk5/Kcnn4/Mef2c/Mpp2/Nalcn/Piezo2/Pmp22/Pycr1/Rgs7/Rnf122/Ryr2/Scn2b/Shank1/Tmem25/Tnf/Trpv4/Xirp1	47
GO:0010950	positive regulation of endopeptidase activity	21/1242	171/18303	0.006013	0.029776	0.020364	Atp2a3/Casp12/Cflar/Efna1/Egln3/Epha4/F3/Hip1r/Lgals9/Ngf/Nlrc4/Nlrp3/Prr7/Pycard/Ripk2/Serpinb3/Stat1/Syk/Tnf/Tnfsf15/Vsir	21
GO:0048634	regulation of muscle organ development	21/1242	171/18303	0.006013	0.029776	0.020364	Bdnf/Bmp2/Bmp4/Col14a1/Cyp26b1/Dll1/Fdps/Fgf9/Fgfr2/Flot1/Gata6/Gli1/Hdac9/Hey2/Hmgcr/Kcnk2/Mef2c/Ncam1/Nrg1/Pi16/Rbpj	21
GO:1904950	negative regulation of establishment of protein localization	21/1242	171/18303	0.006013	0.029776	0.020364	Adra2a/Coro2b/Cx3cl1/Cyp51/Derl3/Frmd4a/Hadh/Hdac9/Hmgcr/Il1b/Il33/Insig1/Ptger4/RGD1311251/Rab11fip1/Rgcc/Rsad2/Sfrp1/Siglec10/Vsnl1/Yod1	21
GO:0002792	negative regulation of peptide secretion	16/1242	118/18303	0.006081	0.030059	0.020558	Adra2a/Cx3cl1/Cyp51/Frmd4a/Hadh/Hdac9/Hmgcr/Il1b/Il33/Ptger4/Rab11fip1/Rgcc/Rsad2/Sfrp1/Siglec10/Vsn	16

ID	Description	GeneRatio	BgRatio	pvalue	p.adjust	qvalue	geneID	Count
GO:0071869	response to catecholamine	16/1242	118/18303	0.006081	0.030059	0.020558	Bdnf/Drd1/Egr1/Gna14/Htr2a/Htr7/Il1rn/Nsg1/Palm/Pde4b/Penk/Ppargc1a/Ptger1/Rgs9/Ryr2/Star	16
GO:0002066	columnar/cuboidal epithelial cell development	12/1242	78/18303	0.006093	0.030067	0.020563	Bmp4/Bmp5/Clic5/Dll1/Ripor2/Myo7a/Nkx3-2/Prdm1/Rfx3/Slc4a7/Whrn/Wnt5a	12
GO:0099601	regulation of neurotransmitter receptor activity	12/1242	78/18303	0.006093	0.030067	0.020563	Ccl2/Cnih2/Dlgap2/Ephb2/Grin2a/Lynx1/Mef2c/Nptxr/Ppargc1a/Prrt1/Rgs9/Shank1	12
GO:0002449	lymphocyte mediated immunity	32/1242	296/18303	0.006173	0.030435	0.020815	Bcl3/C1rl/Cadm1/Cd40/Clcf1/Coro1a/Ercc1/Exosc6/Fzd5/Hfe/Hspa1b/Il13ra2/Il18rap/Il1b/Il1r1/Il23a/Il31ra/Lgals9/Mr1/Nlrp3/Nod2/Pvr/Rasgrp1/Rnf19b/Rsad2/Serpinb3a/Serpinb9/Serping1/Tnf/Tnfrsf1b/Vsir/Xcl1	32
GO:0042310	vasoconstriction	15/1242	108/18303	0.006215	0.030619	0.020941	Adra2a/Agtr1a/Bdkrb2/Cacna1g/Cx3cl1/Dock4/Drd1/Dusp5/Ednrb/Gja5/Hspa1b/Htr2a/Htr7/Npy1r/Ptgs2	15
GO:0046677	response to antibiotic	14/1242	98/18303	0.006279	0.03091	0.02114	Abcb1a/Ccl2/Ciita/Cpt1a/Crip1/Egr1/Ets1/Gjb2/Grin2a/Hyal1/Il1b/Mef2c/Ppp1r15a/Star	14
GO:0070997	neuron death	44/1242	440/18303	0.006387	0.031412	0.021483	Adora1/Adora2a/Bdnf/Btg2/Ccl2/Ccl3/Ccl5/Cd200r1/Clcf1/Coro1a/Cpeb4/Csf1/Csf3/Cx3cl1/Cxcl2/Egln3/Egr1/Gclc/Gpr75/Hipk2/Il1b/Jun/LOC100911625/Mef2c/Nampt/Ndnf/Ngf/Nqo1/Pcp4/Pcsk9/Plau/Ppargc1a/Ptpn5/Myb/Rapsn/Rel/Slc1a1/Slc7a11/Star/Thrb/Tlr6/Tnf/Tnfr	44
GO:0048512	circadian behavior	11/1242	69/18303	0.006459	0.031737	0.021706	Ada/Adora1/Adora2a/Csf2/Drd1/Egr1/Hcrtr2/Htr7/Pglyrp1/Ptger4/Star	11
GO:0010092	specification of animal organ identity	8/1242	42/18303	0.006543	0.032098	0.021953	Bmp2/Bmp4/Fgfr2/Mef2c/Pax8/Rbpj/Wnt2b/Wnt5a	8
GO:0120255	olefinic compound biosynthetic process	8/1242	42/18303	0.006543	0.032098	0.021953	Alox15/Bmp2/Bmp5/Cyp11b2/Egr1/Gstp1/Ppargc1a/Star	8
GO:0150063	visual system development	45/1242	453/18303	0.006594	0.032298	0.022089	Aqp1/Bdnf/Bfsp1/Bmp4/Bmp7/Cfh/Col4a1/Col5a1/Col8a2/Cyp1b1/Dll1/Ephb2/Fat3/Fgf9/Fgfr2/Flt1/Foxc1/Fzd4/Fzd5/Gas1/Hcn1/Hipk2/Ikzf1/Inhba/Jun/Angptl7/Lif/Nectin1/Obsl1/Pdgfb/Prdm1/Rhoj/Rorb/Slc17a6/Slc25a25/Slc4a7/Slc7a11/Smoc1/Tgif1/Thrb/Tmod1/Tub/Twis	45
GO:0071867	response to monoamine	16/1242	119/18303	0.006595	0.032298	0.022089	Bdnf/Drd1/Egr1/Gna14/Htr2a/Htr7/Il1rn/Nsg1/Palm/Pde4b/Penk/Ppargc1a/Ptger1/Rgs9/Ryr2/Star	16
GO:0014072	response to isoquinoline alkaloid	10/1242	60/18303	0.006714	0.032551	0.022262	Abcb1a/Ada/Adcy8/Ccl5/Cxcr4/Drd1/Egr1/Il1b/Pcsk1/Penk	10
GO:0045668	negative regulation of osteoblast differentiation	10/1242	60/18303	0.006714	0.032551	0.022262	Areg/Chrd/Hand2/Igfbp5/Noct/Rorb/Sfrp1/Tnf/Tnn/Twist2	10
GO:0032725	positive regulation of granulocyte macrophage colony-stimulating factor production	4/1242	12/18303	0.00672	0.032551	0.022262	Il1b/Il23a/Rasgrp1/Syk	4
GO:0032815	negative regulation of natural killer cell activation	4/1242	12/18303	0.00672	0.032551	0.022262	Fgr/Lgals9/Pglyrp1/Pglyrp4	4
GO:0046884	follicle-stimulating hormone secretion	4/1242	12/18303	0.00672	0.032551	0.022262	Inha/Inhba/Tbx3/Ucn2	4
GO:0048934	peripheral nervous system neuron differentiation	4/1242	12/18303	0.00672	0.032551	0.022262	Etv1/Hand2/Nefh/Pmp22	4
GO:0048935	peripheral nervous system neuron development	4/1242	12/18303	0.00672	0.032551	0.022262	Etv1/Hand2/Nefh/Pmp22	4
GO:0051798	positive regulation of hair follicle development	4/1242	12/18303	0.00672	0.032551	0.022262	Foxn1/Hpse/Tnf/Wnt5a	4
GO:0060536	cartilage morphogenesis	4/1242	12/18303	0.00672	0.032551	0.022262	Hand2/Mef2c/Snai1/Snai2	4
GO:0070391	response to lipoteichoic acid	4/1242	12/18303	0.00672	0.032551	0.022262	Ccl20/Lbp/Ripk2/Tlr2	4
GO:0071223	cellular response to lipoteichoic acid	4/1242	12/18303	0.00672	0.032551	0.022262	Ccl20/Lbp/Ripk2/Tlr2	4
GO:0150079	negative regulation of neuroinflammatory response	4/1242	12/18303	0.00672	0.032551	0.022262	Cd200r1/Cx3cl1/Ldlr/Tnfrsf1b	4
GO:1902287	semaphorin-plexin signaling pathway involved in axon guidance	4/1242	12/18303	0.00672	0.032551	0.022262	Plxna2/Plxna3/Plxnd1/Sema3f	4
GO:0007422	peripheral nervous system development	12/1242	79/18303	0.006752	0.03265	0.02233	Bdnf/Ednrb/Etv1/Fa2h/Hand2/Lama2/Nefh/Ngf/Nrg1/Pmp22/Sh3tc2/Slc5a3	12
GO:0007566	embryo implantation	12/1242	79/18303	0.006752	0.03265	0.02233	A1cf/Arhgdib/Fbln1/Acod1/Itgb3/Lif/Mmp12/Mmp9/Pcsk5/Plau/Ppard/Ptgs2	12
GO:0043618	regulation of transcription from RNA polymerase II promoter in response to stress	9/1242	51/18303	0.00678	0.032731	0.022386	Atf3/Bach1/Creb3l1/Egr1/Hspa1b/Jun/Klf2/Ppp1r15a/Rbpj	9

ID	Description	GeneRatio	BgRatio	pvalue	p.adjust	qvalue	geneID	Count
GO:2000725	regulation of cardiac muscle cell differentiation	9/1242	51/18303	0.00678	0.032731	0.022386	Bmp2/Bmp4/Col14a1/Dll1/Fdps/Mef2c/Nrg1/Pi16/Rbpj	9
GO:0043567	regulation of insulin-like growth factor receptor signaling pathway	6/1242	26/18303	0.006847	0.032999	0.022568	Bmp2/Bmp5/Cdh3/Igfbp4/Igfbp5/Igfbp6	6
GO:0060004	reflex	6/1242	26/18303	0.006847	0.032999	0.022568	Glra1/Glrb/Npnt/Pmp22/Satb1/Shank1	6
GO:0003203	endocardial cushion morphogenesis	7/1242	34/18303	0.006938	0.033379	0.022829	Bmp2/Bmp5/Bmp7/Rbpj/Snai1/Snai2/Tmem100	7
GO:0044849	estrous cycle	7/1242	34/18303	0.006938	0.033379	0.022829	Cyp1b1/Egr1/Enpp2/Ets1/Has2/Mmp13/Wnt5a	7
GO:0035725	sodium ion transmembrane transport	20/1242	163/18303	0.007294	0.035037	0.023963	Ank3/Asic4/Cacna1g/Hcn1/Nalcn/Pcsk9/Scn2b/Slc17a1/Slc17a3/Slc20a1/Slc24a3/Slc24a4/Slc4a11/Slc4a7/Slc6a12/Slc6a17/Slc9a9/Stom/Trpv4/Wnk4	20
GO:0098739	import across plasma membrane	20/1242	163/18303	0.007294	0.035037	0.023963	Abcc9/Agtr1a/Hfe/Kcnh2/Kcnk5/Slc1a1/Slc1a2/Slc22a4/Slc2a10/Slc47a1/Slc7a11/Slc7a2/Slc7a3/Slc7a8/Slc9a9/Thbs1/Tnf/Trpv3/Trpv4/Wnk4	20
GO:0043200	response to amino acid	28/1242	253/18303	0.00733	0.035178	0.024059	Abcb1a/Ass1/Bdnf/Ccl2/Ccl5/Col1a1/Col3a1/Col4a1/Cpeb4/Drd1/Gclc/Glra1/Glrb/Grin2a/Gstp1/Hcn1/Ipo5/Mmp3/Naip6/Nqo1/Pnpla3/Ppargc1a/Ptger4/Rragd/Sesn3/Slc1a2/Timp3/Tnf	28
GO:0002456	T cell mediated immunity	17/1242	131/18303	0.007423	0.035401	0.024211	Fzd5/Hfe/Hspa1b/Il18rap/Il1b/Il1r1/Il23a/Il31ra/Mr1/Nlrp3/Nod2/Pvr/Rsad2/Serpinb9/Tnfrsf1b/Vsir/Xcl1	17
GO:0032640	tumor necrosis factor production	17/1242	131/18303	0.007423	0.035401	0.024211	Ccl2/Ccl3/Gstp1/Il23a/Lbp/Lgals9/Nod2/Ptger4/Pycard/Rasgrp1/Ripk2/Sirpa/Tlr2/Tlr3/Tnfaip3/Twist2/Vsir	17
GO:0051101	regulation of DNA binding	17/1242	131/18303	0.007423	0.035401	0.024211	Bcl3/Foxc1/Hand2/Hey2/Hipk2/Hmga2/Itga2/Jun/Lgals9/Lif/Mdfi/Mmp9/Ngf/Plaur/Trim6/Twist2/Zbtb7c	17
GO:0033631	cell-cell adhesion mediated by integrin	5/1242	19/18303	0.007444	0.035401	0.024211	Ada/Ccl5/Itga5/Npnt/Plpp3	5
GO:0035970	peptidyl-threonine dephosphorylation	5/1242	19/18303	0.007444	0.035401	0.024211	Dusp10/Dusp16/Dusp5/Dusp6/Pdp2	5
GO:0040037	negative regulation of fibroblast growth factor receptor signaling pathway	5/1242	19/18303	0.007444	0.035401	0.024211	Creb3l1/Fuz/Spry4/Thbs1/Wnt5a	5
GO:0042754	negative regulation of circadian rhythm	5/1242	19/18303	0.007444	0.035401	0.024211	Ada/Adora1/Drd1/Htr7/Ptger4	5
GO:0070365	hepatocyte differentiation	5/1242	19/18303	0.007444	0.035401	0.024211	Bmp4/Frzb/Ggt1/Hnf1b/Itga2	5
GO:0071071	regulation of phospholipid biosynthetic process	5/1242	19/18303	0.007444	0.035401	0.024211	Acsl3/Fabp3/Htr2a/Pdgfb/Rab38	5
GO:0071871	response to epinephrine	5/1242	19/18303	0.007444	0.035401	0.024211	Pde4b/Penk/Ppargc1a/Ryr2/Star	5
GO:2001185	regulation of CD8-positive, alpha-beta T cell activation	5/1242	19/18303	0.007444	0.035401	0.024211	Cd274/Hfe/Irf1/Vsir/Xcl1	5
GO:0006919	activation of cysteine-type endopeptidase activity involved in	12/1242	80/18303	0.007466	0.035479	0.024265	Atp2a3/Cflar/Egln3/F3/Hip1r/Ngf/Nlrc4/Nlrp3/Pycard/Stat1/Tnf/Tnfsf15	12
GO:0010812	negative regulation of cell-substrate	10/1242	61/18303	0.007554	0.035839	0.024511	Cask/Col1a1/Coro2b/Enpp2/Fbln1/Fzd4/Meltf/Mmp12/Plet1/Thbs1	10
GO:0045824	negative regulation of innate immune response	10/1242	61/18303	0.007554	0.035839	0.024511	Ccr1/Dusp10/Acod1/Lgals9/Mmp12/Nlrc5/Serpinb3a/Serpinb9/Serping1/Tnfaip3	10
GO:0006024	glycosaminoglycan biosynthetic process	8/1242	43/18303	0.007572	0.035861	0.024526	Chst14/Has2/Hyal1/Il1b/Nfkb1/Pdgfb/Ptger4/Xylt1	8
GO:0050691	regulation of defense response to virus by host	8/1242	43/18303	0.007572	0.035861	0.024526	Il15/Il1b/Il23a/Micb/Mmp12/Pycard/Stat1/Trim6	8
GO:0009952	anterior/posterior pattern specification	26/1242	231/18303	0.00771	0.036487	0.024954	Bmp2/Bmp4/Btg2/Cobl/Dll1/Dmrt2/Foxc1/Fuz/Fzd5/Gbx2/Hey2/Hipk2/Hnf1b/Hoxb5/Hoxb8/Hoxc6/Meox2/Pax1/Pcsk5/Plxna2/Rbpj/Sfrp1/Tbx3/Wnt2b/Wnt5a/Zeb2	26
GO:1904407	positive regulation of nitric oxide metabolic process	9/1242	52/18303	0.007718	0.036493	0.024958	Ass1/Ddah2/Il1b/Klf2/Ptgs2/Tlr2/Tlr5/Tlr6/Tnf	9
GO:0048880	sensory system development	45/1242	458/18303	0.007984	0.03772	0.025798	Aqp1/Bdnf/Bfsp1/Bmp4/Bmp7/Cfh/Col4a1/Col5a1/Col8a2/Cyp1b1/Dll1/Ephb2/Fat3/Fgf9/Fgfr2/Flt1/Foxc1/Fzd4/Fzd5/Gas1/Hcn1/Hipk2/Ikzf1/Inhba/Jun/Angptl7/Lif/Nectin1/Obsl1/Pdgfb/Prdm1/Rhoj/Rorb/Slc17a6/Slc25a25/Slc4a7/Slc7a11/Smoc1/Tgif1/Thrb/Tmod1/Tub/Twis	45
GO:0002761	regulation of myeloid leukocyte differentiation	17/1242	132/18303	0.007998	0.037755	0.025821	Ccl3/Ccl5/Ccr1/Csf1/Csf2/Gata2/Gpr68/Ikzf1/Il23a/Jun/Kitlg/Lif/Lilrb4/Mafb/Rbp1/Sfrp1/Tnf	17
GO:0007622	rhythmic behavior	11/1242	71/18303	0.008017	0.037817	0.025863	Ada/Adora1/Adora2a/Csf2/Drd1/Egr1/Hcrtr2/Htr7/Pglyrp1/Ptger4/Star	11
GO:0045216	cell-cell junction organization	24/1242	209/18303	0.008028	0.037838	0.025878	Ank2/Cldn3/Ctnnd2/Epha4/F11r/Flcn/Fscn1/Fzd5/Gja5/Gjb2/Il1b/Nectin1/Pard6b/Pmp22/Ptpro/Inava/Ramp2/Shroom2/Snai1/Snai2/Svep1/Tnf/Trpv4/Whrn	24

ID	Description	GeneRatio	BgRatio	pvalue	p.adjust	qvalue	geneID	Count
GO:0007015	actin filament organization	43/1242	434/18303	0.008138	0.038272	0.026175	Alox15/Cdc42ep5/Cobl/Coro1a/Coro2b/Csf3/Cx3cl1/Espnl/F11r/Fscn1/Gas7/Gdpd2/Gmfg/Hip1r/LOC691418/Limch1/Micall1/Myo1f/Myo7a/Pdlim3/Phactr1/Plek/Pstpip2/Ptger4/Pycard/Cracd/Rgcc/Rhobtb1/Scin/Sema5a/Serpinf2/Shank1/Shroom2/Sirpa/Slit2/Sorbs2/Sptbn4/Tlr2/Tmod1/Tmod2/Trpv4/Twf2/Xirp1	43
GO:0043393	regulation of protein binding	26/1242	232/18303	0.00814	0.038272	0.026175	Atp2a3/Bdnf/Bmp2/Bmp4/Csf3/Cthrc1/Epha4/Ripor2/Flot1/Gpsm1/Grem2/Hfe/Hip1r/Hipk2/Mmp9/Ngf/Pcsk9/Pdgfb/Plk2/Plxnd1/Ramp2/Rgma/Ripk2/Spon1/Wnt5a/	26
GO:0060828	regulation of canonical Wnt signaling pathway	26/1242	232/18303	0.00814	0.038272	0.026175	Ankrd6/Bmp2/Cdh3/Col1a1/Cthrc1/Ctnnd2/Egr1/Fgf9/Fgfr2/Frzb/Fuz/Fzd4/Gli1/Lgr5/Nfkb1/Notum/Prickle1/Ptpro/Rbpj/Rspo1/Sema5a/Sfrp1/Snai2/Tmem88/Tnn/W	26
GO:0010837	regulation of keratinocyte proliferation	7/1242	35/18303	0.008175	0.038282	0.026182	Cask/Cdh3/Eppk1/Fgfr2/Has2/Snai2/Twist2	7
GO:0036120	cellular response to platelet-derived growth factor	7/1242	35/18303	0.008175	0.038282	0.026182	Ccl2/Errfi1/Gja5/Has2/Hyal1/Itgb3/Pcsk5	7
GO:0046697	decidualization	7/1242	35/18303	0.008175	0.038282	0.026182	Cyp27b1/Gjb2/Junb/Lif/Ppard/Ptgs2/Vim	7
GO:0071236	cellular response to antibiotic	7/1242	35/18303	0.008175	0.038282	0.026182	Abcb1a/Crip1/Egr1/Il1b/Mef2c/Ppp1r15a/Star	7
GO:0048675	axon extension	18/1242	143/18303	0.008177	0.038282	0.026182	Alcam/Bdnf/Cdh4/Cxcl12/Mgll/Ngf/Nrg1/Plxna3/Sema3f/Sema4a/Sema4b/Sema5a/Sema6a/Sema6d/Slit2/Slit3/Twf2/Wnt5a	18
GO:0001942	hair follicle development	14/1242	101/18303	0.008182	0.038282	0.026182	Cdh3/Edar/Fgfr2/Foxn1/Fuz/Hpse/Igfbp5/Inhba/Lgr5/Nsdhl/Rbpj/Snai1/Tnf/Wnt5a	14
GO:0060191	regulation of lipase activity	12/1242	81/18303	0.008239	0.038493	0.026326	Adcyap1r1/Agtr1a/Angptl4/Anxa8/Ccl5/Fgfr2/Flt1/Gna13/Hdac9/Htr2a/Pla2g5/Pla2r1	12
GO:0060135	maternal process involved in female pregnancy	13/1242	91/18303	0.008278	0.038493	0.026326	Akr1b1/Ccl2/Csmd1/Cyp27b1/Gjb2/Hsd17b7/Junb/Lgals9/Lif/Ppard/Prdm1/Ptgs2/Vim	13
GO:0050680	negative regulation of epithelial cell proliferation	19/1242	154/18303	0.008281	0.038493	0.026326	Bmp4/Cask/Ccl2/Dusp10/Eppk1/Fgfr2/Flt1/Gas1/Mef2c/Ppard/Rap1gap/Rgcc/Sfrp1/Snai2/Stat1/Thbs1/Tnf/Wfdc1/Wnt5a	19
GO:0006836	neurotransmitter transport	29/1242	267/18303	0.008294	0.038493	0.026326	Adora2a/Adra2a/Cacnb2/Cask/Cplx4/Cspg5/Doc2a/Drd1/Erc2/Flot1/Htr2a/Htr7/Itgb3/Kcnh1/Mef2c/Nat8l/Ngf/Nrn1/Nrxn3/P2ry2/Pclo/Ptger4/Rap1b/Slc17a6/Slc1a2/Slc6a12/Slc6a17/Sncaip/Sv2c	29
GO:0001780	neutrophil	6/1242	27/18303	0.008308	0.038493	0.026326	Cd44/Cfh/Hcar2/Pde4b/Pik3cb/Slc7a11	6
GO:0030194	positive regulation of blood coagulation	6/1242	27/18303	0.008308	0.038493	0.026326	F11r/Hpse/Plek/Selp/Serpinf2/Thbs1	6
GO:0030947	regulation of vascular endothelial growth factor receptor signaling pathway	6/1242	27/18303	0.008308	0.038493	0.026326	Cadm4/Emilin1/Fgf9/Vegfd/Flt1/Fzd4	6
GO:0033197	response to vitamin E	6/1242	27/18303	0.008308	0.038493	0.026326	Ada/Ccnd1/Col1a1/Col2a1/Hmgcs1/Mef2c	6
GO:0043032	positive regulation of macrophage	6/1242	27/18303	0.008308	0.038493	0.026326	Il33/Lbp/Lgals9/Thbs1/Tlr6/Wnt5a	6
GO:0045992	negative regulation of embryonic development	6/1242	27/18303	0.008308	0.038493	0.026326	Bmp4/Bmp7/Col5a1/Fuz/Snai1/Wnt5a	6
GO:0061311	cell surface receptor signaling pathway involved in heart development	6/1242	27/18303	0.008308	0.038493	0.026326	Bmp2/Bmp4/Hand2/Rbpj/Snai1/Wnt5a	6
GO:1900048	positive regulation of hemostasis	6/1242	27/18303	0.008308	0.038493	0.026326	F11r/Hpse/Plek/Selp/Serpinf2/Thbs1	6
GO:0043534	blood vessel endothelial cell migration	16/1242	122/18303	0.008353	0.038671	0.026448	Angpt4/Cd40/Efna1/Ets1/Gata2/Hdac9/Mef2c/Meox2/Pdgfb/Plk2/Ptgs2/Rgcc/Rhoj/Slit2/Thbs1/Tnf	16
GO:0030850	prostate gland development	10/1242	62/18303	0.008474	0.039106	0.026745	Bmp4/Bmp7/Cd44/Crip1/Cyp7b1/Fgfr2/Gli1/Plaur/Sfrp1/Wnt5a	10
GO:0048008	platelet-derived growth factor receptor signaling	10/1242	62/18303	0.008474	0.039106	0.026745	Cspg4/Csrnp1/F3/Hip1r/Itgb3/Pdgfb/Plat/Ptgir/Rgs14/Tiparp	10
GO:0060119	inner ear receptor cell development	10/1242	62/18303	0.008474	0.039106	0.026745	Adgrv1/Clic5/Cthrc1/Ripor2/Fat4/Fzd2/Myo7a/Slc4a7/Tshr/Whrn	10
GO:0090102	cochlea development	10/1242	62/18303	0.008474	0.039106	0.026745	Cthrc1/Epha4/Frzb/Fzd2/Gata2/Hey2/Kcnk2/Kcnk3/Tshr/Wnt5a	10
GO:0061387	regulation of extent of cell growth	17/1242	133/18303	0.008608	0.039692	0.027146	Bdnf/Cdh4/Cxcl12/Mgll/Ngf/Nrg1/Plxna3/Rgma/Sema3f/Sema4a/Sema4b/Sema5a/Sema6a/Sema6d/Slit2/Twf2/Wnt5a	17
GO:0016358	dendrite development	33/1242	315/18303	0.008686	0.040022	0.027372	Acsl4/Bdnf/Bmp5/Bmp7/Cask/Cobl/Ctnnd2/Dgkg/Dnm3/Dock10/Efna1/Elavl4/Epha4/Ephb2/Ephb3/Fat3/Igsf9/Kif1a/Lrp8/Map6/Matn2/Mef2c/Nrg1/Obsl1/Palm/Phactr1/Plk2/Scarf1/Sgk1/Shank1/Tet1/Tnik/Zdhhc15	33
GO:0033189	response to vitamin	8/1242	44/18303	0.008717	0.040063	0.0274	Abcb1a/Abcb1b/Bdnf/Cd44/Cyp26b1/Mmp9/Ppard/Rb	8
GO:0046636	negative regulation of alpha-beta T cell activation	8/1242	44/18303	0.008717	0.040063	0.0274	Adora2a/Cd274/Cd44/Hfe/Lgals9/Tnfsf18/Vsir/Xcl1	8

ID	Description	GeneRatio	BgRatio	pvalue	p.adjust	qvalue	geneID	Count
GO:0060122	inner ear receptor cell stereocilium organization	8/1242	44/18303	0.008717	0.040063	0.0274	Adgrv1/Clic5/Cthrc1/Ripor2/Fat4/Myo7a/Tshr/Whrn	8
GO:0032648	regulation of interferon-beta production	9/1242	53/18303	0.008751	0.040063	0.0274	Flot1/Irf1/Pycard/Rel/Relb/Riok3/Sirpa/Tlr2/Tlr3	9
GO:0034331	cell junction maintenance	9/1242	53/18303	0.008751	0.040063	0.0274	Bsn/Cadm1/Cldn3/Erc2/Mpp2/Pclo/Inava/Shroom2/Whrn	9
GO:0050850	positive regulation of calcium-mediated signaling	9/1242	53/18303	0.008751	0.040063	0.0274	Ada/Ccl3/Itgal/Ncam1/Nrg1/P2ry6/RGD1564899/Syk/Tnf	9
GO:0060428	lung epithelium development	9/1242	53/18303	0.008751	0.040063	0.0274	Bmp4/Errfi1/Fgfr2/Gata6/Hmga2/Klf2/Rbpj/Thra/Thrb	9
GO:1900015	regulation of cytokine production involved in	9/1242	53/18303	0.008751	0.040063	0.0274	Abcd1/Chid1/Gbp5/Nod2/Nos2/Pycard/Sirpa/Tlr6/Tnf	9
GO:0010212	response to ionizing radiation	22/1242	188/18303	0.008762	0.040083	0.027414	Abcb1b/Blm/Ccl2/Ccnd1/Col2a1/Cxcl1/Cxcl10/Cxcl2/Egr1/Ercc1/Hmga2/Il1a/Il1b/Nabp1/Nampt/Net1/Sfrp1/Snai2/Socs3/Star/Thbd/Vcam1	22
GO:0035051	cardiocyte differentiation	21/1242	177/18303	0.008841	0.04041	0.027637	Bmp2/Bmp4/Bmp7/Bves/Col14a1/Dll1/Fdps/Gata6/Hand2/Hey2/Mef2c/Nrg1/Pi16/Prickle1/Rbpj/Sgcd/Sorbs2/Tbx3/Tenm4/Vcam1/Xirp1	21
GO:0001894	tissue homeostasis	30/1242	280/18303	0.008848	0.04041	0.027637	Abcb1a/Adgrv1/Akr1b1/Bdkrb2/Cdh3/Cngb1/Col14a1/Col2a1/Coro1a/Csf1/Flt3lg/Foxc1/Gata2/Itgb3/Lca5/Lyz2/Nod2/Ptger4/Ptgs2/Pth1r/Cracd/Inava/Rab3d/Scx/Slc1a1/Syk/Tnfrsf11b/Trpv4/Tub/Whrn	30
GO:0035270	endocrine system development	20/1242	166/18303	0.008876	0.040475	0.027682	Bmp2/Bmp4/Bmp5/Cyp1b1/Dll1/Ets1/Gata2/Gata6/Gli1/Hmga2/Hnf1b/Pax1/Pax8/Pcsk1/Rap1gap/Rbpj/Rfx3/Tg/Thra/Wnt5a	20
GO:0051224	negative regulation of protein transport	20/1242	166/18303	0.008876	0.040475	0.027682	Adra2a/Cx3cl1/Cyp51/Derl3/Frmd4a/Hadh/Hdac9/Hmgcr/Il1b/Il33/Insig1/Ptger4/RGD1311251/Rab11fip1/Rgcc/Rsad2/Sfrp1/Siglec10/Vsnl1/Yod1	20
GO:0002292	T cell differentiation involved in immune response	11/1242	72/18303	0.008899	0.040484	0.027687	Bcl3/Clec4e/Il23a/Lgals9/Nlrp3/Ptger4/Myb/Relb/Ripk2/Sema4a/Tnfsf18	11
GO:0009064	glutamine family amino acid metabolic process	11/1242	72/18303	0.008899	0.040484	0.027687	Aadat/Adhfe1/Ass1/Ddah2/Fah/Gclc/Gfpt2/Ggt1/Nos2/Pycr1/Slc7a11	11
GO:0050771	negative regulation of axonogenesis	11/1242	72/18303	0.008899	0.040484	0.027687	Ephb2/Plxna3/Rgma/Sema3f/Sema4a/Sema4b/Sema5a/Sema6a/Sema6d/Slit2/Wnt5a	11
GO:0034109	homotypic cell-cell adhesion	13/1242	92/18303	0.009058	0.041145	0.02814	Ank3/C1qtnf1/Ccl5/Cd99l2/Cfh/F11r/Itgb3/Ncam1/Plaur/Plek/Plpp3/Slc7a11/Syk	13
GO:0061097	regulation of protein tyrosine kinase	13/1242	92/18303	0.009058	0.041145	0.02814	Adora1/Adra2a/Areg/Ccl5/Efna1/Epha4/Ereg/Errfi1/Itgb3/Lrp8/Nrg1/Pdgfb/Trpv4	13
GO:0006534	cysteine metabolic process	4/1242	13/18303	0.009192	0.041232	0.028199	Cth/Gclc/Ggt1/Slc7a11	4
GO:0033083	regulation of immature T cell	4/1242	13/18303	0.009192	0.041232	0.028199	Bmp4/Il1a/Il1b/Ripk2	4
GO:0033089	positive regulation of T cell differentiation in thymus	4/1242	13/18303	0.009192	0.041232	0.028199	Ada/Il1a/Il1b/Rasgrp1	4
GO:0034350	regulation of glial cell apoptotic process	4/1242	13/18303	0.009192	0.041232	0.028199	Akap12/Ccl2/Dll1/Pmp22	4
GO:0042635	positive regulation of hair cycle	4/1242	13/18303	0.009192	0.041232	0.028199	Foxn1/Hpse/Tnf/Wnt5a	4
GO:0045741	positive regulation of epidermal growth factor-activated receptor activity	4/1242	13/18303	0.009192	0.041232	0.028199	Adora1/Adra2a/Areg/Ereg	4
GO:0048711	positive regulation of astrocyte differentiation	4/1242	13/18303	0.009192	0.041232	0.028199	Bmp2/Clcf1/Il1b/Lif	4
GO:0051775	response to redox state	4/1242	13/18303	0.009192	0.041232	0.028199	Arhgdib/Nos2/Ryr2/Slc7a11	4
GO:0060272	embryonic skeletal joint morphogenesis	4/1242	13/18303	0.009192	0.041232	0.028199	Bmp4/Bmp7/Col2a1/Hyal1	4
GO:0060340	positive regulation of type I interferon-mediated signaling pathway	4/1242	13/18303	0.009192	0.041232	0.028199	Mmp12/Nlrc5/Trim6/Wnt5a	4
GO:0060601	lateral sprouting from an epithelium	4/1242	13/18303	0.009192	0.041232	0.028199	Bmp4/Bmp7/Fgfr2/Wnt5a	4
GO:0060670	branching involved in labyrinthine layer morphogenesis	4/1242	13/18303	0.009192	0.041232	0.028199	Fgfr2/Fzd5/Socs3/St14	4
GO:0070255	regulation of mucus secretion	4/1242	13/18303	0.009192	0.041232	0.028199	Ada/Adora1/P2ry2/Ptger4	4
GO:0072216	positive regulation of metanephros development	4/1242	13/18303	0.009192	0.041232	0.028199	Egr1/Lif/Pax8/Pdgfb	4

ID	Description	GeneRatio	BgRatio	pvalue	p.adjust	qvalue	geneID	Count
GO:0090193	positive regulation of glomerulus development	4/1242	13/18303	0.009192	0.041232	0.028199	Cflar/Egr1/Itgb3/Pdgfb	4
GO:1902285	semaphorin-plexin signaling pathway involved in neuron projection guidance	4/1242	13/18303	0.009192	0.041232	0.028199	Plxna2/Plxna3/Plxnd1/Sema3f	4
GO:0043010	camera-type eye development	40/1242	401/18303	0.009369	0.041761	0.028561	Aqp1/Bfsp1/Bmp4/Bmp7/Cfh/Col4a1/Col8a2/Cyp1b1/Dll1/Ephb2/Fat3/Fgfr2/Flt1/Foxc1/Fzd4/Fzd5/Gas1/Hcn1/Hipk2/Ikzf1/Inhba/Jun/Angptl7/Lif/Nectin1/Obsl1/Pdgfb/Rhoj/Rorb/Slc17a6/Slc25a25/Slc4a7/Slc7a11/Tgif1/Thrb/Tmod1/Tub/Twist2/Vim/Zeb2	40
GO:0002577	regulation of antigen processing and presentation	5/1242	20/18303	0.00938	0.041761	0.028561	Cd68/Hfe/Nod2/Pycard/Thbs1	5
GO:0007252	I-kappaB phosphorylation	5/1242	20/18303	0.00938	0.041761	0.028561	Cx3cl1/Sirpa/Tlr2/Tlr3/Tnf	5
GO:0032695	negative regulation of interleukin-12 production	5/1242	20/18303	0.00938	0.041761	0.028561	Cmklr1/Nfkb1/Nod2/Thbs1/Tlr2	5
GO:0043116	negative regulation of vascular permeability	5/1242	20/18303	0.00938	0.041761	0.028561	Adora2a/Akap12/Pde2a/Ramp2/Slit2	5
GO:0050965	detection of temperature stimulus involved in sensory perception of pain	5/1242	20/18303	0.00938	0.041761	0.028561	Adora1/Cxcl12/Cxcr4/Htr2a/Lxn	5
GO:0097094	craniofacial suture morphogenesis	5/1242	20/18303	0.00938	0.041761	0.028561	Bmp4/Fgfr2/Frem1/Insig1/Mmp16	5
GO:0050803	regulation of synapse structure or activity	31/1242	293/18303	0.009382	0.041761	0.028561	Abhd17c/Bdnf/Cask/Clstn2/Dnm3/Efna1/Epha4/Ephb2/Ephb3/Flrt3/Igsf9/Kif1a/Lrfn1/Lrp8/Mef2c/Nectin1/Nptxr/Ptpro/Ptprt/Rapsn/Sema3f/Sema4a/Slc17a6/Slc7a11/Sorbs2/Sybu/Thbs2/Tlr2/Tnf/Wnt5a/Zdhhc15	31
GO:0001776	leukocyte homeostasis	15/1242	113/18303	0.009382	0.041761	0.028561	Ada/Cd44/Cfh/Coro1a/Dock10/Foxn1/Kitlg/Lgals9/Mef2c/Hcar2/Pde4b/Pik3cb/Sh2b2/Slc7a11/Tnfaip3	15
GO:0045446	endothelial cell differentiation	15/1242	113/18303	0.009382	0.041761	0.028561	Abcb1b/Bmp4/Cldn3/Cxcr4/Dll1/F11r/Hey2/Hoxb5/Il1b/Nrg1/Pde2a/Rap1b/Rbpj/Tmem100/Tnf	15
GO:0071383	cellular response to steroid hormone stimulus	28/1242	258/18303	0.009465	0.042097	0.028791	Abcb1a/Agtr1a/Aqp1/Ass1/Bdnf/Bmp4/Bmp7/Cacna1g/Ccl2/Cflar/Crebrf/Cyp1b1/Cyp7b1/Egr1/Errfi1/Gjb2/Gstp1/Hmga2/Acod1/Lbh/Plat/Serpina3n/Sfrp1/Sgk1/Star/Trim68/Ucn2/Ugt1a6	28
GO:0002294	CD4-positive, alpha-beta T cell differentiation involved in immune	10/1242	63/18303	0.009477	0.042116	0.028804	Bcl3/Il23a/Lgals9/Nlrp3/Ptger4/Myb/Relb/Ripk2/Sema4a/Tnfsf18	10
GO:0032369	negative regulation of lipid transport	7/1242	36/18303	0.009568	0.042421	0.029013	Acsl4/Itgb3/Nrg1/Pcsk9/Pla2r1/Thbs1/Tnf	7
GO:0036119	response to platelet-derived growth factor	7/1242	36/18303	0.009568	0.042421	0.029013	Ccl2/Errfi1/Gja5/Has2/Hyal1/Itgb3/Pcsk5	7
GO:2000516	positive regulation of CD4-positive, alpha-beta T cell activation	7/1242	36/18303	0.009568	0.042421	0.029013	Cd83/Il23a/Lgals9/Nlrp3/Myb/Ripk2/Xcl1	7
GO:0022404	molting cycle process	14/1242	103/18303	0.009686	0.042848	0.029305	Cdh3/Edar/Fgfr2/Foxn1/Fuz/Hpse/Igfbp5/Inhba/Lgr5/Nsdhl/Rbpj/Snai1/Tnf/Wnt5a	14
GO:0022405	hair cycle process	14/1242	103/18303	0.009686	0.042848	0.029305	Cdh3/Edar/Fgfr2/Foxn1/Fuz/Hpse/Igfbp5/Inhba/Lgr5/Nsdhl/Rbpj/Snai1/Tnf/Wnt5a	14
GO:0045833	negative regulation of lipid metabolic	14/1242	103/18303	0.009686	0.042848	0.029305	Adora1/Adra2a/Bmp2/Bmp5/Cyp27b1/Il1b/Insig1/Nfkb1/Hcar2/Pdgfb/Pik3ip1/Snai1/Snai2/Tnf	14
GO:0032570	response to progesterone	11/1242	73/18303	0.009853	0.043529	0.02977	Ccl2/Cyp1b1/Fosl1/Gjb2/Acod1/Junb/Nrg1/Ptger4/Ramp2/Socs3/Tlr2	11
GO:0006692	prostanoid metabolic process	9/1242	54/18303	0.009885	0.043529	0.02977	Bdkrb1/Fabp5/Gsta2/Gstp1/Hpgd/Il1b/Ptges/Ptgr1/Ptgs2	9
GO:0006693	prostaglandin metabolic process	9/1242	54/18303	0.009885	0.043529	0.02977	Bdkrb1/Fabp5/Gsta2/Gstp1/Hpgd/Il1b/Ptges/Ptgr1/Ptgs2	9
GO:0030834	regulation of actin filament depolymerization	9/1242	54/18303	0.009885	0.043529	0.02977	Plek/Cracd/Scin/Sema5a/Shroom2/Sptbn4/Tmod1/Tmod2/Twf2	9
GO:0032608	interferon-beta production	9/1242	54/18303	0.009885	0.043529	0.02977	Flot1/Irf1/Pycard/Rel/Relb/Riok3/Sirpa/Tlr2/Tlr3	9
GO:0072132	mesenchyme morphogenesis	9/1242	54/18303	0.009885	0.043529	0.02977	Bmp2/Bmp5/Bmp7/Foxc1/Rbpj/Snai1/Snai2/Tmem100/Wnt5a	9
GO:0030177	positive regulation of Wnt signaling	17/1242	135/18303	0.009939	0.043708	0.029893	Ankrd6/Bmp2/Cdh3/Col1a1/Fgf9/Fgfr2/Lgr5/Nfkb1/Rbpj/Rspo1/Rspo4/Sema5a/Sfrp1/Tlr2/Tnfaip3/Wnt5a/Zeb	17
GO:0032088	negative regulation of NF-kappaB transcription factor activity	12/1242	83/18303	0.009969	0.043708	0.029893	Cmklr1/Cyp1b1/Irak2/Acod1/Nfkbia/Nfkbib/Nlrc5/Nlrp3/Nod2/Peli1/Pycard/Tnfaip3	12
GO:0002825	regulation of T-helper 1 type immune response	6/1242	28/18303	0.009979	0.043708	0.029893	Il1b/Il1r1/Il23a/Il33/Ripk2/Xcl1	6

ID	Description	GeneRatio	BgRatio	pvalue	p.adjust	qvalue	geneID	Count
GO:0032816	positive regulation of natural killer cell activation	6/1242	28/18303	0.009979	0.043708	0.029893	Flt3lg/Il15/Il15ra/Il23a/Rasgrp1/Tox	6
GO:0032928	regulation of superoxide anion generation	6/1242	28/18303	0.009979	0.043708	0.029893	Agtr1a/Cxcl1/Gstp1/Hvcn1/Olr1/Syk	6
GO:0048521	negative regulation of behavior	6/1242	28/18303	0.009979	0.043708	0.029893	Ada/Adora1/Arrdc3/Drd1/Htr7/Ptger4	6
GO:0048714	positive regulation of oligodendrocyte differentiation	6/1242	28/18303	0.009979	0.043708	0.029893	Aspa/Cxcr4/Enpp2/Tenm4/Tlr2/Tnfrsf1b	6
GO:0042311	vasodilation	8/1242	45/18303	0.009987	0.043709	0.029894	Adora1/Adora2a/Bdkrb2/Drd1/Ednrb/Gch1/Tnf/Trpv4	8
GO:0007156	homophilic cell adhesion via plasma membrane adhesion molecules	18/1242	146/18303	0.010062	0.043969	0.030071	Cadm1/Cdh3/Cdh4/Clstn2/Fat3/Fat4/Igsf9/Igsf9b/Pcdhb6l/Nectin1/Obsl1/Pvr/Pcdh18/Pcdhb14/Pcdhb3/Pcdhb5/Pik3cb/Ptprt	18
GO:0048593	camera-type eye morphogenesis	18/1242	146/18303	0.010062	0.043969	0.030071	Aqp1/Bmp4/Bmp7/Cfh/Col8a2/Dll1/Ephb2/Fat3/Flt1/Fzd5/Hcn1/Hipk2/Ikzf1/Nectin1/Obsl1/Rorb/Slc4a7/Thrb	18
GO:0032412	regulation of ion transmembrane transporter activity	29/1242	271/18303	0.010093	0.044075	0.030144	Abcb1a/Adra2a/Ank2/Ank3/Bmp4/Cacnb2/Casq1/Ccl2/Cnih2/Ephb2/Fhl1/Grin2a/Hcn1/Lrrc38/Mef2c/Mmp9/P2ry6/Pcsk9/Pde4b/Ppargc1a/Ppif/Prrt1/Rgs9/Ryr2/Scn2b/Shank1/Stom/Trpv4/Wnk4	29
GO:0009410	response to xenobiotic stimulus	15/1242	114/18303	0.010147	0.044275	0.030281	Acsl1/Aox1/As3mt/Cyp1b1/Cyp26b1/Cyp2j4/Gclc/Gsta2/Gsta5/Gsto2/Gstp1/LOC108348061/Nqo1/Star/Ugt1a	15
GO:0044242	cellular lipid catabolic process	25/1242	225/18303	0.010348	0.045118	0.030857	Abcd1/Acat2/Acat2l1/Cpt1a/Cpt1b/Cyp1b1/Cyp26b1/Ech1/Echdc2/Enpp2/Fabp3/Hadh/Hpgd/Ldlr/Lipg/Liph/Mgll/Neu3/Phyh/Pla2g4e/Pla2g5/Pla2g7/Plcb1/Pnpla3/	25
GO:0031341	regulation of cell killing	16/1242	125/18303	0.010465	0.045595	0.031183	Cadm1/Ccl2/Cfh/Cxcl1/Hspa1b/Il18rap/Il23a/Lgals9/Mr1/Nos2/Pvr/Rasgrp1/Serpinb3a/Serpinb9/Syk/Xcl1	16
GO:0050772	positive regulation of axonogenesis	14/1242	104/18303	0.010515	0.045778	0.031308	Bdnf/Cdh4/Cxcl12/Map6/Metrn/Ngf/Nrg1/Plxna2/Plxna3/Plxnd1/Sema5a/Tubb2b/Twf2/Zeb2	14
GO:0002293	alpha-beta T cell differentiation involved in immune	10/1242	64/18303	0.010568	0.045868	0.03137	Bcl3/Il23a/Lgals9/Nlrp3/Ptger4/Myb/Relb/Ripk2/Sema4a/Tnfsf18	10
GO:0008347	glial cell migration	10/1242	64/18303	0.010568	0.045868	0.03137	Ccl2/Ccl3/Csf1/Cspg4/Cx3cl1/Epha4/Lrp8/Matn2/Pmp22/Vim	10
GO:0050982	detection of mechanical stimulus	10/1242	64/18303	0.010568	0.045868	0.03137	Adgrv1/Col11a1/Cxcl12/Cxcr4/Htr2a/Htr7/Itga2/Piezo2/Tnf/Whrn	10
GO:0060443	mammary gland morphogenesis	10/1242	64/18303	0.010568	0.045868	0.03137	Areg/Bmp4/Csf1/Csmd1/Elf3/Fgfr2/Igfbp5/Slit2/Tbx3/Wnt5a	10
GO:0006814	sodium ion transport	26/1242	237/18303	0.010598	0.045966	0.031437	Ank3/Asic4/Cacna1g/Cxcl1/Hcn1/Nalcn/Nkain4/Pcsk9/Scn2b/Sgk1/Slc17a1/Slc17a3/Slc17a6/Slc20a1/Slc22a4/Slc24a3/Slc24a4/Slc4a11/Slc4a7/Slc6a12/Slc6a17/Slc9a9/Sptbn4/Stom/Trpv4/Wnk4	26
GO:0007586	digestion	17/1242	136/18303	0.010664	0.046214	0.031607	Abcb1a/Acat2/Aqp1/Ctrb1/F11r/Hip1r/Kcnn4/Ldlr/Mmp13/Mogat2/Nod2/Npr3/Ptger1/Cracd/Inava/Ucn2/Wnk	17
GO:0001101	response to acid chemical	30/1242	284/18303	0.010697	0.046325	0.031682	Abcb1a/Akr1b1/Ass1/Bdnf/Ccl2/Ccl5/Col1a1/Col3a1/Col4a1/Cpeb4/Drd1/Gclc/Glra1/Glrb/Grin2a/Gstp1/Hcn1/Ipo5/Klf2/Mmp3/Naip6/Nqo1/Pnpla3/Ppargc1a/Ptger4/Rragd/Sesn3/Slc1a2/Timp3/Tnf	30
GO:0002832	negative regulation of response to biotic stimulus	13/1242	94/18303	0.010792	0.046665	0.031915	Ccr1/Dusp10/Acod1/Lgals9/Micb/Mmp12/Nlrc5/Prdm1/Riok3/Serpinb3a/Serpinb9/Serping1/Tnfaip3	13
GO:0021536	diencephalon development	13/1242	94/18303	0.010792	0.046665	0.031915	Bmp2/Bmp4/Ets1/Gata2/Gbx2/Gli1/Hmga2/Ncam1/Pcsk1/Rbpj/Sema5a/Wnt5a/Zeb2	13
GO:0051781	positive regulation of cell division	11/1242	74/18303	0.010885	0.047031	0.032165	Ereg/Fgf9/Fgfr2/Vegfd/Il1a/Il1b/Lbh/Pdgfb/Pgf/Rxfp3/Thbs4	11
GO:0050805	negative regulation of synaptic transmission	12/1242	84/18303	0.010934	0.047174	0.032263	Adcy8/Adora1/Bdnf/Drd1/Htr2a/Il1b/Mgll/Plk2/Ptgs2/Rap1b/Sorcs2/Tmem25	12
GO:0072078	nephron tubule morphogenesis	12/1242	84/18303	0.010934	0.047174	0.032263	Agtr1a/Bmp2/Bmp4/Cd44/Fat4/Hnf1b/Irx3/Npnt/Pax8/Pgf/Wnk4/Wnt2b	12
GO:0010042	response to manganese ion	7/1242	37/18303	0.011126	0.047793	0.032686	A3galt2/Atp7b/Casp12/Ern1/Fibin/Grin2a/Ptgs2	7
GO:0042537	benzene-containing compound metabolic process	7/1242	37/18303	0.011126	0.047793	0.032686	Aadat/Cyp1b1/Cyp4b1/Fah/Star/Txnrd1/Ugt1a6	7
GO:1905209	positive regulation of cardiocyte differentiation	7/1242	37/18303	0.011126	0.047793	0.032686	Bmp4/Bmp7/Fdps/Gata6/Mef2c/Nrg1/Rbpj	7
GO:0006023	aminoglycan biosynthetic process	9/1242	55/18303	0.011127	0.047793	0.032686	B3gnt9/Chst14/Has2/Hyal1/Il1b/Nfkb1/Pdgfb/Ptger4/Xylt1	9
GO:0032892	positive regulation of organic acid transport	9/1242	55/18303	0.011127	0.047793	0.032686	Adora2a/Erfe/Fabp3/Il1a/Il1b/P2ry2/Pla2r1/Ptges/Trpv4	9
GO:0045604	regulation of epidermal cell differentiation	9/1242	55/18303	0.011127	0.047793	0.032686	Bmp4/Cyp27b1/Dll1/Errfi1/Foxc1/Macroh2a2/Hey2/Maff/Mycl	9
GO:0072329	monocarboxylic acid catabolic process	16/1242	126/18303	0.011256	0.048275	0.033016	Abcd1/Acat2/Acat2l1/Cpt1a/Cpt1b/Cyp26b1/Ech1/Echdc2/Fabp3/Fah/Hadh/Hoga1/Hpgd/Idnk/Phyh/Ppard	16

ID	Description	GeneRatio	BgRatio	pvalue	p.adjust	qvalue	geneID	Count
GO:0090090	negative regulation of canonical Wnt signaling pathway	16/1242	126/18303	0.011256	0.048275	0.033016	Ankrd6/Bmp2/Cthrc1/Egr1/Frzb/Fuz/Fzd4/Gli1/Notum/Prickle1/Ptpro/Sfrp1/Snai2/Tmem88/Tnn/Wnt5a	16
GO:0002753	cytoplasmic pattern recognition receptor signaling pathway	8/1242	46/18303	0.011389	0.0487	0.033307	Hspa1b/Nfkbia/Nod2/Inava/Riok3/Ripk2/Tifa/Tnfaip3	8
GO:0032881	regulation of polysaccharide metabolic process	8/1242	46/18303	0.011389	0.0487	0.033307	Has2/Nfkb1/Pdgfb/Ppp1r3b/Ppp1r3c/Ppp1r3e/Ppp1r3g/Ptger4	8
GO:0035456	response to interferon-beta	8/1242	46/18303	0.011389	0.0487	0.033307	Gbp4/Hcn1/Ifit3/Irf1/Acod1/Stat1/Tlr3/Trim6	8
GO:0043330	response to exogenous dsRNA	8/1242	46/18303	0.011389	0.0487	0.033307	Ciita/Flot1/Irf1/Nfkbia/Nod2/Ripk2/Stat1/Tlr3	8
GO:0051896	regulation of protein kinase B signaling	20/1242	170/18303	0.011403	0.048723	0.033323	C1qtnf1/C1qtnf3/Ccl3/Csf3/Cx3cl1/F3/Fam110c/Flcn/Gdf15/Hpse/Igfbp5/Mmp3/Mst1r/Nrg1/Pik3r5/Cavin3/Sema5a/Sesn3/Thbs1/Tnf	20
GO:0010811	positive regulation of cell-substrate	17/1242	137/18303	0.011429	0.048799	0.033374	Alox15/Ccdc80/Csf1/Cspg5/Edil3/Emilin1/Enpp2/Fbln1/Has2/Itga5/Itgb3/Ndnf/Net1/Npnt/Pcsk5/Smoc1/Thbs	17
GO:1901214	regulation of neuron death	39/1242	394/18303	0.011487	0.04901	0.033519	Adora1/Adora2a/Bdnf/Btg2/Ccl2/Ccl3/Ccl5/Cd200r1/Clcf1/Coro1a/Cpeb4/Csf1/Csf3/Cx3cl1/Egln3/Egr1/Gclc/Gpr75/Hipk2/Il1b/Jun/LOC100911625/Mef2c/Ndnf/Ngf/Nqo1/Pcp4/Pcsk9/Ppargc1a/Ptpn5/Myb/Rapsn/Rel/Slc7a11/Star/Tlr6/Tnf/Tnfrsf1b/Wnt5a	39
GO:0002827	positive regulation of T-helper 1 type immune response	5/1242	21/18303	0.011636	0.049391	0.033779	Il1b/Il1r1/Il23a/Ripk2/Xcl1	5
GO:0003180	aortic valve morphogenesis	5/1242	21/18303	0.011636	0.049391	0.033779	Bmp4/Efna1/Emilin1/Slit2/Slit3	5
GO:0010743	regulation of macrophage derived foam cell differentiation	5/1242	21/18303	0.011636	0.049391	0.033779	Csf1/Csf2/Itgb3/Nfkbia/Pla2g5	5
GO:0032693	negative regulation of interleukin-10 production	5/1242	21/18303	0.011636	0.049391	0.033779	Cd274/Dll1/Il23a/Trib2/Vsir	5
GO:0072537	fibroblast activation	5/1242	21/18303	0.011636	0.049391	0.033779	Akap12/Gclc/Pdgfb/Myb/Rgcc	5
GO:0090050	positive regulation of cell migration involved in sprouting angiogenesis	5/1242	21/18303	0.011636	0.049391	0.033779	Gata2/Hdac9/Plk2/Ptgs2/Rhoj	5
GO:1904754	positive regulation of vascular associated smooth muscle cell migration	5/1242	21/18303	0.011636	0.049391	0.033779	Dock4/Fgf9/Igfbp5/Pcsk5/Pdgfb	5
GO:0022600	digestive system process	15/1242	116/18303	0.011823	0.050145	0.034295	Abcb1a/Acat2/Aqp1/F11r/Hip1r/Kcnn4/Ldlr/Mmp13/Mogat2/Nod2/Npr3/Ptger1/Cracd/Inava/Wnk4	15
GO:0003309	type B pancreatic cell differentiation	6/1242	29/18303	0.011876	0.05026	0.034374	Bmp4/Bmp5/Dll1/Gata6/Rfx3/Wnt5a	6
GO:0043372	positive regulation of CD4-positive, alpha-beta T cell differentiation	6/1242	29/18303	0.011876	0.05026	0.034374	Cd83/Il23a/Lgals9/Nlrp3/Myb/Ripk2	6
GO:0060571	morphogenesis of an epithelial fold	6/1242	29/18303	0.011876	0.05026	0.034374	Bmp4/Bmp5/Bmp7/Fgfr2/Wnt2b/Wnt5a	6
GO:0019915	lipid storage	12/1242	85/18303	0.011969	0.050617	0.034618	B4galnt1/Cpt1a/Hilpda/Il1b/Itgb3/Nfkbia/Plin2/Ppard/Sqle/Stard4/Tnf/Ttc39b	12
GO:0035094	response to nicotine	11/1242	75/18303	0.011997	0.050695	0.034672	Casp12/Col2a1/Drd1/Mmp9/Nfkb1/Ngf/Penk/Slc7a11/Star/Tnf/Vcam1	11
GO:0090257	regulation of muscle system process	28/1242	263/18303	0.012085	0.050832	0.034765	Ada/Adora1/Adra2a/Ank2/Bdkrb1/Calcrl/Casq1/Col14a1/Dock4/Errfi1/Fdps/Flt1/Gja5/Hand2/Igfbp5/Itga2/Mef2c/Npnt/Pde4b/Pi16/Ppargc1a/Ptger4/Ptgs2/Ryr2/Sgca/Tnfrsf1b/Tnnt3/Trpv4	28
GO:1903169	regulation of calcium ion transmembrane transport	20/1242	171/18303	0.012117	0.050832	0.034765	Adra2a/Agtr1a/Ank2/Bdkrb1/Bmp4/Cacnb2/Casq1/Cemip/Coro1a/Cx3cl1/Cxcl10/Cxcl11/Drd1/Itgb3/Ngf/P2ry6/Pde4b/Rgs9/Ryr2/Xcl1	20
GO:0001781	neutrophil apoptotic process	4/1242	14/18303	0.012188	0.050832	0.034765	Cd44/Hcar2/Pik3cb/Slc7a11	4
GO:0010819	regulation of T cell chemotaxis	4/1242	14/18303	0.012188	0.050832	0.034765	Ccl5/Cxcl10/Wnt5a/Xcl1	4
GO:0019370	leukotriene biosynthetic process	4/1242	14/18303	0.012188	0.050832	0.034765	Ggt1/Mgst3/Pla2g5/Syk	4
GO:0031652	positive regulation of heat generation	4/1242	14/18303	0.012188	0.050832	0.034765	Ccl5/Il1b/Ptgs2/Tnf	4
GO:0032604	granulocyte macrophage colony-stimulating factor production	4/1242	14/18303	0.012188	0.050832	0.034765	Il1b/Il23a/Rasgrp1/Syk	4

ID	Description	GeneRatio	BgRatio	pvalue	p.adjust	qvalue	geneID	Count
GO:0032645	regulation of granulocyte macrophage colony-stimulating factor production	4/1242	14/18303	0.012188	0.050832	0.034765	Il1b/Il23a/Rasgrp1/Syk	4
GO:0032736	positive regulation of interleukin-13 production	4/1242	14/18303	0.012188	0.050832	0.034765	Il33/Lgals9/Nlrp3/Tslp	4
GO:0033079	immature T cell proliferation	4/1242	14/18303	0.012188	0.050832	0.034765	Bmp4/Il1a/Il1b/Ripk2	4
GO:0045906	negative regulation of vasoconstriction	4/1242	14/18303	0.012188	0.050832	0.034765	Cx3cl1/Dock4/Dusp5/Hspa1b	4
GO:0070307	lens fiber cell development	4/1242	14/18303	0.012188	0.050832	0.034765	Bfsp1/Fgfr2/Tmod1/Vim	4
GO:0072160	nephron tubule epithelial cell differentiation	4/1242	14/18303	0.012188	0.050832	0.034765	Fat4/Lif/Mef2c/Pax8	4
GO:0086014	atrial cardiac muscle cell action potential	4/1242	14/18303	0.012188	0.050832	0.034765	Ank2/Cacnb2/Gja5/Ryr2	4
GO:0086026	atrial cardiac muscle cell to AV node cell signaling	4/1242	14/18303	0.012188	0.050832	0.034765	Ank2/Cacnb2/Gja5/Ryr2	4
GO:0086066	atrial cardiac muscle cell to AV node cell communication	4/1242	14/18303	0.012188	0.050832	0.034765	Ank2/Cacnb2/Gja5/Ryr2	4
GO:2000052	positive regulation of non-canonical Wnt signaling pathway	4/1242	14/18303	0.012188	0.050832	0.034765	Ankrd6/Rspo1/Sfrp1/Wnt5a	4
GO:2001279	regulation of unsaturated fatty acid biosynthetic process	4/1242	14/18303	0.012188	0.050832	0.034765	Abcd1/Fabp5/Il1b/Ptgs2	4
GO:0071695	anatomical structure maturation	29/1242	275/18303	0.0122	0.050848	0.034776	Akr1b1/Ank3/Bfsp1/Bmp2/Cabyr/Cd44/Cdh3/Cspg4/Ednrb/Ereg/Fam20c/Fat4/Fzd5/Gata2/Il15/Klf2/Ngf/Ntn4/Pdgfb/Pmp22/Prkar2a/Pth1r/Rbpj/Rfx3/Scarf1/Sptbn4/Tcp11/Wnt5a/Xylt1	29
GO:0007043	cell-cell junction assembly	18/1242	149/18303	0.012277	0.051132	0.03497	Ank2/Cldn3/Ctnnd2/F11r/Flcn/Fscn1/Fzd5/Gja5/Gjb2/Il1b/Pard6b/Pmp22/Ptpro/Ramp2/Snai1/Snai2/Tnf/Trpv4	18
GO:0002027	regulation of heart rate	14/1242	106/18303	0.012339	0.051279	0.03507	Ada/Ank2/Bves/Cacna1g/Cacnb2/Gch1/Gja5/Hey2/Kcnh2/Ryr2/Scn2b/Slc1a1/Sptbn4/Tnf	14
GO:0045778	positive regulation of ossification	14/1242	106/18303	0.012339	0.051279	0.03507	Adgrv1/Bmp2/Bmp4/Bmp7/Cebpd/Cthrc1/Fam20c/Gdpd2/Isg15/Mef2c/Npnt/Ptger4/Thrb/Wnt5a	14
GO:1901264	carbohydrate derivative transport	14/1242	106/18303	0.012339	0.051279	0.03507	Abcb1a/Abcd1/Ada/Adora1/Lbp/P2ry2/Pltp/Ptger4/Slc17a1/Slc17a3/Slc25a25/Slc25a43/Slc25a48/Trpv4	14
GO:0071805	potassium ion transmembrane transport	24/1242	217/18303	0.012467	0.051687	0.03535	Abcc9/Ank2/Ank3/Aqp1/Fhl1/Hcn1/Kcna3/Kcnh1/Kcnh2/Kcnip4/Kcnk2/Kcnk3/Kcnk5/Kcnn4/Kcnq5/Kcnt2/Lrrc38/Nalcn/Rgs7/Slc24a3/Slc24a4/Slc9a9/Trpv4/Wnk4	24
GO:0002067	glandular epithelial cell differentiation	9/1242	56/18303	0.012482	0.051687	0.03535	Bmp2/Bmp4/Bmp5/Dll1/Fgfr2/Gata6/Il31ra/Rfx3/Wnt5a	9
GO:0002534	cytokine production involved in inflammatory	9/1242	56/18303	0.012482	0.051687	0.03535	Abcd1/Chid1/Gbp5/Nod2/Nos2/Pycard/Sirpa/Tlr6/Tnf	9
GO:0051353	positive regulation of oxidoreductase activity	9/1242	56/18303	0.012482	0.051687	0.03535	Cdh3/Cyp27b1/Gch1/Il1b/Lgals9/Nod2/Npr3/Pdp2/Tnf	9
GO:0071392	cellular response to estradiol stimulus	9/1242	56/18303	0.012482	0.051687	0.03535	Abcb1a/Ccl2/Cflar/Enpp2/Itga2/Kif18a/Nefh/Ppargc1a/Sfrp1	9
GO:0007623	circadian rhythm	28/1242	264/18303	0.012673	0.052439	0.035864	Abcb1a/Ada/Adora1/Adora2a/Arntl2/Ass1/Atp7b/Bdnf/Csf2/Cyp7b1/Drd1/Egr1/Hcrtr2/Hnf1b/Htr7/Jun/Nampt/Ngf/Noct/Nos2/Pglyrp1/Ppargc1a/Cavin3/Ptger4/Relb/Rorb/Star/Tnf	28
GO:0061053	somite development	13/1242	96/18303	0.012773	0.052813	0.03612	Cobl/Dll1/Dmrt2/Foxc1/Frzb/Meox2/Pax1/Plxna2/Rbpj/Scx/Sfrp1/Wnt5a/Zeb2	13
GO:0002701	negative regulation of production of molecular mediator of immune response	7/1242	38/18303	0.01286	0.05302	0.036262	Hfe/Il13ra2/Il33/Siglec10/Tnf/Vsir/Xcl1	7
GO:0009069	serine family amino acid metabolic	7/1242	38/18303	0.01286	0.05302	0.036262	Cth/Gclc/Ggt1/Sardh/Slc7a11/Thnsl2/Txnrd1	7
GO:0042092	type 2 immune response	7/1242	38/18303	0.01286	0.05302	0.036262	Bcl3/Il31ra/Il33/Nlrp3/Nod2/Rsad2/Xcl1	7
GO:0060325	face morphogenesis	7/1242	38/18303	0.01286	0.05302	0.036262	Col1a1/Crispld2/Csrnp1/Pax9/Scx/Tiparp/Twist2	7
GO:0008038	neuron recognition	8/1242	47/18303	0.012932	0.053165	0.03636	Bdnf/Cxcr4/Epha4/Ephb2/Ephb3/Ncam1/Sema5a/Tnn	8
GO:0010518	positive regulation of phospholipase	8/1242	47/18303	0.012932	0.053165	0.03636	Adcyap1r1/Agtr1a/Ccl5/Fgfr2/Flt1/Gna13/Htr2a/Pla2g5	8
GO:0070229	negative regulation of lymphocyte apoptotic process	8/1242	47/18303	0.012932	0.053165	0.03636	Ada/Blm/Bmp4/Ccl5/Cd44/Dock8/Efna1/Serpinb9	8
GO:1901031	regulation of response to reactive oxygen species	8/1242	47/18303	0.012932	0.053165	0.03636	Fbln5/Gch1/Gpr37/Gpr37l1/Hk3/Pycr1/Sesn3/Tnf	8

ID	Description	GeneRatio	BgRatio	pvalue	p.adjust	qvalue	geneID	Count
GO:0001658	branching involved in ureteric bud morphogenesis	10/1242	66/18303	0.013033	0.053354	0.036489	Agtr1a/Bmp2/Bmp4/Cd44/Fat4/Hnf1b/Npnt/Pax8/Pgf/Wnt2b	10
GO:0002287	alpha-beta T cell activation involved in immune response	10/1242	66/18303	0.013033	0.053354	0.036489	Bcl3/Il23a/Lgals9/Nlrp3/Ptger4/Myb/Relb/Ripk2/Sema4a/Tnfsf18	10
GO:0042446	hormone biosynthetic process	10/1242	66/18303	0.013033	0.053354	0.036489	Bmp2/Bmp5/Cyp11b2/Cyp27a1/Egr1/Hfe/Hsd17b7/Ppargc1a/Star/Tg	10
GO:0051148	negative regulation of muscle cell differentiation	10/1242	66/18303	0.013033	0.053354	0.036489	Bdnf/Bmp2/Cflar/Cxcl10/Dll1/Ereg/Fgf9/Pdgfb/Pi16/Prdm6	10
GO:0055008	cardiac muscle tissue morphogenesis	10/1242	66/18303	0.013033	0.053354	0.036489	Ankrd1/Bmp2/Col11a1/Fgfr2/Foxc1/Hey2/Nrg1/Rbpj/Ryr2/Wnt5a	10
GO:1903428	positive regulation of reactive oxygen species biosynthetic process	10/1242	66/18303	0.013033	0.053354	0.036489	Ass1/Ddah2/Il1b/Klf2/Ptgs2/Slc5a3/Tlr2/Tlr5/Tlr6/Tnf	10
GO:0014068	positive regulation of phosphatidylinositol 3-kinase signaling	11/1242	76/18303	0.013193	0.053971	0.036911	Ccl5/Csf3/Dcn/Fgr/Flt1/Nrg1/Pdgfb/Ppard/Ror1/Selp/Tnf	11
GO:0048704	embryonic skeletal system	14/1242	107/18303	0.013339	0.05449	0.037266	Bmp4/Bmp7/Col11a1/Col2a1/Fgfr2/Fuz/Gas1/Hoxb5/Hoxb8/Hyal1/Mdfi/Mef2c/Mmp16/Twist2	14
GO:0106027	neuron projection organization	14/1242	107/18303	0.013339	0.05449	0.037266	Abcd1/Cask/Ctnnd2/Dnm3/Dock10/Efna1/Epha4/Ephb2/Ephb3/Grin2a/Kif1a/Lrp8/Shank1/Zdhhc15	14
GO:0048588	developmental cell growth	29/1242	277/18303	0.01338	0.05462	0.037355	Alcam/Bdnf/Cdh4/Cobl/Col14a1/Cxcl12/Cxcr4/Fdps/Flrt3/Mgll/Ngf/Nrg1/Nrn1/Pi16/Plxna3/Rgma/Sema3f/Sema4a/Sema4b/Sema5a/Sema6a/Sema6d/Slit2/Slit3/Sorbs2/Tnn/Twf2/Wnt5a/Zeb2	29
GO:0051607	defense response to virus	25/1242	230/18303	0.01341	0.054703	0.037412	Abcc9/Cd40/Cxcl10/Ifit3/Il15/Il1b/Il23a/Il33/Irf1/Acod1/Isg15/Itgax/Map3k14/Micb/Mmp12/Nlrc5/Nlrp3/Oasl/Pycard/Riok3/Rsad2/Slfn13/Stat1/Tlr3/Trim6	25

Supplementary Table 8 Clusterprofiler enriched GO terms in compare between Degenerated vs Con

ID	Description	GeneRatio	BgRatio	pvalue	p.adjust	qvalue	geneID	Count
GO:0031503	protein-containing complex localization	127/5495	291/18866	8.10E-08	0.00052	0.0005	FKBP4/UPF1/AKAP8L/NUP160/IFT88/NSUN2/TPR/AP3D1/PRKCZ/SMG6/KIFAP3/THOC1/DNM2/DLG3/XPO1/YTHDC1/KIF3C/SEH1L/EPS15/DYNLL1/BIRC5/IFT74/POLDIP3/IFT27/TRIP11/IFT52/NUP93/CLUAP1/RAB11A/CACNG7/LGI1/NUP88/NUP98/NUP155/NCBP2/RPS15/RAP1A/SMG7/CPSF3/DYNC2I2/GLE1/IQSEC2/NUP153/NUP85/THOC2/PCID2/NUP214/DLG4/TERF2/LTV1/NUP42/NCBP1/EIF4A3/SNAPIN/TERF1/NACC2/ATM/UPF2/DRD3/KIF5A/SMG1/DDX19B/WDR19/MCM3AP/CHTOP/SRSF2/KLHL21/SLBP/NOL6/CACNG2/TTC21A/KIF5C/DDX19A/NLGN1/SGCD/HRAS/CNIH2/EXOC3L1/EXOC3/SETD2/XPOT/TNFAIP2/DYNC2H1/IFT140/SMG5/EXOC3L4/EIF6/WDR33/SHISA6/SLU7/TUB/LCA5/MAGOHB/ARHGAP44/STX7/KIF5B/RAB8A/RPGR/CPSF2/EXOC1/THOC5/SRRM1/NOP9/NMD3/IFT81/WDR35/GSG1L/RAN/NETO1/IWS1/DYNC2I1/AGFG1/KIF17/RAE1/MX2/OPHN1/IFT57/NUP188/ATAD1/[illegible]/HSPB11/THOC6/[illegible]/TTC30	127
GO:1902115	regulation of organelle assembly	88/5495	194/18866	1.08E-06	0.0027	0.0024	ZMYND10/MARK4/CDKL5/FUZ/BRCA1/IFT88/TPR/MAP4/MAPK9/RNF4/RHOA/TSG101/EDN1/VDAC3/DNM2/CYLD/CHMP5/MTMR3/CDKL1/HCK/CEP76/CCP110/PTPRS/KAT2A/TBC1D30/KAT2B/RAB3GAP1/ARHGEF2/RAB3GAP2/SEPTIN7/CNOT1/LRFN1/LRRC4B/PRKAA1/TBC1D14/STAM/MNS1/MYLK3/PLK2/TBC1D7/DCDC2/C9orf72/PAN3/PTPRD/C10orf90/VPS11/SPICE1/PLK1/PIP4K2C/ATXN2L/STX18/TAPT1/PDCD6IP/AKAP13/TRAPPC12/RAB1B/RABEP2/IFT140/SRC/HTT/CCDC151/CHMP1B/SMAD4/SENP6/ATG5/NTRK3/MSN/CBLN1/CEP120/G3BP2/ULK1/TRIM37/SDCBP/SH3GLB1/DZIP1/PINK1/ATG3/WRAP73/SEPTIN9/DYNC1H1/GSN/SCFD1/G3BP1/STIL/NOTO/ENTR1/LCP1/CAV3	88
GO:0008361	regulation of cell size	83/5495	181/18866	1.25E-06	0.0027	0.0024	CDKL5/SEMA3G/FSTL4/RHOA/CLNS1A/MAP2/EDN1/DNM2/ULK2/ADD1/P2RX7/ARHGAP4/DPYSL2/ABL1/NRP1/RAB11A/PTPRS/MEGF8/LIMK1/SLC9A3R1/GNB3/SRF/SEMA5A/SLC12A7/PLEK/SPP1/SLC12A4/MACF1/PLXNA3/MAP1B/NGF/CDK4/SEMA4F/SLC12A6/RPTOR/VAV1/SEMA6C/GOLGA4/SLC12A9/PAK1/TMEM123/DRAXIN/DISC1/SEMA6B/IL7R/SEMA4C/AQP11/IST1/EFNA5/MAPT/CLN3/SEMA4A/MTOR/E2F4/SLC12A8/CDH4/SEMA4D/NTRK3/MSN/WNT5A/GDI1/SLC26A5/LPAR3/RARG/VAV2/ISLR2/AKT1/SLC12A1/VAV3/SCTR/ANO6/CXCL12/TNR/LRRC8A/OLFM1/RTN4R/ANXA7/SEMA3C/VEGFA/ANAPC2/PTEN/CDK5R1/CAV3	83
GO:0060491	regulation of cell projection assembly	84/5495	185/18866	1.78E-06	0.00289	0.0025	ZMYND10/RALA/MARK4/CDKL5/FUZ/WAS/IFT88/SH3YL1/MAP4/HDAC4/RAP1GAP/CAPZB/VDAC3/DNM2/CYLD/P2RX7/ICAM1/NRP1/PALM/CDKL1/ZMYND8/CCP110/CAV1/KANK1/TBC1D30/RIPOR2/SRF/HRG/ACTR3/RHOQ/SEPTIN7/CCR7/AVIL/CCL21/FNBP1L/MSTN/MNS1/DEF8/BCAS3/WDPCP/MYO10/TBC1D7/DCDC2/DOCK11/FER/EPS8/DMTN/CARMIL2/NLGN1/TAPT1/HRAS/EPS8L2/RABEP2/CDC42EP4/FAM110C/ARAP1/IFT140/SRC/HTT/CCDC151/MTOR/PLEKHM1/KLHL41/CDC42EP3/ARHGAP44/ATG5/CEP120/BIN3/ABITRAM/CDC42EP1/PODXL/DZIP1/AKIRIN1/ATG3/KLF5/WRAP73/SEPTIN9/ARF6/PPP1R16B/PRKCD/TACSTD2/NOTO/E	84
GO:0046777	protein autophosphorylation	102/5495	237/18866	3.15E-06	0.00409	0.0036	CDKL5/FYN/MVP/DAPK2/EIF2AK2/SLK/FGFR2/FGFR3/PRKACA/TXK/MKNK1/PTPRC/ULK2/EIF2AK1/MAPKAPK5/TRPM7/TYRO3/JAK2/ABL1/MKNK2/VRK1/RASSF2/HCK/EEF2K/DYRK1B/CAV1/ENG/TESK1/CAMKK2/PDGFRB/STK16/STK11/TEK/FLT3/PRKCG/MAP2K2/CSNK1G2/PDGFRA/RIPK1/PDPK1/EGFR/TAF1/TNKS1BP1/PAK1/ATM/TAOK2/VEGFC/FER/UHMK1/TNIK/SMG1/TAOK1/DDR2/MELK/DAPK3/INSR/OXSR1/PEAK1/YES1/ERN1/CSF1R/EPHB3/CHEK2/IRAK1/CHP1/SRC/MTOR/DDR1/PRKD2/ALK/CLK3/FLT1/STK4/ERBB4/ZAP70/LCK/PPP2R5E/WNK2/EPHA8/CAMK2B/STK24/SLA/ULK1/EPHB1/AATK/PIM3/ERBB2/GRK5/PDGFA/CDK12/PINK1/AKT1/CLK1/MUSK/FRK/MYLK2/RAD50/PASK/MARK2/BLK/LY	102
GO:0010970	transport along microtubule	74/5495	161/18866	4.15E-06	0.00411	0.0036	IFT88/DTNBP1/KIF1B/AP3D1/PRKCZ/AP3M2/KIFAP3/CAMSAP3/DYNC1I2/MAP2/KIF3C/DYNLL1/IFT74/SUN2/IFT27/TRIP11/IFT52/CLUAP1/AP3B2/KLC3/HSPB1/STK11/DYNC2I2/MAP1B/TERF2/AP3B1/ARL8B/KIF13A/MAPK8IP3/RHOT2/PEX14/SNAPIN/BICD1/DST/KIF5A/WDR19/AP3S2/COPG2/SUN1/TTC21A/KIF5C/CNIH2/TRAK1/MAPT/DYNC2H1/IFT140/BLOC1S5/CLN3/HTT/TUB/LCA5/KIF23/RAB27B/KIF5B/RPGR/IFT81/MAP1A/FLOT2/APBA1/WDR35/OPA1/RHOT1/DYNC2I1/SYBU/KIF17/BICD2/KIF1A/DYNC1H1/IFT57/ARL8A/HSPB11/WASF1/TTC30B/DYNLL2	74
GO:0120032	regulation of plasma membrane bounded cell projection assembly	82/5495	183/18866	4.45E-06	0.00411	0.0036	ZMYND10/RALA/MARK4/CDKL5/FUZ/WAS/IFT88/SH3YL1/MAP4/HDAC4/RAP1GAP/CAPZB/VDAC3/DNM2/CYLD/P2RX7/ICAM1/NRP1/PALM/CDKL1/ZMYND8/CCP110/CAV1/KANK1/TBC1D30/RIPOR2/SRF/HRG/ACTR3/RHOQ/SEPTIN7/CCR7/AVIL/CCL21/FNBP1L/MSTN/MNS1/DEF8/BCAS3/WDPCP/MYO10/TBC1D7/DCDC2/DOCK11/FER/EPS8/DMTN/CARMIL2/NLGN1/TAPT1/HRAS/EPS8L2/RABEP2/CDC42EP4/ARAP1/IFT140/HTT/CCDC151/MTOR/PLEKHM1/KLHL41/CDC42EP3/ARHGAP44/ATG5/CEP120/BIN3/ABITRAM/CDC42EP1/PODXL/DZIP1/AKIRIN1/ATG3/KLF5/WRAP73/SEPTIN9/ARF6/PPP1R16B/PRKCD/TACSTD2/NOTO/ENTR1/MIEN1	82
GO:0016569	covalent chromatin modification	185/5495	481/18866	5.28E-06	0.00427	0.0037	CREBBP/PAF1/JARID2/AKAP8L/DPF1/BRCA1/UBR2/PHF20/ZZZ3/RSF1/HDAC4/JMJD6/MBD3/BAZ2A/SIRT6/SRCAP/WDR70/NCOA1/HUWE1/ATXN7L3/UIMC1/KDM2B/JAK2/SETD1A/TTLL12/VRK1/BRMS1L/TBL1X/JADE3/HSF4/CTCF/KAT8/RNF40/EYA1/DOT1L/AKAP8/MIER2/PHF14/KDM4C/KAT2A/HDAC5/NSD2/ING4/APOBEC1/PHC1/BRD8/MACROH2A1/KAT2B/KANSL3/ATF2/ASH1L/KDM5B/RBBP5/KMT2A/ZNF541/SET/EPC1/NAA50/NCOA3/H1-3/KDM5C/KDM4B/ASH2L/PER2/PRKAA1/KDM6B/PRMT7/MORC2/DPF2/CDC73/DDB2/TAF5L/EPC2/PWP1/IRF4/JDP2/WDR61/USP3/GLYR1/CBX8/DMRTC2/SIRT3/CARM1/PADI3/SETDB1/SNCA/TAF1/NSD3/ATM/KAT14/MTA2/KAT6B/BRPF1/ELK4/CTBP1/MCM3AP/CHTOP/CRTC2/LMNA/ATXN7/DTX3L/TASOR/NIPBL/KDM1B/TAF10/KMT2D/OTUB1/SETD5/H1-4/UBE2E1/ASXL1/ZNF274/PHF8/TADA2B/KDM2A/MSL2/DMAP1/PER1/SETD2/SMYD3/PCGF3/NAP1L2/TET3/H1-2/MSL1/TRRAP/WDR5B/PIWIL2/PHF2/ZNF335/CTR9/PRKD2/JADE2/SMAD4/HLCS/EP400/OGT/PADI2/CHD5/ARID4A/PAXIP1/ATG5/MIER1/SDR16C5/EYA2/EYA4/MYOD1/MPHOSPH8/USP36/AICDA/BRD1/TRIM37/PHF20L1/CDK2/KAT7/RPS6KA4/BAP1/PINK1/SART3/PHF10/IWS1/PRDM16/CUL4B/SMYD2/CHEK1/DDB1/TET1/UBE2B/PRDM9/SETDB2/PRDM13/RUVBL1/PRMT5/VEGFA/PRKCD/ING5/RYBP/ACTL6B/DYDC1/GTF2B/MBD	185

GO:0016570	histone modification	179/5495	468/18866	1.10E-05	0.00788	0.0069	CREBBP/PAF1/JARID2/AKAP8L/DPF1/BRCA1/UBR2/PHF20/ZZZ3/RSF1/HDAC4/JMJD6/MBD3/BAZ2A/SIRT6/SRCAP/WDR70/NCOA1/HUWE1/ATXN7L3/UIMC1/KDM2B/JAK2/SETD1A/TTLL12/VRK1/BRMS1L/TBL1X/JADE3/HSF4/CTCF/KAT8/RNF40/EYA1/DOT1L/AKAP8/MIER2/PHF14/KDM4C/KAT2A/HDAC5/NSD2/ING4/PHC1/BRD8/MACROH2A1/KAT2B/KANSL3/ATF2/ASH1L/KDM5B/RBBP5/KMT2A/ZNF541/SET/EPC1/NAA50/NCOA3/H1-3/KDM5C/KDM4B/ASH2L/PER2/PRKAA1/KDM6B/PRMT7/DPF2/CDC73/DDB2/TAF5L/EPC2/PWP1/IRF4/JDP2/WDR61/USP3/GLYR1/CBX8/DMRTC2/SIRT3/CARM1/PADI3/SETDB1/SNCA/TAF1/NSD3/ATM/KAT14/MTA2/KAT6B/BRPF1/ELK4/CTBP1/MCM3AP/CHTOP/CRTC2/LMNA/ATXN7/DTX3L/NIPBL/KDM1B/TAF10/KMT2D/OTUB1/SETD5/H1-4/UBE2E1/ASXL1/ZNF274/PHF8/TADA2B/KDM2A/MSL2/DMAP1/PER1/SETD2/SMYD3/PCGF3/NAP1L2/TET3/H1-2/MSL1/TRRAP/WDR5B/PIWIL2/PHF2/ZNF335/CTR9/PRKD2/JADE2/SMAD4/HLCS/EP400/OGT/PADI2/CHD5/ARID4A/PAXIP1/ATG5/MIER1/SDR16C5/EYA2/EYA4/MYOD1/USP36/BRD1/TRIM37/PHF20L1/CDK2/KAT7/RPS6KA4/BAP1/PINK1/SART3/PHF10/IWS1/PRDM16/CUL4B/SMYD2/CHEK1/	179
GO:0099111	microtubule-based transport	83/5495	190/18866	1.28E-05	0.00829	0.0072	IFT88/OFD1/DTNBP1/KIF1B/AP3D1/PRKCZ/AP3M2/KIFAP3/CAMSAP3/DYNC1I2/MAP2/RFX3/KIF3C/DYNLL1/IFT74/SUN2/TTLL1/IFT27/TRIP11/IFT52/CLUAP1/AP3B2/KLC3/DNAH11/HSPB1/SLC9A3R1/STK11/DYNC2I2/MAP1B/TERF2/AP3B1/ARL8B/KIF13A/MAPK8IP3/RHOT2/GAS8/PEX14/ADCY10/SNAPIN/BICD1/DST/SPEF2/KIF5A/WDR19/AP3S2/COPG2/SUN1/TTC21A/KIF5C/CNIH2/TRAK1/MAPT/DYNC2H1/IFT140/BLOC1S5/CLN3/HTT/TUB/LCA5/KIF23/RAB27B/KIF5B/RPGR/IFT81/MAP1A/FLOT2/APBA1/WDR35/OPA1/RHOT1/NPHP3/DYNC2I1/SYBU/KIF17/BICD2/KIF1A/DYNC1H1/IFT57/ARL8A/HSPB11/WASF1/TTC30B/DYNLL2	83
GO:0070507	regulation of microtubule cytoskeleton organization	82/5495	190/18866	2.46E-05	0.01451	0.0126	FKBP4/MARK4/BRCA1/TACC3/PHLDB1/EPHA3/TPR/RNF4/RHOA/ROCK1/RASSF1/GBA2/CAMSAP3/MAP2/XPO1/CYLD/MAPRE3/CHMP5/NSFL1C/ABL1/RASSF7/NIN/CEP76/MID1/CCP110/STMN2/MET/KAT2A/SLAIN2/CEP70/KAT2B/ARHGEF2/AKAP9/TTBK2/MAP1B/PRKAA1/TRIM54/KIF11/SNCA/PLK2/PAK1/DIXDC1/BICD1/TRIM36/C10orf90/TAOK1/CLASP2/SPICE1/ATXN7/BMERB1/PLK1/PDCD6IP/PKHD1/CKAP5/EFNA5/CIB1/MAPT/TOGARAM2/DCTN1/CHMP1B/GEN1/SENP6/APC2/CEP120/CHMP3/MAP1A/TRIM37/XRCC3/CLASP1/CHEK1/BICD2/RAE1/ATF5/MARK2/STMND1/DYNC1H1/POC1A/STIL/FAM107A/MAPRE2/CDK5R1/CAV3	82
GO:0030705	cytoskeleton-dependent intracellular transport	85/5495	200/18866	3.51E-05	0.01761	0.0153	IFT88/DTNBP1/KIF1B/AP3D1/PRKCZ/AP3M2/KIFAP3/CAMSAP3/DYNC1I2/MAP2/KIF3C/DYNLL1/HOOK2/IFT74/SUN2/IFT27/TRIP11/IFT52/CLUAP1/AP3B2/KLC3/HSPB1/STK11/DYNC2I2/MYO5C/MAP1B/TERF2/AP3B1/ARL8B/KIF13A/FNBP1L/MAPK8IP3/RHOT2/PEX14/SNAPIN/MYO10/BICD1/DST/KIF5A/MYO1E/WDR19/AP3S2/COPG2/SUN1/MYO1A/TTC21A/HOOK3/KIF5C/CNIH2/TRAK1/MAPT/DYNC2H1/IFT140/BLOC1S5/CLN3/HTT/MYO1C/MYO6/TUB/LCA5/KIF23/RAB27B/KIF5B/RPGR/IFT81/MAP1A/FLOT2/APBA1/WDR35/OPA1/RHOT1/DYNC2I1/SYBU/MYO7B/KIF17/BICD2/KIF1A/MYO1D/DYNC1H1/IFT57/ARL8A/HSPB11/WASF1/TTC30B/	85
GO:1905383	protein localization to presynapse	10/5495	11/18866	3.53E-05	0.01761	0.0153	HSPB1/DVL1/TERF2/MAPK8IP3/KIF5A/KIF5C/RAB27B/KIF5B/MAP1A/WNT7A	10
GO:0006402	mRNA catabolic process	145/5495	376/18866	4.52E-05	0.02088	0.0182	UPF1/PSMC4/RNH1/EDC4/THRAP3/PUM2/RPL18/ROCK1/PSME4/SMG6/IGF2BP2/MLH1/TNRC6C/XPO1/TUT7/KHSRP/XRN2/RPL6/FUS/TNRC6A/CIRBP/HNRNPM/GSPT1/RPS16/RPS19/CACNG7/HSPB1/EXOSC3/MTPAP/APOBEC1/MAPK14/TENT4A/NCBP2/RPS15/SMG7/SET/ETF1/TENT4B/RPL5/FASTKD3/CNOT1/PCID2/NUP214/PSMA1/RPL36/LSM7/LSM4/RPS15A/AGO4/DHX34/DHX9/RC3H1/NCBP1/RPS6/PNPT1/RPS24/HNRNPD/WDR61/PARN/FTO/EIF4A3/PSMB6/RPL13A/DIS3L2/RPS3A/RPL37/TNKS1BP1/ATM/UBC/UPF2/NBAS/BOLL/PAN3/SMG1/PSMD4/RPL26/RPL9/RPS14/SMG8/RPL13/UPF3A/EXOSC1/PAIP1/CSDC2/RPL15/PDE12/LSM1/RPLP2/ERN1/MEX3D/CNOT10/TENT5C/PSMD13/NANOS3/PLEKHN1/NANOS2/RPL12/RPL23A/YTHDF2/MTOR/SMG5/SKIV2L/LSM2/PSMB11/RPL41/RPS18/RPS28/SLFN14/MYEF2/FASTKD1/ELAVL4/AGO2/ANP32A/MAGOHB/TBRG4/DAZL/RBM46/PSMD2/SUPV3L1/RPL31/YWHAB/RC3H2/ZC3H14/HNRNPC/AKT1/PSMA2/PSME1/PSMD11/EDC3/MOV10/NT5C3B/PSMD6/PS	145
GO:0006109	regulation of carbohydrate metabolic process	88/5495	210/18866	4.84E-05	0.02088	0.0182	SLC4A1/MLXIPL/NUP160/TPR/DGAT2/PDK3/HDAC4/RORA/FAM3A/SIRT6/SLC4A4/DUSP12/C1QTNF3/SEH1L/P2RX7/MAEA/NUP93/GAPDHS/PRKAG2/NUP88/KAT2A/NUP98/GNB3/NUP155/KAT2B/PFKFB4/POMC/PLEK/LEPR/NUP153/NUP85/NUP214/DYRK2/CBFA2T3/PRKAA1/INPP5K/NUP42/LHCGR/NCOR1/PMAIP1/IGFBP4/ARNT/SNCA/SOGA1/PDK1/PHKG2/JMJD8/GPER1/FOXK1/SERPINA12/MIDN/DDIT4/PFKFB3/INSR/LEP/PTPN2/ZBTB7A/ZBTB20/PPARA/SRC/MTOR/LCMT1/PPP1R3G/EIF6/PDK2/RUBCNL/OGT/PPP1CA/SCARB2/IFNG/RORC/AKT2/IRS2/ADCYAP1R1/AKT1/CRY1/DDB1/RAE1/MYOG/PASK/FOXA2/PPP1R3B/BAD/NUP188/PPP1R3D/PTH1R/KHK/PHLDA2	88
GO:0051222	positive regulation of protein transport	137/5495	354/18866	5.96E-05	0.02411	0.021	CD38/NADK/GIPR/FYN/CASR/RIPOR1/TPR/CASP8/TM7SF3/IPO5/PRKCZ/HYAL2/CSNK2A2/PRKACA/TSG101/SAR1A/C1QTNF3/OXCT1/HUWE1/ACHE/DNM1L/MAVS/P2RX7/JAK2/XBP1/GZMB/TM9SF4/MYOM1/PARD6A/ZFAND1/OAZ1/YWHAE/MAPK14/GOLPH3/TFDP2/SEC16B/SOX4/YWHAH/ADORA2A/NR1H2/ANO1/PRKAA1/EDEM1/YWHAQ/IL10/IL33/TLR2/KIF20B/BCAS3/PMAIP1/SIRT3/SAE1/GOLPH3L/EGFR/PAK1/B3GAT3/VEGFC/BMP6/ADCY8/UBE2J2/VPS11/GPER1/ITGAM/NLGN2/PTGER4/FGA/C2CD2L/LEP/HRAS/GLMN/ATG13/ARIH2/DMAP1/AKAP5/HCAR2/CADM1/ABAT/GAS6/CIB1/UBL4B/CHP1/CLN3/HLA-DRB1/PPIA/MYO18A/FLNA/SRC/MYO1C/CD2AP/UNC13B/SREBF2/HLA-E/SORL1/ECT2/IFNG/SLC30A8/SEC16A/PPM1A/MFF/SERP1/DRD2/ADAM8/KIF5B/EXOC1/AACS/DOC2B/ACSL3/USP36/AKT2/ERBB2/CLEC5A/PPP3CB/IRS2/YWHAB/MCU/SH3GLB1/TMEM30A/RAN/BAP1/PINK1/SYBU/CAPN10/CLEC9A/BLK/HPS4/SHH/BAD/ARF6/ACSL4/PRKCD/TREM2/	137

GO:0000280	nuclear division	161/5495	428/18866	8.07E-05	0.02508	0.0218	NCAPD2/AKAP8L/TACC3/UBR2/TPR/ANAPC4/CDK13/ASPM/RHOA/SMC1A/MLH1/EDN1/CDC14A/NDC80/PDS5B/SEH1L/CHMP5/NSFL1C/ANAPC5/BIRC5/HIRA/DMC1/VRK1/MYBL2/CDC25B/CEP192/NAA10/RAB11A/ARHGEF10/AKAP8/FZR1/CAV2/RAD51C/TENT4A/MSH3/RAD1/MACROH2A1/PDGFRB/ACTR3/STAG1/KIF14/TEX14/NAA50/PDS5A/PCID2/HSPA2/PSMG2/INO80/PSMC3IP/TOP2A/VPS4A/SPIRE1/AGO4/USP44/BRIP1/BRDT/KIF11/KIF20B/DMRTC2/POGZ/HORMAD1/DIS3L2/DYNC1LI1/TERF1/ATM/DSN1/EPS8/DRD3/TDRD9/CCNB2/CDC25C/NPM2/KASH5/LRP5/HFM1/TGFA/CLASP2/SPICE1/NIPBL/SUN1/SHOC1/PLK1/MEI1/GOLGA2/DAPK3/INSR/PDE3A/MUS81/CENPS/PPP2R2D/TOM1L2/AURKAIP1/CTDNEP1/SPHK1/CNTD1/SH2B1/RAD51B/PLCB1/CHEK2/KNTC1/L3MBTL1/PSMD13/FANCA/NANOS2/SLX4/TOGARAM2/ANAPC7/FLNA/PIWIL2/EME2/LCMT1/WEE2/KIFC1/CORT/TERB1/PTTG2/CHMP1B/GEN1/NCAPD3/SPDYA/KIF23/DAZL/PIN1/SGO2/BUB3/WNT5A/SYCP2/CDCA8/CCNA1/SPDL1/RB1/LPIN1/KIF2C/MKI67/KIF2A/CDC14B/REC8/XRCC3/RAN/KIFC2/CLASP1/CHEK1/BMP7/DUSP1/RAD50/MOV10L1/WRAP73/SMC2/UBE2B/NC	161
GO:0032535	regulation of cellular component size	146/5495	383/18866	8.20E-05	0.02508	0.0218	ALS2/CDKL5/SEMA3G/WAS/FSTL4/RHOA/GBA2/WDR1/CLNS1A/CDHR2/ADD2/CAPZB/MAP2/EDN1/DNM2/ULK2/ADD1/ARHGAP28/P2RX7/ARHGAP4/ICAM1/PLEKHG2/DPYSL2/ABL1/NRP1/CDHR5/PICK1/HCK/RAB11A/PTPRS/MEGF8/ACTR3C/LIMK1/KANK1/SLC9A3R1/GNB3/SRF/SEMA5A/SLC12A7/ACTR3/PLEK/SPP1/SLC12A4/VASP/MKKS/CCR7/MACF1/GMFG/HIP1R/PLXNA3/MAP1B/SWAP70/NGF/SPIRE1/AVIL/CDK4/SEMA4F/TMOD1/CCL21/FCHSD2/ABI2/SLC12A6/SSH2/RPTOR/VAV1/SEMA6C/GOLGA4/SLC12A9/PAK1/ESAM/FER/EPS8/TMEM123/DMTN/SNF8/CARMIL2/ALOX15/DRAXIN/DISC1/LMOD3/RICTOR/SEMA6B/IL7R/SEMA4C/RAB3B/FLII/AQP11/CDC42EP4/IST1/EFNA5/MAPT/CLN3/SEMA4A/SPTAN1/MYO1C/MTOR/E2F4/SLC12A8/ARPC4/CDH4/CDC42EP3/SSH1/CTNNA2/FHOD3/PPP1CA/SEMA4D/NTRK3/MSN/WNT5A/CAPZA2/CYRIA/ABITRAM/GDI1/SLC26A5/CDC42EP1/LPAR3/RARG/VAV2/ISLR2/AKT1/SLC12A1/VAV3/SCTR/ARPC5L/ANO6/BAIAP2/CXCL12/TNR/RAB22A/ARFIP1/LRRC8A/OLFM1/RTN4R/ANXA7/SEMA3C/GSN/ARF6/ARHGAP40/VEG	146
GO:0044262	cellular carbohydrate metabolic process	116/5495	294/18866	8.34E-05	0.02508	0.0218	SLC4A1/PHKA2/DGAT2/PDK3/HDAC4/RORA/FAM3A/SIRT6/DUSP12/C1QTNF3/B4GALT1/MTMR2/MAEA/GNPTG/PYGL/PHKB/HAS3/PRKAG2/KAT2A/GNB3/GNPTAB/KAT2B/PFKFB4/GBE1/POMC/PLEK/LEPR/PCK1/DYRK2/IDUA/CBFA2T3/ENOSF1/PER2/INPP5K/COQ3/ANGPTL3/IMPA1/PPP1R1A/DYSF/LHCGR/NCOR1/IMPA2/PMAIP1/IGFBP4/PGD/SNCA/CSGALNACT1/SOGA1/EXT2/PDK1/PHKG2/GALK2/B3GNT7/GNE/HK3/PHKG1/GPER1/FOXK1/SERPINA12/MOGAT2/MIDN/DDIT4/PGM2/GLB1/B3GNT2/PFKFB3/INSR/COQ2/LEP/GK5/PTPN2/B3GNT4/B3GALT5/PPARA/SRC/MTOR/GK/LCMT1/PPP1R3G/RPEL1/PDK2/RUBCNL/OGT/PPP1CA/SCARB2/RORC/IDH1/AKT2/INPP5E/IRS2/B3GALNT1/ADCYAP1R1/GYG1/AKT1/ITPK1/CRY1/PPP1R2/EXT1/DDB1/MYOG/PASK/PGM1/FOXA2/UGP2/PPP1R3B/RAD/INPP5A/PPP1R3D/CSGALNACT2/PCK2/IDNK/PTH1R/KHK/PTE	116
GO:0016573	histone acetylation	69/5495	159/18866	8.49E-05	0.02508	0.0218	CREBBP/DPF1/BRCA1/PHF20/ZZZ3/RSF1/MBD3/SRCAP/NCOA1/JADE3/CTCF/KAT8/PHF14/KAT2A/ING4/BRD8/KAT2B/KANSL3/ATF2/KMT2A/SET/EPC1/NAA50/NCOA3/DPF2/TAF5L/EPC2/IRF4/GLYR1/SNCA/TAF1/KAT14/KAT6B/BRPF1/CTBP1/MCM3AP/CRTC2/TAF10/SETD5/TADA2B/MSL2/DMAP1/PER1/NAP1L2/MSL1/TRRAP/PIWIL2/JADE2/SMAD4/EP400/OGT/CHD5/PAXIP1/ATG5/SDR16C5/MYOD1/BRD1/PHF20L1/KAT7/RPS6KA4/PHF10/IWS1/CHEK1/RUVBL1/ING5/ACTL6B/GTF2B/MRGBP/MORF	69
GO:0098840	protein transport along microtubule	35/5495	68/18866	8.75E-05	0.02508	0.0218	IFT88/KIFAP3/CAMSAP3/KIF3C/DYNLL1/IFT74/IFT27/TRIP11/IFT52/CLUAP1/HSPB1/DYNC2I2/TERF2/MAPK8IP3/KIF5A/WDR19/TTC21A/KIF5C/DYNC2H1/IFT140/TUB/LCA5/RAB27B/KIF5B/RPGR/IFT81/MAP1A/WDR35/DYNC2I1/KIF17/KIF1A/IFT57/HSPB11/TTC30B/DYNLL2	35
GO:0099118	microtubule-based protein transport	35/5495	68/18866	8.75E-05	0.02508	0.0218	IFT88/KIFAP3/CAMSAP3/KIF3C/DYNLL1/IFT74/IFT27/TRIP11/IFT52/CLUAP1/HSPB1/DYNC2I2/TERF2/MAPK8IP3/KIF5A/WDR19/TTC21A/KIF5C/DYNC2H1/IFT140/TUB/LCA5/RAB27B/KIF5B/RPGR/IFT81/MAP1A/WDR35/DYNC2I1/KIF17/KIF1A/IFT57/HSPB11/TTC30B/DYNLL2	35
GO:0043467	regulation of generation of precursor metabolites and energy	70/5495	162/18866	8.91E-05	0.02508	0.0218	SLC4A1/MLXIPL/NUP160/TPR/RHOA/HDAC4/SIRT6/SLC4A4/SEH1L/P2RX7/NUP93/ANTKMT/GAPDHS/PRKAG2/NUP88/NUP98/NUP155/PFKFB4/POMC/NUP153/NUP85/SLC25A23/NUP214/DYRK2/CBFA2T3/PRKAA1/INPP5K/NUP42/PNPT1/COX17/NCOR1/ARNT/SNCA/ATPSCKMT/PHKG2/COX7A1/JMJD8/VCP/DDIT4/PFKFB3/INSR/PDE12/ZBTB7A/ZBTB20/PPARA/MTOR/PPP1R3G/EIF6/COA8/RUBCNL/PIK3CA/OGT/PPP1CA/IFNG/AKT2/IRS2/PINK1/AKT1/PRDM16/RAE1/MYOG/PASK/PPP1R3B/NUP188/PPP1R3D/IL4/KHK/SHMT2/PHLDA2/PGK2	70
GO:0016049	cell growth	181/5495	490/18866	9.60E-05	0.02589	0.0226	CD38/CDKL5/CDK11A/SEMA3G/EXTL3/BCAR1/FSTL4/KIF26A/RHOA/PRKCZ/HYAL2/DERL2/CDHR2/TSG101/ENO1/SIRT6/MAP2/EDN1/ITCH/DNM2/SMARCA2/EPB41L3/ULK2/KDM2B/ARHGAP4/DPYSL2/ABL1/NRP1/CRKL/NIN/HNF4A/CSNK2A1/RBBP7/RAB11A/PTPRS/MEGF8/CACNG7/LIMK1/DVL1/LGI1/CYP27B1/CHPT1/SRF/SEMA5A/HRG/STK11/KIF14/SPP1/NPPB/ZNF639/USP9X/FLRT1/MACF1/SMARCA4/INO80/BST2/UNC13A/PAK4/PLXNA3/PPT1/MAP1B/PPARG/POSTN/NGF/CDC73/RERG/SEMA4F/GATA4/NCBP1/CCN3/PLAA/PAK6/FBLN5/RPTOR/IGFBP4/SEMA6C/CPNE9/VGLL4/GOLGA4/AGTR1/EGFR/CDKN2A/C9orf72/PTPRJ/PAK1/TAOK2/SPOCK1/SORBS2/G6PD/DRAXIN/DISC1/TGFBR2/APBB2/FXN/KMT2D/HDGFL2/SEMA6B/CDKN2AIP/SEMA4C/USP47/AKAP13/LAMB2/NPPA/CCDC85B/SPHK1/RPS6KA3/ARIH2/F2/IST1/TSPYL2/EFNA5/CIB1/NDUFA13/MAPT/PPARA/SLIT1/SEMA4A/SLC9A6/MTOR/SLC44A4/DDR1/TRIM40/CPNE1/CDK11B/INHBA/CDH4/SMAD4/ZEB2/PAPPA2/ADAM15/SEMA4D/PIN1/BCL11A/NTRK3/WNT5A/RIMS1/BIN3/SUPV3L1/BRAT1/SH3GL2/ULK1/CPNE6/ERBB2/GDI1/PPP3CB/RB1/TP53TG5/PDLIM5/LPAR3/CEP43/RARG/SDCBP/TNN/SYT2/BAP1/ISLR2/AKT1/CXCL12/TNR/MINAR1/KRT17/OLFM1/SH3BP4/RTN4R/FOXL2/CCAR2/SEMA3C/VEGFA/GDF9/	181

GO:0051047	positive regulation of secretion	131/5495	340/18866	0.000107	0.02651	0.0231	CD38/TAC1/NADK/GIPR/HFE/GAB2/CASR/DTNBP1/SLC4A8/TM7SF3/GAL/PLA2G10/ABCB11/TSG101/IL4R/EDN1/C1QTNF3/OXCT1/ACHE/DNM1L/SNX5/P2RX7/JAK2/XBP1/MYOM1/PARD6A/RAB3D/RAB3A/GHRHR/NCS1/SLC6A4/GOLPH3/RAB3GAP1/SPP1/NPPB/RAB9A/SOX4/ADORA2A/UNC13A/NR1H2/ANO1/VPS4A/LACRT/EDNRB/IL10/STAM/IL33/TLR2/SYTL2/RAB15/SIRT3/GOLPH3L/TMF1/SNCA/EGFR/VEGFC/DRD3/BMP6/ADCY8/SNF8/CLASP2/GPER1/AP1G1/PRRT2/NLGN1/ITGAM/NLGN2/PDCD6IP/PTGER4/FGA/C2CD2L/LEP/GLMN/NPPA/ALOX12B/HCAR2/CADM1/ABAT/FFAR4/HLA-DRB1/PPIA/MYO18A/TRPV1/SRC/CD2AP/UNC13B/HLA-E/ITSN1/SORL1/INHBA/CADPS/SMAD4/IFNG/SLC30A8/ATG5/RAB27B/SERP1/DRD2/ADAM8/KIF5B/EXOC1/AACS/DOC2B/ACSL3/CLEC5A/PPP3CB/IRS2/MCU/SDCBP/CACNA1H/HLA-E/SYT10/PINK1/CCK/CLASP1/SYBU/CXCL12/CAPN10/CLEC9A/SLC6A1/	131
GO:1904951	positive regulation of establishment of protein localization	141/5495	370/18866	0.000109	0.02651	0.0231	CD38/NADK/GIPR/FYN/CASR/RIPOR1/TPR/CASP8/TM7SF3/IPO5/PRKCZ/HYAL2/CSNK2A2/PRKACA/TSG101/SAR1A/C1QTNF3/OXCT1/HUWE1/ACHE/DNM1L/MAVS/P2RX7/JAK2/XBP1/GZMB/TM9SF4/MYOM1/PARD6A/ZFAND1/OAZ1/YWHAE/MAPK14/GOLPH3/TFDP2/SEC16B/SOX4/YWHAH/ADORA2A/NR1H2/ANO1/PRKAA1/EDEM1/YWHAQ/IL10/IL33/TLR2/KIF20B/BCAS3/PMAIP1/SIRT3/SAE1/GOLPH3L/EGFR/CCT6A/PAK1/B3GAT3/VEGFC/CCT5/BMP6/ADCY8/UBE2J2/VPS11/GPER1/ITGAM/NLGN2/PTGER4/FGA/C2CD2L/LEP/HRAS/GLMN/ATG13/ARIH2/DMAP1/AKAP5/HCAR2/CADM1/ABAT/GAS6/CIB1/UBL4B/CHP1/CLN3/HLA-DRB1/PPIA/MYO18A/FLNA/SRC/MYO1C/CD2AP/UNC13B/SREBF2/HLA-E/SORL1/ECT2/IFNG/SLC30A8/SEC16A/PPM1A/MFF/SERP1/DRD2/ADAM8/KIF5B/EXOC1/AACS/CEP120/DOC2B/ACSL3/USP36/AKT2/ERBB2/CLEC5A/PPP3CB/IRS2/YWHAB/CCT7/MCU/SH3GLB1/TMEM30A/RAN/BAP1/PINK1/SYBU/CAPN10/CLEC9A/BLK/HPS4/SHH/BAD/ARF6/ACSL4/PRK	141
GO:0006475	internal protein amino acid acetylation	71/5495	166/18866	0.000117	0.02651	0.0231	CREBBP/DPF1/BRCA1/PHF20/ZZZ3/RSF1/MBD3/SRCAP/NCOA1/NAA10/JADE3/CTCF/KAT8/PHF14/KAT2A/ING4/BRD8/KAT2B/KANSL3/ATF2/KMT2A/SET/EPC1/NAA50/NCOA3/DPF2/TAF5L/EPC2/IRF4/GLYR1/SNCA/TAF1/KAT14/KAT6B/BRPF1/CTBP1/MCM3AP/CRTC2/TAF10/SETD5/TADA2B/MSL2/DMAP1/PER1/NAP1L2/MAPT/MSL1/TRRAP/PIWIL2/JADE2/SMAD4/EP400/OGT/CHD5/PAXIP1/ATG5/SDR16C5/MYOD1/BRD1/PHF20L1/KAT7/RPS6KA4/PHF10/IWS1/CHEK1/RUVBL1/ING5/ACTL6B/GTF2B/MRGBP/MORF4L2	71
GO:0051056	regulation of small GTPase mediated signal transduction	125/5495	323/18866	0.000122	0.02651	0.0231	ALS2/RALBP1/TRIO/KITLG/GDI2/RHOA/MAP4K4/RAP1GAP/ARHGEF1/DNM2/ARHGAP28/ARHGAP4/PLEKHG2/JAK2/ABL1/NRP1/MYO9B/CRKL/ARHGAP5/ARHGEF7/TSC2/ARHGEF10/RASAL3/RASIP1/MET/KANK1/RAPGEF1/TNFAIP1/MADD/ARHGDIB/RIPOR2/PDGFRB/RALGPS2/ARHGEF2/MFN2/APOA1/KIF14/DENND1A/RHOQ/ARHGAP9/IQSEC2/TRIP10/CHN1/CGNL1/STARD13/NGF/GPR55/DENND4C/FGD4/ARHGAP17/RHOT2/VAV1/ABL2/PSD2/TIAM2/EPS8/OBSCN/RASA2/F11R/ABR/DLC1/ARHGAP42/CRK/RHOH/AKAP13/RHOD/HEG1/HRAS/KCTD13/ARHGAP1/A2M/EPS8L2/GPR4/ARHGAP45/RASA3/ARAP1/RALGAPA2/SRC/CDC42SE1/CD2AP/DENND4B/ITSN1/ARHGAP19/NOTCH2/ARHGEF26/ECT2/OGT/LRRC59/SYDE2/ARHGEF25/ARHGAP44/RALGAPB/STARD8/ITGA3/COL3A1/CADM4/ARHGAP26/FGD3/GDI1/ITPKB/ARHGEF9/IRS2/EPHB2/DEPDC7/VAV2/ADCYAP1R1/NOTCH1/VAV3/RHOT1/MCF2L/CDC42SE2/ARHGAP8/OPHN1/RTN4R/LYN/ARHGAP22/ARF6/ARHGAP40/ARHGEF16/LPAR1/	125
GO:0030098	lymphocyte differentiation	140/5495	368/18866	0.000126	0.02651	0.0231	CD79B/AP3D1/RHOA/PRKCZ/CBFB/HDAC4/BCL3/RORA/JMJD6/TCF3/IL4R/TCF7/PTPRC/CYLD/BAX/PPP2R3C/TYRO3/ABL1/LGALS1/XBP1/DNAJA3/CD79A/TNFSF8/KAT2A/HDAC5/ZBTB16/MDK/IL23A/CD83/VNN1/SRF/DROSHA/CD86/TUSC2/IL18R1/GON4L/LEPR/PLA2G2D/GPR89A/STK11/CD80/FLT3/PCK1/BTN2A2/SOX4/TNFSF9/PCID2/CCR7/ZBTB1/NDFIP1/AP3B1/IL2RA/RC3H1/DOCK10/IL10/IRF4/IL21/LMBR1L/VAV1/ZC3H8/DOCK11/PTPRJ/ATM/DCLRE1C/MR1/CMTM7/PLCL2/FZD7/CD3G/PGLYRP2/IL23R/VCAM1/SLAMF6/TGFBR2/STAT6/AXL/PRDX2/FADD/RBPJ/IRF2BP2/RHOH/IL7R/ONECUT1/PTGER4/PIK3CD/LEP/PTPN2/SART1/SOX12/ZBTB7A/RNF41/GAS6/IKZF1/TNFRSF18/FANCA/SEMA4A/IL27/PLCG2/MTOR/CD3E/FNIP1/PSMB11/SP3/INHBA/NOTCH2/IL12RB1/CR2/IFNG/ATG5/MAFB/IL12B/ZAP70/RORC/LCK/ADAM8/CDH17/KLF6/ERBB2/AICDA/ITPKB/PPP3CB/RC3H2/TPD52/KAT7/ZFPM1/RSAD2/ID2/EGR3/LRRC8A/PKNOX1/SHH/PNP/BAD/MFNG/IL4/CTSL/METTL3/MMP14/HMGB3/IE	140
GO:0006367	transcription initiation from RNA polymerase II promoter	79/5495	189/18866	0.000126	0.02651	0.0231	CREBBP/MED24/PSMC4/NR1H3/CBFB/RORA/TEAD2/BAX/POLR2E/POLR2F/HNF4A/MAZ/MED25/MED26/POLR2I/GTF2H1/E2F3/SRF/SUB1/KAT2B/NRBP1/CCN2/NR2C1/MED20/GTF2F1/THRA/NR1H2/PPARG/MED10/CDK7/HNF1A/CDK4/YAP1/GTF2A2/NR1I2/TAF1/POLR2K/MED27/GTF2A1/TAF3/TAF10/RBPJ/PPM1D/NR1D2/NPPA/MED16/NR2C2/POLR2L/CTNNBIP1/POLR2A/MAML2/RXRA/PPARA/TEAD1/GTF2E2/TEAD4/GTF2A1L/TAF4B/NOTCH2/ESRRA/PAXIP1/TAF7L/RORC/NR2F1/RXRG/MED1/ERCC3/MED17/RARG/MED6/NOTCH1/NR5A1/TAF2/CCNC/GTF2B/TAF4/PTEN/POLR2G/NR0B1	79
GO:0018394	peptidyl-lysine acetylation	73/5495	172/18866	0.000127	0.02651	0.0231	CREBBP/DPF1/BRCA1/PHF20/ZZZ3/RSF1/MBD3/SRCAP/NCOA1/JADE3/CTCF/KAT8/PHF14/KAT2A/ING4/BRD8/KAT2B/KANSL3/ATF2/KMT2A/SET/EPC1/NAA50/NCOA3/SOX4/PRKAA1/DPF2/TAF5L/EPC2/HINT2/IRF4/GLYR1/SNCA/TAF1/KAT14/KAT6B/BRPF1/CTBP1/MCM3AP/CRTC2/KLF15/TAF10/SETD5/TADA2B/MSL2/DMAP1/PER1/NAP1L2/MSL1/TRRAP/PIWIL2/JADE2/SMAD4/EP400/OGT/CHD5/PAXIP1/ATG5/SDR16C5/MYOD1/BRD1/PHF20L1/KAT7/RPS6KA4/PHF10/IWS1/CHEK1/RUVBL1/ING5/ACTL6B/GTF2B/MRGBP/MORF4L2	73
GO:0001672	regulation of chromatin assembly or disassembly	17/5495	26/18866	0.000136	0.02749	0.0239	TPR/SIRT6/APOBEC1/MORC2/SETDB1/TLK2/TASOR/PHF8/PHF2/PADI2/MPHOSPH8/AICDA/TAL1/TET1/SETDB2/PARP10/ZNF304	17

GO:0043161	proteasome-mediated ubiquitin-dependent protein catabolic process	158/5495	424/18866	0.000159	0.03038	0.0265	HFE/RNF216/PSMC4/UBR2/CLEC16A/MAPK9/ANAPC4/RNF4/PSME4/UFD1/RNF126/DERL2/KLHL20/DNAJC10/SIRT6/UBE2K/ITCH/KEAP1/PCNP/XPO1/HUWE1/KLHL42/NSFL1C/ANAPC5/MAEA/FBXL19/CDC34/TRIM9/TRIB3/USP14/TBL1X/SGTA/FZR1/CAV1/SEC61B/DVL1/CUL2/CCDC47/TNFAIP1/UBE4A/TRIM38/FAF2/CSNK1A1/EDEM3/UCHL5/KIF14/FBXL5/RBCK1/PSMA1/CBFA2T3/DDA1/UBE4B/UBE2G1/ARNTL/EDEM1/USP44/IL33/PLAA/FOXF2/RNF144B/ARRB2/PSMB6/COP1/MARCHF6/SKP2/PLK2/GNA12/TLK2/TAF1/UBC/AMN1/FBXO4/DAB2/FBXL2/KLHL40/UBXN11/PSMD4/UBR1/UBE2J2/STT3B/VCP/BTRC/CUL5/PLK1/FBXO22/KCTD5/TMUB2/TMEM129/UBE2E1/SPSB1/USP19/COMMD1/SMARCC1/GLMN/KCTD13/SPSB4/ARIH2/GBA/SHARPIN/SOCS4/FBXL7/PSMD13/ZNRF1/UBE2H/NCCRP1/ANAPC7/PCBP2/PELI1/FBXL22/TOPORS/CD2AP/WWP2/FBXO48/PSMB11/NEMF/AGAP3/DNAJB12/DCAF11/OGT/SH3RF2/JKAMP/FBXL20/BUB3/UBXN1/FBXO38/YOD1/PSMD2/ANAPC16/MAP1A/SPOP/SDCBP/CDK2/RCHY1/AKT1/PSMA2/PSME1/CUL4B/TRIB2/DMAC2/DDB1/PSMD11/CHFR/UBE2B/IFI27/ECPAS/PSMD6/PSMB7/ZER1/SHH/CCAR2/SPS	158
GO:1990089	response to nerve growth factor	28/5495	52/18866	0.000161	0.03038	0.0265	TAC1/KIF1B/EEF2K/STMN2/PTN/RAPGEF1/KAT2A/EHD1/RAP1A/NGF/DYNC1LI2/PDPK1/EIF4A3/CRK/EEF2/CIB1/MAPT/TRPV1/NTF4/UBE3A/NTRK3/SH3GL2/NTF3/AKT1/KCNC1/ARF6/WASF1/PTEN	28
GO:0048285	organelle fission	175/5495	476/18866	0.000164	0.03038	0.0265	NCAPD2/AKAP8L/TACC3/UBR2/TPR/ANAPC4/CDK13/ASPM/RHOA/SMC1A/MLH1/EDN1/CDC14A/DNM2/NDC80/PDS5B/SEH1L/CHMP5/DNM1L/NSFL1C/ANAPC5/BIRC5/HIRA/DMC1/VRK1/MYBL2/CDC25B/ACOT8/CEP192/NAA10/RAB11A/ARHGEF10/AKAP8/FZR1/CAV2/RAD51C/TENT4A/MSH3/RAD1/MACROH2A1/PDGFRB/ACTR3/MTFR1L/STAG1/KIF14/SEC16B/TEX14/NAA50/PDS5A/PCID2/HSPA2/PSMG2/INO80/PSMC3IP/TOP2A/VPS4A/AP3B1/SPIRE1/AGO4/USP44/BRIP1/BRDT/KIF11/KIF20B/DMRTC2/POGZ/HORMAD1/DIS3L2/DYNC1LI1/TERF1/ATM/DSN1/EPS8/DRD3/TDRD9/CCNB2/CDC25C/NPM2/KASH5/LRP5/HFM1/PEX19/TGFA/CLASP2/SPICE1/NIPBL/SUN1/SHOC1/PLK1/MEI1/GOLGA2/DAPK3/INSR/PDE3A/MUS81/CENPS/PPP2R2D/TOM1L2/AURKAIP1/CTDNEP1/SPHK1/CNTD1/SH2B1/RAD51B/PLCB1/CHEK2/KNTC1/L3MBTL1/PSMD13/MAPT/FANCA/NANOS2/SLX4/TOGARAM2/ANAPC7/FLNA/PIWIL2/EME2/LCMT1/WEE2/KIFC1/CORT/TERB1/PTTG2/CHMP1B/GEN1/NCAPD3/SPDYA/KIF23/DAZL/PIN1/SGO2/MFF/BUB3/WNT5A/SYCP2/CDCA8/CCNA1/SPDL1/RB1/LPIN1/MCU/KIF2C/MKI67/KIF2A/CDC14B/REC8/XRCC3/MARCHF5/RAN/PINK1/KIFC2/CLASP1/OPA1/CHEK1/BMP7/DUSP1/RAD50/MOV10L1/WRAP73/SMC2/UBE2B/NCAPH/MX2/PRDM9/PRMT5/SMC3/CHAMP1/PP	175
GO:0048588	developmental cell growth	95/5495	237/18866	0.000181	0.0313	0.0273	CDKL5/SEMA3G/FSTL4/PRKCZ/SIRT6/MAP2/EDN1/DNM2/ULK2/ARHGAP4/DPYSL2/ABL1/NRP1/NIN/RAB11A/PTPRS/MEGF8/CACNG7/LIMK1/DVL1/SRF/SEMA5A/STK11/SPP1/USP9X/FLRT1/MACF1/UNC13A/PLXNA3/MAP1B/POSTN/NGF/SEMA4F/GATA4/PLAA/PAK6/SEMA6C/CPNE9/GOLGA4/C9orf72/PAK1/SORBS2/G6PD/DRAXIN/DISC1/TGFBR2/KMT2D/SEMA6B/SEMA4C/AKAP13/LAMB2/NPPA/ARIH2/IST1/EFNA5/MAPT/PPARA/SLIT1/SEMA4A/SLC9A6/MTOR/DDR1/CPNE1/CDH4/ZEB2/SEMA4D/PIN1/BCL11A/NTRK3/WNT5A/RIMS1/SH3GL2/ULK1/CPNE6/GDI1/PPP3CB/PDLIM5/LPAR3/RARG/TNN/SYT2/ISLR2/CXCL12/TNR/OLFM1/RTN4R/FOXL2/SEMA3C/VEGFA/GDF9/WASF1/ANAPC2/RGS2/CDK5R1/CAV3	95
GO:0010498	proteasomal protein catabolic process	177/5495	483/18866	0.000183	0.0313	0.0273	OSBPL7/HFE/RNF216/PSMC4/UBR2/CLEC16A/MAPK9/ANAPC4/RNF4/PSME4/UFD1/RNF126/PRKACA/DERL2/KLHL20/DNAJC10/SIRT6/UBE2K/ITCH/KEAP1/PCNP/XPO1/HUWE1/KLHL42/NSFL1C/ANAPC5/MAEA/FBXL19/CDC34/TRIM9/TRIB3/USP14/TBL1X/UGGT2/RNF40/SGTA/FZR1/CAV1/SEC61B/DVL1/CUL2/CCDC47/TNFAIP1/PRPF19/UBE4A/TRIM38/FAF2/CSNK1A1/EDEM3/UCHL5/KIF14/FBXL5/RBCK1/PRKCG/PSMA1/CBFA2T3/DDA1/UBE4B/UBE2G1/ARNTL/EDEM1/USP44/IL33/PLAA/FOXF2/RNF144B/ARRB2/PMAIP1/PSMB6/COP1/TMF1/MARCHF6/SKP2/PLK2/GNA12/TLK2/TAF1/UBC/AMN1/FBXO4/DAB2/FBXL2/KLHL40/UBXN11/PSMD4/UBR1/UBE2J2/STT3B/VCP/BTRC/CUL5/PLK1/FBXO22/UBXN6/KCTD5/TMUB2/TMEM129/UBE2E1/SPSB1/USP19/COMMD1/SMARCC1/GLMN/KCTD13/SPSB4/ARIH2/GBA/AQP11/SHARPIN/SOCS4/RNF41/TMEM259/FBXL7/PSMD13/ZNRF1/UBE2H/NCCRP1/RNFT1/ANAPC7/PCBP2/PELI1/FBXL22/TOPORS/CD2AP/WWP2/FBXO48/PSMB11/GET4/NEMF/AGAP3/DNAJB12/DCAF11/OGT/SH3RF2/JKAMP/FBXL20/BUB3/UBXN1/FBXO38/YOD1/PSMD2/ANAPC16/MAP1A/TOR1A/SPOP/SDCBP/CDK2/RCHY1/PINK1/AKT1/PSMA2/PSME1/CUL4B/TRIB2/DMAC2/DDB1/PSMD11/CHFR/UBE2B/IFI27/ECPAS/PSMD6/PSMB7/OPHN1/ZER1/SHH/CCAR2/RHBDF1/SPSB3/	177
GO:0032886	regulation of microtubule-based process	96/5495	240/18866	0.000184	0.0313	0.0273	FKBP4/MARK4/BRCA1/TACC3/PHLDB1/EPHA3/TPR/RNF4/RHOA/ROCK1/RASSF1/GBA2/CAMSAP3/MAP2/XPO1/CYLD/MAPRE3/CHMP5/KLHL42/NSFL1C/ABL1/RASSF7/NIN/CEP76/MID1/CCP110/STMN2/DNAH11/MET/KAT2A/SLAIN2/CEP70/KAT2B/ARHGEF2/STK11/BBS2/MKKS/MACF1/AKAP9/TTBK2/MAP1B/PRKAA1/TRIM54/KIF11/SNCA/PLK2/PAK1/DIXDC1/BICD1/TRIM36/C10orf90/TAOK1/CLASP2/SPICE1/ATXN7/BMERB1/PLK1/TACR3/PDCD6IP/TTLL6/PKHD1/CNIH2/CKAP5/EFNA5/CIB1/MAPT/TOGARAM2/DCTN1/CHMP1B/GEN1/TPPP2/SENP6/APC2/CEP120/CHMP3/ERBB2/MAP1A/TRIM37/XRCC3/CLASP1/RHOT1/CHEK1/BICD2/RAE1/ATF5/MARK2/STMND1/DYNC1H1/POC1A/STIL/FAM107A/MAPRE2/EPPIN/SEMG2/CDK5R1/CAV3	96
GO:1903829	positive regulation of cellular protein localization	129/5495	338/18866	0.000194	0.03193	0.0278	MARK4/FYN/PRKCH/RIPOR1/EPHA3/TPR/ATP2B4/CASP8/ABCA7/IPO5/HYAL2/CSNK2A2/PRKACA/TFRC/TSG101/SAR1A/NDC80/CYLD/HUWE1/MAVS/JAK2/GZMB/TM9SF4/PARD6A/RAB11A/ZFAND1/OAZ1/PTN/YWHAE/MAPK14/TFDP2/STK11/SEC16B/YWHAH/MGAT3/ABHD17A/LGALS3/PRKAA1/SYT11/EDEM1/YWHAQ/LRP4/KIF20B/PDPK1/GAS8/BCAS3/PMAIP1/SAE1/PARP1/EGFR/CCT6A/PAK1/B3GAT3/CCT5/BICD1/EPB41/UBE2J2/VPS11/DTX3L/NIPBL/GPER1/PLK1/CACNG2/ITGAM/TRAPPC12/LEP/HRAS/ATG13/ARIH2/DMAP1/AKAP5/F2/GAS6/CIB1/UBL4B/MAPT/CHP1/CLN3/FLNA/SRC/MYO1C/CD2AP/CD247/SREBF2/SORL1/SSH1/ECT2/IFNG/ERBB4/SEC16A/PPM1A/MFF/ITGA3/MSN/KIF5B/ACSL3/USP36/AKT2/ERBB2/NMD3/CENPQ/EPHB2/MAP1A/YWHAB/CCT7/EPB41L2/KAT7/SH3GLB1/TMEM30A/RAN/BAP1/PINK1/AKT1/RER1/HPS4/SHH/BAD/ARF6/PRKCD/PPP1R9B/TREM2/ARHGEF16/TYROBP/ENTR1/MAPK8/ZIC1/RHOU/CDK5R1/CTSE1	129

GO:0022604	regulation of cell morphogenesis	182/5495	499/18866	0.000198	0.03193	0.0278	PLXND1/CDKL5/SEMA3G/FYN/ZRANB1/CD44/HEXB/FSTL4/RHOA/WDR1/PLXNA2/CAPZB/MAP2/DNM2/EPB41L3/ULK2/DNM1L/LZTS3/ARHGAP4/ICAM1/DPYSL2/ABL1/NRP1/PALM/CRKL/MYH9/NIN/HCK/ARHGEF7/OLFM4/METRN/EEF2K/RAB11A/MYH14/PTPRS/MEGF8/CACNG7/PTN/LIMK1/KANK1/DVL1/C1QBP/SLC9A3R1/MDK/NEDD9/SRF/SEMA5A/STK11/APOA1/MYL12B/SPP1/RHOQ/SEPTIN7/RREB1/MACF1/YWHAH/CHN1/UNC13A/PLXNA3/MAP1B/DLG4/POSTN/NGF/LRP4/AGO4/CAPRIN1/SEMA4F/PLXNC1/PLAA/PARP6/ABI2/FGD4/SEMA6C/WDPCP/CPNE9/GOLGA4/LIMD1/MYO10/TIAM2/GNA12/DOCK5/PAK1/TAOK2/EPS8/PTPRD/CFDP1/TNIK/F11R/DMTN/EPB41/DRAXIN/DISC1/DLC1/EPB42/CRK/DAPK3/SEMA6B/RHOH/SEMA4C/NLGN1/LINGO1/FGA/RHOD/DAB1/CDC42EP4/F2/AMIGO1/IST1/CSF1R/EPHB3/TRAK1/EFNA5/CIB1/BCL9L/ARAP1/MAPT/SEMA4A/PRPF40A/FLNA/SRC/CDC42SE1/PARVA/LST1/ATP10A/CDH4/CDC42EP3/ZMYM4/ZEB2/SKIL/ARHGAP44/SEMA4D/DNMBP/ZMYM6/BCL11A/UBE3A/NTRK3/MSN/WNT5A/CAMK2B/RIMS1/CPNE6/FGD3/GDI1/SLC26A5/LZTS1/CASS4/CDC42EP1/NTNG2/BRWD3/EPHB2/PDLIM5/LPAR3/CHODL/TLX2/SYT2/ISLR2/OPA1/SYNE3/BAIAP2/KNDC1/CXCL12/TNR/SPARC/KIF1A/MARK2/CDC42SE2/MOV10/OLFM1/RTN4R/	182
GO:0150115	cell-substrate junction organization	47/5495	102/18866	0.000206	0.03193	0.0278	EPHA3/LAMA3/LIMCH1/SLK/RHOA/ROCK1/TESK2/MAP4K4/CAMSAP3/TRIP6/ABL1/NRP1/ARHGEF7/DUSP22/HRG/TEK/MACF1/LAMC1/TLN1/PDPK1/BCAS3/PIP5K1A/WDPCP/PTPRJ/TAOK2/DST/PTPRK/DMTN/CLASP2/DLC1/DAPK3/RHOD/PEAK1/CD151/EFNA5/KRT14/SRC/TNS1/LAMB3/CLASP1/ARF6/KRT5/VEGFA/FAM107A/MAPRE2/PTEN/MMP14	47
GO:0018205	peptidyl-lysine modification	151/5495	405/18866	0.000214	0.03193	0.0278	CREBBP/JARID2/DPF1/BRCA1/PHF20/NUP160/ZZZ3/TPR/RSF1/RNF4/HMG20B/HDAC4/JMJD6/MBD3/PIAS2/SRCAP/NCOA1/SEH1L/SETD1A/TTLL12/RANGAP1/JADE3/NUP93/CTCF/ANTKMT/KAT8/EYA1/DOT1L/PIAS4/PHF14/KDM4C/NUP88/KAT2A/NSD2/NUP98/ING4/BRD8/NUP155/MACROH2A1/KAT2B/KANSL3/ATF2/ASH1L/RBBP5/KMT2A/SET/EPC1/NAA50/LIAS/NCOA3/SIRT5/H1-3/SOX4/NUP153/NUP85/NUP214/ASH2L/DOHH/PRKAA1/DPF2/LOXL2/TAF5L/EPC2/PWP1/NUP42/HINT2/IRF4/WDR61/GLYR1/CBX4/DMRTC2/SIRT3/SAE1/SETDB1/ARNT/CAMKMT/LIPT1/SNCA/TAF1/NSD3/KAT14/ATPSCKMT/KAT6B/BRPF1/CTBP1/MCM3AP/CRTC2/LMNA/KLF15/TAF10/KMT2D/KIAA1586/SETD5/H1-4/ZNF274/TADA2B/RELA/MSL2/DMAP1/PER1/SETD2/SMYD3/NAP1L2/TET3/H1-2/MSL1/TRRAP/WDR5B/PIWIL2/TOPORS/ZNF335/CTR9/EEF1AKMT2/USPL1/JADE2/SMAD4/EP400/OGT/CHD5/ARID4A/SENP6/PAXIP1/ATG5/PLOD3/BCL11A/SDR16C5/MYOD1/SETD4/BRD1/PHF20L1/KAT7/RPS6KA4/PHF10/IWS1/PRDM16/SMYD2/CHEK1/RAE1/PRDM9/PLOD2/SETDB2/RU	151
GO:0035303	regulation of dephosphorylation	85/5495	209/18866	0.000219	0.03193	0.0278	PTBP1/BOD1L1/EIF2AK2/WNK1/ROCK1/SMG6/PPP2R3A/CAMSAP3/PTPRC/DLG3/MTMR2/FKBP1A/PPP2R3C/JAK2/MTMR3/CEP192/PPP1R37/DMPK/VRK3/PTN/YWHAE/PHACTR1/PDGFRB/PLEK/SMG7/FKBP15/SET/PTPA/CRY2/ZCCHC9/LGALS3/INPP5K/SWAP70/PPP1R1A/SYTL2/PPP2R1B/TIPRL/ENSA/B3GAT3/PPP1R1C/PPP4R1/SPPL3/PPP1R15B/RRP1B/PPP4R2/DLC1/PPP1R36/PPP1R14D/PPP1R14A/WDR81/PPP2R2D/GBA/CHP1/SRC/HTT/SFI1/MTOR/SMG5/ITGA1/SH3RF2/IFNG/SEMA4D/RIMBP2/KNL1/PIN1/PPP2R5E/DRD2/CDCA2/FARP1/SAG/YWHAB/RBM26/PINK1/PPP1R2/MPHOSPH10/MYO1D/PPP1R3B/MTMR9/PPP1R16B/PRKCD/PPP1R9B/PPP1R42/PPP1R1B/PPP1R7/TMEM225	85
GO:0050804	modulation of chemical synaptic transmission	167/5495	454/18866	0.000222	0.03193	0.0278	CD38/TAC1/CACNA2D2/FYN/RNF216/NCDN/DTNBP1/SLC4A8/PRKCZ/PRKACA/EDN1/SLC1A3/MTMR2/ACHE/DNM1L/JAK2/ABL1/PICK1/PACSIN2/ZMYND8/PLCB4/RAB11A/DMPK/PTPRS/CACNG7/RAB3A/CRTC1/PTN/NPTX2/NCS1/DVL1/LGI1/SLC6A4/KAT2A/P2RX3/SRF/HRH2/RAB3GAP1/RAP1A/IQSEC2/NAPB/PRKCG/AKAP9/YWHAH/ADORA2A/UNC13A/AKAP12/MAP1B/DLG4/PTPRA/SYT11/NGF/EGLN1/CDH11/ARRB2/EIF4A3/CACNA1A/SH3GL1/SNAPIN/SNCA/PLK2/CPLX2/EGFR/CASK/STAR/EIF4EBP2/DGKZ/DRD3/PLCL2/ADCY8/ABR/DISC1/ABHD6/GPER1/RAPSN/CACNG2/PRRT2/RAB3B/NLGN1/NLGN2/SYNPO/SYT12/ATP2A2/NPAS4/HRAS/RIN1/CNIH2/KCTD13/PPFIA3/AKAP5/PLCB1/ABAT/BTBD9/MAPT/PRKAR1B/CLN3/SRC/UNC13B/MTOR/GRM3/AGER/GRID2IP/NTF4/SHISA6/SSH1/CHRNA7/PLCG1/FBXL20/SORCS2/ADCY1/DRD2/KIF5B/CAMK2B/RIMS1/RAB8A/CBLN1/DLGAP3/CHRNB4/EPHB1/TSHZ3/SLC7A11/GRM4/LZTS1/PPP3CB/NTNG2/NTF3/EPHB2/MAP1A/TOR1A/LRRC4/GRIN3A/ATP1A2/CLSTN2/APBA1/NETO1/PINK1/RHOT1/SCTR/SYBU/BAIAP2/TPRG1L/TNR/SLC6A1/CYP46A1/SLC24A2/OPHN1/VGF/MPP2/CACNA1B/ATAD1/SYNGR1/OXTR/EGR2/PPP1R9B/GRM7/PLG/PNKD/FAM107A/TYR	167
GO:0006090	pyruvate metabolic process	65/5495	152/18866	0.000223	0.03193	0.0278	SLC4A1/MLXIPL/NUP160/TPR/HAGH/PFKP/PKM/PDK3/HDAC4/ENO1/SIRT6/SLC4A4/SEH1L/P2RX7/PDPR/NUP93/GAPDHS/OGDH/PRKAG2/ENO3/NUP88/ALDOC/PDHX/NUP98/ENO2/LDHB/NUP155/PFKFB4/PCK1/NUP153/NUP85/NUP214/CBFA2T3/PRKAA1/NUP42/NCOR1/PFKL/ARNT/DLAT/PDK1/HK3/JMJD8/FOXK1/LDHC/DDIT4/PFKFB3/INSR/BSG/BPGM/ZBTB7A/ZBTB20/PPARA/HOGA1/EIF6/PDK2/OGT/IFNG/PFKM/GSTZ1/RAE1/MYOG/PGM1/NUP188/PCK2/PGK2	65
GO:0006338	chromatin remodeling	88/5495	218/18866	0.000232	0.03193	0.0278	JARID2/MPND/TPR/RSF1/HDAC4/PSME4/MBD3/BAZ2A/SIRT6/SMARCA2/SRCAP/SMARCD3/CENPM/SMCHD1/RBBP7/OIP5/KDM4C/SMARCD2/KAT2A/HDAC5/CHD4/APOBEC1/FAM172A/KAT2B/KDM5B/ARID1A/CENPL/CBX3/KDM5C/ZBTB1/SMARCA4/KDM4B/INO80/RRP8/KDM6B/MORC2/BRDT/CENPO/SMARCC2/RERE/SETDB1/GATAD2B/CDKN2A/PAK1/MTA2/CENPH/H4C8/NPM2/RBBP4/VPS72/PBRM1/TASOR/CENPV/INO80E/PHF8/TADA2B/SMARCC1/CENPS/DMAP1/ZBTB7A/H4C3/PHF2/CENPW/CHD5/KNL1/PABPC1L/MPHOSPH8/HCFC2/AICDA/CENPQ/RB1/HNRNPC/TOP1/CHD1L/TET1/SETDB2/ACTR5/RUVBL1/HMGB4/CENPA/ACTL6B/TNP2/CENPC/CHRAC1/MBD2/HMGB3/MIS18BP1/ZNF304	88
GO:0006413	translational initiation	79/5495	192/18866	0.000232	0.03193	0.0278	DHX33/TPR/EIF2AK2/RPL18/DHX29/EIF4G3/MTIF2/EIF2AK1/RPL6/EIF5/MTFMT/RPS16/RPS19/HSPB1/EIF3A/NCBP2/RPS15/GLE1/KHDRBS1/RPL5/RPL36/EIF2S3/RPS15A/EIF4E2/NCBP1/RPS6/RPS24/RPL13A/RPS3A/RPL37/EIF3H/EIF4EBP2/EIF3M/CDC123/UHMK1/BOLL/EIF4A2/PPP1R15B/RPL26/RPL9/RPS14/RPL13/PAIP1/RPL15/EIF4E1B/RPLP2/EIF3K/EIF3C/RXRA/RPL12/RPL23A/YTHDF2/MTOR/EIF3CL/RPL41/RPS18/MCTS1/RPS28/EIF6/C8orf88/DNAJC3/AGO2/PPP1CA/DAZL/RPL31/HABP4/EIF2B3/ALKBH1/EIF3I/RPS20/KLHL25/RPL24/RPL32/EIF4B/MTIF3/METTL3/RPL10A/R	79

GO:0002831	regulation of response to biotic stimulus	152/5495	409/18866	0.000243	0.03197	0.0278	TSPAN6/CREBBP/FYN/RNF216/PSMC4/NR1H3/TAB2/PUM2/PSME4/UFD1/FAM3A/PRKACA/PVR/TXK/ICAM3/UBE2K/ITCH/ARG2/MAVS/NLRC4/PPP2R3C/TYRO3/JAK2/TTLL12/TAB1/HCK/LILRB1/CACTIN/RPS19/CDC37/DDX58/C1QBP/HPX/IL23A/TRIM38/LY86/HRG/STAT1/IL18RAP/CD160/CD274/ZBP1/PSMA1/NR1H2/PPARG/TRIM5/SYT11/IL2RA/TRAFD1/LACRT/DHX9/IL21/NLRC5/PDPK1/ARRB2/VAV1/PSMB6/MUC4/SERPING1/PAK1/MR1/BMP6/CD96/GBP5/APPL1/TAB3/PSMD4/NLRX1/IL23R/SLAMF6/DCST1/IFI16/DTX3L/BTRC/TRIM44/AP1G1/CRK/PRDX2/FADD/POLR3D/KLK7/SERPINB9/SLC22A13/RELA/LEP/HRAS/PTPN2/A2M/CADM1/TBK1/USP18/SIGIRR/IFNLR1/IRF7/PSMD13/ILRUN/PCBP2/SRC/IL27/PLCG2/YTHDF2/SH2D1B/UNC13B/RAET1G/HLA-E/CHUK/PSMB11/IRGM/APOBEC3G/IL12RB1/IFNG/IL12B/WNT5A/DRD2/NRAS/ADAM8/FBXO38/POLR3B/PSMD2/MED1/PRKACB/PARP14/HLA-F/PSMA2/PSME1/NLRP10/RIOK3/PSMD11/PSMD6/PSMB7/TSPAN32/HMGB4/LYN/MUC2/PRKCD/XIAP/PSME2/TREM2/SEC14L1/IL4/MUC5B/HLA-	152
GO:0071426	ribonucleoprotein complex export from nucleus	57/5495	130/18866	0.000243	0.03197	0.0278	UPF1/AKAP8L/NUP160/NSUN2/TPR/SMG6/THOC1/XPO1/YTHDC1/SEH1L/POLDIP3/NUP93/NUP88/NUP98/NUP155/NCBP2/RPS15/SMG7/CPSF3/GLE1/NUP153/NUP85/THOC2/PCID2/NUP214/LTV1/NUP42/NCBP1/EIF4A3/UPF2/SMG1/DDX19B/MCM3AP/CHTOP/SRSF2/SLBP/NOL6/DDX19A/SETD2/XPOT/SMG5/EIF6/WDR33/SLU7/MAGOHB/CPSF2/THOC5/SRRM1/NOP9/NMD3/RAN/IWS1/AGFG1/RAE1/NUP188/CDC40/THOC6	57
GO:0099177	regulation of trans-synaptic signaling	167/5495	455/18866	0.000251	0.03197	0.0278	CD38/TAC1/CACNA2D2/FYN/RNF216/NCDN/DTNBP1/SLC4A8/PRKCZ/PRKACA/EDN1/SLC1A3/MTMR2/ACHE/DNM1L/JAK2/ABL1/PICK1/PACSIN2/ZMYND8/PLCB4/RAB11A/DMPK/PTPRS/CACNG7/RAB3A/CRTC1/PTN/NPTX2/NCS1/DVL1/LGI1/SLC6A4/KAT2A/P2RX3/SRF/HRH2/RAB3GAP1/RAP1A/IQSEC2/NAPB/PRKCG/AKAP9/YWHAH/ADORA2A/UNC13A/AKAP12/MAP1B/DLG4/PTPRA/SYT11/NGF/EGLN1/CDH11/ARRB2/EIF4A3/CACNA1A/SH3GL1/SNAPIN/SNCA/PLK2/CPLX2/EGFR/CASK/STAR/EIF4EBP2/DGKZ/DRD3/PLCL2/ADCY8/ABR/DISC1/ABHD6/GPER1/RAPSN/CACNG2/PRRT2/RAB3B/NLGN1/NLGN2/SYNPO/SYT12/ATP2A2/NPAS4/HRAS/RIN1/CNIH2/KCTD13/PPFIA3/AKAP5/PLCB1/ABAT/BTBD9/MAPT/PRKAR1B/CLN3/SRC/UNC13B/MTOR/GRM3/AGER/GRID2IP/NTF4/SHISA6/SSH1/CHRNA7/PLCG1/FBXL20/SORCS2/ADCY1/DRD2/KIF5B/CAMK2B/RIMS1/RAB8A/CBLN1/DLGAP3/CHRNB4/EPHB1/TSHZ3/SLC7A11/GRM4/LZTS1/PPP3CB/NTNG2/NTF3/EPHB2/MAP1A/TOR1A/LRRC4/GRIN3A/ATP1A2/CLSTN2/APBA1/NETO1/PINK1/RHOT1/SCTR/SYBU/BAIAP2/TPRG1L/TNR/SLC6A1/CYP46A1/SLC24A2/OPHN1/VGF/MPP2/CACNA1B/ATAD1/SYNGR1/OXTR/EGR2/PPP1R9B/GRM7/PLG/PNKD/FAM107A/TYR	167
GO:0048732	gland development	163/5495	443/18866	0.000258	0.03197	0.0278	PLXND1/FKBP4/JARID2/HFE/CAPN1/HGF/TNC/TG/PTCD2/FOXC1/FGFR2/PKM/FSTL3/ASNS/CSNK2A2/ACAT1/NCOA1/BAX/JAK2/ABL1/NRP1/CRKL/XBP1/ACO2/ARHGAP5/HNF4A/LAMA1/FA2H/RPGRIP1L/DBP/PTN/CAV1/MET/GHRHR/LHX3/SLC9A3R1/MDK/SRF/SLC29A1/HMGCS1/FGF1/PDGFRB/HRH2/WDR77/RAP1A/ASH1L/KDM5B/SERPINC1/STK11/APOA1/PKD2/ATP7B/PCK1/RREB1/GOT2/THRA/ZBTB1/PPAT/TWSG1/CDO1/LAMA5/SERPINF1/PDGFRA/HNF1A/IL10/PNPT1/HNRNPD/SLC6A3/SRD5A1/EGFR/NFIB/ATM/UPF2/QDPR/CCNB2/XDH/LRP5/TGFBR2/PITX2/BTRC/TAF10/STAT6/PRDX2/FADD/RBPJ/ONECUT1/INSR/ASXL1/ID4/RELA/SMARCC1/HRAS/PROP1/ZDHHC21/TYMS/DEAF1/FOXE1/CSF1R/EPHB3/ZNF703/PBX1/RXRA/SERPINA5/SULF2/IGF2R/SRC/PHF2/DDR1/ALDH1A2/SP3/SEC63/NOTCH2/SMAD4/PIK3CA/BTBD7/MAFB/ERBB4/HOXA9/UBE3A/MSN/WNT5A/DRD2/AACS/AKT2/ETS1/MED1/IRS2/PTPN3/PDGFA/RARG/WDR35/NOG/AKT1/NOTCH1/ID2/NPHP3/TFCP2L1/SERPINB5/NR5A1/BMP7/HMOX1/TBX3/PRMT5/USF2/SHH/SEMA3C/ARF6/GATA6/O	163
GO:0018393	internal peptidyl-lysine acetylation	69/5495	164/18866	0.00026	0.03197	0.0278	CREBBP/DPF1/BRCA1/PHF20/ZZZ3/RSF1/MBD3/SRCAP/NCOA1/JADE3/CTCF/KAT8/PHF14/KAT2A/ING4/BRD8/KAT2B/KANSL3/ATF2/KMT2A/SET/EPC1/NAA50/NCOA3/DPF2/TAF5L/EPC2/IRF4/GLYR1/SNCA/TAF1/KAT14/KAT6B/BRPF1/CTBP1/MCM3AP/CRTC2/TAF10/SETD5/TADA2B/MSL2/DMAP1/PER1/NAP1L2/MSL1/TRRAP/PIWIL2/JADE2/SMAD4/EP400/OGT/CHD5/PAXIP1/ATG5/SDR16C5/MYOD1/BRD1/PHF20L1/KAT7/RPS6KA4/PHF10/IWS1/CHEK1/RUVBL1/ING5/ACTL6B/GTF2B/MRGBP/MORF	69
GO:0006611	protein export from nucleus	76/5495	184/18866	0.000262	0.03197	0.0278	UPF1/AKAP8L/NUP160/RANBP3/NSUN2/TPR/SMG6/PRKACA/THOC1/STRADB/XPO1/YTHDC1/SEH1L/POLDIP3/RANGAP1/NUP93/NUP88/YWHAE/NUP98/NUP155/NCBP2/RPS15/SMG7/CPSF3/GLE1/XPO5/NUP153/NUP85/THOC2/PCID2/NUP214/LTV1/NUP42/TXN/NCBP1/EIF4A3/UPF2/PTPN14/UHMK1/SMG1/DDX19B/ANKLE1/MCM3AP/CHTOP/SRSF2/SLBP/NOL6/DDX19A/XPO6/SETD2/GAS6/XPOT/CHP1/SMG5/CCHCR1/EIF6/WDR33/SLU7/MAGOHB/PPM1A/CPSF2/THOC5/SRRM1/NOP9/XPO7/NMD3/RAN/IWS1/AGFG1/RAE1/IFI27/NUP188/CDC40/EGR2/THOC6/GTSE1	76
GO:0045785	positive regulation of cell adhesion	158/5495	428/18866	0.000267	0.03197	0.0278	FYN/CD44/PTPRU/AP3D1/RHOA/PRKCZ/ROCK1/CBFB/TFE3/FSTL3/MAP4K4/TFRC/KIFAP3/IL4R/DNM2/PTPRC/CYLD/CEACAM6/ICAM1/IFT74/JAK2/ABL1/NRP1/CRKL/LGALS1/XBP1/ARHGEF7/OLFM4/NFAT5/PIEZO1/DNAJA3/LILRB1/RASAL3/EBI3/PTN/CAV1/DOCK8/C1QBP/ZBTB16/MDK/IL23A/GCNT2/NEDD9/CD83/VNN1/HRG/CD86/HHLA2/NID1/CD160/APOA1/TEK/CD274/CD80/PCK1/BTN2A2/SOX4/RREB1/TNFSF9/CD70/TNFSF14/CCR7/ZBTB1/RIN2/AP3B1/IL2RA/DYSF/IL10/CCL21/IL21/PDPK1/SKAP1/VAV1/MYO10/MEGF10/DOCK5/HSD17B12/PTPRJ/PAK1/ATM/MAGI1/CCL28/DMP1/UTRN/F11R/DMTN/TNFRSF13C/ALOX15/IL23R/VCAM1/DISC1/TGFBR2/ICOS/CRK/SERPINF2/FADD/RHOH/IL7R/PLEKHA2/FGA/RELA/RHOD/LEP/DENND6A/FUT1/SART1/YES1/SOX12/IRAK1/CIB1/TNFRSF18/FLNA/SRC/CD3E/AGER/HLA-E/VIT/EMP2/ITGAV/PRKD2/IL12RB1/PIK3CA/IFNG/STK4/IL12B/ZAP70/LCK/ITGA3/WNT5A/ADAM8/ICOSLG/ERBB2/ITPKB/EPB41L4B/CASS4/ETS1/FLOT2/GPAM/PODXL/AKT1/VAV3/ECM2/EGR3/SMOC2/AFDN/CXCL12/BMP7/TNFRSF14/FOXA2/SHH/LYN/PNP/BAD/ALOX5/VEGFA/IL4/HL	158

GO:0031589	cell-substrate adhesion	135/5495	359/18866	0.000296	0.03429	0.0299	ITGAL/CD44/EPHA3/VAMP3/LAMC3/LIMCH1/SLK/RHOA/PRKCZ/ROCK1/TESK2/MAP4K4/CAMSAP3/DNM2/CEACAM6/TRIP6/ATRN/SIGLEC1/PXN/TRPM7/TYRO3/JAK2/ABL1/NRP1/CRKL/LGALS1/JAG1/ARHGEF7/OLFM4/PTN/KANK1/C1QBP/MDK/VWF/GCNT2/NEDD9/SRF/DUSP22/HRG/ITGB6/NID1/APOA1/KIF14/CCN2/TEK/RREB1/CCR7/MACF1/LAMA5/RIN2/ANGPTL3/POSTN/LAMC1/CCL21/SMAD6/FBLN5/PDPK1/SKAP1/BCAS3/PIP5K1A/WDPCP/MUC4/CASK/DOCK5/CDKN2A/HSD17B12/PTPRJ/TAOK2/FER/CCL28/SPOCK1/DMP1/UTRN/PTPRK/CD96/FZD7/DMTN/ALOX15/VCAM1/DISC1/CLASP2/ADAMTS9/DLC1/CRK/AXL/DAPK3/THBS3/PLEKHA2/ONECUT1/FGA/LAMB2/HPSE/RHOD/PEAK1/FUT1/ACER2/EPHB3/GAS6/EFNA5/CIB1/NF2/FLNA/SRC/PARVA/CD3E/DDR1/VIT/EMP2/ITGA1/ITGAV/PPFIA1/ADAM15/STK4/ITGA3/COL3A1/PIK3CB/EPHB1/CASS4/NTNG2/LAMB3/TNN/CLASP1/NOTCH1/ECM2/SMOC2/FAT2/MEGF9/VEGFA/TACSTD2/LYVE1/PLG/FAM107A/SORBS2/PTEN/MMP14	135
GO:1902017	regulation of cilium assembly	29/5495	56/18866	0.000301	0.03429	0.0299	ZMYND10/MARK4/CDKL5/FUZ/IFT88/MAP4/VDAC3/DNM2/CYLD/CDKL1/CCP110/TBC1D30/SEPTIN7/MNS1/TBC1D7/DCDC2/TAPT1/RABEP2/IFT140/HTT/CCDC151/ATG5/CEP120/DZIP1/ATG3/WRAP73/SEPTIN9/NOTO/ENTR1	29
GO:0071166	ribonucleoprotein complex localization	57/5495	131/18866	0.000309	0.03429	0.0299	UPF1/AKAP8L/NUP160/NSUN2/TPR/SMG6/THOC1/XPO1/YTHDC1/SEH1L/POLDIP3/NUP93/NUP88/NUP98/NUP155/NCBP2/RPS15/SMG7/CPSF3/GLE1/NUP153/NUP85/THOC2/PCID2/NUP214/LTV1/NUP42/NCBP1/EIF4A3/UPF2/SMG1/DDX19B/MCM3AP/CHTOP/SRSF2/SLBP/NOL6/DDX19A/SETD2/XPOT/SMG5/EIF6/WDR33/SLU7/MAGOHB/CPSF2/THOC5/SRRM1/NOP9/NMD3/RAN/IWS1/AGFG1/RAE1/NUP188/CDC40/THOC6	57
GO:0008334	histone mRNA metabolic process	16/5495	25/18866	0.00031	0.03429	0.0299	UPF1/SNRPD3/MTPAP/TENT4A/NCBP2/CPSF3/TENT4B/SNRPB/NCBP1/SNRPF/ZNF473/SNRPG/ATM/SLBP/LSM1/CPSF2	16
GO:0043470	regulation of carbohydrate catabolic process	42/5495	90/18866	0.000312	0.03429	0.0299	SLC4A1/MLXIPL/NUP160/TPR/HDAC4/SIRT6/SLC4A4/SEH1L/P2RX7/NUP93/GAPDHS/PRKAG2/NUP88/NUP98/NUP155/PFKFB4/NUP153/NUP85/NUP214/CBFA2T3/PRKAA1/NUP42/NCOR1/ARNT/PHKG2/JMJD8/DDIT4/PFKFB3/INSR/ZBTB7A/ZBTB20/PPARA/EIF6/OGT/PPP1CA/SCARB2/IFNG/RAE1/MYOG/PPP1R3B/NUP188/PPP1R3D	42
GO:0009451	RNA modification	70/5495	168/18866	0.000331	0.03573	0.0311	RPUSD1/NSUN2/METTL1/TRIT1/JMJD6/BUD23/HSD17B10/TRMT61A/AARS1/PUS7/APOBEC3H/CDK5RAP1/METTL4/RNMT/MTFMT/DTWD1/DUS4L/TRDMT1/FTSJ3/ELP4/PUS3/APOBEC1/NSUN4/TRMT1L/TRMT13/ZC3H13/EMG1/GTPBP3/NAT10/PARN/FTO/DUS3L/CDKAL1/TFB2M/RBM47/ELP6/ADAD1/METTL2B/TRMT10B/ALKBH3/TRUB2/ZCCHC4/LCMT2/METTL15/PUSL1/ELP5/CTU2/TRMT12/ADARB1/QTRT1/APOBEC3G/FDXACB1/AICDA/TRMU/ADARB2/THG1L/THUMPD3/TRMT5/ELP2/ELP1/PUS1/WDR4/ALKBH1/URM1/TSR3/OSGEP/KTI12/GAR1/METTL3/TRMT2A	70
GO:0022409	positive regulation of cell-cell adhesion	108/5495	279/18866	0.000337	0.03573	0.0311	FYN/CD44/PTPRU/AP3D1/RHOA/PRKCZ/CBFB/FSTL3/TFRC/KIFAP3/IL4R/PTPRC/CYLD/CEACAM6/ICAM1/LGALS1/XBP1/NFAT5/PIEZO1/DNAJA3/LILRB1/RASAL3/EBI3/CAV1/DOCK8/ZBTB16/MDK/IL23A/GCNT2/CD83/VNN1/CD86/HHLA2/CD160/CD274/CD80/PCK1/BTN2A2/SOX4/TNFSF9/CD70/TNFSF14/CCR7/ZBTB1/AP3B1/IL2RA/IL10/CCL21/IL21/PDPK1/SKAP1/VAV1/MYO10/MEGF10/PAK1/MAGI1/F11R/DMTN/TNFRSF13C/ALOX15/IL23R/VCAM1/TGFBR2/ICOS/SERPINF2/FADD/RHOH/IL7R/FGA/RELA/LEP/DENND6A/SART1/YES1/SOX12/IRAK1/SRC/CD3E/AGER/HLA-E/IL12RB1/PIK3CA/IFNG/IL12B/ZAP70/LCK/WNT5A/ADAM8/ICOSLG/ITPKB/ETS1/FLOT2/GPAM/PODXL/AKT1/EGR3/AFDN/BMP7/TNFRSF14/FOXA2/SHH/LYN/PNP/BAD/ALOX5/IL4/HLA-DPB1/CITED2	108
GO:0006401	RNA catabolic process	153/5495	415/18866	0.000354	0.03648	0.0318	UPF1/PSMC4/RNH1/NSUN2/EDC4/THRAP3/PUM2/RPL18/ROCK1/PSME4/SMG6/IGF2BP2/MLH1/TNRC6C/XPO1/TUT7/KHSRP/XRN2/RPL6/FUS/TNRC6A/CIRBP/HNRNPM/GSPT1/POP1/RPS16/RPS19/CACNG7/HSPB1/EXOSC3/TRDMT1/MTPAP/APOBEC1/MAPK14/TENT4A/DROSHA/NCBP2/RPS15/SMG7/SET/ETF1/TENT4B/RPL5/TRIR/FASTKD3/CNOT1/PCID2/NUP214/PSMA1/RPL36/LSM7/LSM4/RPS15A/AGO4/DHX34/DHX9/RC3H1/NCBP1/RPS6/PNPT1/RPS24/HNRNPD/WDR61/PARN/FTO/EIF4A3/PSMB6/RPL13A/DIS3L2/RPS3A/RPL37/TNKS1BP1/ATM/UBC/UPF2/NBAS/BOLL/PAN3/SMG1/PSMD4/RPL26/RPL9/RPS14/SMG8/RPL13/UPF3A/EXOSC1/PAIP1/CSDC2/RPL15/PDE12/LSM1/RPLP2/ERN1/MEX3D/CNOT10/TENT5C/PSMD13/NANOS3/PLEKHN1/NANOS2/RPL12/RPL23A/YTHDF2/MTOR/SMG5/SKIV2L/LSM2/PSMB11/RPL41/RPS18/RPS28/SLFN14/MYEF2/FASTKD1/RNASEH2B/ELAVL4/AGO2/ANP32A/MAGOHB/TBRG4/DAZL/ISG20/RBM46/PSMD2/SUPV3L1/RPL31/YWHAB/DIS3L/RC3H2/ZC3H14/HNRNPC/AKT1/PSMA2/PSME1/PSMD11/EDC3/MOV10/NT5C3B/PSMD6/PSMB7/RPS20	153
GO:0016052	carbohydrate catabolic process	81/5495	200/18866	0.000355	0.03648	0.0318	SLC4A1/TKTL1/MLXIPL/MAN2B2/NUP160/PHKA2/TPR/HEXB/PFKP/PKM/HDAC4/ENO1/SIRT6/SLC4A4/SEH1L/P2RX7/PYGL/PHKB/NUP93/MAN2B1/GAPDHS/OGDH/PRKAG2/ENO3/NUP88/ALDOC/NUP98/ENO2/NUP155/PFKFB4/NUP153/NUP85/NUP214/CBFA2T3/ENOSF1/PRKAA1/NUP42/NCOR1/PFKL/PGD/ARNT/PHKG2/HK3/JMJD8/LRP5/PHKG1/FOXK1/DDIT4/PGM2/GLB1/PFKFB3/INSR/BPGM/FUT1/GK5/ZBTB7A/ZBTB20/PPARA/GK/NAGA/NEU1/GALT/RPEL1/EIF6/OGT/PPP1CA/SCARB2/IFNG/PFKM/RAE1/MYOG/PGM1/PPP1R3B/BAD/NUP188/PPP1R3D/NEU4/IDNK/KH	81
GO:0006096	glycolytic process	51/5495	115/18866	0.000361	0.0365	0.0318	SLC4A1/MLXIPL/NUP160/TPR/PFKP/PKM/HDAC4/ENO1/SIRT6/SLC4A4/SEH1L/P2RX7/NUP93/GAPDHS/OGDH/PRKAG2/ENO3/NUP88/ALDOC/NUP98/ENO2/NUP155/PFKFB4/NUP153/NUP85/NUP214/CBFA2T3/PRKAA1/NUP42/NCOR1/PFKL/ARNT/HK3/JMJD8/FOXK1/DDIT4/PFKFB3/INSR/BPGM/ZBTB7A/ZBTB20/PPARA/EIF6/OGT/IFNG/PFKM/RAE1/MYOG/PGM1/NUP188/PGK2	51
GO:1990090	cellular response to nerve growth factor stimulus	26/5495	49/18866	0.000371	0.03699	0.0322	TAC1/KIF1B/EEF2K/STMN2/RAPGEF1/KAT2A/EHD1/RAP1A/NGF/DYNC1LI2/PDPK1/EIF4A3/CRK/EEF2/CIB1/MAPT/TRPV1/NTF4/UBE3A/NTRK3/SH3GL2/NTF3/AKT1/ARF6/WASF1/PTEN	26
GO:0010847	regulation of chromatin assembly	14/5495	21/18866	0.000402	0.03749	0.0326	TPR/SIRT6/APOBEC1/MORC2/SETDB1/TASOR/PHF8/PHF2/MPHOSPH8/AICDA/TET1/SETDB2/PARP10/ZNF304	14
GO:0035357	peroxisome proliferator activated receptor signaling pathway	14/5495	21/18866	0.000402	0.03749	0.0326	PDK3/PTGIS/PPARG/ASXL2/ALOX15/ASXL1/LEP/RXRA/PLIN5/STARD10/MED1/LMO3/FAM120B/CITED2	14
GO:0048015	phosphatidylinositol-mediated signaling	78/5495	192/18866	0.000404	0.03749	0.0326	FYN/PIK3C2A/HGF/GAB2/PREX2/RHOA/EDN1/PITPNM2/TYRO3/JAK2/PLCB4/TSC2/MAZ/NCS1/CRYBA1/C1QBP/SLC9A3R1/NPR3/PDGFRB/PLCH1/CD160/TEK/CAT/FLT3/BTN2A2/PIK3C2B/NGF/PDGFRA/PI4KB/PIP5K1A/EGFR/PLCH2/FBXL2/PLCL2/PLCD3/PTPN13/GPER1/SERPINA12/PIP4K2C/NYAP1/INSR/PIK3CD/LEP/F2/CSF1R/PLCB1/PIP5K1C/SRC/PIK3CA/OGT/FLT1/SEMA4D/FSHR/ERBB4/UBE3A/NTRK3/EXOC1/PIK3CB/PEAR1/ERBB2/INPP5E/NTF3/IRS2/GAB1/PDGFA/PIK3C3/CA8/PLCB3/AKT1/LYN/PLEKHA1/GSN/CRNN/PPP1R16B/PLCD1/PTEN/PRR5/MUC5AC	78

GO:0007044	cell-substrate junction assembly	45/5495	99/18866	0.000404	0.03749	0.0326	EPHA3/LAMA3/LIMCH1/SLK/RHOA/ROCK1/TESK2/MAP4K4/CAMSAP3/TRIP6/ABL1/NRP1/ARHGEF7/DUSP22/HRG/TEK/MACF1/LAMC1/TLN1/PDPK1/BCAS3/PIP5K1A/WDPCP/PTPRJ/TAOK2/DST/PTPRK/DMTN/CLASP2/DLC1/DAPK3/RHOD/PEAK1/CD151/EFNA5/KRT14/SRC/TNS1/LAMB3/CLASP1/KRT5/VEGFA/FAM107A/PTEN/MMP14	45
GO:0007613	memory	53/5495	121/18866	0.000405	0.03749	0.0326	TAC1/ABCA7/PRKCZ/CRTC1/PTN/SLC6A4/KAT2A/MDK/SRF/HRH2/DBH/NQO2/ATXN1/LDLR/SERPINF1/SYT11/NGF/PAK6/PLK2/VLDLR/EIF4EBP2/ADCY8/INSR/NPAS4/RIN1/PLCB1/BTBD9/MAPT/MTOR/ATXN1L/NTF4/SCN2A/CHRNA7/ADCY1/ITGA3/DRD2/PPP3CB/NTF3/MAP1A/B4GALT2/KCNK2/NETO1/CCK/MUSK/SLC6A1/SLC24A2/KCNK10/ATAD1/OXTR/PTCHD1/PPP1R1B/PTEN/ARC	53
GO:0046031	ADP metabolic process	54/5495	124/18866	0.000427	0.03827	0.0333	SLC4A1/MLXIPL/NUP160/TPR/PFKP/PKM/HDAC4/ENO1/SIRT6/SLC4A4/SEH1L/P2RX7/NUP93/GAPDHS/OGDH/PRKAG2/ENO3/NUP88/ALDOC/NUP98/ENO2/NUP155/PFKFB4/NUP153/NUP85/NUP214/CBFA2T3/PRKAA1/NUP42/NCOR1/PFKL/ARNT/AK3/HK3/JMJD8/FOXK1/DDIT4/PFKFB3/INSR/BPGM/ZBTB7A/ZBTB20/PPARA/EIF6/OGT/IFNG/AK5/PFKM/RAE1/MYOG/PGM1/BAD/NUP188/PGK2	54
GO:0051168	nuclear export	82/5495	204/18866	0.000434	0.03827	0.0333	UPF1/AKAP8L/NUP160/RANBP3/NSUN2/TPR/SMG6/PRKACA/THOC1/STRADB/XPO1/YTHDC1/SEH1L/POLDIP3/RANGAP1/NUP93/NUP88/YWHAE/NUP98/NUP155/NCBP2/RPS15/SMG7/CPSF3/GLE1/KHDRBS1/XPO5/ATXN1/NUP153/NUP85/THOC2/PCID2/NUP214/LTV1/DHX9/NUP42/TXN/NCBP1/EIF4A3/UPF2/PTPN14/UHMK1/SMG1/DDX19B/ANKLE1/MCM3AP/CHTOP/SRSF2/SLBP/NOL6/DDX19A/XPO6/AKAP13/SETD2/GAS6/XPOT/CHP1/SMG5/CCHCR1/EIF6/WDR33/NEMF/SLU7/MAGOHB/PPM1A/CPSF2/THOC5/SRRM1/NOP9/XPO7/NMD3/RBM26/RAN/IWS1/AGFG1/RAE1/IFI27/NUP188/CDC40/EGR2/THOC6/GTSE1	82
GO:0048193	Golgi vesicle transport	139/5495	374/18866	0.000435	0.03827	0.0333	ARF5/VAMP3/USE1/AP3D1/KIF26A/BCAP29/KIFAP3/KLHL20/DYNC1I2/CAPZB/SAR1A/DNM2/KIF3C/CD59/SCAMP1/EPS15/DYNLL1/RNF215/PICK1/SEC23B/VAPA/GGA2/VPS35L/SNX8/GOSR2/GOSR1/SNX3/EXOC2/GOLPH3/COL7A1/NRBP1/PROC/KLHL12/TMED5/SEC16B/COPA/CCDC91/BBS2/C16orf70/TBC1D20/ERGIC3/TMEM115/MACF1/COPB1/EXOC4/TBC1D14/NAPG/SPIRE1/RINT1/DYNC1LI2/STX6/KDELR2/DCTN3/KIF13A/KIF11/GOLPH3L/CNIH4/CNIH3/DYNC1LI1/GOLGA4/ANK2/VPS51/NBAS/MPPE1/RAB6B/KIF5A/STEAP2/COPG2/TGFA/COG5/VCP/COG1/AP1G1/DCTN5/GOLGA2/ARF4/COG7/RAB31/STX18/VAMP5/TMED10/TRAPPC12/COMMD1/BBS1/YIF1A/CNIH2/RAB1B/KLC2/SEC24C/BET1L/GAK/F2/GAS6/RABIF/ANKFY1/CLN3/MYO18A/HTT/SPTAN1/DCTN1/ANKRD28/AP1G2/VPS52/CTAGE8/PDCD6/CORO7/SORL1/BET1/KIF23/VPS13C/SEC16A/RAB8A/EXOC1/GOLGA3/CAPZA2/TFG/ACSL3/SEC22C/GGA1/PITPNB/KIF2C/MIA3/TRAPPC4/COPZ1/KIF2A/TMED3/LYPLA1/F7/BICD2/RER1/DYNC	139
GO:0031445	regulation of heterochromatin assembly	13/5495	19/18866	0.000446	0.03827	0.0333	TPR/SIRT6/APOBEC1/MORC2/SETDB1/TASOR/PHF8/PHF2/MPHOSPH8/AICDA/TET1/SETDB2/ZNF304	13
GO:1903311	regulation of mRNA metabolic process	129/5495	344/18866	0.000453	0.03827	0.0333	RBM3/UPF1/PAF1/CDK11A/PTBP1/PSMC4/CELF2/PTCD2/THRAP3/PUM2/ROCK1/PSME4/JMJD6/DAZAP1/IGF2BP2/TNRC6C/XPO1/YTHDC1/KHSRP/FUS/TNRC6A/SRPK1/CIRBP/HNRNPM/DDX17/RNF40/CACNG7/HSPB1/EXOSC3/C1QBP/PRPF19/NUP98/APOBEC1/MAPK14/TENT4A/FAM172A/NCBP2/TIA1/SET/TENT4B/KHDRBS1/SMU1/FASTKD3/CNOT1/PCID2/NUP214/PSMA1/SAFB2/CDC73/DHX34/SRPK2/ZC3H10/DHX9/RC3H1/NCBP1/SLTM/PNPT1/HNRNPD/CELF6/PARN/FTO/PSMB6/SF3B4/CELF1/SAP18/UBC/NBAS/BOLL/SREK1/PSMD4/SAFB/SRSF2/CWC22/NUDT21/EXOSC1/PAIP1/CSDC2/PDE12/ZBTB7A/MEX3D/TENT5C/SRPK3/PSMD13/NANOS3/PLEKHN1/NANOS2/YTHDF2/CTR9/MTOR/PSMB11/CDK11B/MBNL3/MYEF2/FASTKD1/ELAVL4/AGO2/SRRM4/ANP32A/RBM23/TBRG4/DAZL/MYOD1/SON/RBM46/PSMD2/RBM17/KHDRBS3/YWHAB/QKI/RC3H2/ZC3H14/HNRNPC/AKT1/PSMA2/SART3/IWS1/PSME1/SFSWAP/PSMD11/MOV10/PSMD6/PSMB7/PRKCD/PSME2/RBM25/C9orf78/METTL3/POLR2G/PS	129
GO:0006405	RNA export from nucleus	61/5495	144/18866	0.000463	0.03827	0.0333	UPF1/AKAP8L/NUP160/NSUN2/TPR/SMG6/THOC1/XPO1/YTHDC1/SEH1L/POLDIP3/NUP93/NUP88/NUP98/NUP155/NCBP2/RPS15/SMG7/CPSF3/GLE1/KHDRBS1/XPO5/NUP153/NUP85/THOC2/PCID2/NUP214/LTV1/DHX9/NUP42/NCBP1/EIF4A3/UPF2/SMG1/DDX19B/MCM3AP/CHTOP/SRSF2/SLBP/NOL6/DDX19A/SETD2/XPOT/SMG5/EIF6/WDR33/SLU7/MAGOHB/CPSF2/THOC5/SRRM1/NOP9/NMD3/RBM26/RAN/IWS1/AGFG1/RAE1/NUP188/CDC40/THOC6	61
GO:0006757	ATP generation from ADP	51/5495	116/18866	0.000464	0.03827	0.0333	SLC4A1/MLXIPL/NUP160/TPR/PFKP/PKM/HDAC4/ENO1/SIRT6/SLC4A4/SEH1L/P2RX7/NUP93/GAPDHS/OGDH/PRKAG2/ENO3/NUP88/ALDOC/NUP98/ENO2/NUP155/PFKFB4/NUP153/NUP85/NUP214/CBFA2T3/PRKAA1/NUP42/NCOR1/PFKL/ARNT/HK3/JMJD8/FOXK1/DDIT4/PFKFB3/INSR/BPGM/ZBTB7A/ZBTB20/PPARA/EIF6/OGT/IFNG/PFKM/RAE1/MYOG/PGM1/NUP188/PGK2	51
GO:0032970	regulation of actin filament-based process	149/5495	405/18866	0.000466	0.03827	0.0333	TAC1/WAS/EPHA3/PKP2/LIMCH1/CNN2/RHOA/ROCK1/GBA2/WDR1/ADD2/CAPZB/EDN1/ADD1/ASAP3/ARHGAP28/PXN/ICAM1/PLEKHG2/DSP/ABL1/NRP1/PICK1/MYH9/HCK/ARHGEF10/CAV1/MET/ACTR3C/LIMK1/KANK1/TESK1/MDK/ARHGDIB/SEMA5A/PDE4D/PDGFRB/HRG/ACTR3/PLEK/APOA1/CCN2/RHOQ/TEK/VASP/MKKS/CCR7/AKAP9/CGNL1/BST2/GMFG/HIP1R/INPP5K/SWAP70/SPIRE1/PDGFRA/AVIL/GATA4/TMOD1/CCL21/FCHSD2/ABI2/FRMD6/ARHGAP17/MYLK3/SSH2/BCAS3/ABL2/ANK2/C9orf72/PAK1/ESAM/TAOK2/DIXDC1/FER/EPS8/F11R/DMTN/CARMIL2/TAOK1/ALOX15/LMOD3/CLASP2/RICTOR/DLC1/CRK/DAPK3/SERPINF2/RHOH/AKAP13/PTGER4/SYNPO/RHOD/ATP2A2/HRAS/PPM1E/KCNE3/FLII/CDC42EP4/CSF1R/EFNA5/NF2/ARAP1/FLNA/SPTAN1/MYO1C/CD2AP/MTOR/ARPC4/CDC42EP3/NOTCH2/SSH1/SMAD4/CTNNA2/ECT2/FHOD3/PPFIA1/ARHGAP44/NTRK3/CAPZA2/CYRIA/SCN5A/ABITRAM/ATP1A1/CDC42EP1/NTF3/PDGFA/ATP1A2/CLASP1/ARPC5L/BAIAP2/CXCL12/KCNJ2/MYLK2/CELSR1/ARFIP1/RNF207/GSN/ARF6/ARHGAP40/PR	149
GO:0031532	actin cytoskeleton reorganization	47/5495	105/18866	0.000467	0.03827	0.0333	RALA/DTNBP1/BCAR1/RHOA/ABL1/NRP1/MYH9/HCK/MDK/ARHGDIB/NEDD9/PHACTR1/PTK7/PLEK/TEK/GMFG/PDLIM4/PDGFRA/BCAS3/ABL2/PIP5K1A/PAK1/ESAM/FER/EPS8/TNIK/F11R/DMTN/RICTOR/DAPK3/RHOD/HRAS/CSF1R/ARAP1/FLNA/PARVA/CD2AP/NOTCH2/NTRK3/CASS4/NTF3/MCU/GAB1/BAIAP2/CAPN10/GSN/GPR65	47

GO:0022407	regulation of cell-cell adhesion	160/5495	439/18866	0.000484	0.03918	0.0341	HFE/FYN/CD44/WNK1/PTPRU/AP3D1/RHOA/PRKCZ/CBFB/FSTL3/TFRC/KIFAP3/PAG1/IL4R/ARG2/PTPRC/CYLD/CEACAM6/ICAM1/JAK2/ABL1/LGALS1/XBP1/JAG1/NFAT5/PIEZO1/DNAJA3/LILRB1/RASAL3/EBI3/CAV1/AKNA/DOCK8/ZBTB16/MDK/IL23A/GCNT2/RIPOR2/MAPK14/CD83/VNN1/DUSP22/CD86/HHLA2/PLA2G2D/CD160/APOA1/CD274/CD80/LAX1/PCK1/BTN2A2/SOX4/TNFSF9/CD70/TNFSF14/CCR7/ZBTB1/ADORA2A/TWSG1/NDFIP1/LGALS3/AP3B1/SWAP70/IL2RA/RC3H1/IL10/CCL21/IL21/PDPK1/SKAP1/VAV1/ZC3H8/MYO10/MEGF10/PAK1/MAGI1/CCL28/BMP6/F11R/DMTN/TNFRSF13C/ALOX15/IL23R/VCAM1/TGFBR2/ICOS/SERPINF2/PRDX2/FADD/RHOH/IL7R/FGA/RELA/LEP/DENND6A/GLMN/PTPN2/SART1/YES1/SOX12/EPHB3/ABAT/ZNF703/IRAK1/EFNA5/NF2/PPARA/HLA-DRB1/SRC/PELI1/YTHDF2/CD3E/AGER/CDSN/HLA-E/TARM1/IDO1/IL12RB1/PIK3CA/IFNG/IL12B/ZAP70/LCK/WNT5A/ADAM8/ICOSLG/ERBB2/ITPKB/ETS1/MIA3/RC3H2/FLOT2/GPAM/PODXL/AKT1/NOTCH1/EGR3/AFDN/CXCL12/TNR/BMP7/TNFRSF14/BLK/FOXA2/SHH/LYN/PNP/BAD/ALOX5/VEGFA/PRKCD/SFTPD/PLG/IL4/HLA-	160
GO:0048017	inositol lipid-mediated signaling	79/5495	196/18866	0.000495	0.0396	0.0345	FYN/PIK3C2A/HGF/GAB2/PREX2/RHOA/EDN1/PITPNM2/TYRO3/JAK2/PLCB4/TSC2/MAZ/NCS1/CRYBA1/C1QBP/SLC9A3R1/NPR3/PDGFRB/PLCH1/CD160/TEK/CAT/FLT3/BTN2A2/PIK3C2B/NGF/PDGFRA/PI4KB/PIP5K1A/EGFR/PLCH2/FBXL2/PLCL2/PLCD3/PTPN13/GPER1/SERPINA12/PIP4K2C/NYAP1/INSR/PIK3CD/LEP/F2/CSF1R/PLCB1/TNFAIP8L3/PIP5K1C/SRC/PIK3CA/OGT/FLT1/SEMA4D/FSHR/ERBB4/UBE3A/NTRK3/EXOC1/PIK3CB/PEAR1/ERBB2/INPP5E/NTF3/IRS2/GAB1/PDGFA/PIK3C3/CA8/PLCB3/AKT1/LYN/PLEKHA1/GSN/CRNN/PPP1R16B/PLCD1/PTEN/PRR5/MU	79
GO:0006352	DNA-templated transcription, initiation	97/5495	249/18866	0.00051	0.03987	0.0347	CREBBP/MED24/PSMC4/NR1H3/RSF1/CBFB/RORA/TEAD2/BAZ2A/RRN3/BAX/POLR2E/POLRMT/POLR2F/POLR3H/HNF4A/MAZ/MED25/MED26/POLR2I/UBTF/GTF2H1/E2F3/SRF/SUB1/KAT2B/NRBP1/POLR1G/CCN2/NR2C1/MED20/GTF2F1/THRA/SMARCA4/NR1H2/PPARG/MED10/CDK7/HNF1A/CDK4/POLR1E/YAP1/GTF2A2/NR1I2/BDP1/TAF1/POLR2K/H4C8/MED27/TFB2M/GTF2A1/TAF3/TAF10/RBPJ/PPM1D/POLR1C/NR1D2/NPPA/MED16/NR2C2/POLR2L/CTNNBIP1/POLR2A/MAML2/RXRA/PPARA/TEAD1/MITF/H4C3/GTF2E2/TEAD4/CRCP/GTF2A1L/TAF4B/NOTCH2/ESRRA/PAXIP1/TAF7L/RORC/NR2F1/RXRG/MED1/ERCC3/MED17/RARG/MED6/NOTCH1/NR5A1/TAF2/BCLAF1/CCNC/GTF2B/TAF4/PTEN/POLR1F/POL	97
GO:0006400	tRNA modification	41/5495	89/18866	0.000511	0.03987	0.0347	NSUN2/METTL1/TRIT1/HSD17B10/TRMT6/AARS1/PUS7/CDK5RAP1/MTFMT/DTWD1/DUS4L/TRDMT1/ELP4/PUS3/TRMT1L/TRMT13/GTPBP3/NAT10/DUS3L/CDKAL1/ELP6/METTL2B/TRMT10B/LCMT2/PUSL1/ELP5/CTU2/TRMT12/QTRT1/TRMU/THG1L/THUMPD3/TRMT5/ELP2/ELP1/PUS1/WDR4/ALKBH1/URM1/OSGEP/KTI12	41
GO:0050777	negative regulation of immune response	66/5495	159/18866	0.000545	0.04165	0.0363	HFE/NR1H3/FAM3A/IL4R/THOC1/ARG2/PTPRC/CD59/TYRO3/TTLL12/LILRB1/CACTIN/RPS19/DUSP22/IL1RL1/CD160/APOA1/PSMA1/BST2/NR1H2/NDFIP1/LGALS3/PPARG/IL2RA/TRAFD1/RC3H1/IL10/IL33/NLRC5/ARRB2/SERPING1/FER/CD96/ABR/NLRX1/PGLYRP2/ALOX15/FCRLB/DCST1/IFI16/STAT6/CRK/IL7R/SERPINB9/PTPN2/A2M/HLA-DRB1/YTHDF2/HLA-E/ENPP3/IL12B/COL3A1/DRD2/PPP3CB/RC3H2/PARP14/C4BPA/HLA-	66
GO:0010810	regulation of cell-substrate adhesion	86/5495	217/18866	0.000547	0.04165	0.0363	EPHA3/LIMCH1/SLK/RHOA/PRKCZ/ROCK1/MAP4K4/CAMSAP3/DNM2/CEACAM6/JAK2/ABL1/NRP1/CRKL/LGALS1/JAG1/ARHGEF7/OLFM4/PTN/KANK1/C1QBP/MDK/GCNT2/NEDD9/DUSP22/HRG/NID1/APOA1/TEK/RREB1/CCR7/MACF1/RIN2/POSTN/CCL21/SKAP1/BCAS3/WDPCP/CASK/DOCK5/CDKN2A/HSD17B12/PTPRJ/CCL28/SPOCK1/DMP1/UTRN/FZD7/DMTN/ALOX15/DISC1/CLASP2/DLC1/CRK/DAPK3/PLEKHA2/ONECUT1/FGA/RHOD/PEAK1/FUT1/ACER2/EFNA5/CIB1/NF2/FLNA/SRC/CD3E/DDR1/VIT/EMP2/ADAM15/STK4/ITGA3/PIK3CB/CASS4/CLASP1/NOTCH1/ECM2/SMOC2/VEGFA/TACSTD2/PLG/FAM107A/PTEN/MMP14	86
GO:0031032	actomyosin structure organization	80/5495	200/18866	0.000603	0.04438	0.0386	TAC1/WAS/LIMCH1/CNN2/RHOA/ROCK1/MEF2A/WDR1/EDN1/EPB41L3/ASAP3/ARHGAP28/PXN/TRPM7/ABL1/NRP1/MYH9/ARHGEF10/MYH14/MET/LIMK1/TESK1/PDLIM1/TNFAIP1/PHACTR1/SRF/PDGFRB/APOA1/CCN2/MKKS/CGNL1/CNN1/INPP5K/VPS4A/MYH11/PDGFRA/TMOD1/FRMD6/MYLK3/PAK1/OBSCN/F11R/EPB41/LMOD3/CLASP2/DLC1/SERPINF2/PDCD6IP/AKAP13/PTGER4/KCTD13/PPM1E/CSRP2/FLII/NF2/ARAP1/MYO18A/SRC/MYH6/MTOR/KLHL41/SMAD4/ECT2/FHOD3/PPFIA1/KIF23/EPB41L1/EPB41L4B/EPB41L2/LURAP1/TNNT2/CLASP1/TNNT1/LDB3/TACSTD2/LPAR1/SORBS3/TCAP/GPR65/CAV3	80
GO:0032147	activation of protein kinase activity	124/5495	331/18866	0.000603	0.04438	0.0386	MAP4K3/GPRC5A/HGF/TAB2/EIF2AK2/WNK1/KARS1/PRKCZ/MAP4K4/PRKACA/TRAF4/MAP2K7/PTPRC/STRADB/MAPKAPK5/P2RX7/OSBPL8/PPP2R3C/JAK2/ABL1/GADD45B/CRKL/TAB1/AXIN1/TNFRSF10A/PRKAG2/RAPGEF1/MADD/IL23A/MAPK14/GHR/FGF1/CD86/PRKAR2A/STK11/KIF14/TNFRSF10B/MAP2K2/PAK4/DUSP9/PRKAA1/IRAK2/NGF/AGAP2/TXN/RIPK1/PAK6/MAPK8IP3/PDPK1/CARD14/RPTOR/PROK1/EGFR/PAK1/TAOK2/UBC/TNIK/ADCY8/TAB3/TAOK1/IL6R/DBF4B/IL23R/PEA15/TGFA/TGFBR2/RICTOR/CRK/PRDX2/MLKL/INSR/ADCY5/CSPG4/TLR6/LEP/ATG13/ERN1/DYNAP/GAS6/IRAK1/EFNA5/PRKAR1B/SRC/MAP3K5/MTOR/EMP2/ITGA1/UBE2V1/ALK/PIK3CA/ADCY4/ECT2/CHRNA7/SPDYA/TRAF2/STK4/ADCY1/IL12B/NTRK3/WNT5A/DRD2/PIK3CB/GRM4/NTF3/PRKACB/LPAR3/PINK1/AKT1/ANGPT4/MARK2/TRAF7/MAP2K3/ADCY6[illegible]	124
GO:0051236	establishment of RNA localization	81/5495	203/18866	0.000611	0.04438	0.0386	UPF1/AKAP8L/MVP/NUP160/NSUN2/TPR/SMG6/SIDT1/IGF2BP2/THOC1/XPO1/YTHDC1/SEH1L/KHSRP/POLDIP3/NUP93/NUP88/NUP98/NUP155/NCBP2/RPS15/SMG7/CPSF3/GLE1/KHDRBS1/XPO5/NUP153/NUP85/THOC2/PCID2/NUP214/RFTN1/TERF2/LTV1/DHX9/NUP42/NCBP1/PNPT1/EIF4A3/TERF1/ATM/UPF2/SMG1/DDX19B/MCM3AP/CHTOP/SRSF2/TGFBR2/EIF5A2/SLBP/NOL6/KIF5C/DDX19A/UPF3A/CKAP5/SETD2/XPOT/MYO1C/SMG5/EIF6/WDR33/SLU7/MAGOHB/CPSF2/THOC5/SRRM1/NOP9/G3BP2/NMD3/QKI/RBM26/RAN/IWS1/AGFG1/BICD2/RAE1/MX2/NUP188/CDC40	81

GO:1903532	positive regulation of secretion by cell	118/5495	313/18866	0.000613	0.04438	0.0386	CD38/TAC1/NADK/GIPR/HFE/GAB2/CASK/DTNBP1/SLC4A8/TM7SF3/GAL/PLA2G10/TSG101/IL4R/EDN1/C1QTNF3/OXCT1/ACHE/DNM1L/P2RX7/JAK2/XBP1/MYOM1/PARD6A/RAB3D/RAB3A/GHRHR/NCS1/SLC6A4/GOLPH3/RAB3GAP1/SPP1/RAB9A/SOX4/ADORA2A/UNC13A/NR1H2/ANO1/VPS4A/IL10/STAM/IL33/TLR2/RAB15/SIRT3/GOLPH3L/TMF1/SNCA/EGFR/VEGFC/BMP6/ADCY8/SNF8/CLASP2/GPER1/AP1G1/PRRT2/NLGN1/ITGAM/NLGN2/PDCD6IP/PTGER4/FGA/C2CD2L/LEP/GLMN/NPPA/HCAR2/CADM1/ABAT/FFAR4/HLA-DRB1/PPIA/MYO18A/SRC/CD2AP/UNC13B/HLA-E/ITSN1/SORL1/INHBA/CADPS/SMAD4/IFNG/SLC30A8/RAB27B/SERP1/DRD2/ADAM8/KIF5B/EXOC1/AACS/DOC2B/CLEC5A/PPP3CB/IRS2/MCU/SDCBP/CACNA1H/HLA-E/SYT10/PINK1/CCK/CLASP1/SYBU/CXCL12/CAPN10/CLEC9A/BLK/FO	118
GO:0140014	mitotic nuclear division	109/5495	286/18866	0.000617	0.04438	0.0386	NCAPD2/AKAP8L/TACC3/TPR/ANAPC4/CDK13/RHOA/SMC1A/EDN1/CDC14A/NDC80/PDS5B/SEH1L/CHMP5/NSFL1C/ANAPC5/BIRC5/HIRA/VRK1/MYBL2/CEP192/NAA10/RAB11A/ARHGEF10/AKAP8/CAV2/TENT4A/MACROH2A1/PDGFRB/STAG1/KIF14/TEX14/NAA50/PDS5A/PCID2/PSMG2/INO80/VPS4A/USP44/KIF11/KIF20B/POGZ/DIS3L2/DYNC1LI1/ATM/DSN1/EPS8/DRD3/CCNB2/CDC25C/NPM2/LRP5/TGFA/CLASP2/SPICE1/NIPBL/PLK1/GOLGA2/DAPK3/INSR/PPP2R2D/TOM1L2/AURKAIP1/CTDNEP1/SPHK1/SH2B1/CHEK2/KNTC1/L3MBTL1/TOGARAM2/ANAPC7/FLNA/LCMT1/KIFC1/PTTG2/CHMP1B/GEN1/NCAPD3/KIF23/PIN1/SGO2/BUB3/CDCA8/SPDL1/RB1/LPIN1/KIF2C/MKI67/KIF2A/CDC14B/REC8/XRCC3/RAN/KIFC2/CLASP1/CHEK1/BMP7/DUSP1/WRAP73/SMC2/NCAPH/PRMT5/SMC3/CHAMP1/PPP1R9B/XIAP/CENPC/EPN3/MZT1	109
GO:0018212	peptidyl-tyrosine modification	139/5495	377/18866	0.000638	0.04541	0.0395	CKLF1/FYN/MVP/GPRC5A/HGF/CD44/EPHA5/KITLG/EIF2AK2/FGFR2/PRKCZ/HYAL2/FGFR3/TESK2/TSG101/TXK/MAP2K7/PTPRC/IL12RB2/DLG3/ICAM1/TYRO3/JAK2/ABL1/NRP1/HCK/ARL2BP/DYRK1B/CAV1/MET/TESK1/HPX/HIPK3/CAMKK2/IL23A/NEDD9/DUSP22/GHR/THBS4/PDGFRB/HRG/STK16/ARHGEF2/TEK/CD80/FLT3/MAP2K2/DYRK2/SH3BP5/DLG4/DSTYK/LRP4/PDGFRA/LACRT/GH2/ABI1/ABI2/IL21/ARRB2/ABL2/DYRK3/EGFR/PTPRJ/FCGR1A/FER/BMP6/DMTN/IL6R/IL23R/DDR2/IL24/TGFA/HIPK1/RICTOR/MELK/AXL/TPST1/INSR/PEAK1/CSPG4/LEP/PTPN2/YES1/STAP2/SOCS4/CSF1R/EPHB3/GAS6/EFNA5/NF2/TNFRSF18/SRC/MTOR/CD3E/DDR1/WEE2/ALK/CLK3/TPST2/FLT1/IFNG/SEMA4D/IL12B/ERBB4/ZAP70/LCK/NTRK3/EPHA8/CADM4/SLA/EPHB1/AATK/ERBB2/CASS4/NTF3/EPHB2/TAL1/PDGFA/CEP43/MST1R/PARP14/CCK/ANGPT4/CLK1/MUSK/FRK/EPHA10/TNFRSF14/MAP2K3/BLK/LYN/SH3BP5L/VEGFA/PRKCD/DOK7/TREM2/IL4/HES5/ADRA2A	139
GO:0031331	positive regulation of cellular catabolic process	143/5495	390/18866	0.000703	0.04949	0.0431	UPF1/OSBPL7/MLXIPL/PIK3C2A/PSMC4/CLEC16A/VPS13D/MAPK9/ROCK1/SIRT6/TNRC6C/ITCH/KEAP1/SLC4A4/MID2/ULK2/HUWE1/BAX/KHSRP/P2RX7/SPTLC1/TNRC6A/TRIB3/AXIN1/TSC2/RNF40/BNIP3L/SGTA/FZR1/CDC37/GAPDHS/CAV1/DVL1/APOA5/APOA4/AMBRA1/CAMKK2/TRIM38/NNT/CSNK1A1/PFKFB4/RAB3GAP1/MFN2/STK11/APOA1/FBXL5/RAB3GAP2/CNOT1/TICAM1/CBFA2T3/LDLR/DDA1/TRIM5/PRKAA1/EDEM1/LACRT/RC3H1/IL33/RNF144B/PNPT1/HNRNPD/FTO/COP1/ARNT/VGLL4/SNCA/PLK2/TAF1/C9orf72/ATM/DAB2/PHKG2/KLHL40/VPS11/DISC1/DTX3L/VCP/PACSIN3/PLK1/PIP4K2C/FBXO22/PAFAH1B2/PFKFB3/INSR/MAGEF1/ARIH2/GBA/PNPLA2/SOCS4/MEX3D/ZBTB20/TMEM259/TBK1/PPARA/NANOS3/PLEKHN1/NANOS2/RNFT1/HTT/ATG7/YTHDF2/RAB12/PLIN5/IRGM/AGO2/SH3RF2/IFNG/ATG5/VPS13C/EPM2A/MSN/ADAM8/SUPV3L1/PIK3CB/ULK1/AKT2/IRS2/TRIM68/SH3GLB1/RCHY1/PINK1/AKT1/TRIM65/TRIB2/HMOX1/MOV10/CHFR/AADAC/SH3BP4/ZER1/B	143
